# Deep divergence between island populations in lichenized fungi

**DOI:** 10.1038/s41598-021-86448-z

**Published:** 2021-04-01

**Authors:** Silke Werth, Peter Meidl, Christoph Scheidegger

**Affiliations:** 1 Systematic Botany and Mycology, Ludwig-Maximilians Universität München, Menzingerstraße 67, 80638 Munich, Germany; 2 Department of Life and Environmental Sciences, University of Iceland, Sturlugata 7, 101 Reykjavik, Iceland; 3 Swiss Federal Research Institute WSL, Zürcherstrasse 111, 8903 Birmensdorf, Switzerland

**Keywords:** Population genetics, Genetic variation

## Abstract

Macaronesia is characterized by a high degree of endemism and represents a noteworthy system to study the evolutionary history of populations and species. Here, we compare the population-genetic structure in three lichen-forming fungi, the widespread *Lobaria pulmonaria* and two Macaronesian endemics, *L. immixta* and *L. macaronesica,* based on microsatellites. We utilize population genetic approaches to explore population subdivision and evolutionary history of these taxa on the Canary Islands, Madeira, Azores, and the western Iberian Peninsula. A common feature in all species was the deep divergence between populations on the Azores, a pattern expected by the large geographic distance among islands. For both endemic species, there was a major split between archipelagos. In contrast, in the widespread *L. pulmonaria*, divergent individuals were distributed across multiple archipelagos, suggesting a complex evolutionary history involving repeated migration between islands and mainland.

## Introduction

The study of large-scale geographic genetic patterns can provide valuable insight regarding the evolutionary history of taxa. Archipelagos of volcanic origins are of a particular interest because one can safely assume the absence of habitat connectivity between islands. Exceptions exist in areas where sea levels have receded, leading to connectivity between islands surrounded by shallow sea. Volcanic islands are fascinating systems to study owing to their isolation from large mainland populations, and their high degree of endemism^1–4^. Many of the world’s volcanic island systems are characterized by radiations in specific genera^5, 6^. In volcanic islands, one main driver of environmental change is the erosion stage of the island, which creates substantial environmental change on a time scale of millions of years and can lead to massive altitudinal differences and hence, to differences in plant communities^7^.

One of the most distinctive places in the world to study the evolutionary history of taxa is Macaronesia, a group of volcanic archipelagos in the Atlantic Ocean, consisting of the Azores, Madeira and nearby islands, the Canary Islands, the Selvagem Islands and the Cape Verde Islands. Macaronesia harbors a highly diverse biota, comprising many endemics^4^. Island groups within Macaronesia have been the cradle of recent radiations in several plant and fungal genera^4–6, 8–10^. Thus, due to its complex, but well-known volcanic history, its geographic isolation from mainland sites, its high diversity and high degree of endemism, Macaronesia has for a long time been a major playground for evolutionary biologists and biodiversity researchers.

However, few prior studies have investigated the genetic relationships among lichenized fungi on the Macaronesian islands. Tehler et al.^11, 12^ investigated the genus *Roccella* in North America and Europe including Macaronesia and found that North American and Macaronesian species formed sister clades. Only a single species in the genus, *R. elisabethae*, was found to be endemic to Macaronesia. Sérusiaux et al.^4^ found that multiple lichen fungi belonging to the genus *Nephroma* were neoendemics which originated in Macaronesia and subsequently spread to the European continent.

We used six fungal nuclear microsatellite loci to investigate the geographic genetic patterns in three lichen fungi, the wide-spread *Lobaria pulmonaria* and two closely related taxa, the Macaronesian endemics *L. immixta* and *L. macaronesica*, which are sister taxa^13, 14^. *Lobaria pulmonaria* is a lichen associated with old forest habitats in Europe, Asia, Africa and North America. All three species are epiphytes and share the same primary photobiont, the green-alga *Symbiochloris reticulata*, which is mainly vertically transmitted via soredia in *L. pulmonaria*, although photobiont switches do occur occasionally^15, 16^.

Using population genetic approaches on a large dataset comprising microsatellite repeats, we compare the genetic patterns across species. Second, we explore the genetic relationships among geographic regions in each species. Finally, we compare our results with the phylogeographic patterns of phorophyte species of the *Lobaria* lichens, i.e. with the woody plant species the lichen is growing on: laurel trees and heather.

## Results

### RealTime PCR

To identify the lichen species, we confirmed our field identifications with quantitative RealTime PCR. We found 16.5% of mismatches between field species identifications and molecular identifications in *L. immixta*, 21.7% in *L. macaronesica*, and 24.6% in *L. pulmonaria.* These results illustrate that a considerable percentage of thalli in each species were morphologically poorly developed so that reliable morphology-based species identifications were not possible, and they caution against uncritically including poorly developed specimens into population genetic studies of lichen-forming fungi.

### Microsatellite repeat data

**Figure 1:**
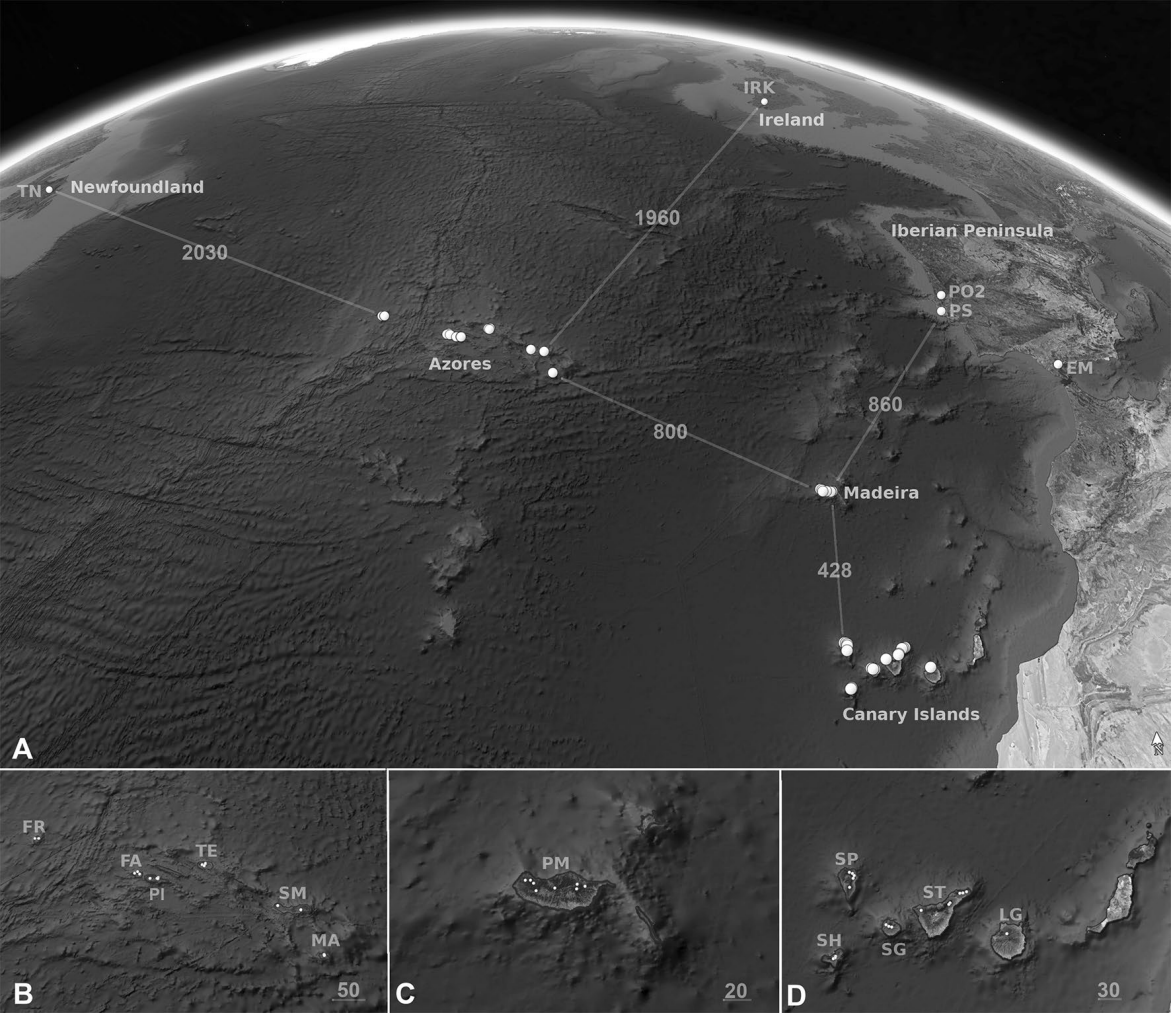
Map of the sampling sites (white dots) for *Lobaria immixta*, *L. macaronesica* and *L. pulmonaria*. (**A**) Overview of the sampling sites. (**B**) Azores. (**C**) Madeira. (**D**) Canary Islands. Numbers represent geographic distances (km). Base map: Google Earth Pro v. 7.3.3.7786, https://earth.google.com/. Population names as in Table 1. The figure was compiled in GIMP v. 2.8, https://www.gimp.org/.

**Figure 2:**
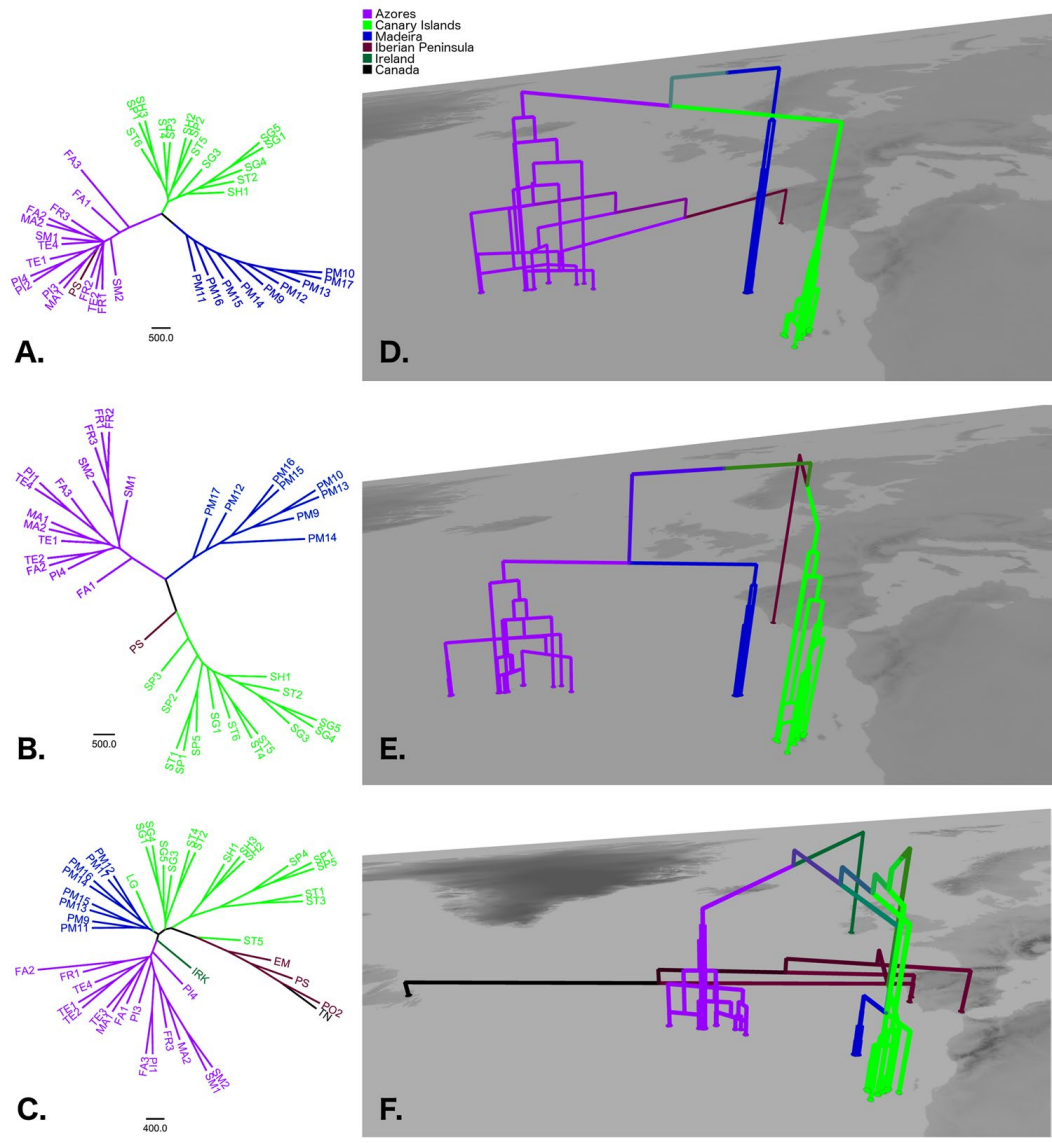
Unrooted population trees and geographic tree models for *Lobaria* sampled from the Macaronesian islands, the Iberian Peninsula and adjacent areas. Trees are neighbor-joining trees based on chord distance between populations. (**A**–**C**) Unrooted neighbor-joining population trees based on 1000 bootstrap replicates, created with PHYLIP v. 3.6.9.5, https://evolution.genetics.washington.edu/phylip.html, and displayed with FigTree v. 1.3.1. (**D**–**F**). Midpoint-rooted geographic population tree models from GenGIS v. 2.5.3, https://beikolab.cs.dal.ca/gengis/Main_Page. (**A**, **D**). *Lobaria immixta*. (**B**, **E**) *L. macaronesica*. (**C**, **F**) *L. pulmonaria*. The figure was compiled in GIMP v. 2.8, https://www.gimp.org/.

**Figure 3:**
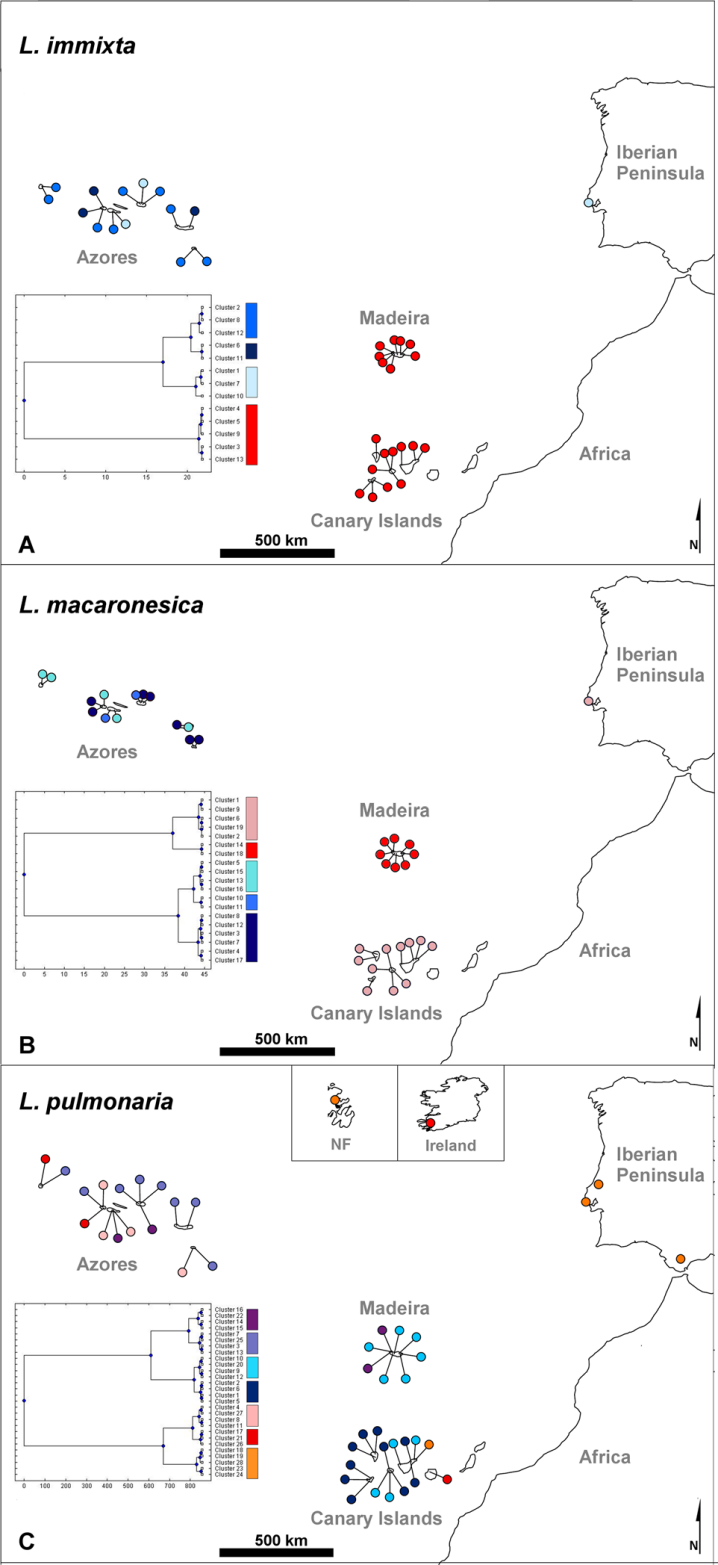
Population structure analysis for *Lobaria* sampled from the Macaronesian islands, the Iberian Peninsula and adjacent areas. Shown are genetic clusters from mixture analysis performed in BAPS v. 5.4, http://www.helsinki.fi/bsg/software/BAPS/; inset: neighbor-joining trees of Nei’s genetic distance among clusters, averaged over loci. (**A**) *Lobaria immixta*. (**B**) *L. macaronesica*. (**C**) *L. pulmonaria*. NF, Newfoundland.

**Figure 4:**
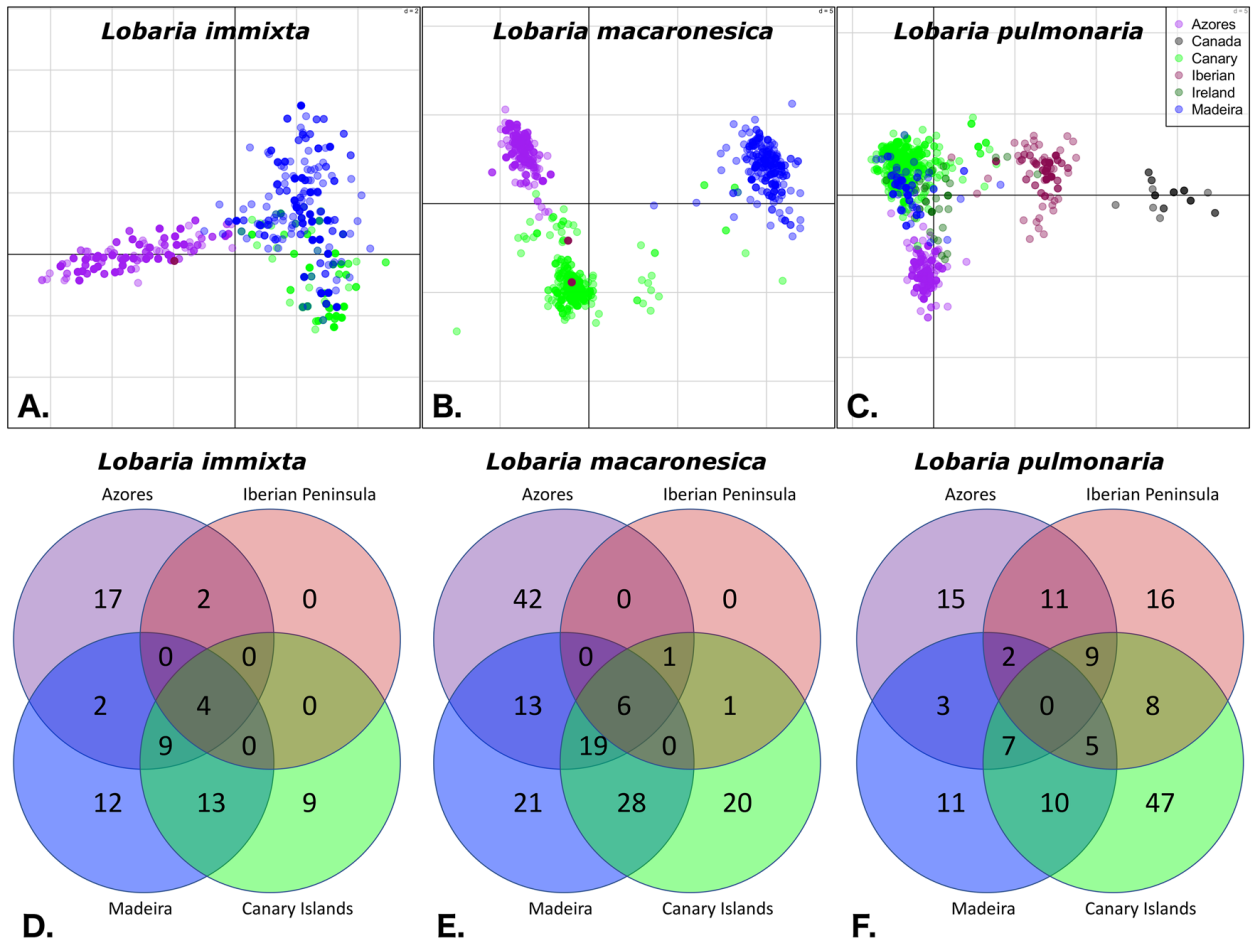
Microsatellite variation between geographic regions. (**A**–**C**) Discriminant Analysis of Principal Components (DAPC) performed in R v. 3.6.1, https://cran.r-project.org/. Sampled individuals are represented by dots, while groups are distinguished by colors. (**D**–**F**) Venn diagrams of the number of private and shared alleles. (**A**, **D**). *Lobaria immixta*. (**B**, **E**). *L. macaronesica*. (**C**, **F**). *L. pulmonaria.* The dimensions of grid cells were (**A**) 2, (**B**) and (**C**) 5, with the units being scores from Principal Components Analysis. The figure was compiled in GIMP v. 2.8, https://www.gimp.org/.

Highly variable fungal microsatellite loci were utilized to quantify the regional population genetic structure in Macaronesia (for sampling sites, see Fig. 1). Diversity statistics are reported in Table 1. For *L. immixta*, population trees indicated that populations on the Canary Islands and Madeira were related to one another, but also isolated, as indicated by them being occupied by separate clades (Fig. 2A,D). In total, 13 genetic clusters were found by Bayesian analysis of population structure in BAPS. The five clusters found on the Canary Islands and Madeira were closely related, but only one cluster was found on Madeira, and it was not shared with the Canary Islands. Sites on the Azores were occupied by eight somewhat more divergent clusters, comprising three major groups. The first group of clusters was widespread throughout the Azores, while the remaining two groups were exclusively found in the central Azores islands. The single site on the Iberian Peninsula grouped with one of the two cluster groups distributed in the central Azores (Figs. 2A,D, 3A). Furthering these patterns, results of Discriminant Analysis of Principal Components (DAPC) revealed a hierarchical steppingstone like model for microsatellite variation of *Lobaria immixta* between islands (Fig. 4A)*.* This highlights gene flow between adjacent islands (Canary Islands & Madeira), with minimal gene flow between these groups and the Azores cluster. The Iberian genotypes grouped with those from the Azores (Fig. 4A). Of all three species investigated, *L. immixta* exhibited the lowest allele counts, e.g. with respect to private alleles (Fig. 4D).

**Table 1:** Diversity statistics and location information for 51 populations situated in six geographic regions (Azores, Canada, Canary Islands, Iberian Peninsula, Ireland, Madeira) for the lichen-forming fungi *Lobaria immixta, L. macaronesica,* and *L. pulmonaria,* based on six fungal microsatellite loci.

Pop	Region	Locality	Longitude	Latitude	*L. immixta*			*L. macaronesica*			*L. pulmonaria*		
					N	MLG	H	N	MLG	H	N	MLG	H
FA1	Azores	Faial	− 28.7722	38.5846	55	16	0.4094	108	19	0.3091	4	4	0.5936
FA2	Azores	Faial	− 28.6600	38.5636	56	13	0.2953	71	13	0.2926	13	7	0.6389
FA3	Azores	Faial	− 28.7070	38.6166	58	6	0.3006	27	10	0.2877	19	6	0.2949
FR1	Azores	Flores	− 31.1642	39.4467	62	29	0.3948	23	16	0.3228	8	6	0.4483
FR2	Azores	Flores	− 31.2513	39.4481	6	4	0.2778	64	26	0.3362	1	–	–
FR3	Azores	Flores	− 31.1543	39.4472	73	22	0.3308	15	10	0.4619	6	5	0.0000
MA1	Azores	Santa Maria	− 25.0888	36.9721	34	12	0.2516	18	6	0.1372	5	3	0.3167
MA2	Azores	Santa Maria	− 25.0904	36.9836	38	12	0.0000	55	12	0.2227	20	11	0.4605
PI1	Azores	Pico	− 28.4279	38.4679	1	–	–	41	6	0.2473	38	7	0.5444
PI2	Azores	Pico	− 28.2765	38.4623	12	1	0.3092	0	–	–	0	–	–
PI3	Azores	Pico	− 28.2575	38.4561	31	3	0.0000	0	–	–	7	3	0.6233
PI4	Azores	Pico	− 28.2534	38.4804	5	1	0.0000	35	7	0.3319	21	4	0.2140
SM1	Azores	Sao Miguel	− 25.7825	37.8513	37	16	0.4074	68	23	0.4000	9	7	0.3736
SM2	Azores	Sao Miguel	− 25.3249	37.7571	37	8	0.3977	43	18	0.4444	43	16	0.4286
TE1	Azores	Terceira	− 27.2037	38.7508	9	3	0.4113	7	2	0.3979	26	5	0.5421
TE2	Azores	Terceira	− 27.2089	38.7338	45	8	0.4065	47	16	0.5132	34	13	0.5327
TE3	Azores	Terceira	− 27.2791	38.7200	1	–	–	1	–	–	23	3	0.4111
TE4	Azores	Terceira	− 27.2352	38.6948	27	6	0.4524	21	10	0.6005	24	3	0.5085
LG	Canary Islands	Gran Canaria	− 15.6193	28.0395	0	–	–	0	–	–	20	6	0.5500
SG1	Canary Islands	La Gomera	− 17.2963	28.1503	34	14	0.0000	30	19	0.5547	13	9	0.5891
SG3	Canary Islands	La Gomera	− 17.2563	28.1310	36	20	0.2813	24	15	0.1758	27	8	0.6017
SG4	Canary Islands	La Gomera	− 17.2151	28.1222	27	16	0.3492	39	33	0.4130	15	14	0.6154
SG5	Canary Islands	La Gomera	− 17.2563	28.1310	26	10	0.3191	64	45	0.3635	13	10	0.2949
SH1	Canary Islands	El Hierro	− 17.9808	27.7610	48	19	0.3966	16	14	0.5447	24	8	0.3889
SH2	Canary Islands	El Hierro	− 17.9865	27.7432	13	6	0.3858	1	–	–	50	29	0.2909
SH3	Canary Islands	El Hierro	− 18.0113	27.7329	48	9	0.4404	0	–	–	61	20	0.4921
SP1	Canary Islands	La Palma	− 17.8351	28.6122	26	7	0.2400	41	16	0.4157	105	73	0.6175
SP2	Canary Islands	La Palma	− 17.7779	28.7602	8	4	0.2586	79	47	0.3881	0	–	–
SP3	Canary Islands	La Palma	− 17.8048	28.7882	13	10	0.1859	47	33	0.3708	1	–	–
SP4	Canary Islands	La Palma	− 17.8502	28.8023	1	–	–	2	1	0.0000	66	56	0.4964
SP5	Canary Islands	La Palma	− 17.7907	28.7260	1	–	–	10	10	0.3507	91	66	0.5895
ST1	Canary Islands	Tenerife	− 16.4078	28.4205	0	–	–	10	4	0.4053	56	40	0.5735
ST2	Canary Islands	Tenerife	− 16.8106	28.3286	87	19	0.3801	21	8	0.3443	27	7	0.5046
ST3	Canary Islands	Tenerife	− 16.4244	28.4020	0	–	–	0	–	–	61	28	0.5111
ST4	Canary Islands	Tenerife	− 16.2712	28.5398	29	7	0.2692	52	21	0.4333	5	2	0.4893
ST5	Canary Islands	Tenerife	− 16.1767	28.5581	41	8	0.1726	66	32	0.4989	6	4	0.0000
ST6	Canary Islands	Tenerife	− 16.2278	28.5433	17	9	0.3932	56	28	0.0000	0	–	–
PM9	Madeira	Madeira	− 16.8847	32.7380	43	32	0.0000	49	37	0.0000	5	5	0.4113
PM10	Madeira	Madeira	− 16.8762	32.7632	18	12	0.0000	10	4	0.3778	0	–	–
PM11	Madeira	Madeira	− 16.8830	32.7384	33	21	0.0000	3	3	0.2926	18	13	0.6174
PM12	Madeira	Madeira	− 17.0159	32.7615	29	16	0.3098	23	14	0.3151	6	4	0.5519
PM13	Madeira	Madeira	− 17.1317	32.7633	59	39	0.2845	46	31	0.3473	27	13	0.5919
PM14	Madeira	Madeira	− 17.1893	32.8307	48	22	0.1413	61	17	0.3678	9	6	0.4221
PM15	Madeira	Madeira	− 17.1579	32.8267	48	33	0.3162	51	30	0.3594	4	3	0.5525
PM16	Madeira	Madeira	− 17.1409	32.8068	56	34	0.2685	32	22	0.0952	11	3	0.4000
PM17	Madeira	Madeira	− 16.8307	32.7449	28	18	0.3054	13	9	0.3141	7	5	0.6000
EM	Ib. Peninsula	Spain	− 5.5900	36.5200	0	–	–	0	–	–	34	19	0.0705
PO2	Ib. Peninsula	Portugal	− 8.8800	39.4900	0	–	–	0	–	–	28	23	0.0550
PS	Ib. Peninsula	Portugal	− 9.3886	38.7894	5	1	0.3523	14	2	0.3071	91	22	0.5700
IRK	Ireland	Killarney	− 9.5300	52.0200	0	–	–	0	–	–	34	27	0.4825
TN	Canada	Terra Nova	− 53.9715	48.5577	0	–	–	0	–	–	25	13	0.3565

The table gives locality information and the sample size (N), number of multilocus genotypes (MLG), and Nei’s unbiased gene diversity (H).

Madeiran and Azorean populations of *L. macaronesica* represented separate groups in the population tree (Fig. 2B,E) and separate groups of genetic clusters from Bayesian analysis of population structure in BAPS (Fig. 3B). In total, 19 genetic clusters were found. Similar to the pattern in *L. immixta*, in *L. macaronesica*, the largest genetic difference resolved with BAPS was between the Azores vs. Canary Islands and Madeira. Other than in *L. immixta*, sites on Madeira and the Canary Islands hosted dissimilar groups of genetic clusters. Sites on the Azores harbored three distinct groups of genetic clusters with Eastern, central, and Western distributions on that archipelago. The site on the Iberian Peninsula grouped with sites on the Canary Islands (Figs. 2B,E, 3B). DAPC clustering reinforced the presence of highly distinct genetic clusters for the Azores, Madeira, and the Canary Islands. The Iberian genotypes grouped within the Canarian cluster of genotypes (Fig. 4B). *Lobaria macaronesica* had about twice as many private alleles on each archipelago as *L. immixta* (Fig. 4E).

In *L. pulmonaria*, the pattern was somewhat more complicated than in the endemic species. A total of 28 genetic clusters were found, reflecting the higher number of sites analyzed in this species and the more extensive (but not rangewide) geographic sampling. The site in Newfoundland, Canada showed some similarity to sites on the Iberian Peninsula and the single site investigated in Ireland showed similarity to the Azores (Fig. 2C,F). One cluster group was shared between the Azores and Canary Islands (Gran Canaria), and another between the Azores and Madeira (Fig. 3C). However, other than in the endemic species, in *L. pulmonaria*, individuals belonging to more or less divergent genetic groups of clusters co-occurred on all archipelagos, indicating a more complicated evolutionary history, with repeated geneflow between archipelagos. Nevertheless, some groups of clusters had the tendency to be more frequent on a single archipelago or were restricted to a single archipelago. Results of DAPC for *Lobaria pulmonaria* exhibited a more complex picture of genetic variation, because more geographic regions could be included in the analysis of this widespread species (Fig. 4C). The Canadian samples represented the only fully distinct cluster. Also Iberian and Azorean genotypes represented distinct clusters. In contrast, the Irish samples formed the major bridge to Macaronesian samples. Similar to the endemic species *L. immixta,* the most overlap in genotypes occurred between the Canarian and Madeiran populations. *Lobaria pulmonaria* had more than twice as many private alleles on the Canary Islands than *L. macaronesica* and five-fold more than *L. immixta* (Fig. 4D–F). On the Azores and Madeira, *L. pulmonaria* showed similar allele counts as *L. immixta* (Fig. 4D,F)*.* Only *L. pulmonaria* had private alleles on the Iberian Peninsula, which could partly be an effect of the larger sample size (Fig. 4F).

There was high genetic differentiation among archipelagos for all species, as indicated by highly significant pairwise F_ST_ values between geographic regions (*p* < 0.001, Table 2). Additionally, there was also considerable genetic differentiation among populations within geographic regions. For all species, most pairwise F_ST_ values between populations were significant (*L. immixta*, Azores: 120 of 120, Canary Islands: 90 of 91, Madeira: 32 of 36 significant; *L. macaronesica*, Azores: 105 of 105, Canary Islands: 91 of 91, Madeira: 27 of 28 significant; *L. pulmonaria*, Azores: 108 of 120, Canary Islands: 118 of 120, Iberian Peninsula: three of three, Madeira: 20 of 28 pairwise F_ST_ values significant at *p* < 0.05), indicating substantial population subdivision. The amount of genetic differentiation between populations differed between species, with *L. immixta* showing lowest and *L. pulmonaria* showing the highest differentiation among Madeiran populations.

**Table 2:** Pairwise F_ST_ values for each geographic region for three lichen fungi *Lobaria immixta*, *L. macaronesica*, and *L. pulmonaria* collected from sites in Macaronesia and in adjacent areas.

*Lobaria immixta*						
	Azores	Canary Islands	Madeira	Ib. Peninsula		
Azores	0.291					
Canary Islands	0.381	0.130				
Madeira	0.283	0.127	0.039			
Ib. Peninsula	0.361	0.575	0.478	–		

*Lobaria macaronesica*						
	Azores	Canary Islands	Madeira	Ib. Peninsula		
Azores	0.244					
Canary Islands	0.340	0.114				
Madeira	0.321	0.365	0.111			
Ib. Peninsula	0.303	0.213	0.367	–		

*Lobaria pulmonaria*						
	Azores	Canada	Canary Islands	Ib. Peninsula	Ireland	Madeira
Azores	0.372					
Canada	0.417	–				
Canary Islands	0.137	0.406	0.202			
Ib. Peninsula	0.345	0.201	0.336	0.193		
Ireland	0.170	0.401	0.140	0.277	–	
Madeira	0.134	0.439	0.063	0.364	0.186	0.275

All values for differentiation between archipelagos were statistically significant (*p* < 0.001). Values on the diagonal, i.e. within geographic regions, represent average pairwise F_ST_ values between populations. These were calculated for regions including more than two populations.

**Figure 5:**
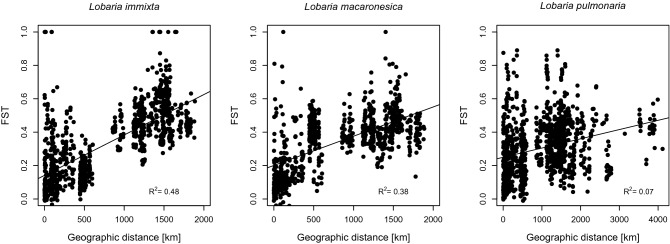
Analysis of isolation by distance, showing the relationship between F_ST_ and geographic distance in *Lobaria immixta*, *L. macaronesica* and *L. pulmonaria* from Macaronesia. All species exhibited a significant relationship. The figure was created in R 3.6.1, https://cran.r-project.org/.

All species showed significant isolation by distance (Fig. 5), as indicated by highly significant relationships between pairwise F_ST_ values and geographic distance in linear models. The relationships were strongest and explained the most variance in *Lobaria immixta* (R^2^= 0.48), followed by *L. macaronesica* (R^2^ = 0.38). *Lobaria pulmonaria* had a statistically significant, but somewhat weaker signal of isolation by distance, where the geographic distance explained only 7% of the variance in pairwise F_ST_ data.

## Discussion

### Comparison of genetic patterns across species

A unifying motive in the microsatellite repeat data of *Lobaria pulmonaria* and its two endemic relatives, *L. immixta* and *L. macaronesica*, was that all three species showed high divergence between archipelagos and significant isolation by distance. This pattern was expected considering the large geographic distance between archipelagos and the lack of stepping-stone habitats characteristic of oceanic island biota. Overall, the population models in DAPC provided evidence for a hierarchical island model. This is consistent with the fact that the studied archipelagos were far apart, supporting divergence, but some islands in closer proximity had the opportunity for occasional gene flow. Previous studies comparing Swiss and Canadian populations of *L. pulmonaria* have found substantial differentiation, as expected in populations situated on different continents^17^. So, not surprisingly, the occurrence and frequency of gene flow appears to decrease with geographic distance in *L. pulmonaria*, which is also evident in our data on isolation by distance. Significant differentiation over large geographic scales has been shown in other lichen fungi as well^18–24^.

Taken together, our data indicate that *L. pulmonaria* has a complex population history in Macaronesia, the coexistence of divergent groups of genetic clusters on the same archipelago suggesting recurrent immigrations from sites on the European continent, but less from the North American continent. By comparison, the population histories of the two endemic species were more straightforward. Homogeneity of groups of genetic clusters within archipelagos rejects the hypothesis of repeated recent migration among archipelagos in the endemics. For both endemic species, each archipelago contained numerous private alleles. Thus, the populations on archipelagos evolved by genetic drift and/or by accumulation of new mutations, causing divergence.

Recent studies of lichen fungi and bryophytes emphasize the importance of Macaronesia for the colonization of sites in continental Europe. First, a study of lichen fungi in the genus *Nephroma* inferred the colonization of mainland sites from sites on the Macaronesian islands^4^. Second, an investigation of a liverwort showed that (1) sites in Macaronesia hosted a hidden diversity in the liverwort comparable to the radiation in higher plants at the genus level, and (2) low-diversity sites in western Europe were recolonized from Macaronesian refugial populations^25^. This diversity pattern was not evident in *L. pulmonaria* (the only species with more occurrences outside of than within Macaronesia)—sites on the mainland were highly diverse - see also^26^ - and were differentiated from island sites. If mainland sites received *L. pulmonaria* from the islands, this migration must have occurred a long time ago because the observed substantial differentiation of the large island and mainland populations would require that many generations have passed.

In contrast, for the lichen *Parmelina carporrhizans*, unidirectional gene flow to the Macaronesian Islands was inferred^27^. Our data for *Lobaria pulmonaria* show a contrasting pattern with repeated gene flow between mainland and Macaronesian Islands and an overall higher differentiation among populations.

A previous study dated the origin of *L. pulmonaria* to 6.9–11.9 Myr BP when it diverged from its Asian relative *Lobaria tuberculata*, and the divergence of the two Macaronesian endemic species from *L. pulmonaria* to 5.5–9.9 Myr BP^28^. Thus, the endemic species are likely to represent neo-endemics that may have evolved on the Macaronesian Islands after diverging from the older and widespread species, *L. pulmonaria*. This is similar to what has been found for Macaronesian *Nephroma* species^4^.

### *Relationship between island and mainland sites in* Lobaria

In all species, we found substantial differentiation among archipelagos, indicating long-term isolation. This was also true for the widespread species, *L.pulmonaria*: most island populations were genetically distinct from populations on the mainland. This reflects a history of low migration to the islands and genetic drift and diversification of island populations, which seem to have evolved from the continental populations and accumulated private alleles, which might have resulted from mutation or from genetic drift leading to loss of variation. Populations of *L. pulmonaria* from the Canary Islands were related to the Iberian Peninsula and those from the Azores to the Irish population. Divergence between geographic regions was also confirmed by our analysis of private and shared alleles. All regions contained a comparatively high number of private alleles in *L. pulmonaria*, with few alleles shared between regions. Hence, gene flow between archipelagos or between continental populations and archipelagos is rather low.

Previous studies of *L. pulmonaria* have found that differentiation between regional populations is mainly dependent on the geographic scale of sampling^29, 30^. On a small spatial scale, individuals of this species showed spatial autocorrelation in genotypes and alleles due to local deposition of diaspores^31–33^, but no or little differentiation was found among forested areas within a pasture woodland^29^ or even among sites tens of km apart within a region^17^. However, significant differentiation among geographic regions has been found when different refugial regions were included in the analysis^26, 34^, or when populations growing in different habitats such as floodplain forests and mountain ridges were compared within one region^35^.

The lack of genetic differentiation between subsets of a landscape or a forested region in *L. pulmonaria* could be due to the overarching role of stepping-stone habitats leading to effective gene flow. In volcanic archipelagos situated off the continental shelf and surrounded by ocean, the islands themselves represent the few stepping stones, but there are no additional populations in between. Thus, we expected a substantial amount of genetic differentiation between sites on different archipelagos, which was indeed found.

However, genetic distance was not simply a function of geographic distance for some of the studied populations of *L.pulmonaria*. Interestingly, sites on the Iberian Peninsula unexpectedly grouped with the site investigated on Newfoundland (but not with the spatially more proximate site in Ireland). Moreover, the site in Ireland was more similar to Azorean populations, rather than to the geographically closer sites on the Iberian Peninsula.

A particularly interesting facet of our data set was the relationship between island and mainland sites in the endemic species. Both endemic species are likely to have evolved on the Macaronesian islands, given their divergence time^28^. In *L. macaronesica*, the single known small population of this species on the Iberian Peninsula is genetically distinct from the Macaronesian island populations and could be the remains of what might have been a much larger population during phases of past, moister climate.

Given its diversity pattern in the microsatellite data, the single site where *L. immixta* is known from the Iberian Peninsula grouped within the Azorean populations (Fig. 2A,D). Therefore, it seems likely that this species has been introduced to the Iberian Peninsula by Man with plant material from the Azores in recent time. An additional consideration that makes this scenario even more likely is that all individuals of this species were found in a Royal palace garden, a site that certainly has received massive amounts of plants (e.g. exotic trees) from various regions in the world, including Macaronesia. Hence, the Iberian populations of the two endemic species of *Lobaria* appear to have different histories.

### Similarity of geographic patterns with Macaronesian phorophytes

For an oceanic archipelago, the Azores host relatively few endemic plant taxa, a pattern which has been ascribed to a less variable paleoclimate because climatic variability can drive radiations^36–38^. The endemic plant species of the Azores are widespread across the archipelago and exhibit little divergence between populations, relative to those of the Canary Islands^36^. In opposition to this pattern, we found substantially higher levels of divergence between Azorean than between Canarian populations in our data, a pattern consistent across all three species. This pattern could be explained by the greater distances among islands on the Azores as compared to the Canary Islands, leading to less geneflow among islands. In the variable paleoclimate of the Canary Islands with its frequent shifts in humidity, the *Lobaria* lichens would have become locally extinct during arid periods, and populations may have gone through repeated genetic bottlenecks, leading to lower genetic variability as observed in our data. In a study of eight plant taxa, substantial genetic diversification was found on the Azores, emphasizing that in some endemic Azorean species, diversification may exist^39^, a pattern consistent with our data on *Lobaria* lichen fungi. A study on *Erica* (phanerogams) had a similar result^40^.

Epiphytic lichens such as the investigated species of *Lobaria* are dependent on a woody plant community. Two phorophyte taxa of the lichens have also been investigated in a phylogeographic context in Macaronesia. In the heather *Erica scoparia*, a western European and Macaronesian shrub which is a frequent phorophyte of *Lobaria* sp. on the Macaronesian Islands, Azorean populations were far more diverse than those in western continental Europe^40^. The authors explained this pattern by recurrent migration to the Azores and extinctions on the mainland. In *L. pulmonaria,* populations on the Azores were diverse and consisted of divergent genetic clusters, but in contrast with the pattern of *E. scoparia*, populations located on the Iberian Peninsula, Newfoundland and Ireland showed high diversity as well. A study targeting the phylogeography of *L. pulmonaria* on the European continent using microsatellite repeat data on thousands of samples showed no tendency for continental populations to have low allelic richness^26^. This could be the case if this lichen spread efficiently into sites affected by Pleistocene glaciations.

The second phorophyte of our study species investigated in a phylogeographic context was laurel tree (*Laurus nobilis* L.*, Laurus azorica* (Seub.) Franco). *Laurus azorica* is a common phorophyte of *Lobaria* lichens in Macaronesia, whereas the drought resistant *Laurus nobilis* on the mainland is only rarely hosting the species. In the *Laurus* species complex, chloroplast haplotypes of Macaronesian *L. azorica* were closely related to those of *L. nobilis* from the western Mediterranean and from Northwestern Africa. The ‘Macaronesian’ haplotype occupied northwestern Africa, the Canary Islands and Madeira. A second, closely related haplotype occupied the Azores, and two others the western Mediterranean. The authors inferred the existence of multiple refugia in *Laurus*, and strong range dynamics in the western Mediterranean part of the range^41^. Climatic reconstructions showed that large, suitable areas for *Laurus* existed in the Pliocene, but during the last glacial maximum, these were reduced to the Mediterranean Basin and the Macaronesian islands^42^. These areas could also have enabled the survival of *Lobaria* spp., given that the climate was moist enough. With some exceptions (e.g. Annaba, Algeria), current sites where *Laurus* is found in northern Africa do not harbor *Lobaria* spp. as the climate is too dry, indicating that the ecological niches of the lichens and their phorophyte do only partially overlap. The large differentiation between Azorean and Canarian/Madeiran populations in all of our study species appears to somewhat resemble the phylogeographic pattern of *Laurus*. We would not have expected a close similarity of genetic patterns due to the differences in life history including dispersal syndrome in trees vs. lichens and as we used different loci.

This study further elucidates the complex evolutionary history of *Lobaria*, revealing the role of geography on the extent of genetic divergence in widespread *Lobaria pulmonaria* and the Macaronesian endemics, and provides one of the few studies dedicated to understanding lichen fungal genetics in an island setting. In summary, the occurrence of highly divergent individuals of *L. pulmonaria* in Macaronesia point towards a complex history with multiple migrations between islands and the mainland. In contrast, the Macaronesian endemics *L. immixta* and *L. macaronesica* with a reduced geographic range on the mainland which likely originated in Macaronesia, exhibit high divergence between archipelagos. While the single population of *L. macaronesica* on the Iberian Peninsula may represent an old population that has diverged from the Macaronesian island populations, *L. immixta* appeared to have recently been introduced by Man to the Iberian Peninsula. Although this report improves our understanding of *Lobaria’*s evolutionary history, these findings could be furthered by more extensive sampling of *Lobaria* populations both in Macaronesia and elsewhere, and the subsequent addition of more loci.

## Materials and methods

### Study area

Our study area included three volcanic archipelagos located in the North Atlantic—the five westernmost Canary Islands, Madeira, the Azores, and in adjacent areas. The vegetation of the study sites is characterized by moist forests: laurel forests, *Pinus canariensis* forests, and high altitudinal shrub vegetation. The study sites were located in high elevational areas that were often covered with fog or were located within the Passat cloud zone. On the Azores islands, we often found *Lobaria* spp. in the near-natural forests covering the margins of calderas and in high elevation forests.

The Canary Islands are situated c. 100 km NW of the coast of southern Morocco, an area which is currently too dry for *Lobaria* spp. In contrast, the Rif mountains of Morocco are known to harbor *L. pulmonaria*^43, 44^, but they are much further away from the Canary Islands (ca. 1000 km, Fig. 1). Both the Azores and Madeira are far away from the Iberian Peninsula (3600 km and 1400 km). Due to the large distances, gene movement between archipelagos and mainland is expected to be very low.

The age of the three archipelagos is well documented. Island age decreases from East to West on the Canary Island archipelago: Fuerteventura, 20.6 Ma; Lanzarote, 15.5 Ma; Gran Canaria, 14.5 Ma; La Gomera, 12.0 Ma; Tenerife, ~ 7.5 Ma; La Palma, 2.0 Ma; El Hierro, 1.12 Ma^7^. Tenerife is a special case because it consists of three palaeoislands (Adeje, 12 Ma; Teno, 6 Ma; Anaga, 4 Ma), which were connected by a rather recent (1 Ma) event^8^. The Selvagem archipelago (27 Ma) is situated between the Canary Islands and Madeira and is characterized by a dry climate and of low elevation (≤ 153 m) vegetation lacking forest^45^. Due to lack of suitable forest habitat, Selvagem could not have served as a steppingstone between the other archipelagos for our study species in recent time. Madeira consists of two main islands: Madeira (< 5.6 Ma) and the older Porto Santo (14 Ma)^45–47^.

The Azores were formed along spreading midoceanic ridges at the joint of the African, American and European plates^48, 49^. The studied islands are Santa Maria (8.12 Ma), Sao Miguel (4.01 Ma)^50^, Terceira (3.52 Ma), Faial (0.73 Ma), Pico (0.25 Ma)^51^, and Flores (2.16 Ma)^52^. The occurrence of a land bridge connecting Pico and Faial at the maximum of the last glaciation (18,000 BP) has been reported^53, 54^. It seems unlikely that this land bridge would have led to increased connectivity between the high-altitude forest types characteristically inhabited by *Lobaria* lichens, unless the habitat suitable for *Lobaria* spp. occurred at considerably lower elevations in the past.

### Sampling

From each collecting area, we collected specimens from one (Gran Canaria) to nine (Madeira) populations, depending on island size and availability of populations of *Lobaria* (Fig. 1; Table 1). Prior to field sampling, collecting permits were obtained for all studied regions. All collected material was deposited in the publically available cryoherbarium Christoph Scheidegger at WSL Swiss Federal Research Institute, cryopreserved at − 20 °C for long-term storage. Voucher numbers and collection information is provided in the Supplementary Information (Table S1). Within each site, we collected about 60–80 samples from each species, or fewer if the local population size was small. In total, 4144 samples were collected from 51 sites. The investigated material included 1528 specimens from 18 sites on the Azores, 1649 from 19 sites on the Canary Islands, 736 from nine sites on Madeira, 25 from Newfoundland, 34 from Ireland, and 172 samples from three sites on the Iberian Peninsula, including Sintra, a locality close to Lisbon with a humid Mediterranean climate (Table 1). This site was characterized by a vegetation element similar to that of the Macaronesian islands. From the collecting sites in Newfoundland, Ireland, and sites EM and PO2 on the Iberian Peninsula, only *Lobaria pulmonaria* was collected. The endemic species have not been reported from these sites.

Samples were collected along a transect through the population in large populations. If the local population size was small, we deviated from this sampling scheme and sampled lichens from all trees situated within approximately 1 hectare of forest. We attempted to include three specimens per species from each sampled tree. Our species identifications in the field were sometimes difficult as many specimens were poorly developed. Hence, we had to rely on molecular species identifications by RealTime PCR^55^.

### Study species

Our study species were *Lobaria pulmonaria*, *L. immixta* and *L. macaronesica*. *Lobaria pulmonaria* is a widespread epiphytic lichen in large parts of the northern hemisphere, with a few occurrences in the southern hemisphere^56^. The other two species are endemic to Macaronesia but are both known from one spot on the mainland in Portugal (Sintra). From this site, *L. immixta* had been reported by Christa and Josef Poelt in 1961^57^. This is the second report of this species from this site. Moreover, *L. macaronesica* was discovered in Sintra in 2007 by C. Scheidegger^13^. For the endemic species, all samples from Sintra were found inside of the Royal Gardens, while *L. pulmonaria* also occurred in natural forests located in the vicinity.

### Molecular analysis

DNA was extracted following the manufacturer’s protocol using the DNeasy 96 plant kit (Qiagen, Hilden, Germany). Each thallus was inspected for the presence of apothecia and parasites, and only thallus parts free of these structures were utilized for DNA extractions. Field identifications of the lichen material was done by SW and CS, lab identifications by SW. As many specimens were not well developed and lacked the diagnostic propagules^13^, we utilized RealTime PCR for species identifications. The RealTime PCR used a small (10 bp), species-specific stretch of the Internal Transcribed Spacer (ITS) region. For protocols, see Werth et al.^55^.

The genotyping of microsatellites followed Werth et al.^58^. Only six out of eight loci worked for all species and these were used for the analyses to ensure comparability among data sets; these markers were fungus-specific according to Widmer et al.^59^. We recently developed additional microsatellite loci for *L. pulmonaria*^60^, but the variability in the present set of six markers was highly suitable for a regional-scale study^58^.

All markers were run in a single multiplex PCR. For two loci, we used primers optimized to work in all species. Each PCR reaction contained 200 nmol LPu09F, 200 nmol LPu09R-PET, 350 nmol of LPu15F, 350 nmol LPu15R-PET, 200 nmol LPu23F-6FAM, 200 nmol LPu23R, 200 nmol LPu24F2-VIC (5′-TGA GGA GTA GAG ATA CAA CGT-3′, this study), 200 nmol LPu24R, 300 nmol LPu25F3-NED (5′-CTA TTC ATT TCT TGT GTT GAG TG -3′, this study), 300 nmol LPu25R 5′-CAT GAA ACG GTT TTG GTT GA-3′ ^26,59^, 200 nmol LPu28F, and 200 nmol LPu28R-VIC. Primer sequences not shown above are given in Walser et al.^61^. Fluorescent labels VIC, PET, NED, and 6FAM were used, supplied by Life Technologies (Rotkreuz, Switzerland) or Sigma-Aldrich (Buchs, Switzerland). Additionally, each reaction contained 2.5 μL Qiagen multiplex PCR mix (Qiagen, Hilden), 0.5 μL diluted DNA (c. 0.5–10 ng) and H_2_O to 5 μL. Cycling conditions were 95 °C for 15 min; 35 cycles of 95 °C for 15 s, 52 °C for 30 s, 72 °C for 1 min; followed by a final elongation step of 60 °C for 30 min. Fragment analyses were run on an automated capillary sequencer (3730xl DNA Analyzer, Life Technologies, Rotkreuz). Alleles were genotyped with an internal size standard (LIZ500) using GeneMapper version 3.7 (Life Technologies, Rotkreuz).

### Data analysis

In order to compare regional genetic structures among endemic and widespread species, we utilized extensive microsatellite datasets of each species to quantify the overall genetic relationships between sites, to infer the grouping of populations, and to analyze the partitioning of genetic variability upon sites and geographic regions.

For each species, 1000 bootstrapped microsatellite allele frequency datasets were generated using the ‘seqboot’ module of PHYLIP version 3.69^62^. The genetic relationships between all studied sites were evaluated for each species using the chord distance *D*_C_^63^, as implemented in the ‘gendist’ module of PHYLIP. Neighbor-joining trees were constructed with ‘neighbor’, and a majority-rule consensus tree was computed using the ‘consense’ module of PHYLIP. The consensus trees were midpoint rooted with FigTree version 1.2.1^64^ and visualized on a map with the geo-spatial information system GenGIS version 2.4.1^65^.

In order to analyze the main grouping in the data of each species and to infer the number of genetic groups (K) in each species, sites were clustered in a mixture analysis using BAPS version 5.4^66, 67^. After an initial run for values of K up to 39 (number of sites), an additional 10 runs were performed for the optimum partition in each species. Admixture analysis (data not shown) was run and none of 1402 individuals (*L. immixta*), three of 1499 (*L. macaronesica*), and nine of 1239 samples (*L. pulmonaria*) were found to be admixed when a Bonferroni correction for multiple testing had been applied to the data. Neighbor-joining trees depicting the relationships between clusters were generated in BAPS using Nei’s genetic distance, averaged over loci^68, 69^. The major groupings were mapped in ArcGIS version 10 (ESRI). To quantify genetic differentiation between geographic regions, population pairwise F_ST_ values were computed in Arlequin version 3.5^70^ for each of the three fungal species.

Discriminant Analysis of Principal Components (DAPC) can provide valuable information about the most likely population model of a set of sampling sites, as different geneflow patterns lead to different genetic clustering^71^. Thus, it is possible to distinguish e.g. island models of gene flow from hierarchical island models or stepping-stone models^72, 73^. DAPC was implemented in the R-package ‘adegenet’ v. 2.1.3^74^ in R v. 3.6.1^75^.

To assess whether there was isolation by distance, pairwise F_ST_ values were calculated in R using the ‘hierfstat’ package v. 0.04-22 and geographic distances between sites were calculated with the package ‘geosphere’ v. 1.5-10 using the ‘distm’ and ‘distGeo’ functions. Significance of isolation by distance was assessed with linear regression models, where F_ST_ was the response variable and geographic distance the predictor.

### Data

The current data set consists of six fungus-specific microsatellite data and all allele frequency are accessible in the Dryad repository^76^.

## Supplementary Information

**Supplementary Table S1**.
