# Supplementary material for: Deep divergence between island populations in lichenized fungi
Source: Sci Rep. 2021 Apr 1;11:7428. doi: 10.1038/s41598-021-86448-z (PMC8016866; doi:10.1038/s41598-021-86448-z)
Supplement: Supplementary file 1 — Supplementary Table S1. [file 41598_2021_86448_MOESM1_ESM.pdf]

Supplementary Information of:

Deep Divergence among Macaronesian Island Populations in Lichenized fungi

Silke Werth, Peter Meidl & Christoph Scheidegger, Scientific Reports

TABLE S1. Overview over herbarium voucher material deposited in the frozen herbarium of Christoph Scheidegger at WSL. The table gives the sample identity as used in our population genetic study, population name, combination of population name and tree number, the herbarium voucher number, longitude and latitude (map datum: WGS84), location of collecting site and geographic area. VoucherIDs are printed in fat where one voucher consists of several lichen thalli.

ID	Pop	PopTree	Species	VoucherID	X	Y	Location	Area
FA1-01f	FA1	FA1-1	*Lobaria immixta*	14247	-28,77222001	38,58463	Faial	Azores
FA1-10a	FA1	FA1-10	*Lobaria immixta*	14286	-28,7720098	38,5848187	Faial	Azores
FA1-10f	FA1	FA1-10	*Lobaria immixta*	14291	-28,7720098	38,5848187	Faial	Azores
FA1-11a	FA1	FA1-11	*Lobaria immixta*	14292	-28,77200679	38,58481739	Faial	Azores
FA1-11b	FA1	FA1-11	*Lobaria immixta*	14293	-28,77200679	38,58481739	Faial	Azores
FA1-11c	FA1	FA1-11	*Lobaria immixta*	14294	-28,77200679	38,58481739	Faial	Azores
FA1-11f	FA1	FA1-11	*Lobaria immixta*	14297	-28,77200679	38,58481739	Faial	Azores
FA1-12a	FA1	FA1-12	*Lobaria immixta*	14301	-28,77200506	38,58481857	Faial	Azores
FA1-12b	FA1	FA1-12	*Lobaria immixta*	14302	-28,77200506	38,58481857	Faial	Azores
FA1-12c	FA1	FA1-12	*Lobaria immixta*	14303	-28,77200506	38,58481857	Faial	Azores
FA1-15b	FA1	FA1-15	*Lobaria immixta*	14313	-28,77196856	38,58477668	Faial	Azores
FA1-16c	FA1	FA1-16	*Lobaria immixta*	14318	-28,77197238	38,58478707	Faial	Azores
FA1-17e	FA1	FA1-17	*Lobaria immixta*	14332	-28,77194347	38,58477238	Faial	Azores
FA1-18a	FA1	FA1-18	*Lobaria immixta*	14333	-28,77182883	38,58476784	Faial	Azores

ID	Pop	PopTree	Species	VoucherID	X	Y	Location	Area
FA1-02a	FA1	FA1-2	*Lobaria immixta*	14250	-28,77216275	38,58462694	Faial	Azores
FA1-02b	FA1	FA1-2	*Lobaria immixta*	14251	-28,77216275	38,58462694	Faial	Azores
FA1-02c	FA1	FA1-2	*Lobaria immixta*	14252	-28,77216275	38,58462694	Faial	Azores
FA1-02d	FA1	FA1-2	*Lobaria immixta*	14253	-28,77216275	38,58462694	Faial	Azores
FA1-02e	FA1	FA1-2	*Lobaria immixta*	14254	-28,77216275	38,58462694	Faial	Azores
FA1-21b	FA1	FA1-21	*Lobaria immixta*	14346	-28,77180056	38,5847705	Faial	Azores
FA1-21c	FA1	FA1-21	*Lobaria immixta*	14347	-28,77180056	38,5847705	Faial	Azores
FA1-22a	FA1	FA1-22	*Lobaria immixta*	14348	-28,77179502	38,58476935	Faial	Azores
FA1-23b	FA1	FA1-23	*Lobaria immixta*	14354	-28,77180583	38,58475883	Faial	Azores
FA1-26b	FA1	FA1-26	*Lobaria immixta*	14363	-28,77179746	38,58473485	Faial	Azores
FA1-27b	FA1	FA1-27	*Lobaria immixta*	14367	-28,77183351	38,58468358	Faial	Azores
FA1-28a	FA1	FA1-28	*Lobaria immixta*	14370	-28,77185428	38,58465144	Faial	Azores
FA1-28b	FA1	FA1-28	*Lobaria immixta*	14371	-28,77185428	38,58465144	Faial	Azores
FA1-28c	FA1	FA1-28	*Lobaria immixta*	14372	-28,77185428	38,58465144	Faial	Azores
FA1-03b	FA1	FA1-3	*Lobaria immixta*	14256	-28,77215573	38,58463001	Faial	Azores
FA1-03c	FA1	FA1-3	*Lobaria immixta*	14257	-28,77215573	38,58463001	Faial	Azores
FA1-31c	FA1	FA1-31	*Lobaria immixta*	14383	-28,77159357	38,58465672	Faial	Azores
FA1-04a	FA1	FA1-4	*Lobaria immixta*	14258	-28,77215021	38,58463126	Faial	Azores
FA1-04b	FA1	FA1-4	*Lobaria immixta*	14259	-28,77215021	38,58463126	Faial	Azores
FA1-40a	FA1	FA1-40	*Lobaria immixta*	14410	-28,77119246	38,58444418	Faial	Azores
FA1-40b	FA1	FA1-40	*Lobaria immixta*	14411	-28,77119246	38,58444418	Faial	Azores
FA1-41a	FA1	FA1-41	*Lobaria immixta*	14414	-28,77140081	38,58455886	Faial	Azores
FA1-05a	FA1	FA1-5	*Lobaria immixta*	14261	-28,7721542	38,5847934	Faial	Azores
FA1-05c	FA1	FA1-5	*Lobaria immixta*	14263	-28,7721542	38,5847934	Faial	Azores
FA1-05d	FA1	FA1-5	*Lobaria immixta*	14264	-28,7721542	38,5847934	Faial	Azores
FA1-06a	FA1	FA1-6	*Lobaria immixta*	14266	-28,77211981	38,58479203	Faial	Azores
FA1-06b	FA1	FA1-6	*Lobaria immixta*	14267	-28,77211981	38,58479203	Faial	Azores

ID	Pop	PopTree	Species	VoucherID	X	Y	Location	Area
FA1-06c	FA1	FA1-6	*Lobaria immixta*	14268	-28,77211981	38,58479203	Faial	Azores
FA1-06d	FA1	FA1-6	*Lobaria immixta*	14269	-28,77211981	38,58479203	Faial	Azores
FA1-07a	FA1	FA1-7	*Lobaria immixta*	14271	-28,77206467	38,58480453	Faial	Azores
FA1-07b	FA1	FA1-7	*Lobaria immixta*	14272	-28,77206467	38,58480453	Faial	Azores
FA1-07c	FA1	FA1-7	*Lobaria immixta*	14273	-28,77206467	38,58480453	Faial	Azores
FA1-08a	FA1	FA1-8	*Lobaria immixta*	14274	-28,77205434	38,58480059	Faial	Azores
FA1-08b	FA1	FA1-8	*Lobaria immixta*	14275	-28,77205434	38,58480059	Faial	Azores
FA1-09a	FA1	FA1-9	*Lobaria immixta*	14277	-28,77205894	38,58479542	Faial	Azores
FA1-09b	FA1	FA1-9	*Lobaria immixta*	14278	-28,77205894	38,58479542	Faial	Azores
FA1-09c	FA1	FA1-9	*Lobaria immixta*	14279	-28,77205894	38,58479542	Faial	Azores
FA1-09d	FA1	FA1-9	*Lobaria immixta*	14280	-28,77205894	38,58479542	Faial	Azores
FA1-09g	FA1	FA1-9	*Lobaria immixta*	14283	-28,77205894	38,58479542	Faial	Azores
FA1-09h	FA1	FA1-9	*Lobaria immixta*	14284	-28,77205894	38,58479542	Faial	Azores
FA1-09i	FA1	FA1-9	*Lobaria immixta*	14285	-28,77205894	38,58479542	Faial	Azores
FA2-01a	FA2	FA2-1	*Lobaria immixta*	14426	-28,65995	38,56355	Faial	Azores
FA2-01b	FA2	FA2-1	*Lobaria immixta*	**14427**	-28,65995	38,56355	Faial	Azores
FA2-01b1	FA2	FA2-1	*Lobaria immixta*	**14427**	-28,65995	38,56355	Faial	Azores
FA2-01c	FA2	FA2-1	*Lobaria immixta*	**14428**	-28,65995	38,56355	Faial	Azores
FA2-01c1	FA2	FA2-1	*Lobaria immixta*	**14428**	-28,65995	38,56355	Faial	Azores
FA2-01d	FA2	FA2-1	*Lobaria immixta*	**14429**	-28,65995	38,56355	Faial	Azores
FA2-01d1	FA2	FA2-1	*Lobaria immixta*	**14429**	-28,65995	38,56355	Faial	Azores
FA2-10a	FA2	FA2-10	*Lobaria immixta*	14508	-28,66021695	38,56357897	Faial	Azores
FA2-10b	FA2	FA2-10	*Lobaria immixta*	14509	-28,66021695	38,56357897	Faial	Azores
FA2-10c	FA2	FA2-10	*Lobaria immixta*	14510	-28,66021695	38,56357897	Faial	Azores
FA2-10d	FA2	FA2-10	*Lobaria immixta*	14511	-28,66021695	38,56357897	Faial	Azores
FA2-10e	FA2	FA2-10	*Lobaria immixta*	14512	-28,66021695	38,56357897	Faial	Azores
FA2-11e	FA2	FA2-11	*Lobaria immixta*	14519	-28,66025081	38,56358416	Faial	Azores

ID	Pop	PopTree	Species	VoucherID	X	Y	Location	Area
FA2-11f	FA2	FA2-11	*Lobaria immixta*	14520	-28,66025081	38,56358416	Faial	Azores
FA2-11g	FA2	FA2-11	*Lobaria immixta*	14521	-28,66025081	38,56358416	Faial	Azores
FA2-12a	FA2	FA2-12	*Lobaria immixta*	14523	-28,66042269	38,56357695	Faial	Azores
FA2-12b	FA2	FA2-12	*Lobaria immixta*	14524	-28,66042269	38,56357695	Faial	Azores
FA2-12c	FA2	FA2-12	*Lobaria immixta*	14525	-28,66042269	38,56357695	Faial	Azores
FA2-12d	FA2	FA2-12	*Lobaria immixta*	14526	-28,66042269	38,56357695	Faial	Azores
FA2-12e	FA2	FA2-12	*Lobaria immixta*	14527	-28,66042269	38,56357695	Faial	Azores
FA2-12f	FA2	FA2-12	*Lobaria immixta*	14528	-28,66042269	38,56357695	Faial	Azores
FA2-12h	FA2	FA2-12	*Lobaria immixta*	14530	-28,66045833	38,5635996	Faial	Azores
FA2-18b	FA2	FA2-18	*Lobaria immixta*	14554	-28,66011476	38,56357708	Faial	Azores
FA2-02a	FA2	FA2-2	*Lobaria immixta*	**14432**	-28,65997279	38,56355215	Faial	Azores
FA2-02a1	FA2	FA2-2	*Lobaria immixta*	**14432**	-28,65997279	38,56355215	Faial	Azores
FA2-02b	FA2	FA2-2	*Lobaria immixta*	**14433**	-28,65997279	38,56355215	Faial	Azores
FA2-02b1	FA2	FA2-2	*Lobaria immixta*	**14433**	-28,65997279	38,56355215	Faial	Azores
FA2-02c	FA2	FA2-2	*Lobaria immixta*	14434	-28,65997279	38,56355215	Faial	Azores
FA2-02d	FA2	FA2-2	*Lobaria immixta*	**14435**	-28,65997279	38,56355215	Faial	Azores
FA2-02d1	FA2	FA2-2	*Lobaria immixta*	**14435**	-28,65997279	38,56355215	Faial	Azores
FA2-22c	FA2	FA2-22	*Lobaria immixta*	14562	-28,65969564	38,56362808	Faial	Azores
FA2-04e	FA2	FA2-4	*Lobaria immixta*	14453	-28,66000579	38,56355493	Faial	Azores
FA2-04f	FA2	FA2-4	*Lobaria immixta*	14454	-28,66000579	38,56355493	Faial	Azores
FA2-04g	FA2	FA2-4	*Lobaria immixta*	14455	-28,66000579	38,56355493	Faial	Azores
FA2-04h	FA2	FA2-4	*Lobaria immixta*	14456	-28,66000579	38,56355493	Faial	Azores
FA2-04i	FA2	FA2-4	*Lobaria immixta*	14457	-28,66000579	38,56355493	Faial	Azores
FA2-05a	FA2	FA2-5	*Lobaria immixta*	14462	-28,66003446	38,5635538	Faial	Azores
FA2-05c	FA2	FA2-5	*Lobaria immixta*	14464	-28,66003446	38,5635538	Faial	Azores
FA2-06a	FA2	FA2-6	*Lobaria immixta*	14468	-28,66004375	38,56354844	Faial	Azores
FA2-06b	FA2	FA2-6	*Lobaria immixta*	14469	-28,66004375	38,56354844	Faial	Azores

ID	Pop	PopTree	Species	VoucherID	X	Y	Location	Area
FA2-06c	FA2	FA2-6	*Lobaria immixta*	14470	-28,66004375	38,56354844	Faial	Azores
FA2-06d	FA2	FA2-6	*Lobaria immixta*	14471	-28,66004375	38,56354844	Faial	Azores
FA2-07e	FA2	FA2-7	*Lobaria immixta*	14483	-28,660155	38,5635702	Faial	Azores
FA2-07f	FA2	FA2-7	*Lobaria immixta*	14484	-28,660155	38,5635702	Faial	Azores
FA2-07g	FA2	FA2-7	*Lobaria immixta*	14485	-28,660155	38,5635702	Faial	Azores
FA2-07h	FA2	FA2-7	*Lobaria immixta*	14486	-28,660155	38,5635702	Faial	Azores
FA2-08f	FA2	FA2-8	*Lobaria immixta*	14492	-28,66016076	38,56357093	Faial	Azores
FA2-08g	FA2	FA2-8	*Lobaria immixta*	14493	-28,66016076	38,56357093	Faial	Azores
FA2-08h	FA2	FA2-8	*Lobaria immixta*	14494	-28,66016076	38,56357093	Faial	Azores
FA2-09b	FA2	FA2-9	*Lobaria immixta*	14496	-28,66019407	38,5635774	Faial	Azores
FA2-09c	FA2	FA2-9	*Lobaria immixta*	14497	-28,66019407	38,5635774	Faial	Azores
FA2-09d	FA2	FA2-9	*Lobaria immixta*	14498	-28,66019407	38,5635774	Faial	Azores
FA2-09e	FA2	FA2-9	*Lobaria immixta*	14499	-28,66019407	38,5635774	Faial	Azores
FA2-09g	FA2	FA2-9	*Lobaria immixta*	14501	-28,66019407	38,5635774	Faial	Azores
FA2-09j	FA2	FA2-9	*Lobaria immixta*	14504	-28,66019407	38,5635774	Faial	Azores
FA2-09k	FA2	FA2-9	*Lobaria immixta*	14505	-28,66019407	38,5635774	Faial	Azores
FA3-10a	FA3	FA3-10	*Lobaria immixta*	14615	-28,69755049	38,61241365	Faial	Azores
FA3-10b	FA3	FA3-10	*Lobaria immixta*	14616	-28,69755049	38,61241365	Faial	Azores
FA3-10c	FA3	FA3-10	*Lobaria immixta*	14617	-28,69755049	38,61241365	Faial	Azores
FA3-11a	FA3	FA3-11	*Lobaria immixta*	14618	-28,69753396	38,61240115	Faial	Azores
FA3-11b	FA3	FA3-11	*Lobaria immixta*	14619	-28,69753396	38,61240115	Faial	Azores
FA3-11c	FA3	FA3-11	*Lobaria immixta*	14620	-28,69753396	38,61240115	Faial	Azores
FA3-12a	FA3	FA3-12	*Lobaria immixta*	14621	-28,6974268	38,61243352	Faial	Azores
FA3-12b	FA3	FA3-12	*Lobaria immixta*	14622	-28,6974268	38,61243352	Faial	Azores
FA3-12c	FA3	FA3-12	*Lobaria immixta*	14623	-28,6974268	38,61243352	Faial	Azores
FA3-13a	FA3	FA3-13	*Lobaria immixta*	14624	-28,69741026	38,61242102	Faial	Azores
FA3-13b	FA3	FA3-13	*Lobaria immixta*	14625	-28,69741026	38,61242102	Faial	Azores

ID	Pop	PopTree	Species	VoucherID	X	Y	Location	Area
FA3-13c	FA3	FA3-13	*Lobaria immixta*	14626	-28,69741026	38,61242102	Faial	Azores
FA3-13d	FA3	FA3-13	*Lobaria immixta*	14627	-28,69741026	38,61242102	Faial	Azores
FA3-15a	FA3	FA3-15	*Lobaria immixta*	14633	-28,69754752	38,61231935	Faial	Azores
FA3-15b	FA3	FA3-15	*Lobaria immixta*	14634	-28,69754752	38,61231935	Faial	Azores
FA3-15c	FA3	FA3-15	*Lobaria immixta*	14635	-28,69754752	38,61231935	Faial	Azores
FA3-15d	FA3	FA3-15	*Lobaria immixta*	14636	-28,69754752	38,61231935	Faial	Azores
FA3-15e	FA3	FA3-15	*Lobaria immixta*	14637	-28,69754752	38,61231935	Faial	Azores
FA3-16a	FA3	FA3-16	*Lobaria immixta*	14639	-28,69749734	38,61234123	Faial	Azores
FA3-16b	FA3	FA3-16	*Lobaria immixta*	14640	-28,69749734	38,61234123	Faial	Azores
FA3-16c	FA3	FA3-16	*Lobaria immixta*	14641	-28,69749734	38,61234123	Faial	Azores
FA3-17a	FA3	FA3-17	*Lobaria immixta*	14642	-28,69747746	38,61235026	Faial	Azores
FA3-17b	FA3	FA3-17	*Lobaria immixta*	14643	-28,69747746	38,61235026	Faial	Azores
FA3-17c	FA3	FA3-17	*Lobaria immixta*	14644	-28,69747746	38,61235026	Faial	Azores
FA3-18a	FA3	FA3-18	*Lobaria immixta*	14645	-28,69746628	38,61237582	Faial	Azores
FA3-18b	FA3	FA3-18	*Lobaria immixta*	14646	-28,69746628	38,61237582	Faial	Azores
FA3-18c	FA3	FA3-18	*Lobaria immixta*	14647	-28,69746628	38,61237582	Faial	Azores
FA3-18d	FA3	FA3-18	*Lobaria immixta*	14648	-28,69746628	38,61237582	Faial	Azores
FA3-19a	FA3	FA3-19	*Lobaria immixta*	14649	-28,68943	38,60944	Faial	Azores
FA3-19b	FA3	FA3-19	*Lobaria immixta*	14650	-28,68943	38,60944	Faial	Azores
FA3-19c	FA3	FA3-19	*Lobaria immixta*	14651	-28,68943	38,60944	Faial	Azores
FA3-19d	FA3	FA3-19	*Lobaria immixta*	14652	-28,68943	38,60944	Faial	Azores
FA3-19e	FA3	FA3-19	*Lobaria immixta*	14653	-28,68943	38,60944	Faial	Azores
FA3-20a	FA3	FA3-20	*Lobaria immixta*	14654	-28,68914	38,60935	Faial	Azores
FA3-20b	FA3	FA3-20	*Lobaria immixta*	14655	-28,68914	38,60935	Faial	Azores
FA3-20c	FA3	FA3-20	*Lobaria immixta*	14656	-28,68914	38,60935	Faial	Azores
FA3-21a	FA3	FA3-21	*Lobaria immixta*	14657	-28,68914015	38,60935631	Faial	Azores
FA3-21b	FA3	FA3-21	*Lobaria immixta*	14658	-28,68914015	38,60935631	Faial	Azores

ID	Pop	PopTree	Species	VoucherID	X	Y	Location	Area
FA3-21c	FA3	FA3-21	*Lobaria immixta*	14659	-28,68914015	38,60935631	Faial	Azores
FA3-22a	FA3	FA3-22	*Lobaria immixta*	14660	-28,68913441	38,60935639	Faial	Azores
FA3-22b	FA3	FA3-22	*Lobaria immixta*	14661	-28,68913441	38,60935639	Faial	Azores
FA3-22c	FA3	FA3-22	*Lobaria immixta*	14662	-28,68913441	38,60935639	Faial	Azores
FA3-22d	FA3	FA3-22	*Lobaria immixta*	14663	-28,68913441	38,60935639	Faial	Azores
FA3-22e	FA3	FA3-22	*Lobaria immixta*	14664	-28,68913441	38,60935639	Faial	Azores
FA3-23a	FA3	FA3-23	*Lobaria immixta*	14665	-28,68914	38,60943	Faial	Azores
FA3-23b	FA3	FA3-23	*Lobaria immixta*	14666	-28,68914	38,60943	Faial	Azores
FA3-23c	FA3	FA3-23	*Lobaria immixta*	14667	-28,68914	38,60943	Faial	Azores
FA3-04d	FA3	FA3-4	*Lobaria immixta*	14587	-28,70741628	38,61725537	Faial	Azores
FA3-04e	FA3	FA3-4	*Lobaria immixta*	14588	-28,70741628	38,61725537	Faial	Azores
FA3-08e	FA3	FA3-8	*Lobaria immixta*	14606	-28,70025391	38,61255483	Faial	Azores
FA3-08f	FA3	FA3-8	*Lobaria immixta*	14607	-28,70025391	38,61255483	Faial	Azores
FA3-08g	FA3	FA3-8	*Lobaria immixta*	14608	-28,70025391	38,61255483	Faial	Azores
FA3-08h	FA3	FA3-8	*Lobaria immixta*	14609	-28,70025391	38,61255483	Faial	Azores
FA3-09a	FA3	FA3-9	*Lobaria immixta*	14610	-28,69754	38,61241	Faial	Azores
FA3-09b	FA3	FA3-9	*Lobaria immixta*	14611	-28,69754	38,61241	Faial	Azores
FA3-09c	FA3	FA3-9	*Lobaria immixta*	14612	-28,69754	38,61241	Faial	Azores
FA3-09d	FA3	FA3-9	*Lobaria immixta*	14613	-28,69754	38,61241	Faial	Azores
FA3-09e	FA3	FA3-9	*Lobaria immixta*	14614	-28,69754	38,61241	Faial	Azores
FR1-01b	FR1	FR1-1	*Lobaria immixta*	15160	-31,164153	39,44671	Flores	Azores
FR1-01c	FR1	FR1-1	*Lobaria immixta*	15161	-31,164153	39,44671	Flores	Azores
FR1-01d	FR1	FR1-1	*Lobaria immixta*	15162	-31,164153	39,44671	Flores	Azores
FR1-01e	FR1	FR1-1	*Lobaria immixta*	15163	-31,164153	39,44671	Flores	Azores
FR1-01f	FR1	FR1-1	*Lobaria immixta*	15164	-31,164153	39,44671	Flores	Azores
FR1-01g	FR1	FR1-1	*Lobaria immixta*	15165	-31,164153	39,44671	Flores	Azores
FR1-01h	FR1	FR1-1	*Lobaria immixta*	15166	-31,164153	39,44671	Flores	Azores

ID	Pop	PopTree	Species	VoucherID	X	Y	Location	Area
FR1-01i	FR1	FR1-1	*Lobaria immixta*	15167	-31,164153	39,44671	Flores	Azores
FR1-11a	FR1	FR1-11	*Lobaria immixta*	15209	-31,16396679	39,44664757	Flores	Azores
FR1-11b	FR1	FR1-11	*Lobaria immixta*	15210	-31,16396679	39,44664757	Flores	Azores
FR1-11c	FR1	FR1-11	*Lobaria immixta*	15211	-31,16396679	39,44664757	Flores	Azores
FR1-13a	FR1	FR1-13	*Lobaria immixta*	15215	-31,16348836	39,44666616	Flores	Azores
FR1-13b	FR1	FR1-13	*Lobaria immixta*	15216	-31,16348836	39,44666616	Flores	Azores
FR1-13c	FR1	FR1-13	*Lobaria immixta*	15217	-31,16348836	39,44666616	Flores	Azores
FR1-14a	FR1	FR1-14	*Lobaria immixta*	15218	-31,16348273	39,44665337	Flores	Azores
FR1-14b	FR1	FR1-14	*Lobaria immixta*	15219	-31,16348273	39,44665337	Flores	Azores
FR1-14c	FR1	FR1-14	*Lobaria immixta*	15220	-31,16348273	39,44665337	Flores	Azores
FR1-16c	FR1	FR1-16	*Lobaria immixta*	15229	-31,16408215	39,44646888	Flores	Azores
FR1-16d	FR1	FR1-16	*Lobaria immixta*	15230	-31,16408215	39,44646888	Flores	Azores
FR1-16e	FR1	FR1-16	*Lobaria immixta*	15231	-31,16408215	39,44646888	Flores	Azores
FR1-16f	FR1	FR1-16	*Lobaria immixta*	15232	-31,16408215	39,44646888	Flores	Azores
FR1-17a	FR1	FR1-17	*Lobaria immixta*	15233	-31,16408746	39,44646706	Flores	Azores
FR1-17b	FR1	FR1-17	*Lobaria immixta*	15234	-31,16408746	39,44646706	Flores	Azores
FR1-17c	FR1	FR1-17	*Lobaria immixta*	15235	-31,16408746	39,44646706	Flores	Azores
FR1-18a	FR1	FR1-18	*Lobaria immixta*	15236	-31,1641137	39,44648485	Flores	Azores
FR1-19b	FR1	FR1-19	*Lobaria immixta*	15238	-31,1641172	39,44648722	Flores	Azores
FR1-19e	FR1	FR1-19	*Lobaria immixta*	15241	-31,1641172	39,44648722	Flores	Azores
FR1-19f	FR1	FR1-19	*Lobaria immixta*	15242	-31,1641172	39,44648722	Flores	Azores
FR1-02a	FR1	FR1-2	*Lobaria immixta*	15168	-31,1642104	39,44670309	Flores	Azores
FR1-02b	FR1	FR1-2	*Lobaria immixta*	15169	-31,1642104	39,44670309	Flores	Azores
FR1-02c	FR1	FR1-2	*Lobaria immixta*	15170	-31,1642104	39,44670309	Flores	Azores
FR1-02d	FR1	FR1-2	*Lobaria immixta*	15171	-31,1642104	39,44670309	Flores	Azores
FR1-20a	FR1	FR1-20	*Lobaria immixta*	15243	-31,16415971	39,44647266	Flores	Azores
FR1-20b	FR1	FR1-20	*Lobaria immixta*	15244	-31,16415971	39,44647266	Flores	Azores

ID	Pop	PopTree	Species	VoucherID	X	Y	Location	Area
FR1-20d	FR1	FR1-20	*Lobaria immixta*	15246	-31,16415971	39,44647266	Flores	Azores
FR1-21a	FR1	FR1-21	*Lobaria immixta*	15249	-31,16427369	39,44649011	Flores	Azores
FR1-21b	FR1	FR1-21	*Lobaria immixta*	15250	-31,16427369	39,44649011	Flores	Azores
FR1-03a	FR1	FR1-3	*Lobaria immixta*	15172	-31,16424484	39,44669894	Flores	Azores
FR1-03b	FR1	FR1-3	*Lobaria immixta*	15173	-31,16424484	39,44669894	Flores	Azores
FR1-03c	FR1	FR1-3	*Lobaria immixta*	15174	-31,16424484	39,44669894	Flores	Azores
FR1-04a	FR1	FR1-4	*Lobaria immixta*	15175	-31,16422496	39,44668961	Flores	Azores
FR1-04b	FR1	FR1-4	*Lobaria immixta*	15176	-31,16422496	39,44668961	Flores	Azores
FR1-04c	FR1	FR1-4	*Lobaria immixta*	15177	-31,16422496	39,44668961	Flores	Azores
FR1-04d	FR1	FR1-4	*Lobaria immixta*	15178	-31,16422496	39,44668961	Flores	Azores
FR1-04e	FR1	FR1-4	*Lobaria immixta*	15179	-31,16422496	39,44668961	Flores	Azores
FR1-04f	FR1	FR1-4	*Lobaria immixta*	15180	-31,16422496	39,44668961	Flores	Azores
FR1-04g	FR1	FR1-4	*Lobaria immixta*	15181	-31,16422496	39,44668961	Flores	Azores
FR1-05a	FR1	FR1-5	*Lobaria immixta*	15184	-31,16414365	39,44668833	Flores	Azores
FR1-05b	FR1	FR1-5	*Lobaria immixta*	15185	-31,16414365	39,44668833	Flores	Azores
FR1-05c	FR1	FR1-5	*Lobaria immixta*	15186	-31,16414365	39,44668833	Flores	Azores
FR1-06a	FR1	FR1-6	*Lobaria immixta*	15187	-31,16412267	39,44673033	Flores	Azores
FR1-07a	FR1	FR1-7	*Lobaria immixta*	15191	-31,16412267	39,44673033	Flores	Azores
FR1-07b	FR1	FR1-7	*Lobaria immixta*	15192	-31,16412267	39,44673033	Flores	Azores
FR1-08a	FR1	FR1-8	*Lobaria immixta*	15194	-31,16408769	39,44670661	Flores	Azores
FR1-08b	FR1	FR1-8	*Lobaria immixta*	15195	-31,16408769	39,44670661	Flores	Azores
FR1-08c	FR1	FR1-8	*Lobaria immixta*	15196	-31,16408769	39,44670661	Flores	Azores
FR1-08e	FR1	FR1-8	*Lobaria immixta*	15198	-31,16408769	39,44670661	Flores	Azores
FR1-08f	FR1	FR1-8	*Lobaria immixta*	15199	-31,16408769	39,44670661	Flores	Azores
FR1-08g	FR1	FR1-8	*Lobaria immixta*	15200	-31,16408769	39,44670661	Flores	Azores
FR1-09a	FR1	FR1-9	*Lobaria immixta*	15202	-31,16404396	39,44667696	Flores	Azores
FR1-09b	FR1	FR1-9	*Lobaria immixta*	15203	-31,16404396	39,44667696	Flores	Azores

ID	Pop	PopTree	Species	VoucherID	X	Y	Location	Area
FR1-09c	FR1	FR1-9	*Lobaria immixta*	15204	-31,16404396	39,44667696	Flores	Azores
FR2-01f	FR2	FR2-1	*Lobaria immixta*	15258	-31,251258	39,448117	Flores	Azores
FR2-13c	FR2	FR2-13	*Lobaria immixta*	15299	-31,25142816	39,44669667	Flores	Azores
FR2-14b	FR2	FR2-14	*Lobaria immixta*	15303	-31,25108965	39,4459296	Flores	Azores
FR2-14c	FR2	FR2-14	*Lobaria immixta*	15304	-31,25108965	39,4459296	Flores	Azores
FR2-18c	FR2	FR2-18	*Lobaria immixta*	15317	-31,25092229	39,44570323	Flores	Azores
FR2-02b	FR2	FR2-2	*Lobaria immixta*	15260	-31,25126946	39,44810682	Flores	Azores
FR3-01a	FR3	FR3-1	*Lobaria immixta*	15327	-31,154337	39,447192	Flores	Azores
FR3-01b	FR3	FR3-1	*Lobaria immixta*	15328	-31,154337	39,447192	Flores	Azores
FR3-01c	FR3	FR3-1	*Lobaria immixta*	15329	-31,154337	39,447192	Flores	Azores
FR3-01d	FR3	FR3-1	*Lobaria immixta*	15330	-31,154337	39,447192	Flores	Azores
FR3-01e	FR3	FR3-1	*Lobaria immixta*	15331	-31,154337	39,447192	Flores	Azores
FR3-01f	FR3	FR3-1	*Lobaria immixta*	15332	-31,154337	39,447192	Flores	Azores
FR3-11b	FR3	FR3-11	*Lobaria immixta*	15374	-31,15405975	39,4473737	Flores	Azores
FR3-11c	FR3	FR3-11	*Lobaria immixta*	15375	-31,15405975	39,4473737	Flores	Azores
FR3-11d	FR3	FR3-11	*Lobaria immixta*	15376	-31,15405975	39,4473737	Flores	Azores
FR3-12a	FR3	FR3-12	*Lobaria immixta*	15377	-31,15407136	39,44737388	Flores	Azores
FR3-12b	FR3	FR3-12	*Lobaria immixta*	15378	-31,15407136	39,44737388	Flores	Azores
FR3-12c	FR3	FR3-12	*Lobaria immixta*	15379	-31,15407136	39,44737388	Flores	Azores
FR3-12d	FR3	FR3-12	*Lobaria immixta*	15380	-31,15407136	39,44737388	Flores	Azores
FR3-13d	FR3	FR3-13	*Lobaria immixta*	15384	-31,15404937	39,4473797	Flores	Azores
FR3-13e	FR3	FR3-13	*Lobaria immixta*	15385	-31,15404937	39,4473797	Flores	Azores
FR3-14a	FR3	FR3-14	*Lobaria immixta*	15386	-31,15403894	39,44737572	Flores	Azores
FR3-14b	FR3	FR3-14	*Lobaria immixta*	15387	-31,15403894	39,44737572	Flores	Azores
FR3-14c	FR3	FR3-14	*Lobaria immixta*	15388	-31,15403894	39,44737572	Flores	Azores
FR3-14d	FR3	FR3-14	*Lobaria immixta*	15389	-31,15403894	39,44737572	Flores	Azores
FR3-15a	FR3	FR3-15	*Lobaria immixta*	15390	-31,15402924	39,44736449	Flores	Azores

ID	Pop	PopTree	Species	VoucherID	X	Y	Location	Area
FR3-15b	FR3	FR3-15	*Lobaria immixta*	15391	-31,15402924	39,44736449	Flores	Azores
FR3-16a	FR3	FR3-16	*Lobaria immixta*	15393	-31,15403714	39,44736299	Flores	Azores
FR3-16b	FR3	FR3-16	*Lobaria immixta*	15394	-31,15403714	39,44736299	Flores	Azores
FR3-16c	FR3	FR3-16	*Lobaria immixta*	15395	-31,15403714	39,44736299	Flores	Azores
FR3-16f	FR3	FR3-16	*Lobaria immixta*	15398	-31,15400216	39,44733927	Flores	Azores
FR3-16g	FR3	FR3-16	*Lobaria immixta*	15399	-31,15400216	39,44733927	Flores	Azores
FR3-16h	FR3	FR3-16	*Lobaria immixta*	15400	-31,15400216	39,44733927	Flores	Azores
FR3-17a	FR3	FR3-17	*Lobaria immixta*	15401	-31,15397312	39,44733881	Flores	Azores
FR3-17b	FR3	FR3-17	*Lobaria immixta*	15402	-31,15397312	39,44733881	Flores	Azores
FR3-17c	FR3	FR3-17	*Lobaria immixta*	15403	-31,15397312	39,44733881	Flores	Azores
FR3-17d	FR3	FR3-17	*Lobaria immixta*	15404	-31,15397312	39,44733881	Flores	Azores
FR3-17e	FR3	FR3-17	*Lobaria immixta*	15405	-31,15397312	39,44733881	Flores	Azores
FR3-17f	FR3	FR3-17	*Lobaria immixta*	15406	-31,15397312	39,44733881	Flores	Azores
FR3-17g	FR3	FR3-17	*Lobaria immixta*	15407	-31,15397312	39,44733881	Flores	Azores
FR3-17h	FR3	FR3-17	*Lobaria immixta*	15408	-31,15397312	39,44733881	Flores	Azores
FR3-18a	FR3	FR3-18	*Lobaria immixta*	15409	-31,15394596	39,44735575	Flores	Azores
FR3-18b	FR3	FR3-18	*Lobaria immixta*	15410	-31,15394596	39,44735575	Flores	Azores
FR3-19a	FR3	FR3-19	*Lobaria immixta*	15412	-31,15391244	39,4473807	Flores	Azores
FR3-19b	FR3	FR3-19	*Lobaria immixta*	15413	-31,15391244	39,4473807	Flores	Azores
FR3-19d	FR3	FR3-19	*Lobaria immixta*	15415	-31,15391244	39,4473807	Flores	Azores
FR3-02a	FR3	FR3-2	*Lobaria immixta*	15333	-31,15422302	39,44717454	Flores	Azores
FR3-02b	FR3	FR3-2	*Lobaria immixta*	15334	-31,15422302	39,44717454	Flores	Azores
FR3-02c	FR3	FR3-2	*Lobaria immixta*	15335	-31,15422302	39,44717454	Flores	Azores
FR3-02d	FR3	FR3-2	*Lobaria immixta*	15336	-31,15422302	39,44717454	Flores	Azores
FR3-02e	FR3	FR3-2	*Lobaria immixta*	15337	-31,15422302	39,44717454	Flores	Azores
FR3-02f	FR3	FR3-2	*Lobaria immixta*	15338	-31,15422302	39,44717454	Flores	Azores
FR3-20b	FR3	FR3-20	*Lobaria immixta*	15417	-31,15389886	39,44738917	Flores	Azores

**ID**	**Pop**	**PopTree**	**Species**	**VoucherID**	**X**	**Y**	**Location**	**Area**
FR3-20c	FR3	FR3-20	*Lobaria immixta*	15418	-31,15389886	39,44738917	Flores	Azores
FR3-21a	FR3	FR3-21	*Lobaria immixta*	15419	-31,15386832	39,4474022	Flores	Azores
FR3-21b	FR3	FR3-21	*Lobaria immixta*	15420	-31,15386832	39,4474022	Flores	Azores
FR3-21c	FR3	FR3-21	*Lobaria immixta*	15421	-31,15386832	39,4474022	Flores	Azores
FR3-21d	FR3	FR3-21	*Lobaria immixta*	15422	-31,15386832	39,4474022	Flores	Azores
FR3-21e	FR3	FR3-21	*Lobaria immixta*	15423	-31,15386832	39,4474022	Flores	Azores
FR3-22b	FR3	FR3-22	*Lobaria immixta*	15425	-31,15382212	39,44739832	Flores	Azores
FR3-22c	FR3	FR3-22	*Lobaria immixta*	15426	-31,15382212	39,44739832	Flores	Azores
FR3-22d	FR3	FR3-22	*Lobaria immixta*	15427	-31,15382212	39,44739832	Flores	Azores
FR3-03c	FR3	FR3-3	*Lobaria immixta*	15342	-31,15415085	39,44726703	Flores	Azores
FR3-03h	FR3	FR3-3	*Lobaria immixta*	15347	-31,15412987	39,44730903	Flores	Azores
FR3-04b	FR3	FR3-4	*Lobaria immixta*	15350	-31,15412082	39,44731468	Flores	Azores
FR3-04d	FR3	FR3-4	*Lobaria immixta*	15352	-31,15412082	39,44731468	Flores	Azores
FR3-05b	FR3	FR3-5	*Lobaria immixta*	15354	-31,1541071	39,4473491	Flores	Azores
FR3-06c	FR3	FR3-6	*Lobaria immixta*	15358	-31,15411627	39,44734097	Flores	Azores
FR3-07b	FR3	FR3-7	*Lobaria immixta*	15360	-31,15411177	39,44735864	Flores	Azores
FR3-07c	FR3	FR3-7	*Lobaria immixta*	15361	-31,15411177	39,44735864	Flores	Azores
FR3-08a	FR3	FR3-8	*Lobaria immixta*	15363	-31,15410183	39,44735398	Flores	Azores
FR3-08b	FR3	FR3-8	*Lobaria immixta*	15364	-31,15410183	39,44735398	Flores	Azores
FR3-08c	FR3	FR3-8	*Lobaria immixta*	15365	-31,15410183	39,44735398	Flores	Azores
FR3-08d	FR3	FR3-8	*Lobaria immixta*	15366	-31,15410183	39,44735398	Flores	Azores
FR3-08e	FR3	FR3-8	*Lobaria immixta*	15367	-31,15410183	39,44735398	Flores	Azores
FR3-09b	FR3	FR3-9	*Lobaria immixta*	15369	-31,15407887	39,44735674	Flores	Azores
FR3-09c	FR3	FR3-9	*Lobaria immixta*	15370	-31,15407887	39,44735674	Flores	Azores
FR3-09d	FR3	FR3-9	*Lobaria immixta*	15371	-31,15407887	39,44735674	Flores	Azores
FR3-09e	FR3	FR3-9	*Lobaria immixta*	15372	-31,15407887	39,44735674	Flores	Azores
MA1-01a	MA1	MA1-01	*Lobaria immixta*	25443	-25,08875	36,97206667	Santa Maria	Azores

ID	Pop	PopTree	Species	VoucherID	X	Y	Location	Area
MA1-01b	MA1	MA1-01	*Lobaria immixta*	25444	-25,08875	36,97206667	Santa Maria	Azores
MA1-01c	MA1	MA1-01	*Lobaria immixta*	25445	-25,08875	36,97206667	Santa Maria	Azores
MA1-01d	MA1	MA1-01	*Lobaria immixta*	25446	-25,08875	36,97206667	Santa Maria	Azores
MA1-01e	MA1	MA1-01	*Lobaria immixta*	25447	-25,08875	36,97206667	Santa Maria	Azores
MA1-02a	MA1	MA1-02	*Lobaria immixta*	25448	-25,08935	36,9726	Santa Maria	Azores
MA1-02b	MA1	MA1-02	*Lobaria immixta*	25449	-25,08935	36,9726	Santa Maria	Azores
MA1-02c	MA1	MA1-02	*Lobaria immixta*	25450	-25,08935	36,9726	Santa Maria	Azores
MA1-02d	MA1	MA1-02	*Lobaria immixta*	25451	-25,08935	36,9726	Santa Maria	Azores
MA1-02e	MA1	MA1-02	*Lobaria immixta*	25452	-25,08935	36,9726	Santa Maria	Azores
MA1-02f	MA1	MA1-02	*Lobaria immixta*	25453	-25,08935	36,9726	Santa Maria	Azores
MA1-02g	MA1	MA1-02	*Lobaria immixta*	25454	-25,08935	36,9726	Santa Maria	Azores
MA1-02h	MA1	MA1-02	*Lobaria immixta*	25455	-25,08935	36,9726	Santa Maria	Azores
MA1-03a	MA1	MA1-03	*Lobaria immixta*	25456	-25,08895	36,97263333	Santa Maria	Azores
MA1-03b	MA1	MA1-03	*Lobaria immixta*	25457	-25,08895	36,97263333	Santa Maria	Azores
MA1-03c	MA1	MA1-03	*Lobaria immixta*	25458	-25,08895	36,97263333	Santa Maria	Azores
MA1-03d	MA1	MA1-03	*Lobaria immixta*	25459	-25,08895	36,97263333	Santa Maria	Azores
MA1-03e	MA1	MA1-03	*Lobaria immixta*	25460	-25,08895	36,97263333	Santa Maria	Azores
MA1-03f	MA1	MA1-03	*Lobaria immixta*	25461	-25,08895	36,97263333	Santa Maria	Azores
MA1-04b	MA1	MA1-04	*Lobaria immixta*	25463	-25,08885	36,9724	Santa Maria	Azores
MA1-05a	MA1	MA1-05	*Lobaria immixta*	25467	-25,08493333	36,9675	Santa Maria	Azores
MA1-05b	MA1	MA1-05	*Lobaria immixta*	25468	-25,08493333	36,9675	Santa Maria	Azores
MA1-05e	MA1	MA1-05	*Lobaria immixta*	25471	-25,08493333	36,9675	Santa Maria	Azores
MA1-05f	MA1	MA1-05	*Lobaria immixta*	25472	-25,08493333	36,9675	Santa Maria	Azores
MA1-06a	MA1	MA1-06	*Lobaria immixta*	25474	-25,08491599	36,96749008	Santa Maria	Azores
MA1-06b	MA1	MA1-06	*Lobaria immixta*	25475	-25,08491599	36,96749008	Santa Maria	Azores
MA1-06k	MA1	MA1-06	*Lobaria immixta*	25484	-25,08491599	36,96749008	Santa Maria	Azores
MA1-07d	MA1	MA1-07	*Lobaria immixta*	25489	-25,08489354	36,96748972	Santa Maria	Azores

ID	Pop	PopTree	Species	VoucherID	X	Y	Location	Area
MA1-07e	MA1	MA1-07	*Lobaria immixta*	25490	-25,08489354	36,96748972	Santa Maria	Azores
MA1-07f	MA1	MA1-07	*Lobaria immixta*	25491	-25,08489354	36,96748972	Santa Maria	Azores
MA1-07j	MA1	MA1-07	*Lobaria immixta*	25493	-25,08489354	36,96748972	Santa Maria	Azores
MA1-08a	MA1	MA1-08	*Lobaria immixta*	25497	-25,08628333	36,97138333	Santa Maria	Azores
MA1-08b	MA1	MA1-08	*Lobaria immixta*	25498	-25,08628333	36,97138333	Santa Maria	Azores
MA1-08c	MA1	MA1-08	*Lobaria immixta*	25499	-25,08628333	36,97138333	Santa Maria	Azores
MA2-01a	MA2	MA2-01	*Lobaria immixta*	25500	-25,0904	36,9836	Santa Maria	Azores
MA2-01d	MA2	MA2-01	*Lobaria immixta*	25503	-25,0904	36,9836	Santa Maria	Azores
MA2-01e	MA2	MA2-01	*Lobaria immixta*	25504	-25,0904	36,9836	Santa Maria	Azores
MA2-01g	MA2	MA2-01	*Lobaria immixta*	25506	-25,0904	36,9836	Santa Maria	Azores
MA2-01h	MA2	MA2-01	*Lobaria immixta*	25507	-25,0904	36,9836	Santa Maria	Azores
MA2-01j	MA2	MA2-01	*Lobaria immixta*	25509	-25,0904	36,9836	Santa Maria	Azores
MA2-01k	MA2	MA2-01	*Lobaria immixta*	25510	-25,0904	36,9836	Santa Maria	Azores
MA2-02f	MA2	MA2-02	*Lobaria immixta*	25517	-25,0904	36,98358333	Santa Maria	Azores
MA2-02g	MA2	MA2-02	*Lobaria immixta*	25518	-25,0904	36,98358333	Santa Maria	Azores
MA2-02h	MA2	MA2-02	*Lobaria immixta*	25519	-25,0904	36,98358333	Santa Maria	Azores
MA2-02j	MA2	MA2-02	*Lobaria immixta*	25521	-25,0904	36,98358333	Santa Maria	Azores
MA2-03d	MA2	MA2-03	*Lobaria immixta*	25530	-25,09043333	36,98358333	Santa Maria	Azores
MA2-03e	MA2	MA2-03	*Lobaria immixta*	25531	-25,09043333	36,98358333	Santa Maria	Azores
MA2-03f	MA2	MA2-03	*Lobaria immixta*	25532	-25,09043333	36,98358333	Santa Maria	Azores
MA2-03g	MA2	MA2-03	*Lobaria immixta*	25533	-25,09043333	36,98358333	Santa Maria	Azores
MA2-03h	MA2	MA2-03	*Lobaria immixta*	25534	-25,09043333	36,98358333	Santa Maria	Azores
MA2-10a	MA2	MA2-10	*Lobaria immixta*	25601	-25,09046667	36,98355	Santa Maria	Azores
MA2-10k	MA2	MA2-10	*Lobaria immixta*	25611	-25,09046667	36,98355	Santa Maria	Azores
MA2-10l	MA2	MA2-10	*Lobaria immixta*	25612	-25,09046667	36,98355	Santa Maria	Azores
MA2-04d	MA2	MA2-4	*Lobaria immixta*	25540	-25,09011667	36,98396667	Santa Maria	Azores
MA2-04i	MA2	MA2-4	*Lobaria immixta*	25545	-25,09011667	36,98396667	Santa Maria	Azores

**ID**	**Pop**	**PopTree**	**Species**	**VoucherID**	**X**	**Y**	**Location**	**Area**
MA2-04j	MA2	MA2-4	*Lobaria immixta*	25546	-25,09011667	36,98396667	Santa Maria	Azores
MA2-05a	MA2	MA2-5	*Lobaria immixta*	25547	-25,09028333	36,98371667	Santa Maria	Azores
MA2-05b	MA2	MA2-5	*Lobaria immixta*	25548	-25,09028333	36,98371667	Santa Maria	Azores
MA2-05f	MA2	MA2-5	*Lobaria immixta*	25552	-25,09028333	36,98371667	Santa Maria	Azores
MA2-06a	MA2	MA2-6	*Lobaria immixta*	25558	-25,09038333	36,98356667	Santa Maria	Azores
MA2-06j	MA2	MA2-6	*Lobaria immixta*	25567	-25,09038333	36,98356667	Santa Maria	Azores
MA2-06k	MA2	MA2-6	*Lobaria immixta*	25568	-25,09038333	36,98356667	Santa Maria	Azores
MA2-07a	MA2	MA2-7	*Lobaria immixta*	25569	-25,0904	36,98355	Santa Maria	Azores
MA2-07c	MA2	MA2-7	*Lobaria immixta*	25571	-25,0904	36,98355	Santa Maria	Azores
MA2-07f	MA2	MA2-7	*Lobaria immixta*	25574	-25,0904	36,98355	Santa Maria	Azores
MA2-07i	MA2	MA2-7	*Lobaria immixta*	25577	-25,0904	36,98355	Santa Maria	Azores
MA2-08e	MA2	MA2-8	*Lobaria immixta*	25583	-25,09043333	36,98351667	Santa Maria	Azores
MA2-08f	MA2	MA2-8	*Lobaria immixta*	25584	-25,09043333	36,98351667	Santa Maria	Azores
MA2-08g	MA2	MA2-8	*Lobaria immixta*	25585	-25,09043333	36,98351667	Santa Maria	Azores
MA2-08l	MA2	MA2-8	*Lobaria immixta*	25590	-25,09043333	36,98351667	Santa Maria	Azores
MA2-09c	MA2	MA2-9	*Lobaria immixta*	25593	-25,09048333	36,9835	Santa Maria	Azores
MA2-09d	MA2	MA2-9	*Lobaria immixta*	25594	-25,09048333	36,9835	Santa Maria	Azores
PI1-14a	PI1	PI1-14	*Lobaria immixta*	15120	-28,42782205	38,46802396	Pico	Azores
PI2-01a	PI2	PI2-1	*Lobaria immixta*	14956	-28,2765	38,46231	Pico	Azores
PI2-01b	PI2	PI2-1	*Lobaria immixta*	14957	-28,2765	38,46231	Pico	Azores
PI2-01c	PI2	PI2-1	*Lobaria immixta*	14958	-28,2765	38,46231	Pico	Azores
PI2-02a	PI2	PI2-2	*Lobaria immixta*	14959	-28,27650772	38,46230828	Pico	Azores
PI2-02b	PI2	PI2-2	*Lobaria immixta*	14960	-28,27650772	38,46230828	Pico	Azores
PI2-02c	PI2	PI2-2	*Lobaria immixta*	14961	-28,27650772	38,46230828	Pico	Azores
PI2-03a	PI2	PI2-3	*Lobaria immixta*	14962	-28,27649323	38,46232225	Pico	Azores
PI2-03b	PI2	PI2-3	*Lobaria immixta*	14963	-28,27649323	38,46232225	Pico	Azores
PI2-03c	PI2	PI2-3	*Lobaria immixta*	14964	-28,27649323	38,46232225	Pico	Azores

ID	Pop	PopTree	Species	VoucherID	X	Y	Location	Area
PI2-04a	PI2	PI2-4	*Lobaria immixta*	14965	-28,27649554	38,46234017	Pico	Azores
PI2-04b	PI2	PI2-4	*Lobaria immixta*	14966	-28,27649554	38,46234017	Pico	Azores
PI2-04c	PI2	PI2-4	*Lobaria immixta*	14967	-28,27649554	38,46234017	Pico	Azores
PI3-10a	PI3	PI3-10	*Lobaria immixta*	14984	-28,25744372	38,45602562	Pico	Azores
PI3-10b	PI3	PI3-10	*Lobaria immixta*	14985	-28,25744372	38,45602562	Pico	Azores
PI3-11a	PI3	PI3-11	*Lobaria immixta*	14986	-28,25741529	38,45601041	Pico	Azores
PI3-11b	PI3	PI3-11	*Lobaria immixta*	14987	-28,25741529	38,45601041	Pico	Azores
PI3-11c	PI3	PI3-11	*Lobaria immixta*	14988	-28,25741529	38,45601041	Pico	Azores
PI3-12a	PI3	PI3-12	*Lobaria immixta*	14989	-28,25735942	38,45602039	Pico	Azores
PI3-13d	PI3	PI3-13	*Lobaria immixta*	14993	-28,257245	38,45601532	Pico	Azores
PI3-13e	PI3	PI3-13	*Lobaria immixta*	14994	-28,257245	38,45601532	Pico	Azores
PI3-13f	PI3	PI3-13	*Lobaria immixta*	14995	-28,257245	38,45601532	Pico	Azores
PI3-13g	PI3	PI3-13	*Lobaria immixta*	14996	-28,257245	38,45601532	Pico	Azores
PI3-13h	PI3	PI3-13	*Lobaria immixta*	14997	-28,257245	38,45601532	Pico	Azores
PI3-14a	PI3	PI3-14	*Lobaria immixta*	14998	-28,25719405	38,45599472	Pico	Azores
PI3-14b	PI3	PI3-14	*Lobaria immixta*	14999	-28,25719405	38,45599472	Pico	Azores
PI3-14c	PI3	PI3-14	*Lobaria immixta*	15000	-28,25719405	38,45599472	Pico	Azores
PI3-15b	PI3	PI3-15	*Lobaria immixta*	15002	-28,25719031	38,45599131	Pico	Azores
PI3-16a	PI3	PI3-16	*Lobaria immixta*	15003	-28,25719566	38,45598971	Pico	Azores
PI3-16b	PI3	PI3-16	*Lobaria immixta*	15004	-28,25719566	38,45598971	Pico	Azores
PI3-16c	PI3	PI3-16	*Lobaria immixta*	15005	-28,25719566	38,45598971	Pico	Azores
PI3-16d	PI3	PI3-16	*Lobaria immixta*	15006	-28,25719566	38,45598971	Pico	Azores
PI3-05e	PI3	PI3-5	*Lobaria immixta*	14972	-28,25745	38,45609	Pico	Azores
PI3-05f	PI3	PI3-5	*Lobaria immixta*	14973	-28,25745	38,45609	Pico	Azores
PI3-06a	PI3	PI3-6	*Lobaria immixta*	14974	-28,25743869	38,45608856	Pico	Azores
PI3-06b	PI3	PI3-6	*Lobaria immixta*	14975	-28,25743869	38,45608856	Pico	Azores
PI3-06c	PI3	PI3-6	*Lobaria immixta*	14976	-28,25743869	38,45608856	Pico	Azores

**ID**	**Pop**	**PopTree**	**Species**	**VoucherID**	**X**	**Y**	**Location**	**Area**
PI3-07a	PI3	PI3-7	*Lobaria immixta*	14977	-28,25743169	38,45605294	Pico	Azores
PI3-07b	PI3	PI3-7	*Lobaria immixta*	14978	-28,25743169	38,45605294	Pico	Azores
PI3-07c	PI3	PI3-7	*Lobaria immixta*	14979	-28,25743169	38,45605294	Pico	Azores
PI3-08a	PI3	PI3-8	*Lobaria immixta*	14980	-28,25742546	38,45603559	Pico	Azores
PI3-09a	PI3	PI3-9	*Lobaria immixta*	14981	-28,25743301	38,45602881	Pico	Azores
PI3-09b	PI3	PI3-9	*Lobaria immixta*	14982	-28,25743301	38,45602881	Pico	Azores
PI3-09c	PI3	PI3-9	*Lobaria immixta*	14983	-28,25743301	38,45602881	Pico	Azores
PI4-03a	PI4	PI4-3	*Lobaria immixta*	15018	-28,25331578	38,4805659	Pico	Azores
PI4-03b	PI4	PI4-3	*Lobaria immixta*	15019	-28,25331578	38,4805659	Pico	Azores
PI4-03c	PI4	PI4-3	*Lobaria immixta*	15020	-28,25331578	38,4805659	Pico	Azores
PI4-03d	PI4	PI4-3	*Lobaria immixta*	15021	-28,25331578	38,4805659	Pico	Azores
PI4-03e	PI4	PI4-3	*Lobaria immixta*	15022	-28,25331578	38,4805659	Pico	Azores
PM10-01a	PM10	PM10-1	*Lobaria immixta*	12403	-16,876169	32,763192	Madeira	Madeira
PM10-01b	PM10	PM10-1	*Lobaria immixta*	12404	-16,876169	32,763192	Madeira	Madeira
PM10-01c	PM10	PM10-1	*Lobaria immixta*	12405	-16,876169	32,763192	Madeira	Madeira
PM10-01e	PM10	PM10-1	*Lobaria immixta*	12414	-16,876169	32,763192	Madeira	Madeira
PM10-02a	PM10	PM10-2	*Lobaria immixta*	12407	-16,87622235	32,7631912	Madeira	Madeira
PM10-02b	PM10	PM10-2	*Lobaria immixta*	12408	-16,87622235	32,7631912	Madeira	Madeira
PM10-02c	PM10	PM10-2	*Lobaria immixta*	12409	-16,87622235	32,7631912	Madeira	Madeira
PM10-04a	PM10	PM10-4	*Lobaria immixta*	12415	-16,87632906	32,7631896	Madeira	Madeira
PM10-04b	PM10	PM10-4	*Lobaria immixta*	12416	-16,87632906	32,7631896	Madeira	Madeira
PM10-04c	PM10	PM10-4	*Lobaria immixta*	12417	-16,87632906	32,7631896	Madeira	Madeira
PM10-04d	PM10	PM10-4	*Lobaria immixta*	12418	-16,87632906	32,7631896	Madeira	Madeira
PM10-05a	PM10	PM10-5	*Lobaria immixta*	12419	-16,87638241	32,76318881	Madeira	Madeira
PM10-05b	PM10	PM10-5	*Lobaria immixta*	12420	-16,87638241	32,76318881	Madeira	Madeira
PM10-06a	PM10	PM10-6	*Lobaria immixta*	12424	-16,87643577	32,76318801	Madeira	Madeira
PM10-06b	PM10	PM10-6	*Lobaria immixta*	12425	-16,87643577	32,76318801	Madeira	Madeira

ID	Pop	PopTree	Species	VoucherID	X	Y	Location	Area
PM10-06c	PM10	PM10-6	*Lobaria immixta*	12426	-16,87643577	32,76318801	Madeira	Madeira
PM10-07d	PM10	PM10-7	*Lobaria immixta*	12430	-16,87648912	32,76318721	Madeira	Madeira
PM10-07e	PM10	PM10-7	*Lobaria immixta*	12431	-16,87648912	32,76318721	Madeira	Madeira
PM11-01a	PM11	PM11-1	*Lobaria immixta*	12432	-16,882969	32,7384	Madeira	Madeira
PM11-01b	PM11	PM11-1	*Lobaria immixta*	12433	-16,882969	32,7384	Madeira	Madeira
PM11-01c	PM11	PM11-1	*Lobaria immixta*	12434	-16,882969	32,7384	Madeira	Madeira
PM11-10a	PM11	PM11-10	*Lobaria immixta*	12457	-16,88215017	32,73867761	Madeira	Madeira
PM11-11d	PM11	PM11-11	*Lobaria immixta*	12462	-16,88176343	32,73883014	Madeira	Madeira
PM11-11e	PM11	PM11-11	*Lobaria immixta*	12463	-16,88176343	32,73883014	Madeira	Madeira
PM11-11f	PM11	PM11-11	*Lobaria immixta*	12464	-16,88176343	32,73883014	Madeira	Madeira
PM11-12c	PM11	PM11-12	*Lobaria immixta*	12468	-16,88163739	32,73870768	Madeira	Madeira
PM11-12d	PM11	PM11-12	*Lobaria immixta*	12469	-16,88163739	32,73870768	Madeira	Madeira
PM11-12e	PM11	PM11-12	*Lobaria immixta*	12470	-16,88163739	32,73870768	Madeira	Madeira
PM11-13a	PM11	PM11-13	*Lobaria immixta*	12471	-16,88146234	32,73853759	Madeira	Madeira
PM11-13b	PM11	PM11-13	*Lobaria immixta*	12472	-16,88146234	32,73853759	Madeira	Madeira
PM11-13c	PM11	PM11-13	*Lobaria immixta*	12473	-16,88146234	32,73853759	Madeira	Madeira
PM11-14c	PM11	PM11-14	*Lobaria immixta*	12478	-16,88142032	32,73849677	Madeira	Madeira
PM11-14d	PM11	PM11-14	*Lobaria immixta*	12479	-16,88142032	32,73849677	Madeira	Madeira
PM11-15a	PM11	PM11-15	*Lobaria immixta*	12480	-16,88136431	32,73844234	Madeira	Madeira
PM11-15b	PM11	PM11-15	*Lobaria immixta*	12481	-16,88136431	32,73844234	Madeira	Madeira
PM11-02a	PM11	PM11-2	*Lobaria immixta*	12438	-16,88288522	32,73841378	Madeira	Madeira
PM11-02b	PM11	PM11-2	*Lobaria immixta*	12439	-16,88288522	32,73841378	Madeira	Madeira
PM11-04	PM11	PM11-4	*Lobaria immixta*	12442	-16,88293416	32,73854382	Madeira	Madeira
PM11-05a	PM11	PM11-5	*Lobaria immixta*	12443	-16,88268926	32,73878208	Madeira	Madeira
PM11-05b	PM11	PM11-5	*Lobaria immixta*	12444	-16,88268926	32,73878208	Madeira	Madeira
PM11-05c	PM11	PM11-5	*Lobaria immixta*	12486	-16,88268926	32,73878208	Madeira	Madeira
PM11-06a	PM11	PM11-6	*Lobaria immixta*	12445	-16,88262773	32,73879701	Madeira	Madeira

ID	Pop	PopTree	Species	VoucherID	X	Y	Location	Area
PM11-06b	PM11	PM11-6	*Lobaria immixta*	12446	-16,88262773	32,73879701	Madeira	Madeira
PM11-06c	PM11	PM11-6	*Lobaria immixta*	12447	-16,88262773	32,73879701	Madeira	Madeira
PM11-08a	PM11	PM11-8	*Lobaria immixta*	12450	-16,8826322	32,73883329	Madeira	Madeira
PM11-08b	PM11	PM11-8	*Lobaria immixta*	12451	-16,8826322	32,73883329	Madeira	Madeira
PM11-08c	PM11	PM11-8	*Lobaria immixta*	12452	-16,8826322	32,73883329	Madeira	Madeira
PM11-08d	PM11	PM11-8	*Lobaria immixta*	12453	-16,8826322	32,73883329	Madeira	Madeira
PM11-09a	PM11	PM11-9	*Lobaria immixta*	12454	-16,88246632	32,73871984	Madeira	Madeira
PM11-09b	PM11	PM11-9	*Lobaria immixta*	12455	-16,88246632	32,73871984	Madeira	Madeira
PM11-09d	PM11	PM11-9	*Lobaria immixta*	12489	-16,88246632	32,73871984	Madeira	Madeira
PM12-10b	PM12	PM12-10	*Lobaria immixta*	12533	-17,01617491	32,76162247	Madeira	Madeira
PM12-10c	PM12	PM12-10	*Lobaria immixta*	12534	-17,01617491	32,76162247	Madeira	Madeira
PM12-11a	PM12	PM12-11	*Lobaria immixta*	12536	-17,01600466	32,76173123	Madeira	Madeira
PM12-11b	PM12	PM12-11	*Lobaria immixta*	12537	-17,01600466	32,76173123	Madeira	Madeira
PM12-11c	PM12	PM12-11	*Lobaria immixta*	12538	-17,01600466	32,76173123	Madeira	Madeira
PM12-12a	PM12	PM12-12	*Lobaria immixta*	12539	-17,01600033	32,76180328	Madeira	Madeira
PM12-13a	PM12	PM12-13	*Lobaria immixta*	12543	-17,01663508	32,76014917	Madeira	Madeira
PM12-13b	PM12	PM12-13	*Lobaria immixta*	12544	-17,01663508	32,76014917	Madeira	Madeira
PM12-02a	PM12	PM12-2	*Lobaria immixta*	12491	-17,01602013	32,76156538	Madeira	Madeira
PM12-02b	PM12	PM12-2	*Lobaria immixta*	12492	-17,01602013	32,76156538	Madeira	Madeira
PM12-02c	PM12	PM12-2	*Lobaria immixta*	12493	-17,01602013	32,76156538	Madeira	Madeira
PM12-03a	PM12	PM12-3	*Lobaria immixta*	12497	-17,01604107	32,76156191	Madeira	Madeira
PM12-03b	PM12	PM12-3	*Lobaria immixta*	12498	-17,01604107	32,76156191	Madeira	Madeira
PM12-03c	PM12	PM12-3	*Lobaria immixta*	12499	-17,01604107	32,76156191	Madeira	Madeira
PM12-04a	PM12	PM12-4	*Lobaria immixta*	12502	-17,0160617	32,76153034	Madeira	Madeira
PM12-04c	PM12	PM12-4	*Lobaria immixta*	12504	-17,0160617	32,76153034	Madeira	Madeira
PM12-05a	PM12	PM12-5	*Lobaria immixta*	12509	-17,01616672	32,76159226	Madeira	Madeira
PM12-05b	PM12	PM12-5	*Lobaria immixta*	12510	-17,01616672	32,76159226	Madeira	Madeira

ID	Pop	PopTree	Species	VoucherID	X	Y	Location	Area
PM12-05c	PM12	PM12-5	*Lobaria immixta*	12511	-17,01616672	32,76159226	Madeira	Madeira
PM12-06a	PM12	PM12-6	*Lobaria immixta*	12516	-17,01617206	32,76159218	Madeira	Madeira
PM12-06b	PM12	PM12-6	*Lobaria immixta*	12517	-17,01617206	32,76159218	Madeira	Madeira
PM12-07a	PM12	PM12-7	*Lobaria immixta*	12519	-17,01619248	32,76159742	Madeira	Madeira
PM12-07b	PM12	PM12-7	*Lobaria immixta*	12520	-17,01619248	32,76159742	Madeira	Madeira
PM12-07c	PM12	PM12-7	*Lobaria immixta*	12521	-17,01619248	32,76159742	Madeira	Madeira
PM12-07d	PM12	PM12-7	*Lobaria immixta*	12522	-17,01619248	32,76159742	Madeira	Madeira
PM12-08a	PM12	PM12-8	*Lobaria immixta*	12523	-17,01626628	32,76160719	Madeira	Madeira
PM12-08b	PM12	PM12-8	*Lobaria immixta*	12524	-17,01626628	32,76160719	Madeira	Madeira
PM12-08c	PM12	PM12-8	*Lobaria immixta*	12525	-17,01626628	32,76160719	Madeira	Madeira
PM12-09c	PM12	PM12-9	*Lobaria immixta*	12531	-17,01620654	32,76162666	Madeira	Madeira
PM13-01a	PM13	PM13-1	*Lobaria immixta*	12573	-17,13167	32,76333	Madeira	Madeira
PM13-01b	PM13	PM13-1	*Lobaria immixta*	12574	-17,13167	32,76333	Madeira	Madeira
PM13-01c	PM13	PM13-1	*Lobaria immixta*	12575	-17,13167	32,76333	Madeira	Madeira
PM13-11c	PM13	PM13-11	*Lobaria immixta*	12635	-17,13152084	32,76342911	Madeira	Madeira
PM13-12a	PM13	PM13-12	*Lobaria immixta*	12642	-17,1315229	32,76343795	Madeira	Madeira
PM13-12b	PM13	PM13-12	*Lobaria immixta*	12643	-17,1315229	32,76343795	Madeira	Madeira
PM13-12c	PM13	PM13-12	*Lobaria immixta*	12644	-17,1315229	32,76343795	Madeira	Madeira
PM13-13d	PM13	PM13-13	*Lobaria immixta*	12651	-17,13152783	32,76341122	Madeira	Madeira
PM13-13e	PM13	PM13-13	*Lobaria immixta*	12652	-17,13152783	32,76341122	Madeira	Madeira
PM13-13f	PM13	PM13-13	*Lobaria immixta*	12653	-17,13152783	32,76341122	Madeira	Madeira
PM13-13g	PM13	PM13-13	*Lobaria immixta*	12654	-17,13152783	32,76341122	Madeira	Madeira
PM13-14b	PM13	PM13-14	*Lobaria immixta*	12656	-17,13147612	32,76353922	Madeira	Madeira
PM13-14c	PM13	PM13-14	*Lobaria immixta*	12657	-17,13147612	32,76353922	Madeira	Madeira
PM13-14d	PM13	PM13-14	*Lobaria immixta*	12658	-17,13147612	32,76353922	Madeira	Madeira
PM13-14e	PM13	PM13-14	*Lobaria immixta*	12659	-17,13147612	32,76353922	Madeira	Madeira
PM13-15a	PM13	PM13-15	*Lobaria immixta*	12661	-17,13158146	32,76364105	Madeira	Madeira

ID	Pop	PopTree	Species	VoucherID	X	Y	Location	Area
PM13-15b	PM13	PM13-15	*Lobaria immixta*	12662	-17,13158146	32,76364105	Madeira	Madeira
PM13-15c	PM13	PM13-15	*Lobaria immixta*	12663	-17,13158146	32,76364105	Madeira	Madeira
PM13-16a	PM13	PM13-16	*Lobaria immixta*	12664	-17,13158949	32,76363512	Madeira	Madeira
PM13-16b	PM13	PM13-16	*Lobaria immixta*	12665	-17,13158949	32,76363512	Madeira	Madeira
PM13-16c	PM13	PM13-16	*Lobaria immixta*	12666	-17,13158949	32,76363512	Madeira	Madeira
PM13-17b	PM13	PM13-17	*Lobaria immixta*	12673	-17,13166646	32,76369758	Madeira	Madeira
PM13-17c	PM13	PM13-17	*Lobaria immixta*	12674	-17,13166646	32,76369758	Madeira	Madeira
PM13-17d	PM13	PM13-17	*Lobaria immixta*	12675	-17,13166646	32,76369758	Madeira	Madeira
PM13-17e	PM13	PM13-17	*Lobaria immixta*	12676	-17,13166646	32,76369758	Madeira	Madeira
PM13-18a	PM13	PM13-18	*Lobaria immixta*	12681	-17,13166244	32,76370054	Madeira	Madeira
PM13-18b	PM13	PM13-18	*Lobaria immixta*	12682	-17,13166244	32,76370054	Madeira	Madeira
PM13-18c	PM13	PM13-18	*Lobaria immixta*	12683	-17,13166244	32,76370054	Madeira	Madeira
PM13-19a	PM13	PM13-19	*Lobaria immixta*	12686	-17,13165947	32,76370429	Madeira	Madeira
PM13-19b	PM13	PM13-19	*Lobaria immixta*	12687	-17,13165947	32,76370429	Madeira	Madeira
PM13-19c	PM13	PM13-19	*Lobaria immixta*	12688	-17,13165947	32,76370429	Madeira	Madeira
PM13-02f	PM13	PM13-2	*Lobaria immixta*	12581	-17,13163653	32,76336511	Madeira	Madeira
PM13-02g	PM13	PM13-2	*Lobaria immixta*	12582	-17,13163653	32,76336511	Madeira	Madeira
PM13-20a	PM13	PM13-20	*Lobaria immixta*	12689	-17,13165968	32,7637133	Madeira	Madeira
PM13-20b	PM13	PM13-20	*Lobaria immixta*	12690	-17,13165968	32,7637133	Madeira	Madeira
PM13-20c	PM13	PM13-20	*Lobaria immixta*	12691	-17,13165968	32,7637133	Madeira	Madeira
PM13-20d	PM13	PM13-20	*Lobaria immixta*	12692	-17,13165968	32,7637133	Madeira	Madeira
PM13-22a	PM13	PM13-22	*Lobaria immixta*	12700	-17,13170523	32,76371057	Madeira	Madeira
PM13-22b	PM13	PM13-22	*Lobaria immixta*	12701	-17,13170523	32,76371057	Madeira	Madeira
PM13-22c	PM13	PM13-22	*Lobaria immixta*	12702	-17,13170523	32,76371057	Madeira	Madeira
PM13-22e	PM13	PM13-22	*Lobaria immixta*	12704	-17,13170523	32,76371057	Madeira	Madeira
PM13-23b	PM13	PM13-23	*Lobaria immixta*	12710	-17,1317268	32,76366934	Madeira	Madeira
PM13-03b	PM13	PM13-3	*Lobaria immixta*	12584	-17,13158484	32,76339702	Madeira	Madeira

ID	Pop	PopTree	Species	VoucherID	X	Y	Location	Area
PM13-03c	PM13	PM13-3	*Lobaria immixta*	12585	-17,13158484	32,76339702	Madeira	Madeira
PM13-03d	PM13	PM13-3	*Lobaria immixta*	12586	-17,13158484	32,76339702	Madeira	Madeira
PM13-04f	PM13	PM13-4	*Lobaria immixta*	12639	-17,13159743	32,76341159	Madeira	Madeira
PM13-04g	PM13	PM13-4	*Lobaria immixta*	12640	-17,13159743	32,76341159	Madeira	Madeira
PM13-04h	PM13	PM13-4	*Lobaria immixta*	12641	-17,13159743	32,76341159	Madeira	Madeira
PM13-05c	PM13	PM13-5	*Lobaria immixta*	12597	-17,13160257	32,76340369	Madeira	Madeira
PM13-05d	PM13	PM13-5	*Lobaria immixta*	12598	-17,13160257	32,76340369	Madeira	Madeira
PM13-05e	PM13	PM13-5	*Lobaria immixta*	12599	-17,13160257	32,76340369	Madeira	Madeira
PM13-05f	PM13	PM13-5	*Lobaria immixta*	12600	-17,13160257	32,76340369	Madeira	Madeira
PM13-05g	PM13	PM13-5	*Lobaria immixta*	12601	-17,13160257	32,76340369	Madeira	Madeira
PM13-05h	PM13	PM13-5	*Lobaria immixta*	12602	-17,13160257	32,76340369	Madeira	Madeira
PM13-07a	PM13	PM13-7	*Lobaria immixta*	12608	-17,13158559	32,7634285	Madeira	Madeira
PM13-07c	PM13	PM13-7	*Lobaria immixta*	12610	-17,13158559	32,7634285	Madeira	Madeira
PM13-07d	PM13	PM13-7	*Lobaria immixta*	12611	-17,13158559	32,7634285	Madeira	Madeira
PM13-09g	PM13	PM13-9	*Lobaria immixta*	12625	-17,13152848	32,76343994	Madeira	Madeira
PM13-09h	PM13	PM13-9	*Lobaria immixta*	12626	-17,13152848	32,76343994	Madeira	Madeira
PM14-01a	PM14	PM14-1	*Lobaria immixta*	12737	-17,1893	32,83067	Madeira	Madeira
PM14-10d	PM14	PM14-10	*Lobaria immixta*	12796	-17,18926558	32,83075518	Madeira	Madeira
PM14-10e	PM14	PM14-10	*Lobaria immixta*	12797	-17,18926558	32,83075518	Madeira	Madeira
PM14-10f	PM14	PM14-10	*Lobaria immixta*	12798	-17,18926558	32,83075518	Madeira	Madeira
PM14-11a	PM14	PM14-11	*Lobaria immixta*	12800	-17,18924437	32,83075304	Madeira	Madeira
PM14-11b	PM14	PM14-11	*Lobaria immixta*	12801	-17,18924437	32,83075304	Madeira	Madeira
PM14-11c	PM14	PM14-11	*Lobaria immixta*	12802	-17,18924437	32,83075304	Madeira	Madeira
PM14-11d	PM14	PM14-11	*Lobaria immixta*	12803	-17,18924437	32,83075304	Madeira	Madeira
PM14-11j	PM14	PM14-11	*Lobaria immixta*	12809	-17,18924437	32,83075304	Madeira	Madeira
PM14-12a	PM14	PM14-12	*Lobaria immixta*	12810	-17,18927801	32,83081934	Madeira	Madeira
PM14-12b	PM14	PM14-12	*Lobaria immixta*	12811	-17,18927801	32,83081934	Madeira	Madeira

ID	Pop	PopTree	Species	VoucherID	X	Y	Location	Area
PM14-12c	PM14	PM14-12	*Lobaria immixta*	12812	-17,18927801	32,83081934	Madeira	Madeira
PM14-13c	PM14	PM14-13	*Lobaria immixta*	12820	-17,18929137	32,83090879	Madeira	Madeira
PM14-13d	PM14	PM14-13	*Lobaria immixta*	12821	-17,18929137	32,83090879	Madeira	Madeira
PM14-14a	PM14	PM14-14	*Lobaria immixta*	12826	-17,18930166	32,83089299	Madeira	Madeira
PM14-14b	PM14	PM14-14	*Lobaria immixta*	12827	-17,18930166	32,83089299	Madeira	Madeira
PM14-14c	PM14	PM14-14	*Lobaria immixta*	12828	-17,18930166	32,83089299	Madeira	Madeira
PM14-15a	PM14	PM14-15	*Lobaria immixta*	12831	-17,189312	32,83086739	Madeira	Madeira
PM14-16d	PM14	PM14-16	*Lobaria immixta*	12836	-17,18922965	32,83099378	Madeira	Madeira
PM14-16e	PM14	PM14-16	*Lobaria immixta*	12837	-17,18922965	32,83099378	Madeira	Madeira
PM14-17a	PM14	PM14-17	*Lobaria immixta*	12839	-17,18921723	32,83101872	Madeira	Madeira
PM14-17e	PM14	PM14-17	*Lobaria immixta*	12843	-17,18921723	32,83101872	Madeira	Madeira
PM14-18a	PM14	PM14-18	*Lobaria immixta*	12844	-17,18919612	32,83101596	Madeira	Madeira
PM14-18b	PM14	PM14-18	*Lobaria immixta*	12845	-17,18919612	32,83101596	Madeira	Madeira
PM14-18c	PM14	PM14-18	*Lobaria immixta*	12846	-17,18919612	32,83101596	Madeira	Madeira
PM14-19a	PM14	PM14-19	*Lobaria immixta*	12850	-17,18919878	32,83101006	Madeira	Madeira
PM14-19b	PM14	PM14-19	*Lobaria immixta*	12851	-17,18919878	32,83101006	Madeira	Madeira
PM14-02a	PM14	PM14-2	*Lobaria immixta*	12746	-17,18929511	32,83066818	Madeira	Madeira
PM14-02b	PM14	PM14-2	*Lobaria immixta*	12747	-17,18929511	32,83066818	Madeira	Madeira
PM14-20c	PM14	PM14-20	*Lobaria immixta*	12857	-17,18917886	32,83101658	Madeira	Madeira
PM14-20d	PM14	PM14-20	*Lobaria immixta*	12858	-17,18917886	32,83101658	Madeira	Madeira
PM14-20e	PM14	PM14-20	*Lobaria immixta*	12859	-17,18917886	32,83101658	Madeira	Madeira
PM14-03a	PM14	PM14-3	*Lobaria immixta*	12755	-17,18930004	32,83067293	Madeira	Madeira
PM14-04a	PM14	PM14-4	*Lobaria immixta*	12762	-17,18928282	32,8307156	Madeira	Madeira
PM14-04b	PM14	PM14-4	*Lobaria immixta*	12763	-17,18928282	32,8307156	Madeira	Madeira
PM14-05a	PM14	PM14-5	*Lobaria immixta*	12765	-17,18928177	32,83073361	Madeira	Madeira
PM14-05b	PM14	PM14-5	*Lobaria immixta*	12766	-17,18928177	32,83073361	Madeira	Madeira
PM14-05c	PM14	PM14-5	*Lobaria immixta*	12767	-17,18928177	32,83073361	Madeira	Madeira

**ID**	**Pop**	**PopTree**	**Species**	**VoucherID**	**X**	**Y**	**Location**	**Area**
PM14-05d	PM14	PM14-5	*Lobaria immixta*	12768	-17,18928177	32,83073361	Madeira	Madeira
PM14-06a	PM14	PM14-6	*Lobaria immixta*	12771	-17,18933791	32,83075968	Madeira	Madeira
PM14-06b	PM14	PM14-6	*Lobaria immixta*	12772	-17,18933791	32,83075968	Madeira	Madeira
PM14-07a	PM14	PM14-7	*Lobaria immixta*	12777	-17,1893545	32,83078282	Madeira	Madeira
PM14-07b	PM14	PM14-7	*Lobaria immixta*	12778	-17,1893545	32,83078282	Madeira	Madeira
PM14-07f	PM14	PM14-7	*Lobaria immixta*	12799	-17,1893545	32,83078282	Madeira	Madeira
PM14-09a	PM14	PM14-9	*Lobaria immixta*	12789	-17,18932543	32,83079299	Madeira	Madeira
PM14-09b	PM14	PM14-9	*Lobaria immixta*	12790	-17,18932543	32,83079299	Madeira	Madeira
PM14-09c	PM14	PM14-9	*Lobaria immixta*	12791	-17,18932543	32,83079299	Madeira	Madeira
PM14-09d	PM14	PM14-9	*Lobaria immixta*	12792	-17,18932543	32,83079299	Madeira	Madeira
PM15-01a	PM15	PM15-1	*Lobaria immixta*	12863	-17,157942	32,826744	Madeira	Madeira
PM15-01b	PM15	PM15-1	*Lobaria immixta*	12864	-17,157942	32,826744	Madeira	Madeira
PM15-01c	PM15	PM15-1	*Lobaria immixta*	12865	-17,157942	32,826744	Madeira	Madeira
PM15-11a	PM15	PM15-11	*Lobaria immixta*	12905	-17,15779502	32,82586486	Madeira	Madeira
PM15-11b	PM15	PM15-11	*Lobaria immixta*	12906	-17,15779502	32,82586486	Madeira	Madeira
PM15-11c	PM15	PM15-11	*Lobaria immixta*	12907	-17,15779502	32,82586486	Madeira	Madeira
PM15-11d	PM15	PM15-11	*Lobaria immixta*	12908	-17,15779502	32,82586486	Madeira	Madeira
PM15-12a	PM15	PM15-12	*Lobaria immixta*	12909	-17,15774139	32,82589446	Madeira	Madeira
PM15-12b	PM15	PM15-12	*Lobaria immixta*	12910	-17,15774139	32,82589446	Madeira	Madeira
PM15-12c	PM15	PM15-12	*Lobaria immixta*	12911	-17,15774139	32,82589446	Madeira	Madeira
PM15-13a	PM15	PM15-13	*Lobaria immixta*	12916	-17,15779748	32,82578724	Madeira	Madeira
PM15-13b	PM15	PM15-13	*Lobaria immixta*	12917	-17,15779748	32,82578724	Madeira	Madeira
PM15-13c	PM15	PM15-13	*Lobaria immixta*	12918	-17,15779748	32,82578724	Madeira	Madeira
PM15-16a	PM15	PM15-16	*Lobaria immixta*	12921	-17,1585153	32,82564115	Madeira	Madeira
PM15-16b	PM15	PM15-16	*Lobaria immixta*	12922	-17,1585153	32,82564115	Madeira	Madeira
PM15-16c	PM15	PM15-16	*Lobaria immixta*	12923	-17,1585153	32,82564115	Madeira	Madeira
PM15-17a	PM15	PM15-17	*Lobaria immixta*	12926	-17,15858274	32,82571105	Madeira	Madeira

ID	Pop	PopTree	Species	VoucherID	X	Y	Location	Area
PM15-17b	PM15	PM15-17	*Lobaria immixta*	12927	-17,15858274	32,82571105	Madeira	Madeira
PM15-17c	PM15	PM15-17	*Lobaria immixta*	12928	-17,15858274	32,82571105	Madeira	Madeira
PM15-17d	PM15	PM15-17	*Lobaria immixta*	12929	-17,15858274	32,82571105	Madeira	Madeira
PM15-18a	PM15	PM15-18	*Lobaria immixta*	12933	-17,15856117	32,82575228	Madeira	Madeira
PM15-18b	PM15	PM15-18	*Lobaria immixta*	12934	-17,15856117	32,82575228	Madeira	Madeira
PM15-18c	PM15	PM15-18	*Lobaria immixta*	12935	-17,15856117	32,82575228	Madeira	Madeira
PM15-19a	PM15	PM15-19	*Lobaria immixta*	12937	-17,15855026	32,82530157	Madeira	Madeira
PM15-19b	PM15	PM15-19	*Lobaria immixta*	12938	-17,15855026	32,82530157	Madeira	Madeira
PM15-19c	PM15	PM15-19	*Lobaria immixta*	12939	-17,15855026	32,82530157	Madeira	Madeira
PM15-19d	PM15	PM15-19	*Lobaria immixta*	12940	-17,15855026	32,82530157	Madeira	Madeira
PM15-02a	PM15	PM15-2	*Lobaria immixta*	12869	-17,15788252	32,82657081	Madeira	Madeira
PM15-20a	PM15	PM15-20	*Lobaria immixta*	12948	-17,15853156	32,82529288	Madeira	Madeira
PM15-21a	PM15	PM15-21	*Lobaria immixta*	12952	-17,15853927	32,8253097	Madeira	Madeira
PM15-21b	PM15	PM15-21	*Lobaria immixta*	12953	-17,15853927	32,8253097	Madeira	Madeira
PM15-21c	PM15	PM15-21	*Lobaria immixta*	12954	-17,15853927	32,8253097	Madeira	Madeira
PM15-22a	PM15	PM15-22	*Lobaria immixta*	12960	-17,15852097	32,8253319	Madeira	Madeira
PM15-03a	PM15	PM15-3	*Lobaria immixta*	12871	-17,157777	32,82655697	Madeira	Madeira
PM15-03b	PM15	PM15-3	*Lobaria immixta*	12872	-17,157777	32,82655697	Madeira	Madeira
PM15-30b	PM15	PM15-30	*Lobaria immixta*	12971	-17,15814226	32,82441216	Madeira	Madeira
PM15-30c	PM15	PM15-30	*Lobaria immixta*	12972	-17,15814226	32,82441216	Madeira	Madeira
PM15-04a	PM15	PM15-4	*Lobaria immixta*	12878	-17,15777569	32,82650289	Madeira	Madeira
PM15-04b	PM15	PM15-4	*Lobaria immixta*	12879	-17,15777569	32,82650289	Madeira	Madeira
PM15-05a	PM15	PM15-5	*Lobaria immixta*	12882	-17,1577519	32,82643362	Madeira	Madeira
PM15-05b	PM15	PM15-5	*Lobaria immixta*	12883	-17,1577519	32,82643362	Madeira	Madeira
PM15-06a	PM15	PM15-6	*Lobaria immixta*	12888	-17,15783216	32,82607939	Madeira	Madeira
PM15-06b	PM15	PM15-6	*Lobaria immixta*	12889	-17,15783216	32,82607939	Madeira	Madeira
PM15-06c	PM15	PM15-6	*Lobaria immixta*	12890	-17,15783216	32,82607939	Madeira	Madeira

ID	Pop	PopTree	Species	VoucherID	X	Y	Location	Area
PM15-06d	PM15	PM15-6	*Lobaria immixta*	12891	-17,15783216	32,82607939	Madeira	Madeira
PM15-08a	PM15	PM15-8	*Lobaria immixta*	12898	-17,1578293	32,82597899	Madeira	Madeira
PM15-08b	PM15	PM15-8	*Lobaria immixta*	12899	-17,1578293	32,82597899	Madeira	Madeira
PM15-08c	PM15	PM15-8	*Lobaria immixta*	12900	-17,1578293	32,82597899	Madeira	Madeira
PM16-01a	PM16	PM16-1	*Lobaria immixta*	13034	-17,14085	32,80675	Madeira	Madeira
PM16-01b	PM16	PM16-1	*Lobaria immixta*	13035	-17,14085	32,80675	Madeira	Madeira
PM16-10a	PM16	PM16-10	*Lobaria immixta*	13077	-17,14062971	32,80713525	Madeira	Madeira
PM16-10b	PM16	PM16-10	*Lobaria immixta*	13078	-17,14062971	32,80713525	Madeira	Madeira
PM16-10c	PM16	PM16-10	*Lobaria immixta*	13079	-17,14062971	32,80713525	Madeira	Madeira
PM16-11a	PM16	PM16-11	*Lobaria immixta*	13086	-17,1406093	32,80714058	Madeira	Madeira
PM16-11b	PM16	PM16-11	*Lobaria immixta*	13087	-17,1406093	32,80714058	Madeira	Madeira
PM16-12a	PM16	PM16-12	*Lobaria immixta*	13089	-17,14058125	32,80712753	Madeira	Madeira
PM16-12b	PM16	PM16-12	*Lobaria immixta*	13090	-17,14058125	32,80712753	Madeira	Madeira
PM16-13a	PM16	PM16-13	*Lobaria immixta*	13092	-17,14058053	32,80713653	Madeira	Madeira
PM16-13b	PM16	PM16-13	*Lobaria immixta*	13093	-17,14058053	32,80713653	Madeira	Madeira
PM16-13c	PM16	PM16-13	*Lobaria immixta*	13094	-17,14058053	32,80713653	Madeira	Madeira
PM16-13d	PM16	PM16-13	*Lobaria immixta*	13095	-17,14058053	32,80713653	Madeira	Madeira
PM16-14a	PM16	PM16-14	*Lobaria immixta*	13098	-17,14057899	32,80714084	Madeira	Madeira
PM16-15a	PM16	PM16-15	*Lobaria immixta*	13103	-17,14059819	32,80732044	Madeira	Madeira
PM16-15b	PM16	PM16-15	*Lobaria immixta*	13104	-17,14059819	32,80732044	Madeira	Madeira
PM16-15c	PM16	PM16-15	*Lobaria immixta*	13105	-17,14059819	32,80732044	Madeira	Madeira
PM16-16a	PM16	PM16-16	*Lobaria immixta*	13109	-17,1404083	32,80729549	Madeira	Madeira
PM16-16b	PM16	PM16-16	*Lobaria immixta*	13110	-17,1404083	32,80729549	Madeira	Madeira
PM16-16c	PM16	PM16-16	*Lobaria immixta*	13111	-17,1404083	32,80729549	Madeira	Madeira
PM16-16d	PM16	PM16-16	*Lobaria immixta*	13112	-17,1404083	32,80729549	Madeira	Madeira
PM16-18a	PM16	PM16-18	*Lobaria immixta*	13119	-17,1404382	32,80719959	Madeira	Madeira
PM16-18b	PM16	PM16-18	*Lobaria immixta*	13120	-17,1404382	32,80719959	Madeira	Madeira

ID	Pop	PopTree	Species	VoucherID	X	Y	Location	Area
PM16-19a	PM16	PM16-19	*Lobaria immixta*	13121	-17,14042758	32,80720056	Madeira	Madeira
PM16-19b	PM16	PM16-19	*Lobaria immixta*	13122	-17,14042758	32,80720056	Madeira	Madeira
PM16-19c	PM16	PM16-19	*Lobaria immixta*	13123	-17,14042758	32,80720056	Madeira	Madeira
PM16-20a	PM16	PM16-20	*Lobaria immixta*	13126	-17,14034214	32,80714649	Madeira	Madeira
PM16-20b	PM16	PM16-20	*Lobaria immixta*	13127	-17,14034214	32,80714649	Madeira	Madeira
PM16-20c	PM16	PM16-20	*Lobaria immixta*	13128	-17,14034214	32,80714649	Madeira	Madeira
PM16-20d	PM16	PM16-20	*Lobaria immixta*	13129	-17,14034214	32,80714649	Madeira	Madeira
PM16-21a	PM16	PM16-21	*Lobaria immixta*	13132	-17,14025594	32,8071997	Madeira	Madeira
PM16-21b	PM16	PM16-21	*Lobaria immixta*	13133	-17,14025594	32,8071997	Madeira	Madeira
PM16-21c	PM16	PM16-21	*Lobaria immixta*	13134	-17,14025594	32,8071997	Madeira	Madeira
PM16-21d	PM16	PM16-21	*Lobaria immixta*	13135	-17,14025594	32,8071997	Madeira	Madeira
PM16-22a	PM16	PM16-22	*Lobaria immixta*	13137	-17,14027532	32,80720727	Madeira	Madeira
PM16-22b	PM16	PM16-22	*Lobaria immixta*	13138	-17,14027532	32,80720727	Madeira	Madeira
PM16-22c	PM16	PM16-22	*Lobaria immixta*	13139	-17,14027532	32,80720727	Madeira	Madeira
PM16-03a	PM16	PM16-3	*Lobaria immixta*	13041	-17,1408655	32,8068712	Madeira	Madeira
PM16-03b	PM16	PM16-3	*Lobaria immixta*	13042	-17,1408655	32,8068712	Madeira	Madeira
PM16-03c	PM16	PM16-3	*Lobaria immixta*	13043	-17,1408655	32,8068712	Madeira	Madeira
PM16-03d	PM16	PM16-3	*Lobaria immixta*	13044	-17,1408655	32,8068712	Madeira	Madeira
PM16-04a	PM16	PM16-4	*Lobaria immixta*	13045	-17,14079231	32,80695999	Madeira	Madeira
PM16-05a	PM16	PM16-5	*Lobaria immixta*	13053	-17,14080872	32,80690771	Madeira	Madeira
PM16-05b	PM16	PM16-5	*Lobaria immixta*	13054	-17,14080872	32,80690771	Madeira	Madeira
PM16-07a	PM16	PM16-7	*Lobaria immixta*	13061	-17,14062748	32,80701111	Madeira	Madeira
PM16-07b	PM16	PM16-7	*Lobaria immixta*	13062	-17,14062748	32,80701111	Madeira	Madeira
PM16-07c	PM16	PM16-7	*Lobaria immixta*	13063	-17,14062748	32,80701111	Madeira	Madeira
PM16-07d	PM16	PM16-7	*Lobaria immixta*	13064	-17,14062748	32,80701111	Madeira	Madeira
PM16-07e	PM16	PM16-7	*Lobaria immixta*	13065	-17,14062748	32,80701111	Madeira	Madeira
PM16-08a	PM16	PM16-8	*Lobaria immixta*	13068	-17,14061861	32,80700609	Madeira	Madeira

ID	Pop	PopTree	Species	VoucherID	X	Y	Location	Area
PM16-08b	PM16	PM16-8	*Lobaria immixta*	13069	-17,14061861	32,80700609	Madeira	Madeira
PM16-08c	PM16	PM16-8	*Lobaria immixta*	13070	-17,14061861	32,80700609	Madeira	Madeira
PM16-08d	PM16	PM16-8	*Lobaria immixta*	13071	-17,14061861	32,80700609	Madeira	Madeira
PM16-09a	PM16	PM16-9	*Lobaria immixta*	13074	-17,14063857	32,80714028	Madeira	Madeira
PM16-09b	PM16	PM16-9	*Lobaria immixta*	13075	-17,14063857	32,80714028	Madeira	Madeira
PM16-09c	PM16	PM16-9	*Lobaria immixta*	13076	-17,14063857	32,80714028	Madeira	Madeira
PM17-01a	PM17	PM17-1	*Lobaria immixta*	13142	-16,830673	32,74492	Madeira	Madeira
PM17-01b	PM17	PM17-1	*Lobaria immixta*	13143	-16,830673	32,74492	Madeira	Madeira
PM17-01c	PM17	PM17-1	*Lobaria immixta*	13144	-16,830673	32,74492	Madeira	Madeira
PM17-10a	PM17	PM17-10	*Lobaria immixta*	13163	-16,83210544	32,74458861	Madeira	Madeira
PM17-11a	PM17	PM17-11	*Lobaria immixta*	13164	-16,83215753	32,74471651	Madeira	Madeira
PM17-11b	PM17	PM17-11	*Lobaria immixta*	13165	-16,83215753	32,74471651	Madeira	Madeira
PM17-11c	PM17	PM17-11	*Lobaria immixta*	13166	-16,83215753	32,74471651	Madeira	Madeira
PM17-12a	PM17	PM17-12	*Lobaria immixta*	13168	-16,83215236	32,74473401	Madeira	Madeira
PM17-12b	PM17	PM17-12	*Lobaria immixta*	13169	-16,83215236	32,74473401	Madeira	Madeira
PM17-14a	PM17	PM17-14	*Lobaria immixta*	13174	-16,83222912	32,74488639	Madeira	Madeira
PM17-14b	PM17	PM17-14	*Lobaria immixta*	13175	-16,83222912	32,74488639	Madeira	Madeira
PM17-14c	PM17	PM17-14	*Lobaria immixta*	13176	-16,83222912	32,74488639	Madeira	Madeira
PM17-15a	PM17	PM17-15	*Lobaria immixta*	13178	-16,83227811	32,74493404	Madeira	Madeira
PM17-15b	PM17	PM17-15	*Lobaria immixta*	13179	-16,83227811	32,74493404	Madeira	Madeira
PM17-19a	PM17	PM17-19	*Lobaria immixta*	13188	-16,8326191	32,74504703	Madeira	Madeira
PM17-02a	PM17	PM17-2	*Lobaria immixta*	13146	-16,83068698	32,74478525	Madeira	Madeira
PM17-02b	PM17	PM17-2	*Lobaria immixta*	13147	-16,83068698	32,74478525	Madeira	Madeira
PM17-03a	PM17	PM17-3	*Lobaria immixta*	13148	-16,83074436	32,74465899	Madeira	Madeira
PM17-04a	PM17	PM17-4	*Lobaria immixta*	13149	-16,83179182	32,74448707	Madeira	Madeira
PM17-05a	PM17	PM17-5	*Lobaria immixta*	13150	-16,83181265	32,74453822	Madeira	Madeira
PM17-06a	PM17	PM17-6	*Lobaria immixta*	13151	-16,8318848	32,7445219	Madeira	Madeira

ID	Pop	PopTree	Species	VoucherID	X	Y	Location	Area
PM17-07a	PM17	PM17-7	*Lobaria immixta*	13153	-16,83196859	32,74453567	Madeira	Madeira
PM17-08a	PM17	PM17-8	*Lobaria immixta*	13154	-16,83213442	32,74464919	Madeira	Madeira
PM17-08b	PM17	PM17-8	*Lobaria immixta*	13155	-16,83213442	32,74464919	Madeira	Madeira
PM17-08c	PM17	PM17-8	*Lobaria immixta*	13156	-16,83213442	32,74464919	Madeira	Madeira
PM17-08d	PM17	PM17-8	*Lobaria immixta*	13157	-16,83213442	32,74464919	Madeira	Madeira
PM17-08e	PM17	PM17-8	*Lobaria immixta*	13158	-16,83213442	32,74464919	Madeira	Madeira
PM17-09a	PM17	PM17-9	*Lobaria immixta*	13161	-16,83211324	32,74465139	Madeira	Madeira
PM9-10a	PM9	PM9-10	*Lobaria immixta*	12341	-16,88518338	32,73799197	Madeira	Madeira
PM9-10b	PM9	PM9-10	*Lobaria immixta*	12342	-16,88518338	32,73799197	Madeira	Madeira
PM9-10c	PM9	PM9-10	*Lobaria immixta*	12343	-16,88518338	32,73799197	Madeira	Madeira
PM9-13c	PM9	PM9-13	*Lobaria immixta*	12353	-16,88534339	32,73798957	Madeira	Madeira
PM9-13d	PM9	PM9-13	*Lobaria immixta*	12354	-16,88534339	32,73798957	Madeira	Madeira
PM9-13e	PM9	PM9-13	*Lobaria immixta*	12355	-16,88534339	32,73798957	Madeira	Madeira
PM9-15a	PM9	PM9-15	*Lobaria immixta*	12359	-16,88555675	32,73798636	Madeira	Madeira
PM9-15b	PM9	PM9-15	*Lobaria immixta*	12360	-16,88555675	32,73798636	Madeira	Madeira
PM9-15c	PM9	PM9-15	*Lobaria immixta*	12361	-16,88555675	32,73798636	Madeira	Madeira
PM9-15d	PM9	PM9-15	*Lobaria immixta*	12362	-16,88555675	32,73798636	Madeira	Madeira
PM9-16h	PM9	PM9-16	*Lobaria immixta*	12372	-16,88561008	32,73798555	Madeira	Madeira
PM9-17d	PM9	PM9-17	*Lobaria immixta*	12376	-16,88566342	32,73798475	Madeira	Madeira
PM9-17e	PM9	PM9-17	*Lobaria immixta*	12377	-16,88566342	32,73798475	Madeira	Madeira
PM9-17f	PM9	PM9-17	*Lobaria immixta*	12378	-16,88566342	32,73798475	Madeira	Madeira
PM9-17g	PM9	PM9-17	*Lobaria immixta*	12379	-16,88566342	32,73798475	Madeira	Madeira
PM9-18e	PM9	PM9-18	*Lobaria immixta*	12384	-16,88571676	32,73798395	Madeira	Madeira
PM9-18f	PM9	PM9-18	*Lobaria immixta*	12385	-16,88571676	32,73798395	Madeira	Madeira
PM9-18g	PM9	PM9-18	*Lobaria immixta*	12386	-16,88571676	32,73798395	Madeira	Madeira
PM9-02a	PM9	PM9-2	*Lobaria immixta*	12298	-16,88470334	32,7379992	Madeira	Madeira
PM9-02b	PM9	PM9-2	*Lobaria immixta*	12299	-16,88470334	32,7379992	Madeira	Madeira

ID	Pop	PopTree	Species	VoucherID	X	Y	Location	Area
PM9-02c	PM9	PM9-2	*Lobaria immixta*	12300	-16,88470334	32,7379992	Madeira	Madeira
PM9-02d	PM9	PM9-2	*Lobaria immixta*	12301	-16,88470334	32,7379992	Madeira	Madeira
PM9-20d	PM9	PM9-20	*Lobaria immixta*	12391	-16,88577543	32,73798307	Madeira	Madeira
PM9-20e	PM9	PM9-20	*Lobaria immixta*	12392	-16,88577543	32,73798307	Madeira	Madeira
PM9-20f	PM9	PM9-20	*Lobaria immixta*	12393	-16,88577543	32,73798307	Madeira	Madeira
PM9-21a	PM9	PM9-21	*Lobaria immixta*	12394	-16,88582344	32,73798234	Madeira	Madeira
PM9-21b	PM9	PM9-21	*Lobaria immixta*	12395	-16,88582344	32,73798234	Madeira	Madeira
PM9-21c	PM9	PM9-21	*Lobaria immixta*	12396	-16,88582344	32,73798234	Madeira	Madeira
PM9-21d	PM9	PM9-21	*Lobaria immixta*	12397	-16,88582344	32,73798234	Madeira	Madeira
PM9-21e	PM9	PM9-21	*Lobaria immixta*	12398	-16,88582344	32,73798234	Madeira	Madeira
PM9-03a	PM9	PM9-3	*Lobaria immixta*	12306	-16,88475668	32,73799839	Madeira	Madeira
PM9-03b	PM9	PM9-3	*Lobaria immixta*	12307	-16,88475668	32,73799839	Madeira	Madeira
PM9-03c	PM9	PM9-3	*Lobaria immixta*	12308	-16,88475668	32,73799839	Madeira	Madeira
PM9-04a	PM9	PM9-4	*Lobaria immixta*	12313	-16,88481001	32,73799759	Madeira	Madeira
PM9-04b	PM9	PM9-4	*Lobaria immixta*	12314	-16,88481001	32,73799759	Madeira	Madeira
PM9-04c	PM9	PM9-4	*Lobaria immixta*	12315	-16,88481001	32,73799759	Madeira	Madeira
PM9-06d	PM9	PM9-6	*Lobaria immixta*	12327	-16,88486335	32,73799679	Madeira	Madeira
PM9-06e	PM9	PM9-6	*Lobaria immixta*	12328	-16,88486335	32,73799679	Madeira	Madeira
PM9-07a	PM9	PM9-7	*Lobaria immixta*	12329	-16,88491669	32,73799599	Madeira	Madeira
PM9-07d	PM9	PM9-7	*Lobaria immixta*	12332	-16,88491669	32,73799599	Madeira	Madeira
PM9-08b	PM9	PM9-8	*Lobaria immixta*	12334	-16,8850767	32,73799358	Madeira	Madeira
PM9-09d	PM9	PM9-9	*Lobaria immixta*	12339	-16,88513004	32,73799278	Madeira	Madeira
PM9-09e	PM9	PM9-9	*Lobaria immixta*	12340	-16,88513004	32,73799278	Madeira	Madeira
PS2-04a	PS	PS2-4	*Lobaria immixta*	31744	-9,390095	38,787106	Sintra	Ib. Peninsula
PS2-05b	PS	PS2-5	*Lobaria immixta*	31747	-9,390095	38,787106	Sintra	Ib. Peninsula
PS2-05c	PS	PS2-5	*Lobaria immixta*	31748	-9,390095	38,787106	Sintra	Ib. Peninsula
PS2-05d	PS	PS2-5	*Lobaria immixta*	31749	-9,390095	38,787106	Sintra	Ib. Peninsula

ID	Pop	PopTree	Species	VoucherID	X	Y	Location	Area
PS2-05e	PS	PS2-5	*Lobaria immixta*	31750	-9,390095	38,787106	Sintra	Ib. Peninsula
SG1-01a	SG1	SG1-1	*Lobaria immixta*	11936	-17,29628	28,15027	La Gomera	Canary Isl.
SG1-10b	SG1	SG1-10	*Lobaria immixta*	11964	-17,29620425	28,15052903	La Gomera	Canary Isl.
SG1-11c	SG1	SG1-11	*Lobaria immixta*	11968	-17,29630564	28,15052103	La Gomera	Canary Isl.
SG1-11d	SG1	SG1-11	*Lobaria immixta*	11969	-17,29630564	28,15052103	La Gomera	Canary Isl.
SG1-12a	SG1	SG1-12	*Lobaria immixta*	11971	-17,29629077	28,15053336	La Gomera	Canary Isl.
SG1-12b	SG1	SG1-12	*Lobaria immixta*	11972	-17,29629077	28,15053336	La Gomera	Canary Isl.
SG1-13a	SG1	SG1-13	*Lobaria immixta*	11973	-17,29630176	28,15055861	La Gomera	Canary Isl.
SG1-13b	SG1	SG1-13	*Lobaria immixta*	11974	-17,29630176	28,15055861	La Gomera	Canary Isl.
SG1-13c	SG1	SG1-13	*Lobaria immixta*	11975	-17,29630176	28,15055861	La Gomera	Canary Isl.
SG1-13d	SG1	SG1-13	*Lobaria immixta*	11976	-17,29630176	28,15055861	La Gomera	Canary Isl.
SG1-13e	SG1	SG1-13	*Lobaria immixta*	11977	-17,29630176	28,15055861	La Gomera	Canary Isl.
SG1-14a	SG1	SG1-14	*Lobaria immixta*	11980	-17,29634349	28,15058443	La Gomera	Canary Isl.
SG1-14b	SG1	SG1-14	*Lobaria immixta*	11981	-17,29634349	28,15058443	La Gomera	Canary Isl.
SG1-14c	SG1	SG1-14	*Lobaria immixta*	11982	-17,29634349	28,15058443	La Gomera	Canary Isl.
SG1-14d	SG1	SG1-14	*Lobaria immixta*	11983	-17,29634349	28,15058443	La Gomera	Canary Isl.
SG1-14e	SG1	SG1-14	*Lobaria immixta*	11984	-17,29634349	28,15058443	La Gomera	Canary Isl.
SG1-15a	SG1	SG1-15	*Lobaria immixta*	11988	-17,29631375	28,15060908	La Gomera	Canary Isl.
SG1-15b	SG1	SG1-15	*Lobaria immixta*	11989	-17,29631375	28,15060908	La Gomera	Canary Isl.
SG1-15c	SG1	SG1-15	*Lobaria immixta*	11990	-17,29631375	28,15060908	La Gomera	Canary Isl.
SG1-16c	SG1	SG1-16	*Lobaria immixta*	11993	-17,29631433	28,15063614	La Gomera	Canary Isl.
SG1-17a	SG1	SG1-17	*Lobaria immixta*	11995	-17,2962848	28,15068352	La Gomera	Canary Isl.
SG1-17b	SG1	SG1-17	*Lobaria immixta*	11996	-17,2962848	28,15068352	La Gomera	Canary Isl.
SG1-18a	SG1	SG1-18	*Lobaria immixta*	12000	-17,29621095	28,15065311	La Gomera	Canary Isl.
SG1-18b	SG1	SG1-18	*Lobaria immixta*	12001	-17,29621095	28,15065311	La Gomera	Canary Isl.
SG1-02a	SG1	SG1-2	*Lobaria immixta*	11940	-17,29628349	28,15023405	La Gomera	Canary Isl.
SG1-02b	SG1	SG1-2	*Lobaria immixta*	11941	-17,29628349	28,15023405	La Gomera	Canary Isl.

ID	Pop	PopTree	Species	VoucherID	X	Y	Location	Area
SG1-02c	SG1	SG1-2	*Lobaria immixta*	11942	-17,29628349	28,15023405	La Gomera	Canary Isl.
SG1-20	SG1	SG1-20	*Lobaria immixta*	12005	-17,29587104	28,15020504	La Gomera	Canary Isl.
SG1-22	SG1	SG1-22	*Lobaria immixta*	12008	-17,29565685	28,15012744	La Gomera	Canary Isl.
SG1-03a	SG1	SG1-3	*Lobaria immixta*	**11945**	-17,29620358	28,15024792	La Gomera	Canary Isl.
SG1-03a1	SG1	SG1-3	*Lobaria immixta*	**11945**	-17,29620358	28,15024792	La Gomera	Canary Isl.
SG1-03b	SG1	SG1-3	*Lobaria immixta*	11946	-17,29620358	28,15024792	La Gomera	Canary Isl.
SG1-04a	SG1	SG1-4	*Lobaria immixta*	11948	-17,29620749	28,15026563	La Gomera	Canary Isl.
SG1-07a	SG1	SG1-7	*Lobaria immixta*	11956	-17,2962525	28,15033118	La Gomera	Canary Isl.
SG3-01a	SG3	SG3-1	*Lobaria immixta*	10462	-17,25633	28,13098	La Gomera	Canary Isl.
SG3-01b	SG3	SG3-1	*Lobaria immixta*	10463	-17,25633	28,13098	La Gomera	Canary Isl.
SG3-01c	SG3	SG3-1	*Lobaria immixta*	10464	-17,25633	28,13098	La Gomera	Canary Isl.
SG3-10	SG3	SG3-10	*Lobaria immixta*	10487	-17,25642916	28,13147466	La Gomera	Canary Isl.
SG3-11a	SG3	SG3-11	*Lobaria immixta*	10488	-17,25637963	28,13160266	La Gomera	Canary Isl.
SG3-11b	SG3	SG3-11	*Lobaria immixta*	10489	-17,25637963	28,13160266	La Gomera	Canary Isl.
SG3-12a	SG3	SG3-12	*Lobaria immixta*	10490	-17,25633039	28,13168161	La Gomera	Canary Isl.
SG3-12b	SG3	SG3-12	*Lobaria immixta*	10491	-17,25633039	28,13168161	La Gomera	Canary Isl.
SG3-12c	SG3	SG3-12	*Lobaria immixta*	10492	-17,25633039	28,13168161	La Gomera	Canary Isl.
SG3-13a	SG3	SG3-13	*Lobaria immixta*	10493	-17,25645306	28,1318256	La Gomera	Canary Isl.
SG3-13b	SG3	SG3-13	*Lobaria immixta*	10494	-17,25645306	28,1318256	La Gomera	Canary Isl.
SG3-14a	SG3	SG3-14	*Lobaria immixta*	10497	-17,25646975	28,13183593	La Gomera	Canary Isl.
SG3-14b	SG3	SG3-14	*Lobaria immixta*	10498	-17,25646975	28,13183593	La Gomera	Canary Isl.
SG3-14e	SG3	SG3-14	*Lobaria immixta*	10501	-17,25646975	28,13183593	La Gomera	Canary Isl.
SG3-15a	SG3	SG3-15	*Lobaria immixta*	10503	-17,25652323	28,13189035	La Gomera	Canary Isl.
SG3-16c	SG3	SG3-16	*Lobaria immixta*	10508	-17,25653157	28,13189552	La Gomera	Canary Isl.
SG3-16d	SG3	SG3-16	*Lobaria immixta*	10509	-17,25653157	28,13189552	La Gomera	Canary Isl.
SG3-16e	SG3	SG3-16	*Lobaria immixta*	10510	-17,25653157	28,13189552	La Gomera	Canary Isl.
SG3-17a	SG3	SG3-17	*Lobaria immixta*	10511	-17,25658408	28,13197279	La Gomera	Canary Isl.

ID	Pop	PopTree	Species	VoucherID	X	Y	Location	Area
SG3-19a	SG3	SG3-19	*Lobaria immixta*	10514	-17,25662357	28,13205498	La Gomera	Canary Isl.
SG3-02a	SG3	SG3-2	*Lobaria immixta*	10466	-17,25633973	28,13098263	La Gomera	Canary Isl.
SG3-02b	SG3	SG3-2	*Lobaria immixta*	10467	-17,25633973	28,13098263	La Gomera	Canary Isl.
SG3-21c	SG3	SG3-21	*Lobaria immixta*	10524	-17,25692687	28,13197931	La Gomera	Canary Isl.
SG3-23a	SG3	SG3-23	*Lobaria immixta*	10530	-17,25696468	28,13196837	La Gomera	Canary Isl.
SG3-26a	SG3	SG3-26	*Lobaria immixta*	10541	-17,25706458	28,13200226	La Gomera	Canary Isl.
SG3-26b	SG3	SG3-26	*Lobaria immixta*	10542	-17,25706458	28,13200226	La Gomera	Canary Isl.
SG3-26c	SG3	SG3-26	*Lobaria immixta*	10543	-17,25706458	28,13200226	La Gomera	Canary Isl.
SG3-26d	SG3	SG3-26	*Lobaria immixta*	10544	-17,25706458	28,13200226	La Gomera	Canary Isl.
SG3-27a	SG3	SG3-27	*Lobaria immixta*	10547	-17,25697085	28,1319671	La Gomera	Canary Isl.
SG3-27b	SG3	SG3-27	*Lobaria immixta*	10548	-17,25697085	28,1319671	La Gomera	Canary Isl.
SG3-05a	SG3	SG3-5	*Lobaria immixta*	10473	-17,2562981	28,13101892	La Gomera	Canary Isl.
SG3-05c	SG3	SG3-5	*Lobaria immixta*	10475	-17,2562981	28,13101892	La Gomera	Canary Isl.
SG3-05d	SG3	SG3-5	*Lobaria immixta*	10476	-17,2562981	28,13101892	La Gomera	Canary Isl.
SG3-06c	SG3	SG3-6	*Lobaria immixta*	10479	-17,25631763	28,13110747	La Gomera	Canary Isl.
SG3-07a	SG3	SG3-7	*Lobaria immixta*	10480	-17,2563732	28,13120387	La Gomera	Canary Isl.
SG3-07b	SG3	SG3-7	*Lobaria immixta*	10481	-17,2563732	28,13120387	La Gomera	Canary Isl.
SG4-01a	SG4	SG4-1	*Lobaria immixta*	12015	-17,21512	28,12216	La Gomera	Canary Isl.
SG4-10	SG4	SG4-10	*Lobaria immixta*	12049	-17,21534073	28,12183246	La Gomera	Canary Isl.
SG4-11	SG4	SG4-11	*Lobaria immixta*	12050	-17,21536537	28,12179299	La Gomera	Canary Isl.
SG4-15a	SG4	SG4-15	*Lobaria immixta*	12062	-17,21549443	28,121569	La Gomera	Canary Isl.
SG4-15b	SG4	SG4-15	*Lobaria immixta*	12063	-17,21549443	28,121569	La Gomera	Canary Isl.
SG4-15c	SG4	SG4-15	*Lobaria immixta*	12064	-17,21549443	28,121569	La Gomera	Canary Isl.
SG4-15d	SG4	SG4-15	*Lobaria immixta*	12065	-17,21549443	28,121569	La Gomera	Canary Isl.
SG4-18b	SG4	SG4-18	*Lobaria immixta*	12072	-17,21557932	28,12140559	La Gomera	Canary Isl.
SG4-02a	SG4	SG4-2	*Lobaria immixta*	12018	-17,21512684	28,12213362	La Gomera	Canary Isl.
SG4-02b	SG4	SG4-2	*Lobaria immixta*	12019	-17,21512684	28,12213362	La Gomera	Canary Isl.

ID	Pop	PopTree	Species	VoucherID	X	Y	Location	Area
SG4-02c	SG4	SG4-2	*Lobaria immixta*	12020	-17,21512684	28,12213362	La Gomera	Canary Isl.
SG4-23a	SG4	SG4-23	*Lobaria immixta*	12086	-17,21575453	28,12097994	La Gomera	Canary Isl.
SG4-24c	SG4	SG4-24	*Lobaria immixta*	12094	-17,21585369	28,12072396	La Gomera	Canary Isl.
SG4-03a	SG4	SG4-3	*Lobaria immixta*	12022	-17,21515148	28,12209415	La Gomera	Canary Isl.
SG4-04a	SG4	SG4-4	*Lobaria immixta*	12026	-17,21515809	28,12207709	La Gomera	Canary Isl.
SG4-05a	SG4	SG4-5	*Lobaria immixta*	12028	-17,21517779	28,12204551	La Gomera	Canary Isl.
SG4-05b	SG4	SG4-5	*Lobaria immixta*	12029	-17,21517779	28,12204551	La Gomera	Canary Isl.
SG4-05c	SG4	SG4-5	*Lobaria immixta*	12030	-17,21517779	28,12204551	La Gomera	Canary Isl.
SG4-05d	SG4	SG4-5	*Lobaria immixta*	12031	-17,21517779	28,12204551	La Gomera	Canary Isl.
SG4-06a	SG4	SG4-6	*Lobaria immixta*	12033	-17,21522258	28,1219964	La Gomera	Canary Isl.
SG4-07a	SG4	SG4-7	*Lobaria immixta*	12037	-17,21522919	28,12197934	La Gomera	Canary Isl.
SG4-07b	SG4	SG4-7	*Lobaria immixta*	12038	-17,21522919	28,12197934	La Gomera	Canary Isl.
SG4-07c	SG4	SG4-7	*Lobaria immixta*	12039	-17,21522919	28,12197934	La Gomera	Canary Isl.
SG4-07d	SG4	SG4-7	*Lobaria immixta*	12040	-17,21522919	28,12197934	La Gomera	Canary Isl.
SG4-07e	SG4	SG4-7	*Lobaria immixta*	12041	-17,21522919	28,12197934	La Gomera	Canary Isl.
SG4-08a	SG4	SG4-8	*Lobaria immixta*	12042	-17,21526386	28,12194632	La Gomera	Canary Isl.
SG4-09a	SG4	SG4-9	*Lobaria immixta*	12045	-17,21530355	28,12186325	La Gomera	Canary Isl.
SG5-01b	SG5	SG5-1	*Lobaria immixta*	10554	-17,25633	28,13098	La Gomera	Canary Isl.
SG5-01c	SG5	SG5-1	*Lobaria immixta*	10555	-17,25633	28,13098	La Gomera	Canary Isl.
SG5-12a	SG5	SG5-12	*Lobaria immixta*	10598	-17,25655227	28,13067212	La Gomera	Canary Isl.
SG5-15a	SG5	SG5-15	*Lobaria immixta*	10610	-17,25671684	28,13063855	La Gomera	Canary Isl.
SG5-15b	SG5	SG5-15	*Lobaria immixta*	10611	-17,25671684	28,13063855	La Gomera	Canary Isl.
SG5-15d	SG5	SG5-15	*Lobaria immixta*	10613	-17,25671684	28,13063855	La Gomera	Canary Isl.
SG5-16a	SG5	SG5-16	*Lobaria immixta*	10617	-17,25666935	28,13065476	La Gomera	Canary Isl.
SG5-17b	SG5	SG5-17	*Lobaria immixta*	10624	-17,25653576	28,13058928	La Gomera	Canary Isl.
SG5-18a	SG5	SG5-18	*Lobaria immixta*	10627	-17,25652	28,1305661	La Gomera	Canary Isl.
SG5-18b	SG5	SG5-18	*Lobaria immixta*	10628	-17,25652	28,1305661	La Gomera	Canary Isl.

**ID**	**Pop**	**PopTree**	**Species**	**VoucherID**	**X**	**Y**	**Location**	**Area**
SG5-18c	SG5	SG5-18	*Lobaria immixta*	10629	-17,25652	28,1305661	La Gomera	Canary Isl.
SG5-19a	SG5	SG5-19	*Lobaria immixta*	10632	-17,25651508	28,130574	La Gomera	Canary Isl.
SG5-19b	SG5	SG5-19	*Lobaria immixta*	10633	-17,25651508	28,130574	La Gomera	Canary Isl.
SG5-02b	SG5	SG5-2	*Lobaria immixta*	10561	-17,25632723	28,13097886	La Gomera	Canary Isl.
SG5-20a	SG5	SG5-20	*Lobaria immixta*	10635	-17,25661561	28,13058801	La Gomera	Canary Isl.
SG5-20b	SG5	SG5-20	*Lobaria immixta*	10636	-17,25661561	28,13058801	La Gomera	Canary Isl.
SG5-21b	SG5	SG5-21	*Lobaria immixta*	10641	-17,25658219	28,13055401	La Gomera	Canary Isl.
SG5-22a	SG5	SG5-22	*Lobaria immixta*	10644	-17,25650303	28,13049731	La Gomera	Canary Isl.
SG5-22b	SG5	SG5-22	*Lobaria immixta*	10645	-17,25650303	28,13049731	La Gomera	Canary Isl.
SG5-22c	SG5	SG5-22	*Lobaria immixta*	10646	-17,25650303	28,13049731	La Gomera	Canary Isl.
SG5-22d	SG5	SG5-22	*Lobaria immixta*	10647	-17,25650303	28,13049731	La Gomera	Canary Isl.
SG5-23	SG5	SG5-23	*Lobaria immixta*	10651	-17,25642387	28,13044061	La Gomera	Canary Isl.
SG5-24a	SG5	SG5-24	*Lobaria immixta*	10652	-17,25629027	28,13037514	La Gomera	Canary Isl.
SG5-24b	SG5	SG5-24	*Lobaria immixta*	10653	-17,25629027	28,13037514	La Gomera	Canary Isl.
SG5-04a	SG5	SG5-4	*Lobaria immixta*	10567	-17,25636922	28,13094981	La Gomera	Canary Isl.
SG5-05a	SG5	SG5-5	*Lobaria immixta*	10572	-17,25636282	28,13095682	La Gomera	Canary Isl.
SH1-01	SH1	SH1-1	*Lobaria immixta*	10659	-17,98081999	27,76102	El Hierro	Canary Isl.
SH1-10a	SH1	SH1-10	*Lobaria immixta*	10690	-17,98020643	27,76137287	El Hierro	Canary Isl.
SH1-11a	SH1	SH1-11	*Lobaria immixta*	10692	-17,98015481	27,76145048	El Hierro	Canary Isl.
SH1-13a	SH1	SH1-13	*Lobaria immixta*	10700	-17,98009082	27,76144346	El Hierro	Canary Isl.
SH1-13b	SH1	SH1-13	*Lobaria immixta*	10701	-17,98009082	27,76144346	El Hierro	Canary Isl.
SH1-13c	SH1	SH1-13	*Lobaria immixta*	10702	-17,98009082	27,76144346	El Hierro	Canary Isl.
SH1-14a	SH1	SH1-14	*Lobaria immixta*	10704	-17,98004861	27,7614185	El Hierro	Canary Isl.
SH1-14b	SH1	SH1-14	*Lobaria immixta*	10705	-17,98004861	27,7614185	El Hierro	Canary Isl.
SH1-14c	SH1	SH1-14	*Lobaria immixta*	10706	-17,98004861	27,7614185	El Hierro	Canary Isl.
SH1-14e	SH1	SH1-14	*Lobaria immixta*	10708	-17,98004861	27,7614185	El Hierro	Canary Isl.
SH1-15a	SH1	SH1-15	*Lobaria immixta*	10711	-17,97985882	27,7616299	El Hierro	Canary Isl.

ID	Pop	PopTree	Species	VoucherID	X	Y	Location	Area
SH1-16a	SH1	SH1-16	*Lobaria immixta*	10713	-17,9796772	27,76171006	El Hierro	Canary Isl.
SH1-16b	SH1	SH1-16	*Lobaria immixta*	10714	-17,9796772	27,76171006	El Hierro	Canary Isl.
SH1-16c	SH1	SH1-16	*Lobaria immixta*	10715	-17,9796772	27,76171006	El Hierro	Canary Isl.
SH1-17a	SH1	SH1-17	*Lobaria immixta*	10718	-17,97944402	27,7613097	El Hierro	Canary Isl.
SH1-17b	SH1	SH1-17	*Lobaria immixta*	10719	-17,97944402	27,7613097	El Hierro	Canary Isl.
SH1-17c	SH1	SH1-17	*Lobaria immixta*	10720	-17,97944402	27,7613097	El Hierro	Canary Isl.
SH1-17d	SH1	SH1-17	*Lobaria immixta*	10721	-17,97944402	27,7613097	El Hierro	Canary Isl.
SH1-18a	SH1	SH1-18	*Lobaria immixta*	10722	-17,97945066	27,7612833	El Hierro	Canary Isl.
SH1-18b	SH1	SH1-18	*Lobaria immixta*	10723	-17,97945066	27,7612833	El Hierro	Canary Isl.
SH1-18e	SH1	SH1-18	*Lobaria immixta*	10726	-17,97945066	27,7612833	El Hierro	Canary Isl.
SH1-19a	SH1	SH1-19	*Lobaria immixta*	10727	-17,97948101	27,76128501	El Hierro	Canary Isl.
SH1-19b	SH1	SH1-19	*Lobaria immixta*	10728	-17,97948101	27,76128501	El Hierro	Canary Isl.
SH1-19c	SH1	SH1-19	*Lobaria immixta*	10729	-17,97948101	27,76128501	El Hierro	Canary Isl.
SH1-19f	SH1	SH1-19	*Lobaria immixta*	10732	-17,97948101	27,76128501	El Hierro	Canary Isl.
SH1-02d	SH1	SH1-2	*Lobaria immixta*	10663	-17,98084152	27,76108956	El Hierro	Canary Isl.
SH1-02e	SH1	SH1-2	*Lobaria immixta*	10664	-17,98084152	27,76108956	El Hierro	Canary Isl.
SH1-02f	SH1	SH1-2	*Lobaria immixta*	10665	-17,98084152	27,76108956	El Hierro	Canary Isl.
SH1-02g	SH1	SH1-2	*Lobaria immixta*	10666	-17,98084152	27,76108956	El Hierro	Canary Isl.
SH1-21a	SH1	SH1-21	*Lobaria immixta*	10736	-17,9796004	27,76107082	El Hierro	Canary Isl.
SH1-21b	SH1	SH1-21	*Lobaria immixta*	10737	-17,9796004	27,76107082	El Hierro	Canary Isl.
SH1-21c	SH1	SH1-21	*Lobaria immixta*	10738	-17,9796004	27,76107082	El Hierro	Canary Isl.
SH1-22a	SH1	SH1-22	*Lobaria immixta*	10743	-17,97966054	27,76107892	El Hierro	Canary Isl.
SH1-22b	SH1	SH1-22	*Lobaria immixta*	10744	-17,97966054	27,76107892	El Hierro	Canary Isl.
SH1-03a	SH1	SH1-3	*Lobaria immixta*	10668	-17,98080091	27,76117219	El Hierro	Canary Isl.
SH1-03b	SH1	SH1-3	*Lobaria immixta*	10669	-17,98080091	27,76117219	El Hierro	Canary Isl.
SH1-03c	SH1	SH1-3	*Lobaria immixta*	10670	-17,98080091	27,76117219	El Hierro	Canary Isl.
SH1-04b	SH1	SH1-4	*Lobaria immixta*	10672	-17,98077076	27,76117567	El Hierro	Canary Isl.

**ID**	**Pop**	**PopTree**	**Species**	**VoucherID**	**X**	**Y**	**Location**	**Area**
SH1-05a	SH1	SH1-5	*Lobaria immixta*	10673	-17,98072007	27,76117518	El Hierro	Canary Isl.
SH1-05b	SH1	SH1-5	*Lobaria immixta*	10674	-17,98072007	27,76117518	El Hierro	Canary Isl.
SH1-05c	SH1	SH1-5	*Lobaria immixta*	10675	-17,98072007	27,76117518	El Hierro	Canary Isl.
SH1-06b	SH1	SH1-6	*Lobaria immixta*	10677	-17,98067947	27,76125781	El Hierro	Canary Isl.
SH1-07a	SH1	SH1-7	*Lobaria immixta*	10678	-17,98049068	27,7613236	El Hierro	Canary Isl.
SH1-07b	SH1	SH1-7	*Lobaria immixta*	10679	-17,98049068	27,7613236	El Hierro	Canary Isl.
SH1-07c	SH1	SH1-7	*Lobaria immixta*	10680	-17,98049068	27,7613236	El Hierro	Canary Isl.
SH1-09a	SH1	SH1-9	*Lobaria immixta*	10686	-17,98037572	27,76127361	El Hierro	Canary Isl.
SH1-09b	SH1	SH1-9	*Lobaria immixta*	10687	-17,98037572	27,76127361	El Hierro	Canary Isl.
SH1-09c	SH1	SH1-9	*Lobaria immixta*	10688	-17,98037572	27,76127361	El Hierro	Canary Isl.
SH2-12d	SH2	SH2-12	*Lobaria immixta*	10783	-17,98679153	27,74271306	El Hierro	Canary Isl.
SH2-13a	SH2	SH2-13	*Lobaria immixta*	10784	-17,98694353	27,74270978	El Hierro	Canary Isl.
SH2-14b	SH2	SH2-14	*Lobaria immixta*	10788	-17,98695092	27,74272657	El Hierro	Canary Isl.
SH2-14c	SH2	SH2-14	*Lobaria immixta*	10789	-17,98695092	27,74272657	El Hierro	Canary Isl.
SH2-14d	SH2	SH2-14	*Lobaria immixta*	10790	-17,98695092	27,74272657	El Hierro	Canary Isl.
SH2-18d	SH2	SH2-18	*Lobaria immixta*	10804	-17,98684595	27,74253557	El Hierro	Canary Isl.
SH2-19d	SH2	SH2-19	*Lobaria immixta*	10808	-17,98690084	27,74248247	El Hierro	Canary Isl.
SH2-20d	SH2	SH2-20	*Lobaria immixta*	10812	-17,98693118	27,74230414	El Hierro	Canary Isl.
SH2-20e	SH2	SH2-20	*Lobaria immixta*	10813	-17,98693118	27,74230414	El Hierro	Canary Isl.
SH2-20f	SH2	SH2-20	*Lobaria immixta*	10814	-17,98693118	27,74230414	El Hierro	Canary Isl.
SH2-20g	SH2	SH2-20	*Lobaria immixta*	10815	-17,98693118	27,74230414	El Hierro	Canary Isl.
SH2-07d	SH2	SH2-7	*Lobaria immixta*	10767	-17,98661067	27,74288819	El Hierro	Canary Isl.
SH2-09c	SH2	SH2-9	*Lobaria immixta*	10773	-17,98670281	27,74288971	El Hierro	Canary Isl.
SH3-01	SH3	SH3-1	*Lobaria immixta*	11168	-18,01134	27,73292	El Hierro	Canary Isl.
SH3-10g	SH3	SH3-10	*Lobaria immixta*	11191	-18,01245973	27,73128006	El Hierro	Canary Isl.
SH3-10h	SH3	SH3-10	*Lobaria immixta*	11192	-18,01245973	27,73128006	El Hierro	Canary Isl.
SH3-10i	SH3	SH3-10	*Lobaria immixta*	11193	-18,01245973	27,73128006	El Hierro	Canary Isl.

ID	Pop	PopTree	Species	VoucherID	X	Y	Location	Area
SH3-11a	SH3	SH3-11	*Lobaria immixta*	11194	-18,01244196	27,7312887	El Hierro	Canary Isl.
SH3-11b	SH3	SH3-11	*Lobaria immixta*	11195	-18,01244196	27,7312887	El Hierro	Canary Isl.
SH3-11c	SH3	SH3-11	*Lobaria immixta*	11196	-18,01244196	27,7312887	El Hierro	Canary Isl.
SH3-12a	SH3	SH3-12	*Lobaria immixta*	11197	-18,01245157	27,73128584	El Hierro	Canary Isl.
SH3-12c	SH3	SH3-12	*Lobaria immixta*	11199	-18,01245157	27,73128584	El Hierro	Canary Isl.
SH3-02a	SH3	SH3-2	*Lobaria immixta*	11169	-18,01163668	27,73286091	El Hierro	Canary Isl.
SH3-26e	SH3	SH3-26	*Lobaria immixta*	11226	-18,01304316	27,73074484	El Hierro	Canary Isl.
SH3-26f	SH3	SH3-26	*Lobaria immixta*	11227	-18,01304316	27,73074484	El Hierro	Canary Isl.
SH3-26g	SH3	SH3-26	*Lobaria immixta*	11228	-18,01304316	27,73074484	El Hierro	Canary Isl.
SH3-27a	SH3	SH3-27	*Lobaria immixta*	11229	-18,01298652	27,73072513	El Hierro	Canary Isl.
SH3-27b	SH3	SH3-27	*Lobaria immixta*	11230	-18,01298652	27,73072513	El Hierro	Canary Isl.
SH3-27c	SH3	SH3-27	*Lobaria immixta*	11231	-18,01298652	27,73072513	El Hierro	Canary Isl.
SH3-28d	SH3	SH3-28	*Lobaria immixta*	11235	-18,01297216	27,73076838	El Hierro	Canary Isl.
SH3-28e	SH3	SH3-28	*Lobaria immixta*	11236	-18,01297216	27,73076838	El Hierro	Canary Isl.
SH3-28f	SH3	SH3-28	*Lobaria immixta*	11237	-18,01297216	27,73076838	El Hierro	Canary Isl.
SH3-29g	SH3	SH3-29	*Lobaria immixta*	11246	-18,01292079	27,73081193	El Hierro	Canary Isl.
SH3-29h	SH3	SH3-29	*Lobaria immixta*	11247	-18,01292079	27,73081193	El Hierro	Canary Isl.
SH3-29i	SH3	SH3-29	*Lobaria immixta*	11248	-18,01292079	27,73081193	El Hierro	Canary Isl.
SH3-03	SH3	SH3-3	*Lobaria immixta*	11171	-18,0118215	27,73244111	El Hierro	Canary Isl.
SH3-31d	SH3	SH3-31	*Lobaria immixta*	11256	-18,01287002	27,73086342	El Hierro	Canary Isl.
SH3-31e	SH3	SH3-31	*Lobaria immixta*	11257	-18,01287002	27,73086342	El Hierro	Canary Isl.
SH3-31f	SH3	SH3-31	*Lobaria immixta*	11258	-18,01287002	27,73086342	El Hierro	Canary Isl.
SH3-32b	SH3	SH3-32	*Lobaria immixta*	11260	-18,01291864	27,7308762	El Hierro	Canary Isl.
SH3-32c	SH3	SH3-32	*Lobaria immixta*	11261	-18,01291864	27,7308762	El Hierro	Canary Isl.
SH3-33a	SH3	SH3-33	*Lobaria immixta*	11262	-18,01282788	27,73083608	El Hierro	Canary Isl.
SH3-33b	SH3	SH3-33	*Lobaria immixta*	11263	-18,01282788	27,73083608	El Hierro	Canary Isl.
SH3-33c	SH3	SH3-33	*Lobaria immixta*	11264	-18,01282788	27,73083608	El Hierro	Canary Isl.

ID	Pop	PopTree	Species	VoucherID	X	Y	Location	Area
SH3-34a	SH3	SH3-34	*Lobaria immixta*	11265	-18,01279414	27,73079106	El Hierro	Canary Isl.
SH3-34b	SH3	SH3-34	*Lobaria immixta*	11266	-18,01279414	27,73079106	El Hierro	Canary Isl.
SH3-34c	SH3	SH3-34	*Lobaria immixta*	11267	-18,01279414	27,73079106	El Hierro	Canary Isl.
SH3-35a	SH3	SH3-35	*Lobaria immixta*	11268	-18,01275648	27,73077789	El Hierro	Canary Isl.
SH3-35b	SH3	SH3-35	*Lobaria immixta*	11269	-18,01275648	27,73077789	El Hierro	Canary Isl.
SH3-35c	SH3	SH3-35	*Lobaria immixta*	11270	-18,01275648	27,73077789	El Hierro	Canary Isl.
SH3-36a	SH3	SH3-36	*Lobaria immixta*	11271	-18,01276444	27,73074252	El Hierro	Canary Isl.
SH3-36b	SH3	SH3-36	*Lobaria immixta*	11272	-18,01276444	27,73074252	El Hierro	Canary Isl.
SH3-36c	SH3	SH3-36	*Lobaria immixta*	11273	-18,01276444	27,73074252	El Hierro	Canary Isl.
SH3-37a	SH3	SH3-37	*Lobaria immixta*	11274	-18,01269719	27,73076262	El Hierro	Canary Isl.
SH3-37b	SH3	SH3-37	*Lobaria immixta*	11275	-18,01269719	27,73076262	El Hierro	Canary Isl.
SH3-37c	SH3	SH3-37	*Lobaria immixta*	11276	-18,01269719	27,73076262	El Hierro	Canary Isl.
SH3-38b	SH3	SH3-38	*Lobaria immixta*	11278	-18,01269743	27,73075361	El Hierro	Canary Isl.
SH3-38c	SH3	SH3-38	*Lobaria immixta*	11279	-18,01269743	27,73075361	El Hierro	Canary Isl.
SH3-05a	SH3	SH3-5	*Lobaria immixta*	11173	-18,0117952	27,73216785	El Hierro	Canary Isl.
SH3-05b	SH3	SH3-5	*Lobaria immixta*	11174	-18,0117952	27,73216785	El Hierro	Canary Isl.
SH3-08a	SH3	SH3-8	*Lobaria immixta*	11181	-18,01229963	27,73145566	El Hierro	Canary Isl.
SM1-01a	SM1	SM1-1	*Lobaria immixta*	14703	-25,78249	37,85132	Sao Miguel	Azores
SM1-01b	SM1	SM1-1	*Lobaria immixta*	14704	-25,78249	37,85132	Sao Miguel	Azores
SM1-10a	SM1	SM1-10	*Lobaria immixta*	14755	-25,77994767	37,84954579	Sao Miguel	Azores
SM1-11b	SM1	SM1-11	*Lobaria immixta*	14761	-25,77995287	37,8495538	Sao Miguel	Azores
SM1-12a	SM1	SM1-12	*Lobaria immixta*	14767	-25,77989887	37,84960013	Sao Miguel	Azores
SM1-12b	SM1	SM1-12	*Lobaria immixta*	14768	-25,77989887	37,84960013	Sao Miguel	Azores
SM1-14a	SM1	SM1-14	*Lobaria immixta*	14776	-25,77986069	37,850091	Sao Miguel	Azores
SM1-14b	SM1	SM1-14	*Lobaria immixta*	14777	-25,77986069	37,850091	Sao Miguel	Azores
SM1-14f	SM1	SM1-14	*Lobaria immixta*	14781	-25,77986069	37,850091	Sao Miguel	Azores
SM1-14g	SM1	SM1-14	*Lobaria immixta*	14782	-25,77986069	37,850091	Sao Miguel	Azores

ID	Pop	PopTree	Species	VoucherID	X	Y	Location	Area
SM1-14h	SM1	SM1-14	*Lobaria immixta*	14783	-25,77986069	37,850091	Sao Miguel	Azores
SM1-15f	SM1	SM1-15	*Lobaria immixta*	14791	-25,77995407	37,85014236	Sao Miguel	Azores
SM1-15g	SM1	SM1-15	*Lobaria immixta*	14792	-25,77995407	37,85014236	Sao Miguel	Azores
SM1-15h	SM1	SM1-15	*Lobaria immixta*	14793	-25,77995407	37,85014236	Sao Miguel	Azores
SM1-15i	SM1	SM1-15	*Lobaria immixta*	14794	-25,77995407	37,85014236	Sao Miguel	Azores
SM1-16a	SM1	SM1-16	*Lobaria immixta*	14795	-25,77921261	37,85037101	Sao Miguel	Azores
SM1-16b	SM1	SM1-16	*Lobaria immixta*	14796	-25,77921261	37,85037101	Sao Miguel	Azores
SM1-17a	SM1	SM1-17	*Lobaria immixta*	14803	-25,77922983	37,85038278	Sao Miguel	Azores
SM1-17b	SM1	SM1-17	*Lobaria immixta*	14804	-25,77922983	37,85038278	Sao Miguel	Azores
SM1-02a	SM1	SM1-2	*Lobaria immixta*	14710	-25,78244388	37,85129367	Sao Miguel	Azores
SM1-02c	SM1	SM1-2	*Lobaria immixta*	14712	-25,78244388	37,85129367	Sao Miguel	Azores
SM1-20a	SM1	SM1-20	*Lobaria immixta*	14815	-25,77927359	37,85048144	Sao Miguel	Azores
SM1-20b	SM1	SM1-20	*Lobaria immixta*	14816	-25,77927359	37,85048144	Sao Miguel	Azores
SM1-20c	SM1	SM1-20	*Lobaria immixta*	14817	-25,77927359	37,85048144	Sao Miguel	Azores
SM1-04d	SM1	SM1-4	*Lobaria immixta*	14729	-25,78203	37,85154	Sao Miguel	Azores
SM1-04e	SM1	SM1-4	*Lobaria immixta*	14730	-25,78203	37,85154	Sao Miguel	Azores
SM1-05b	SM1	SM1-5	*Lobaria immixta*	14733	-25,78244	37,85155	Sao Miguel	Azores
SM1-05c	SM1	SM1-5	*Lobaria immixta*	14734	-25,78244	37,85155	Sao Miguel	Azores
SM1-06a	SM1	SM1-6	*Lobaria immixta*	14736	-25,78248612	37,85157632	Sao Miguel	Azores
SM1-06b	SM1	SM1-6	*Lobaria immixta*	14737	-25,78248612	37,85157632	Sao Miguel	Azores
SM1-06c	SM1	SM1-6	*Lobaria immixta*	14738	-25,78248612	37,85157632	Sao Miguel	Azores
SM1-07a	SM1	SM1-7	*Lobaria immixta*	14739	-25,78158	37,85164	Sao Miguel	Azores
SM1-07b	SM1	SM1-7	*Lobaria immixta*	14740	-25,78158	37,85164	Sao Miguel	Azores
SM1-09a	SM1	SM1-9	*Lobaria immixta*	14748	-25,77992	37,84953	Sao Miguel	Azores
SM1-09b	SM1	SM1-9	*Lobaria immixta*	14749	-25,77992	37,84953	Sao Miguel	Azores
SM1-09c	SM1	SM1-9	*Lobaria immixta*	14750	-25,77992	37,84953	Sao Miguel	Azores
SM1-09d	SM1	SM1-9	*Lobaria immixta*	14751	-25,77992	37,84953	Sao Miguel	Azores

ID	Pop	PopTree	Species	VoucherID	X	Y	Location	Area
SM2-10a	SM2	SM2-10	*Lobaria immixta*	14860	-25,32515881	37,75631884	Sao Miguel	Azores
SM2-11a	SM2	SM2-11	*Lobaria immixta*	14864	-25,32526894	37,75616125	Sao Miguel	Azores
SM2-11b	SM2	SM2-11	*Lobaria immixta*	14865	-25,32526894	37,75616125	Sao Miguel	Azores
SM2-11c	SM2	SM2-11	*Lobaria immixta*	14866	-25,32526894	37,75616125	Sao Miguel	Azores
SM2-12d	SM2	SM2-12	*Lobaria immixta*	14954	-25,32533161	37,75598804	Sao Miguel	Azores
SM2-13a	SM2	SM2-13	*Lobaria immixta*	14870	-25,32549449	37,75565161	Sao Miguel	Azores
SM2-13b	SM2	SM2-13	*Lobaria immixta*	14871	-25,32549449	37,75565161	Sao Miguel	Azores
SM2-13c	SM2	SM2-13	*Lobaria immixta*	14872	-25,32549449	37,75565161	Sao Miguel	Azores
SM2-14c	SM2	SM2-14	*Lobaria immixta*	14875	-25,32551488	37,75565951	Sao Miguel	Azores
SM2-15a	SM2	SM2-15	*Lobaria immixta*	14877	-25,3255214	37,75563297	Sao Miguel	Azores
SM2-15b	SM2	SM2-15	*Lobaria immixta*	14878	-25,3255214	37,75563297	Sao Miguel	Azores
SM2-15c	SM2	SM2-15	*Lobaria immixta*	14879	-25,3255214	37,75563297	Sao Miguel	Azores
SM2-15d	SM2	SM2-15	*Lobaria immixta*	14880	-25,3255214	37,75563297	Sao Miguel	Azores
SM2-16f	SM2	SM2-16	*Lobaria immixta*	14886	-25,32552647	37,75540774	Sao Miguel	Azores
SM2-18e	SM2	SM2-18	*Lobaria immixta*	14893	-25,32545216	37,75415767	Sao Miguel	Azores
SM2-18f	SM2	SM2-18	*Lobaria immixta*	14894	-25,32545216	37,75415767	Sao Miguel	Azores
SM2-18g	SM2	SM2-18	*Lobaria immixta*	14895	-25,32545216	37,75415767	Sao Miguel	Azores
SM2-19a	SM2	SM2-19	*Lobaria immixta*	14896	-25,32560154	37,75391475	Sao Miguel	Azores
SM2-19b	SM2	SM2-19	*Lobaria immixta*	14897	-25,32560154	37,75391475	Sao Miguel	Azores
SM2-19c	SM2	SM2-19	*Lobaria immixta*	14898	-25,32560154	37,75391475	Sao Miguel	Azores
SM2-19d	SM2	SM2-19	*Lobaria immixta*	14899	-25,32560154	37,75391475	Sao Miguel	Azores
SM2-19f	SM2	SM2-19	*Lobaria immixta*	14901	-25,32560154	37,75391475	Sao Miguel	Azores
SM2-19g	SM2	SM2-19	*Lobaria immixta*	14902	-25,32560154	37,75391475	Sao Miguel	Azores
SM2-20b	SM2	SM2-20	*Lobaria immixta*	14904	-25,32571252	37,75370743	Sao Miguel	Azores
SM2-21a	SM2	SM2-21	*Lobaria immixta*	14907	-25,32576584	37,75362789	Sao Miguel	Azores
SM2-21b	SM2	SM2-21	*Lobaria immixta*	14908	-25,32576584	37,75362789	Sao Miguel	Azores
SM2-21c	SM2	SM2-21	*Lobaria immixta*	14909	-25,32576584	37,75362789	Sao Miguel	Azores

ID	Pop	PopTree	Species	VoucherID	X	Y	Location	Area
SM2-22a	SM2	SM2-22	*Lobaria immixta*	14914	-25,32579074	37,7535874	Sao Miguel	Azores
SM2-22c	SM2	SM2-22	*Lobaria immixta*	14916	-25,32579074	37,7535874	Sao Miguel	Azores
SM2-22d	SM2	SM2-22	*Lobaria immixta*	14917	-25,32579074	37,7535874	Sao Miguel	Azores
SM2-24a	SM2	SM2-24	*Lobaria immixta*	14924	-25,32589133	37,75340631	Sao Miguel	Azores
SM2-07a	SM2	SM2-7	*Lobaria immixta*	14842	-25,32508411	37,75663662	Sao Miguel	Azores
SM2-07b	SM2	SM2-7	*Lobaria immixta*	14843	-25,32508411	37,75663662	Sao Miguel	Azores
SM2-07c	SM2	SM2-7	*Lobaria immixta*	14844	-25,32508411	37,75663662	Sao Miguel	Azores
SM2-08a	SM2	SM2-8	*Lobaria immixta*	14852	-25,32519424	37,75647904	Sao Miguel	Azores
SM2-08b	SM2	SM2-8	*Lobaria immixta*	14853	-25,32519424	37,75647904	Sao Miguel	Azores
SM2-08c	SM2	SM2-8	*Lobaria immixta*	14854	-25,32519424	37,75647904	Sao Miguel	Azores
SP1-13g	SP1	SP1-13	*Lobaria immixta*	11639	-17,83522995	28,61173835	La Palma	Canary Isl.
SP1-21d	SP1	SP1-21	*Lobaria immixta*	11678	-17,83510178	28,6113051	La Palma	Canary Isl.
SP1-21e	SP1	SP1-21	*Lobaria immixta*	11679	-17,83510178	28,6113051	La Palma	Canary Isl.
SP1-24d	SP1	SP1-24	*Lobaria immixta*	11693	-17,83506033	28,61124576	La Palma	Canary Isl.
SP1-25d	SP1	SP1-25	*Lobaria immixta*	11701	-17,83505832	28,61123693	La Palma	Canary Isl.
SP1-25e	SP1	SP1-25	*Lobaria immixta*	11702	-17,83505832	28,61123693	La Palma	Canary Isl.
SP1-25f	SP1	SP1-25	*Lobaria immixta*	11703	-17,83505832	28,61123693	La Palma	Canary Isl.
SP1-26c	SP1	SP1-26	*Lobaria immixta*	11707	-17,83501347	28,61121531	La Palma	Canary Isl.
SP1-26d	SP1	SP1-26	*Lobaria immixta*	11708	-17,83501347	28,61121531	La Palma	Canary Isl.
SP1-26e	SP1	SP1-26	*Lobaria immixta*	11709	-17,83501347	28,61121531	La Palma	Canary Isl.
SP1-26f	SP1	SP1-26	*Lobaria immixta*	11710	-17,83501347	28,61121531	La Palma	Canary Isl.
SP1-29a	SP1	SP1-29	*Lobaria immixta*	11730	-17,83488747	28,61118756	La Palma	Canary Isl.
SP1-29b	SP1	SP1-29	*Lobaria immixta*	11731	-17,83488747	28,61118756	La Palma	Canary Isl.
SP1-29c	SP1	SP1-29	*Lobaria immixta*	11732	-17,83488747	28,61118756	La Palma	Canary Isl.
SP1-31	SP1	SP1-31	*Lobaria immixta*	11741	-17,83497045	28,61115128	La Palma	Canary Isl.
SP1-32a	SP1	SP1-32	*Lobaria immixta*	11742	-17,8349779	28,61116808	La Palma	Canary Isl.
SP1-32b	SP1	SP1-32	*Lobaria immixta*	11743	-17,8349779	28,61116808	La Palma	Canary Isl.

ID	Pop	PopTree	Species	VoucherID	X	Y	Location	Area
SP1-33a	SP1	SP1-33	*Lobaria immixta*	11744	-17,83499651	28,61121007	La Palma	Canary Isl.
SP1-33b	SP1	SP1-33	*Lobaria immixta*	11745	-17,83499651	28,61121007	La Palma	Canary Isl.
SP1-33c	SP1	SP1-33	*Lobaria immixta*	11746	-17,83499651	28,61121007	La Palma	Canary Isl.
SP1-34a	SP1	SP1-34	*Lobaria immixta*	11747	-17,83494542	28,61121114	La Palma	Canary Isl.
SP1-35a	SP1	SP1-35	*Lobaria immixta*	11752	-17,83493536	28,61116693	La Palma	Canary Isl.
SP1-35b	SP1	SP1-35	*Lobaria immixta*	11753	-17,83493536	28,61116693	La Palma	Canary Isl.
SP1-36a	SP1	SP1-36	*Lobaria immixta*	11754	-17,83493778	28,61125708	La Palma	Canary Isl.
SP1-36b	SP1	SP1-36	*Lobaria immixta*	11755	-17,83493778	28,61125708	La Palma	Canary Isl.
SP1-37	SP1	SP1-37	*Lobaria immixta*	11756	-17,83502505	28,61121015	La Palma	Canary Isl.
SP2-10a	SP2	SP2-10	*Lobaria immixta*	12122	-17,77805327	28,76091351	La Palma	Canary Isl.
SP2-10c	SP2	SP2-10	*Lobaria immixta*	12124	-17,77805327	28,76091351	La Palma	Canary Isl.
SP2-10g	SP2	SP2-10	*Lobaria immixta*	12128	-17,77805327	28,76091351	La Palma	Canary Isl.
SP2-14a	SP2	SP2-14	*Lobaria immixta*	12147	-17,77798816	28,76072342	La Palma	Canary Isl.
SP2-21e	SP2	SP2-21	*Lobaria immixta*	12171	-17,77724977	28,76081005	La Palma	Canary Isl.
SP2-24	SP2	SP2-24	*Lobaria immixta*	12181	-17,77662209	28,76095621	La Palma	Canary Isl.
SP2-04a	SP2	SP2-4	*Lobaria immixta*	12107	-17,77782923	28,7607512	La Palma	Canary Isl.
SP2-04b	SP2	SP2-4	*Lobaria immixta*	12108	-17,77782923	28,7607512	La Palma	Canary Isl.
SP3-10a	SP3	SP3-10	*Lobaria immixta*	10838	-17,80443947	28,78837818	La Palma	Canary Isl.
SP3-14a	SP3	SP3-14	*Lobaria immixta*	10847	-17,80434446	28,78849494	La Palma	Canary Isl.
SP3-14b	SP3	SP3-14	*Lobaria immixta*	10848	-17,80434446	28,78849494	La Palma	Canary Isl.
SP3-16a	SP3	SP3-16	*Lobaria immixta*	10850	-17,80416209	28,78875122	La Palma	Canary Isl.
SP3-19b	SP3	SP3-19	*Lobaria immixta*	10857	-17,80371645	28,78872815	La Palma	Canary Isl.
SP3-20b	SP3	SP3-20	*Lobaria immixta*	10859	-17,80366878	28,78874456	La Palma	Canary Isl.
SP3-20c	SP3	SP3-20	*Lobaria immixta*	10860	-17,80366878	28,78874456	La Palma	Canary Isl.
SP3-23a	SP3	SP3-23	*Lobaria immixta*	10872	-17,80369873	28,78861965	La Palma	Canary Isl.
SP3-23b	SP3	SP3-23	*Lobaria immixta*	10873	-17,80369873	28,78861965	La Palma	Canary Isl.
SP3-23c	SP3	SP3-23	*Lobaria immixta*	10874	-17,80369873	28,78861965	La Palma	Canary Isl.

ID	Pop	PopTree	Species	VoucherID	X	Y	Location	Area
SP3-23d	SP3	SP3-23	*Lobaria immixta*	10875	-17,80369873	28,78861965	La Palma	Canary Isl.
SP3-07a	SP3	SP3-7	*Lobaria immixta*	10831	-17,80450122	28,78829043	La Palma	Canary Isl.
SP3-08a	SP3	SP3-8	*Lobaria immixta*	10833	-17,80443728	28,78836087	La Palma	Canary Isl.
SP4-14c	SP4	SP4-14	*Lobaria immixta*	11802	-17,85055731	28,80232328	La Palma	Canary Isl.
SP5-23h	SP5	SP5-23	*Lobaria immixta*	11931	-17,79119914	28,724078	La Palma	Canary Isl.
ST2-01a	ST2	ST2-1	*Lobaria immixta*	10953	-16,81055	28,32856	Tenerife	Canary Isl.
ST2-01b	ST2	ST2-1	*Lobaria immixta*	10954	-16,81055	28,32856	Tenerife	Canary Isl.
ST2-01c	ST2	ST2-1	*Lobaria immixta*	10955	-16,81055	28,32856	Tenerife	Canary Isl.
ST2-01d	ST2	ST2-1	*Lobaria immixta*	10956	-16,81055	28,32856	Tenerife	Canary Isl.
ST2-01e	ST2	ST2-1	*Lobaria immixta*	10957	-16,81055	28,32856	Tenerife	Canary Isl.
ST2-01f	ST2	ST2-1	*Lobaria immixta*	10958	-16,81055	28,32856	Tenerife	Canary Isl.
ST2-01g	ST2	ST2-1	*Lobaria immixta*	10959	-16,81055	28,32856	Tenerife	Canary Isl.
ST2-10a	ST2	ST2-10	*Lobaria immixta*	10995	-16,81061959	28,32874269	Tenerife	Canary Isl.
ST2-10b	ST2	ST2-10	*Lobaria immixta*	10996	-16,81061959	28,32874269	Tenerife	Canary Isl.
ST2-10c	ST2	ST2-10	*Lobaria immixta*	10997	-16,81061959	28,32874269	Tenerife	Canary Isl.
ST2-11a	ST2	ST2-11	*Lobaria immixta*	11000	-16,8106296	28,32874099	Tenerife	Canary Isl.
ST2-11b	ST2	ST2-11	*Lobaria immixta*	11001	-16,8106296	28,32874099	Tenerife	Canary Isl.
ST2-11c	ST2	ST2-11	*Lobaria immixta*	11002	-16,8106296	28,32874099	Tenerife	Canary Isl.
ST2-11d	ST2	ST2-11	*Lobaria immixta*	11003	-16,8106296	28,32874099	Tenerife	Canary Isl.
ST2-11e	ST2	ST2-11	*Lobaria immixta*	11004	-16,8106296	28,32874099	Tenerife	Canary Isl.
ST2-11f	ST2	ST2-11	*Lobaria immixta*	11005	-16,8106296	28,32874099	Tenerife	Canary Isl.
ST2-12b	ST2	ST2-12	*Lobaria immixta*	11007	-16,81063687	28,32875786	Tenerife	Canary Isl.
ST2-12c	ST2	ST2-12	*Lobaria immixta*	11008	-16,81063687	28,32875786	Tenerife	Canary Isl.
ST2-12d	ST2	ST2-12	*Lobaria immixta*	11009	-16,81063687	28,32875786	Tenerife	Canary Isl.
ST2-13a	ST2	ST2-13	*Lobaria immixta*	11011	-16,81064688	28,32875616	Tenerife	Canary Isl.
ST2-13b	ST2	ST2-13	*Lobaria immixta*	11012	-16,81064688	28,32875616	Tenerife	Canary Isl.
ST2-13c	ST2	ST2-13	*Lobaria immixta*	11013	-16,81064688	28,32875616	Tenerife	Canary Isl.

ID	Pop	PopTree	Species	VoucherID	X	Y	Location	Area
ST2-14a	ST2	ST2-14	*Lobaria immixta*	11016	-16,81065652	28,32875911	Tenerife	Canary Isl.
ST2-14b	ST2	ST2-14	*Lobaria immixta*	11017	-16,81065652	28,32875911	Tenerife	Canary Isl.
ST2-14c	ST2	ST2-14	*Lobaria immixta*	11018	-16,81065652	28,32875911	Tenerife	Canary Isl.
ST2-15a	ST2	ST2-15	*Lobaria immixta*	11019	-16,81067272	28,32867003	Tenerife	Canary Isl.
ST2-15b	ST2	ST2-15	*Lobaria immixta*	11020	-16,81067272	28,32867003	Tenerife	Canary Isl.
ST2-15c	ST2	ST2-15	*Lobaria immixta*	11021	-16,81067272	28,32867003	Tenerife	Canary Isl.
ST2-15d	ST2	ST2-15	*Lobaria immixta*	11022	-16,81067272	28,32867003	Tenerife	Canary Isl.
ST2-16a	ST2	ST2-16	*Lobaria immixta*	11023	-16,81065258	28,32866716	Tenerife	Canary Isl.
ST2-16b	ST2	ST2-16	*Lobaria immixta*	11024	-16,81065258	28,32866716	Tenerife	Canary Isl.
ST2-16c	ST2	ST2-16	*Lobaria immixta*	11025	-16,81065258	28,32866716	Tenerife	Canary Isl.
ST2-17a	ST2	ST2-17	*Lobaria immixta*	11030	-16,81061627	28,32858284	Tenerife	Canary Isl.
ST2-17b	ST2	ST2-17	*Lobaria immixta*	11031	-16,81061627	28,32858284	Tenerife	Canary Isl.
ST2-17c	ST2	ST2-17	*Lobaria immixta*	11032	-16,81061627	28,32858284	Tenerife	Canary Isl.
ST2-17d	ST2	ST2-17	*Lobaria immixta*	11033	-16,81061627	28,32858284	Tenerife	Canary Isl.
ST2-17e	ST2	ST2-17	*Lobaria immixta*	11034	-16,81061627	28,32858284	Tenerife	Canary Isl.
ST2-18b	ST2	ST2-18	*Lobaria immixta*	11036	-16,8106411	28,32854343	Tenerife	Canary Isl.
ST2-18c	ST2	ST2-18	*Lobaria immixta*	11037	-16,8106411	28,32854343	Tenerife	Canary Isl.
ST2-18d	ST2	ST2-18	*Lobaria immixta*	11038	-16,8106411	28,32854343	Tenerife	Canary Isl.
ST2-18e	ST2	ST2-18	*Lobaria immixta*	11039	-16,8106411	28,32854343	Tenerife	Canary Isl.
ST2-19a	ST2	ST2-19	*Lobaria immixta*	11042	-16,81064606	28,32853555	Tenerife	Canary Isl.
ST2-19b	ST2	ST2-19	*Lobaria immixta*	11043	-16,81064606	28,32853555	Tenerife	Canary Isl.
ST2-19c	ST2	ST2-19	*Lobaria immixta*	11044	-16,81064606	28,32853555	Tenerife	Canary Isl.
ST2-02a	ST2	ST2-2	*Lobaria immixta*	10961	-16,81055008	28,32856451	Tenerife	Canary Isl.
ST2-02b	ST2	ST2-2	*Lobaria immixta*	10962	-16,81055008	28,32856451	Tenerife	Canary Isl.
ST2-02c	ST2	ST2-2	*Lobaria immixta*	10963	-16,81055008	28,32856451	Tenerife	Canary Isl.
ST2-02d	ST2	ST2-2	*Lobaria immixta*	10964	-16,81055008	28,32856451	Tenerife	Canary Isl.
ST2-02e	ST2	ST2-2	*Lobaria immixta*	10965	-16,81055008	28,32856451	Tenerife	Canary Isl.

ID	Pop	PopTree	Species	VoucherID	X	Y	Location	Area
ST2-20d	ST2	ST2-20	*Lobaria immixta*	11051	-16,81055077	28,32856768	Tenerife	Canary Isl.
ST2-20e	ST2	ST2-20	*Lobaria immixta*	11052	-16,81055077	28,32856768	Tenerife	Canary Isl.
ST2-20f	ST2	ST2-20	*Lobaria immixta*	11053	-16,81055077	28,32856768	Tenerife	Canary Isl.
ST2-21b	ST2	ST2-21	*Lobaria immixta*	11055	-16,81058421	28,32848244	Tenerife	Canary Isl.
ST2-21c	ST2	ST2-21	*Lobaria immixta*	11056	-16,81058421	28,32848244	Tenerife	Canary Isl.
ST2-21d	ST2	ST2-21	*Lobaria immixta*	11057	-16,81058421	28,32848244	Tenerife	Canary Isl.
ST2-25c	ST2	ST2-25	*Lobaria immixta*	11071	-16,81058987	28,32843802	Tenerife	Canary Isl.
ST2-25d	ST2	ST2-25	*Lobaria immixta*	11072	-16,81058987	28,32843802	Tenerife	Canary Isl.
ST2-26d	ST2	ST2-26	*Lobaria immixta*	11080	-16,81043884	28,32841651	Tenerife	Canary Isl.
ST2-26e	ST2	ST2-26	*Lobaria immixta*	11081	-16,81043884	28,32841651	Tenerife	Canary Isl.
ST2-26f	ST2	ST2-26	*Lobaria immixta*	11082	-16,81043884	28,32841651	Tenerife	Canary Isl.
ST2-26g	ST2	ST2-26	*Lobaria immixta*	11083	-16,81043884	28,32841651	Tenerife	Canary Isl.
ST2-27d	ST2	ST2-27	*Lobaria immixta*	11090	-16,81033816	28,32840217	Tenerife	Canary Isl.
ST2-27e	ST2	ST2-27	*Lobaria immixta*	11091	-16,81033816	28,32840217	Tenerife	Canary Isl.
ST2-27f	ST2	ST2-27	*Lobaria immixta*	11092	-16,81033816	28,32840217	Tenerife	Canary Isl.
ST2-03a	ST2	ST2-3	*Lobaria immixta*	10966	-16,81054826	28,32856029	Tenerife	Canary Isl.
ST2-03b	ST2	ST2-3	*Lobaria immixta*	10967	-16,81054826	28,32856029	Tenerife	Canary Isl.
ST2-04a	ST2	ST2-4	*Lobaria immixta*	10968	-16,8104981	28,32868816	Tenerife	Canary Isl.
ST2-04b	ST2	ST2-4	*Lobaria immixta*	10969	-16,8104981	28,32868816	Tenerife	Canary Isl.
ST2-04c	ST2	ST2-4	*Lobaria immixta*	10970	-16,8104981	28,32868816	Tenerife	Canary Isl.
ST2-04d	ST2	ST2-4	*Lobaria immixta*	10971	-16,8104981	28,32868816	Tenerife	Canary Isl.
ST2-05a	ST2	ST2-5	*Lobaria immixta*	10972	-16,81048139	28,32873078	Tenerife	Canary Isl.
ST2-05b	ST2	ST2-5	*Lobaria immixta*	10973	-16,81048139	28,32873078	Tenerife	Canary Isl.
ST2-05c	ST2	ST2-5	*Lobaria immixta*	10974	-16,81048139	28,32873078	Tenerife	Canary Isl.
ST2-05d	ST2	ST2-5	*Lobaria immixta*	10975	-16,81048139	28,32873078	Tenerife	Canary Isl.
ST2-06a	ST2	ST2-6	*Lobaria immixta*	10976	-16,81048782	28,32872378	Tenerife	Canary Isl.
ST2-06b	ST2	ST2-6	*Lobaria immixta*	10977	-16,81048782	28,32872378	Tenerife	Canary Isl.

ID	Pop	PopTree	Species	VoucherID	X	Y	Location	Area
ST2-06c	ST2	ST2-6	*Lobaria immixta*	10978	-16,81048782	28,32872378	Tenerife	Canary Isl.
ST2-07a	ST2	ST2-7	*Lobaria immixta*	10979	-16,81052413	28,3288081	Tenerife	Canary Isl.
ST2-07b	ST2	ST2-7	*Lobaria immixta*	10980	-16,81052413	28,3288081	Tenerife	Canary Isl.
ST2-07c	ST2	ST2-7	*Lobaria immixta*	10981	-16,81052413	28,3288081	Tenerife	Canary Isl.
ST2-07d	ST2	ST2-7	*Lobaria immixta*	10982	-16,81052413	28,3288081	Tenerife	Canary Isl.
ST2-08a	ST2	ST2-8	*Lobaria immixta*	10984	-16,81058851	28,32873812	Tenerife	Canary Isl.
ST2-08b	ST2	ST2-8	*Lobaria immixta*	10985	-16,81058851	28,32873812	Tenerife	Canary Isl.
ST2-08c	ST2	ST2-8	*Lobaria immixta*	10986	-16,81058851	28,32873812	Tenerife	Canary Isl.
ST2-09a	ST2	ST2-9	*Lobaria immixta*	10991	-16,81056178	28,32872494	Tenerife	Canary Isl.
ST2-09b	ST2	ST2-9	*Lobaria immixta*	10992	-16,81056178	28,32872494	Tenerife	Canary Isl.
ST2-09d	ST2	ST2-9	*Lobaria immixta*	10994	-16,81056178	28,32872494	Tenerife	Canary Isl.
ST4-10a	ST4	ST4-10	*Lobaria immixta*	11411	-16,27077282	28,53977081	Tenerife	Canary Isl.
ST4-10b	ST4	ST4-10	*Lobaria immixta*	11412	-16,27077282	28,53977081	Tenerife	Canary Isl.
ST4-10c	ST4	ST4-10	*Lobaria immixta*	11413	-16,27077282	28,53977081	Tenerife	Canary Isl.
ST4-11a	ST4	ST4-11	*Lobaria immixta*	11417	-16,27081231	28,53979945	Tenerife	Canary Isl.
ST4-12a	ST4	ST4-12	*Lobaria immixta*	11419	-16,2708188	28,53979247	Tenerife	Canary Isl.
ST4-12b	ST4	ST4-12	*Lobaria immixta*	11420	-16,2708188	28,53979247	Tenerife	Canary Isl.
ST4-12c	ST4	ST4-12	*Lobaria immixta*	11421	-16,2708188	28,53979247	Tenerife	Canary Isl.
ST4-13a	ST4	ST4-13	*Lobaria immixta*	11425	-16,27083661	28,53980133	Tenerife	Canary Isl.
ST4-13c	ST4	ST4-13	*Lobaria immixta*	11427	-16,27083661	28,53980133	Tenerife	Canary Isl.
ST4-17a	ST4	ST4-17	*Lobaria immixta*	11439	-16,27074582	28,53964409	Tenerife	Canary Isl.
ST4-17b	ST4	ST4-17	*Lobaria immixta*	11440	-16,27074582	28,53964409	Tenerife	Canary Isl.
ST4-17c	ST4	ST4-17	*Lobaria immixta*	11441	-16,27074582	28,53964409	Tenerife	Canary Isl.
ST4-19a	ST4	ST4-19	*Lobaria immixta*	11448	-16,27080403	28,53956749	Tenerife	Canary Isl.
ST4-22a	ST4	ST4-22	*Lobaria immixta*	11459	-16,27092517	28,53949073	Tenerife	Canary Isl.
ST4-22c	ST4	ST4-22	*Lobaria immixta*	11461	-16,27092517	28,53949073	Tenerife	Canary Isl.
ST4-23a	ST4	ST4-23	*Lobaria immixta*	11465	-16,2709591	28,5394056	Tenerife	Canary Isl.

ID	Pop	PopTree	Species	VoucherID	X	Y	Location	Area
ST4-23b	ST4	ST4-23	*Lobaria immixta*	11466	-16,2709591	28,5394056	Tenerife	Canary Isl.
ST4-23c	ST4	ST4-23	*Lobaria immixta*	11467	-16,2709591	28,5394056	Tenerife	Canary Isl.
ST4-24a	ST4	ST4-24	*Lobaria immixta*	11468	-16,27091512	28,53942858	Tenerife	Canary Isl.
ST4-24b	ST4	ST4-24	*Lobaria immixta*	11469	-16,27091512	28,53942858	Tenerife	Canary Isl.
ST4-25	ST4	ST4-25	*Lobaria immixta*	11470	-16,27103361	28,53951447	Tenerife	Canary Isl.
ST4-03a	ST4	ST4-3	*Lobaria immixta*	11391	-16,27107156	28,53983079	Tenerife	Canary Isl.
ST4-05a	ST4	ST4-5	*Lobaria immixta*	11396	-16,27101938	28,53974103	Tenerife	Canary Isl.
ST4-06a	ST4	ST4-6	*Lobaria immixta*	11399	-16,27105004	28,53974074	Tenerife	Canary Isl.
ST4-06b	ST4	ST4-6	*Lobaria immixta*	11400	-16,27105004	28,53974074	Tenerife	Canary Isl.
ST4-09a	ST4	ST4-9	*Lobaria immixta*	11404	-16,27076633	28,53977778	Tenerife	Canary Isl.
ST4-09b	ST4	ST4-9	*Lobaria immixta*	11405	-16,27076633	28,53977778	Tenerife	Canary Isl.
ST4-09c	ST4	ST4-9	*Lobaria immixta*	11406	-16,27076633	28,53977778	Tenerife	Canary Isl.
ST4-09d	ST4	ST4-9	*Lobaria immixta*	11407	-16,27076633	28,53977778	Tenerife	Canary Isl.
ST5-11a	ST5	ST5-11	*Lobaria immixta*	11510	-16,17684743	28,55783639	Tenerife	Canary Isl.
ST5-12a	ST5	ST5-12	*Lobaria immixta*	11515	-16,17687255	28,55779709	Tenerife	Canary Isl.
ST5-12b	ST5	ST5-12	*Lobaria immixta*	11516	-16,17687255	28,55779709	Tenerife	Canary Isl.
ST5-13a	ST5	ST5-13	*Lobaria immixta*	11519	-16,17691138	28,55776774	Tenerife	Canary Isl.
ST5-13b	ST5	ST5-13	*Lobaria immixta*	11520	-16,17691138	28,55776774	Tenerife	Canary Isl.
ST5-13c	ST5	ST5-13	*Lobaria immixta*	11521	-16,17691138	28,55776774	Tenerife	Canary Isl.
ST5-14a	ST5	ST5-14	*Lobaria immixta*	11525	-16,17696249	28,5577673	Tenerife	Canary Isl.
ST5-14c	ST5	ST5-14	*Lobaria immixta*	11527	-16,17696249	28,5577673	Tenerife	Canary Isl.
ST5-15a	ST5	ST5-15	*Lobaria immixta*	11528	-16,17698617	28,5577845	Tenerife	Canary Isl.
ST5-15b	ST5	ST5-15	*Lobaria immixta*	11529	-16,17698617	28,5577845	Tenerife	Canary Isl.
ST5-15c	ST5	ST5-15	*Lobaria immixta*	11530	-16,17698617	28,5577845	Tenerife	Canary Isl.
ST5-15d	ST5	ST5-15	*Lobaria immixta*	11531	-16,17698617	28,5577845	Tenerife	Canary Isl.
ST5-16a	ST5	ST5-16	*Lobaria immixta*	11532	-16,17703642	28,55777623	Tenerife	Canary Isl.
ST5-16b	ST5	ST5-16	*Lobaria immixta*	11533	-16,17703642	28,55777623	Tenerife	Canary Isl.

ID	Pop	PopTree	Species	VoucherID	X	Y	Location	Area
ST5-16c	ST5	ST5-16	*Lobaria immixta*	11534	-16,17703642	28,55777623	Tenerife	Canary Isl.
ST5-17a	ST5	ST5-17	*Lobaria immixta*	11538	-16,17705221	28,5577877	Tenerife	Canary Isl.
ST5-17b	ST5	ST5-17	*Lobaria immixta*	11539	-16,17705221	28,5577877	Tenerife	Canary Isl.
ST5-17c	ST5	ST5-17	*Lobaria immixta*	11540	-16,17705221	28,5577877	Tenerife	Canary Isl.
ST5-18a	ST5	ST5-18	*Lobaria immixta*	11541	-16,17705997	28,55778182	Tenerife	Canary Isl.
ST5-18b	ST5	ST5-18	*Lobaria immixta*	11542	-16,17705997	28,55778182	Tenerife	Canary Isl.
ST5-18c	ST5	ST5-18	*Lobaria immixta*	11543	-16,17705997	28,55778182	Tenerife	Canary Isl.
ST5-21a	ST5	ST5-21	*Lobaria immixta*	11552	-16,17717437	28,55773774	Tenerife	Canary Isl.
ST5-21b	ST5	ST5-21	*Lobaria immixta*	11553	-16,17717437	28,55773774	Tenerife	Canary Isl.
ST5-21c	ST5	ST5-21	*Lobaria immixta*	11554	-16,17717437	28,55773774	Tenerife	Canary Isl.
ST5-22a	ST5	ST5-22	*Lobaria immixta*	11558	-16,17725476	28,55772451	Tenerife	Canary Isl.
ST5-22b	ST5	ST5-22	*Lobaria immixta*	11559	-16,17725476	28,55772451	Tenerife	Canary Isl.
ST5-22c	ST5	ST5-22	*Lobaria immixta*	11560	-16,17725476	28,55772451	Tenerife	Canary Isl.
ST5-23a	ST5	ST5-23	*Lobaria immixta*	11564	-16,177288	28,55775879	Tenerife	Canary Isl.
ST5-23b	ST5	ST5-23	*Lobaria immixta*	11565	-16,177288	28,55775879	Tenerife	Canary Isl.
ST5-25a	ST5	ST5-25	*Lobaria immixta*	11573	-16,1773239	28,55766342	Tenerife	Canary Isl.
ST5-25b	ST5	ST5-25	*Lobaria immixta*	11574	-16,1773239	28,55766342	Tenerife	Canary Isl.
ST5-25c	ST5	ST5-25	*Lobaria immixta*	11575	-16,1773239	28,55766342	Tenerife	Canary Isl.
ST5-26c	ST5	ST5-26	*Lobaria immixta*	11581	-16,17746746	28,55761587	Tenerife	Canary Isl.
ST5-07a	ST5	ST5-7	*Lobaria immixta*	11489	-16,17663966	28,55800035	Tenerife	Canary Isl.
ST5-07b	ST5	ST5-7	*Lobaria immixta*	11490	-16,17663966	28,55800035	Tenerife	Canary Isl.
ST5-07c	ST5	ST5-7	*Lobaria immixta*	11491	-16,17663966	28,55800035	Tenerife	Canary Isl.
ST5-08a	ST5	ST5-8	*Lobaria immixta*	11495	-16,17664804	28,55795583	Tenerife	Canary Isl.
ST5-08b	ST5	ST5-8	*Lobaria immixta*	11496	-16,17664804	28,55795583	Tenerife	Canary Isl.
ST5-08c	ST5	ST5-8	*Lobaria immixta*	11497	-16,17664804	28,55795583	Tenerife	Canary Isl.
ST5-09a	ST5	ST5-9	*Lobaria immixta*	11501	-16,17668686	28,55792649	Tenerife	Canary Isl.
ST5-09b	ST5	ST5-9	*Lobaria immixta*	11502	-16,17668686	28,55792649	Tenerife	Canary Isl.

ID	Pop	PopTree	Species	VoucherID	X	Y	Location	Area
ST6-11a	ST6	ST6-11	*Lobaria immixta*	11123	-16,22821278	28,54281359	Tenerife	Canary Isl.
ST6-11b	ST6	ST6-11	*Lobaria immixta*	11124	-16,22821278	28,54281359	Tenerife	Canary Isl.
ST6-11d	ST6	ST6-11	*Lobaria immixta*	11126	-16,22821278	28,54281359	Tenerife	Canary Isl.
ST6-14	ST6	ST6-14	*Lobaria immixta*	11132	-16,22827619	28,54282067	Tenerife	Canary Isl.
ST6-18a	ST6	ST6-18	*Lobaria immixta*	11141	-16,22823239	28,54289101	Tenerife	Canary Isl.
ST6-20a	ST6	ST6-20	*Lobaria immixta*	11148	-16,22813124	28,54298218	Tenerife	Canary Isl.
ST6-21b	ST6	ST6-21	*Lobaria immixta*	11154	-16,22812475	28,54298915	Tenerife	Canary Isl.
ST6-21c	ST6	ST6-21	*Lobaria immixta*	11155	-16,22812475	28,54298915	Tenerife	Canary Isl.
ST6-22a	ST6	ST6-22	*Lobaria immixta*	11159	-16,22808076	28,54301211	Tenerife	Canary Isl.
ST6-22b	ST6	ST6-22	*Lobaria immixta*	11160	-16,22808076	28,54301211	Tenerife	Canary Isl.
ST6-22c	ST6	ST6-22	*Lobaria immixta*	11161	-16,22808076	28,54301211	Tenerife	Canary Isl.
ST6-23a	ST6	ST6-23	*Lobaria immixta*	11162	-16,22807572	28,54301137	Tenerife	Canary Isl.
ST6-24b	ST6	ST6-24	*Lobaria immixta*	11166	-16,22827039	28,5428022	Tenerife	Canary Isl.
ST6-24c	ST6	ST6-24	*Lobaria immixta*	11167	-16,22827039	28,5428022	Tenerife	Canary Isl.
ST6-05a	ST6	ST6-5	*Lobaria immixta*	11107	-16,22809427	28,54307155	Tenerife	Canary Isl.
ST6-06c	ST6	ST6-6	*Lobaria immixta*	11112	-16,22802938	28,54314127	Tenerife	Canary Isl.
ST6-09c	ST6	ST6-9	*Lobaria immixta*	11119	-16,22802528	28,54288415	Tenerife	Canary Isl.
TE1-01a	TE1	TE1-1	*Lobaria immixta*	13943	-27,20365	38,75077	Terceira	Azores
TE1-01b	TE1	TE1-1	*Lobaria immixta*	13944	-27,20365	38,75077	Terceira	Azores
TE1-12a	TE1	TE1-12	*Lobaria immixta*	13988	-27,20368618	38,75077281	Terceira	Azores
TE1-12d	TE1	TE1-12	*Lobaria immixta*	13991	-27,20368618	38,75077281	Terceira	Azores
TE1-05a	TE1	TE1-5	*Lobaria immixta*	13959	-27,20373049	38,75078985	Terceira	Azores
TE1-05b	TE1	TE1-5	*Lobaria immixta*	13960	-27,20373049	38,75078985	Terceira	Azores
TE1-05c	TE1	TE1-5	*Lobaria immixta*	13961	-27,20373049	38,75078985	Terceira	Azores
TE1-08a	TE1	TE1-8	*Lobaria immixta*	13976	-27,20373911	38,75080093	Terceira	Azores
TE1-08b	TE1	TE1-8	*Lobaria immixta*	13977	-27,20373911	38,75080093	Terceira	Azores
TE2-01a	TE2	TE2-1	*Lobaria immixta*	14137	-27,20891	38,73382	Terceira	Azores

ID	Pop	PopTree	Species	VoucherID	X	Y	Location	Area
TE2-01b	TE2	TE2-1	*Lobaria immixta*	14138	-27,20891	38,73382	Terceira	Azores
TE2-10a	TE2	TE2-10	*Lobaria immixta*	14171	-27,20876224	38,73381437	Terceira	Azores
TE2-10b	TE2	TE2-10	*Lobaria immixta*	14172	-27,20876224	38,73381437	Terceira	Azores
TE2-10d	TE2	TE2-10	*Lobaria immixta*	14174	-27,20876224	38,73381437	Terceira	Azores
TE2-11a	TE2	TE2-11	*Lobaria immixta*	14175	-27,20876248	38,73381349	Terceira	Azores
TE2-11b	TE2	TE2-11	*Lobaria immixta*	14176	-27,20876248	38,73381349	Terceira	Azores
TE2-11f	TE2	TE2-11	*Lobaria immixta*	14180	-27,20876248	38,73381349	Terceira	Azores
TE2-11h	TE2	TE2-11	*Lobaria immixta*	14182	-27,20876248	38,73381349	Terceira	Azores
TE2-12e	TE2	TE2-12	*Lobaria immixta*	31774	-27,20871714	38,73380731	Terceira	Azores
TE2-13a	TE2	TE2-13	*Lobaria immixta*	14186	-27,2087194	38,73380699	Terceira	Azores
TE2-13b	TE2	TE2-13	*Lobaria immixta*	14187	-27,2087194	38,73380699	Terceira	Azores
TE2-13c	TE2	TE2-13	*Lobaria immixta*	14188	-27,2087194	38,73380699	Terceira	Azores
TE2-14a	TE2	TE2-14	*Lobaria immixta*	14189	-27,20869103	38,73379155	Terceira	Azores
TE2-15a	TE2	TE2-15	*Lobaria immixta*	14194	-27,20874808	38,73378514	Terceira	Azores
TE2-15b	TE2	TE2-15	*Lobaria immixta*	14195	-27,20874808	38,73378514	Terceira	Azores
TE2-15c	TE2	TE2-15	*Lobaria immixta*	14196	-27,20874808	38,73378514	Terceira	Azores
TE2-15d	TE2	TE2-15	*Lobaria immixta*	14197	-27,20874808	38,73378514	Terceira	Azores
TE2-16a	TE2	TE2-16	*Lobaria immixta*	14198	-27,20874574	38,73378529	Terceira	Azores
TE2-17a	TE2	TE2-17	*Lobaria immixta*	14200	-27,20873998	38,7337853	Terceira	Azores
TE2-17b	TE2	TE2-17	*Lobaria immixta*	14201	-27,20873998	38,7337853	Terceira	Azores
TE2-18c	TE2	TE2-18	*Lobaria immixta*	14204	-27,20865356	38,73381011	Terceira	Azores
TE2-19a	TE2	TE2-19	*Lobaria immixta*	14205	-27,20865438	38,73380948	Terceira	Azores
TE2-19b	TE2	TE2-19	*Lobaria immixta*	14206	-27,20865438	38,73380948	Terceira	Azores
TE2-19c	TE2	TE2-19	*Lobaria immixta*	14207	-27,20865438	38,73380948	Terceira	Azores
TE2-02d	TE2	TE2-2	*Lobaria immixta*	14142	-27,20885247	38,7338201	Terceira	Azores
TE2-24c	TE2	TE2-24	*Lobaria immixta*	14226	-27,20858741	38,73382861	Terceira	Azores
TE2-24d	TE2	TE2-24	*Lobaria immixta*	14227	-27,20858741	38,73382861	Terceira	Azores

ID	Pop	PopTree	Species	VoucherID	X	Y	Location	Area
TE2-24e	TE2	TE2-24	*Lobaria immixta*	14228	-27,20858741	38,73382861	Terceira	Azores
TE2-25f	TE2	TE2-25	*Lobaria immixta*	14002	-27,20847939	38,73385963	Terceira	Azores
TE2-25g	TE2	TE2-25	*Lobaria immixta*	14003	-27,20847939	38,73385963	Terceira	Azores
TE2-25h	TE2	TE2-25	*Lobaria immixta*	14004	-27,20847939	38,73385963	Terceira	Azores
TE2-28c	TE2	TE2-28	*Lobaria immixta*	14015	-27,20841202	38,73383122	Terceira	Azores
TE2-03a	TE2	TE2-3	*Lobaria immixta*	14143	-27,20882946	38,73382014	Terceira	Azores
TE2-30a	TE2	TE2-30	*Lobaria immixta*	14018	-27,20845436	38,73392496	Terceira	Azores
TE2-31a	TE2	TE2-31	*Lobaria immixta*	14019	-27,20797	38,73377	Terceira	Azores
TE2-31d	TE2	TE2-31	*Lobaria immixta*	14022	-27,20797	38,73377	Terceira	Azores
TE2-32a	TE2	TE2-32	*Lobaria immixta*	14024	-27,2079608	38,73377002	Terceira	Azores
TE2-32b	TE2	TE2-32	*Lobaria immixta*	14025	-27,2079608	38,73377002	Terceira	Azores
TE2-32c	TE2	TE2-32	*Lobaria immixta*	14026	-27,2079608	38,73377002	Terceira	Azores
TE2-32d	TE2	TE2-32	*Lobaria immixta*	14027	-27,2079608	38,73377002	Terceira	Azores
TE2-33c	TE2	TE2-33	*Lobaria immixta*	14030	-27,20793858	38,73377472	Terceira	Azores
TE2-34a	TE2	TE2-34	*Lobaria immixta*	14032	-27,20793858	38,73377472	Terceira	Azores
TE2-04b	TE2	TE2-4	*Lobaria immixta*	14146	-27,20872126	38,73378952	Terceira	Azores
TE2-04c	TE2	TE2-4	*Lobaria immixta*	14147	-27,20872126	38,73378952	Terceira	Azores
TE3-07c	TE3	TE3-7	*Lobaria immixta*	14061	-27,27879888	38,71991025	Terceira	Azores
TE4-01b	TE4	TE4-1	*Lobaria immixta*	14064	-27,23517	38,69481	Terceira	Azores
TE4-13a	TE4	TE4-13	*Lobaria immixta*	14122	-27,235099	38,69493808	Terceira	Azores
TE4-13b	TE4	TE4-13	*Lobaria immixta*	14123	-27,235099	38,69493808	Terceira	Azores
TE4-13c	TE4	TE4-13	*Lobaria immixta*	14124	-27,235099	38,69493808	Terceira	Azores
TE4-14f	TE4	TE4-14	*Lobaria immixta*	14135	-27,23513585	38,69490348	Terceira	Azores
TE4-14g	TE4	TE4-14	*Lobaria immixta*	14136	-27,23513585	38,69490348	Terceira	Azores
TE4-02a	TE4	TE4-2	*Lobaria immixta*	14065	-27,23520989	38,69482794	Terceira	Azores
TE4-02b	TE4	TE4-2	*Lobaria immixta*	14066	-27,23520989	38,69482794	Terceira	Azores
TE4-02c	TE4	TE4-2	*Lobaria immixta*	14067	-27,23520989	38,69482794	Terceira	Azores

ID	Pop	PopTree	Species	VoucherID	X	Y	Location	Area
TE4-02d	TE4	TE4-2	*Lobaria immixta*	14068	-27,23520989	38,69482794	Terceira	Azores
TE4-03e	TE4	TE4-3	*Lobaria immixta*	14074	-27,23520697	38,69485488	Terceira	Azores
TE4-05a	TE4	TE4-5	*Lobaria immixta*	14081	-27,23526088	38,69490229	Terceira	Azores
TE4-05b	TE4	TE4-5	*Lobaria immixta*	14082	-27,23526088	38,69490229	Terceira	Azores
TE4-05c	TE4	TE4-5	*Lobaria immixta*	14083	-27,23526088	38,69490229	Terceira	Azores
TE4-05d	TE4	TE4-5	*Lobaria immixta*	14084	-27,23526088	38,69490229	Terceira	Azores
TE4-06c	TE4	TE4-6	*Lobaria immixta*	14088	-27,23521386	38,69492823	Terceira	Azores
TE4-07b	TE4	TE4-7	*Lobaria immixta*	14093	-27,23515662	38,69500638	Terceira	Azores
TE4-07c	TE4	TE4-7	*Lobaria immixta*	14094	-27,23515662	38,69500638	Terceira	Azores
TE4-07d	TE4	TE4-7	*Lobaria immixta*	14095	-27,23515662	38,69500638	Terceira	Azores
TE4-07e	TE4	TE4-7	*Lobaria immixta*	14096	-27,23515662	38,69500638	Terceira	Azores
TE4-07f	TE4	TE4-7	*Lobaria immixta*	14097	-27,23515662	38,69500638	Terceira	Azores
TE4-08a	TE4	TE4-8	*Lobaria immixta*	14098	-27,23513364	38,69500581	Terceira	Azores
TE4-08b	TE4	TE4-8	*Lobaria immixta*	14099	-27,23513364	38,69500581	Terceira	Azores
TE4-08c	TE4	TE4-8	*Lobaria immixta*	14100	-27,23513364	38,69500581	Terceira	Azores
TE4-09a	TE4	TE4-9	*Lobaria immixta*	14101	-27,2351215	38,69502112	Terceira	Azores
TE4-09b	TE4	TE4-9	*Lobaria immixta*	14102	-27,2351215	38,69502112	Terceira	Azores
TE4-09c	TE4	TE4-9	*Lobaria immixta*	14103	-27,2351215	38,69502112	Terceira	Azores
FA1-01a	FA1	FA1-1	*Lobaria macaronesica*	14242	-28,77222001	38,58463	Faial	Azores
FA1-01b	FA1	FA1-1	*Lobaria macaronesica*	14243	-28,77222001	38,58463	Faial	Azores
FA1-01c	FA1	FA1-1	*Lobaria macaronesica*	14244	-28,77222001	38,58463	Faial	Azores
FA1-01d	FA1	FA1-1	*Lobaria macaronesica*	14245	-28,77222001	38,58463	Faial	Azores
FA1-01e	FA1	FA1-1	*Lobaria macaronesica*	14246	-28,77222001	38,58463	Faial	Azores
FA1-10b	FA1	FA1-10	*Lobaria macaronesica*	14287	-28,7720098	38,5848187	Faial	Azores
FA1-10c	FA1	FA1-10	*Lobaria macaronesica*	14288	-28,7720098	38,5848187	Faial	Azores
FA1-10d	FA1	FA1-10	*Lobaria macaronesica*	14289	-28,7720098	38,5848187	Faial	Azores
FA1-10e	FA1	FA1-10	*Lobaria macaronesica*	14290	-28,7720098	38,5848187	Faial	Azores

ID	Pop	PopTree	Species	VoucherID	X	Y	Location	Area
FA1-11d	FA1	FA1-11	*Lobaria macaronesica*	14295	-28,77200679	38,58481739	Faial	Azores
FA1-11e	FA1	FA1-11	*Lobaria macaronesica*	14296	-28,77200679	38,58481739	Faial	Azores
FA1-11g	FA1	FA1-11	*Lobaria macaronesica*	14298	-28,77200679	38,58481739	Faial	Azores
FA1-11h	FA1	FA1-11	*Lobaria macaronesica*	14299	-28,77200679	38,58481739	Faial	Azores
FA1-11i	FA1	FA1-11	*Lobaria macaronesica*	14300	-28,77200679	38,58481739	Faial	Azores
FA1-12d	FA1	FA1-12	*Lobaria macaronesica*	14304	-28,77200506	38,58481857	Faial	Azores
FA1-12e	FA1	FA1-12	*Lobaria macaronesica*	14305	-28,77200506	38,58481857	Faial	Azores
FA1-13a	FA1	FA1-13	*Lobaria macaronesica*	14306	-28,77197076	38,58482098	Faial	Azores
FA1-13b	FA1	FA1-13	*Lobaria macaronesica*	14307	-28,77197076	38,58482098	Faial	Azores
FA1-14a	FA1	FA1-14	*Lobaria macaronesica*	14308	-28,77196167	38,5847765	Faial	Azores
FA1-14b	FA1	FA1-14	*Lobaria macaronesica*	14309	-28,77196167	38,5847765	Faial	Azores
FA1-14c	FA1	FA1-14	*Lobaria macaronesica*	14310	-28,77196167	38,5847765	Faial	Azores
FA1-14d	FA1	FA1-14	*Lobaria macaronesica*	14311	-28,77196167	38,5847765	Faial	Azores
FA1-15a	FA1	FA1-15	*Lobaria macaronesica*	14312	-28,77196856	38,58477668	Faial	Azores
FA1-15c	FA1	FA1-15	*Lobaria macaronesica*	14314	-28,77196856	38,58477668	Faial	Azores
FA1-15d	FA1	FA1-15	*Lobaria macaronesica*	14315	-28,77196856	38,58477668	Faial	Azores
FA1-16a	FA1	FA1-16	*Lobaria macaronesica*	14316	-28,77197238	38,58478707	Faial	Azores
FA1-16b	FA1	FA1-16	*Lobaria macaronesica*	14317	-28,77197238	38,58478707	Faial	Azores
FA1-16d	FA1	FA1-16	*Lobaria macaronesica*	14319	-28,77197238	38,58478707	Faial	Azores
FA1-16e	FA1	FA1-16	*Lobaria macaronesica*	14320	-28,77197238	38,58478707	Faial	Azores
FA1-16f	FA1	FA1-16	*Lobaria macaronesica*	14321	-28,77197238	38,58478707	Faial	Azores
FA1-16g	FA1	FA1-16	*Lobaria macaronesica*	14322	-28,77197238	38,58478707	Faial	Azores
FA1-17a	FA1	FA1-17	*Lobaria macaronesica*	14328	-28,77194347	38,58477238	Faial	Azores
FA1-17b	FA1	FA1-17	*Lobaria macaronesica*	14329	-28,77194347	38,58477238	Faial	Azores
FA1-17c	FA1	FA1-17	*Lobaria macaronesica*	14330	-28,77194347	38,58477238	Faial	Azores
FA1-18b	FA1	FA1-18	*Lobaria macaronesica*	14334	-28,77182883	38,58476784	Faial	Azores
FA1-18c	FA1	FA1-18	*Lobaria macaronesica*	14335	-28,77182883	38,58476784	Faial	Azores

**ID**	**Pop**	**PopTree**	**Species**	**VoucherID**	**X**	**Y**	**Location**	**Area**
FA1-18d	FA1	FA1-18	*Lobaria macaronesica*	14336	-28,77182883	38,58476784	Faial	Azores
FA1-19a	FA1	FA1-19	*Lobaria macaronesica*	14337	-28,77182854	38,58477233	Faial	Azores
FA1-19b	FA1	FA1-19	*Lobaria macaronesica*	14338	-28,77182854	38,58477233	Faial	Azores
FA1-19c	FA1	FA1-19	*Lobaria macaronesica*	14339	-28,77182854	38,58477233	Faial	Azores
FA1-20a	FA1	FA1-20	*Lobaria macaronesica*	14341	-28,77180559	38,58477268	Faial	Azores
FA1-20c	FA1	FA1-20	*Lobaria macaronesica*	14343	-28,77180559	38,58477268	Faial	Azores
FA1-20d	FA1	FA1-20	*Lobaria macaronesica*	14344	-28,77180559	38,58477268	Faial	Azores
FA1-22b	FA1	FA1-22	*Lobaria macaronesica*	14349	-28,77179502	38,58476935	Faial	Azores
FA1-22c	FA1	FA1-22	*Lobaria macaronesica*	14350	-28,77179502	38,58476935	Faial	Azores
FA1-22e	FA1	FA1-22	*Lobaria macaronesica*	14352	-28,77179502	38,58476935	Faial	Azores
FA1-23a	FA1	FA1-23	*Lobaria macaronesica*	14353	-28,77180583	38,58475883	Faial	Azores
FA1-23c	FA1	FA1-23	*Lobaria macaronesica*	14355	-28,77180583	38,58475883	Faial	Azores
FA1-23d	FA1	FA1-23	*Lobaria macaronesica*	14356	-28,77180583	38,58475883	Faial	Azores
FA1-23e	FA1	FA1-23	*Lobaria macaronesica*	14357	-28,77180583	38,58475883	Faial	Azores
FA1-23f	FA1	FA1-23	*Lobaria macaronesica*	14358	-28,77180583	38,58475883	Faial	Azores
FA1-24a	FA1	FA1-24	*Lobaria macaronesica*	14359	-28,77179166	38,58474465	Faial	Azores
FA1-24b	FA1	FA1-24	*Lobaria macaronesica*	14360	-28,77179166	38,58474465	Faial	Azores
FA1-25a	FA1	FA1-25	*Lobaria macaronesica*	14361	-28,77179746	38,58473485	Faial	Azores
FA1-26a	FA1	FA1-26	*Lobaria macaronesica*	14362	-28,77179746	38,58473485	Faial	Azores
FA1-26c	FA1	FA1-26	*Lobaria macaronesica*	14364	-28,77179746	38,58473485	Faial	Azores
FA1-26d	FA1	FA1-26	*Lobaria macaronesica*	14365	-28,77179746	38,58473485	Faial	Azores
FA1-27a	FA1	FA1-27	*Lobaria macaronesica*	14366	-28,77183351	38,58468358	Faial	Azores
FA1-27c	FA1	FA1-27	*Lobaria macaronesica*	14368	-28,77183351	38,58468358	Faial	Azores
FA1-27d	FA1	FA1-27	*Lobaria macaronesica*	14369	-28,77183351	38,58468358	Faial	Azores
FA1-29a	FA1	FA1-29	*Lobaria macaronesica*	14373	-28,77176779	38,58471067	Faial	Azores
FA1-29b	FA1	FA1-29	*Lobaria macaronesica*	14374	-28,77176779	38,58471067	Faial	Azores
FA1-29c	FA1	FA1-29	*Lobaria macaronesica*	14375	-28,77176779	38,58471067	Faial	Azores

ID	Pop	PopTree	Species	VoucherID	X	Y	Location	Area
FA1-30a	FA1	FA1-30	*Lobaria macaronesica*	14376	-28,77160489	38,5846669	Faial	Azores
FA1-30b	FA1	FA1-30	*Lobaria macaronesica*	14377	-28,77160489	38,5846669	Faial	Azores
FA1-30c	FA1	FA1-30	*Lobaria macaronesica*	14378	-28,77160489	38,5846669	Faial	Azores
FA1-30d	FA1	FA1-30	*Lobaria macaronesica*	14379	-28,77160489	38,5846669	Faial	Azores
FA1-30e	FA1	FA1-30	*Lobaria macaronesica*	14380	-28,77160489	38,5846669	Faial	Azores
FA1-31a	FA1	FA1-31	*Lobaria macaronesica*	14381	-28,77159357	38,58465672	Faial	Azores
FA1-31b	FA1	FA1-31	*Lobaria macaronesica*	14382	-28,77159357	38,58465672	Faial	Azores
FA1-32a	FA1	FA1-32	*Lobaria macaronesica*	14384	-28,77140081	38,58455886	Faial	Azores
FA1-33a	FA1	FA1-33	*Lobaria macaronesica*	14386	-28,77136619	38,58452293	Faial	Azores
FA1-34a	FA1	FA1-34	*Lobaria macaronesica*	14387	-28,77132293	38,58445935	Faial	Azores
FA1-34b	FA1	FA1-34	*Lobaria macaronesica*	14388	-28,77132293	38,58445935	Faial	Azores
FA1-34c	FA1	FA1-34	*Lobaria macaronesica*	14389	-28,77132293	38,58445935	Faial	Azores
FA1-34d	FA1	FA1-34	*Lobaria macaronesica*	14390	-28,77132293	38,58445935	Faial	Azores
FA1-34e	FA1	FA1-34	*Lobaria macaronesica*	14391	-28,77132293	38,58445935	Faial	Azores
FA1-34f	FA1	FA1-34	*Lobaria macaronesica*	14392	-28,77132293	38,58445935	Faial	Azores
FA1-35a	FA1	FA1-35	*Lobaria macaronesica*	14393	-28,77131829	38,5844676	Faial	Azores
FA1-35b	FA1	FA1-35	*Lobaria macaronesica*	14394	-28,77131829	38,5844676	Faial	Azores
FA1-35c	FA1	FA1-35	*Lobaria macaronesica*	14395	-28,77131829	38,5844676	Faial	Azores
FA1-36a	FA1	FA1-36	*Lobaria macaronesica*	14396	-28,77130758	38,58447084	Faial	Azores
FA1-36b	FA1	FA1-36	*Lobaria macaronesica*	14397	-28,77130758	38,58447084	Faial	Azores
FA1-36c	FA1	FA1-36	*Lobaria macaronesica*	14398	-28,77130758	38,58447084	Faial	Azores
FA1-36d	FA1	FA1-36	*Lobaria macaronesica*	14399	-28,77130758	38,58447084	Faial	Azores
FA1-37a	FA1	FA1-37	*Lobaria macaronesica*	14400	-28,77130382	38,58446233	Faial	Azores
FA1-37b	FA1	FA1-37	*Lobaria macaronesica*	14401	-28,77130382	38,58446233	Faial	Azores
FA1-37c	FA1	FA1-37	*Lobaria macaronesica*	14402	-28,77130382	38,58446233	Faial	Azores
FA1-37d	FA1	FA1-37	*Lobaria macaronesica*	14403	-28,77130382	38,58446233	Faial	Azores
FA1-37e	FA1	FA1-37	*Lobaria macaronesica*	14404	-28,77130382	38,58446233	Faial	Azores

ID	Pop	PopTree	Species	VoucherID	X	Y	Location	Area
FA1-37f	FA1	FA1-37	*Lobaria macaronesica*	14405	-28,77130382	38,58446233	Faial	Azores
FA1-38a	FA1	FA1-38	*Lobaria macaronesica*	14406	-28,77130587	38,58445812	Faial	Azores
FA1-38b	FA1	FA1-38	*Lobaria macaronesica*	14407	-28,77130587	38,58445812	Faial	Azores
FA1-38c	FA1	FA1-38	*Lobaria macaronesica*	14408	-28,77130587	38,58445812	Faial	Azores
FA1-40c	FA1	FA1-40	*Lobaria macaronesica*	14412	-28,77119246	38,58444418	Faial	Azores
FA1-40d	FA1	FA1-40	*Lobaria macaronesica*	14413	-28,77119246	38,58444418	Faial	Azores
FA1-41a	FA1	FA1-41	*Lobaria macaronesica*	14414	-28,7710212	38,5844303	Faial	Azores
FA1-41b	FA1	FA1-41	*Lobaria macaronesica*	14415	-28,7710212	38,5844303	Faial	Azores
FA1-41c	FA1	FA1-41	*Lobaria macaronesica*	14416	-28,7710212	38,5844303	Faial	Azores
FA1-42a	FA1	FA1-42	*Lobaria macaronesica*	14417	-28,77101802	38,58442165	Faial	Azores
FA1-42b	FA1	FA1-42	*Lobaria macaronesica*	14418	-28,77101802	38,58442165	Faial	Azores
FA1-42c	FA1	FA1-42	*Lobaria macaronesica*	14419	-28,77101802	38,58442165	Faial	Azores
FA1-42d	FA1	FA1-42	*Lobaria macaronesica*	14420	-28,77101802	38,58442165	Faial	Azores
FA1-05b	FA1	FA1-5	*Lobaria macaronesica*	14262	-28,7721542	38,5847934	Faial	Azores
FA1-05e	FA1	FA1-5	*Lobaria macaronesica*	14265	-28,7721542	38,5847934	Faial	Azores
FA1-08c	FA1	FA1-8	*Lobaria macaronesica*	14276	-28,77205434	38,58480059	Faial	Azores
FA1-09e	FA1	FA1-9	*Lobaria macaronesica*	14281	-28,77205894	38,58479542	Faial	Azores
FA1-09f	FA1	FA1-9	*Lobaria macaronesica*	14282	-28,77205894	38,58479542	Faial	Azores
FA2-10f	FA2	FA2-10	*Lobaria macaronesica*	14513	-28,66021695	38,56357897	Faial	Azores
FA2-10g	FA2	FA2-10	*Lobaria macaronesica*	14514	-28,66021695	38,56357897	Faial	Azores
FA2-11a	FA2	FA2-11	*Lobaria macaronesica*	14515	-28,66025081	38,56358416	Faial	Azores
FA2-11b	FA2	FA2-11	*Lobaria macaronesica*	14516	-28,66025081	38,56358416	Faial	Azores
FA2-11c	FA2	FA2-11	*Lobaria macaronesica*	14517	-28,66025081	38,56358416	Faial	Azores
FA2-11d	FA2	FA2-11	*Lobaria macaronesica*	14518	-28,66025081	38,56358416	Faial	Azores
FA2-12g	FA2	FA2-12	*Lobaria macaronesica*	14529	-28,66045833	38,5635996	Faial	Azores
FA2-12i	FA2	FA2-12	*Lobaria macaronesica*	14531	-28,66045833	38,5635996	Faial	Azores
FA2-13a	FA2	FA2-13	*Lobaria macaronesica*	14532	-28,66044798	38,56358884	Faial	Azores

ID	Pop	PopTree	Species	VoucherID	X	Y	Location	Area
FA2-13b	FA2	FA2-13	*Lobaria macaronesica*	14533	-28,66044798	38,56358884	Faial	Azores
FA2-13c	FA2	FA2-13	*Lobaria macaronesica*	14534	-28,66044798	38,56358884	Faial	Azores
FA2-13d	FA2	FA2-13	*Lobaria macaronesica*	14535	-28,66044798	38,56358884	Faial	Azores
FA2-14a	FA2	FA2-14	*Lobaria macaronesica*	14536	-28,66039094	38,56355866	Faial	Azores
FA2-14b	FA2	FA2-14	*Lobaria macaronesica*	14537	-28,66039094	38,56355866	Faial	Azores
FA2-14c	FA2	FA2-14	*Lobaria macaronesica*	14538	-28,66039094	38,56355866	Faial	Azores
FA2-15a	FA2	FA2-15	*Lobaria macaronesica*	14539	-28,66021883	38,5635611	Faial	Azores
FA2-16c	FA2	FA2-16	*Lobaria macaronesica*	14542	-28,66013312	38,563587	Faial	Azores
FA2-16d	FA2	FA2-16	*Lobaria macaronesica*	14543	-28,66013312	38,563587	Faial	Azores
FA2-16e	FA2	FA2-16	*Lobaria macaronesica*	14544	-28,66013312	38,563587	Faial	Azores
FA2-16f	FA2	FA2-16	*Lobaria macaronesica*	14545	-28,66013312	38,563587	Faial	Azores
FA2-17b	FA2	FA2-17	*Lobaria macaronesica*	14549	-28,66011025	38,56358544	Faial	Azores
FA2-17c	FA2	FA2-17	*Lobaria macaronesica*	14550	-28,66011025	38,56358544	Faial	Azores
FA2-17d	FA2	FA2-17	*Lobaria macaronesica*	14551	-28,66011025	38,56358544	Faial	Azores
FA2-17e	FA2	FA2-17	*Lobaria macaronesica*	14552	-28,66011025	38,56358544	Faial	Azores
FA2-18a	FA2	FA2-18	*Lobaria macaronesica*	14553	-28,66011476	38,56357708	Faial	Azores
FA2-18c	FA2	FA2-18	*Lobaria macaronesica*	14555	-28,66011476	38,56357708	Faial	Azores
FA2-19a	FA2	FA2-19	*Lobaria macaronesica*	14556	-28,65988526	38,56357403	Faial	Azores
FA2-19b	FA2	FA2-19	*Lobaria macaronesica*	14557	-28,65988526	38,56357403	Faial	Azores
FA2-19c	FA2	FA2-19	*Lobaria macaronesica*	14558	-28,65988526	38,56357403	Faial	Azores
FA2-02e	FA2	FA2-2	*Lobaria macaronesica*	14436	-28,65997279	38,56355215	Faial	Azores
FA2-02e1	FA2	FA2-2	*Lobaria macaronesica*	14436	-28,65997279	38,56355215	Faial	Azores
FA2-02f	FA2	FA2-2	*Lobaria macaronesica*	14437	-28,65997279	38,56355215	Faial	Azores
FA2-02f1	FA2	FA2-2	*Lobaria macaronesica*	14437	-28,65997279	38,56355215	Faial	Azores
FA2-02g	FA2	FA2-2	*Lobaria macaronesica*	14438	-28,65997279	38,56355215	Faial	Azores
FA2-02g1	FA2	FA2-2	*Lobaria macaronesica*	14438	-28,65997279	38,56355215	Faial	Azores
FA2-02h	FA2	FA2-2	*Lobaria macaronesica*	14439	-28,65997279	38,56355215	Faial	Azores

ID	Pop	PopTree	Species	VoucherID	X	Y	Location	Area
FA2-02h1	FA2	FA2-2	*Lobaria macaronesica*	14439	-28,65997279	38,56355215	Faial	Azores
FA2-02i	FA2	FA2-2	*Lobaria macaronesica*	14440	-28,65997279	38,56355215	Faial	Azores
FA2-02i1	FA2	FA2-2	*Lobaria macaronesica*	14440	-28,65997279	38,56355215	Faial	Azores
FA2-02j	FA2	FA2-2	*Lobaria macaronesica*	14441	-28,65997279	38,56355215	Faial	Azores
FA2-02j1	FA2	FA2-2	*Lobaria macaronesica*	14441	-28,65997279	38,56355215	Faial	Azores
FA2-22b	FA2	FA2-22	*Lobaria macaronesica*	14561	-28,65969564	38,56362808	Faial	Azores
FA2-03d	FA2	FA2-3	*Lobaria macaronesica*	14445	-28,65998386	38,56354965	Faial	Azores
FA2-03d1	FA2	FA2-3	*Lobaria macaronesica*	14445	-28,65998386	38,56354965	Faial	Azores
FA2-03f	FA2	FA2-3	*Lobaria macaronesica*	14447	-28,65998386	38,56354965	Faial	Azores
FA2-04d	FA2	FA2-4	*Lobaria macaronesica*	14452	-28,66000579	38,56355493	Faial	Azores
FA2-04j	FA2	FA2-4	*Lobaria macaronesica*	14458	-28,66000579	38,56355493	Faial	Azores
FA2-04k	FA2	FA2-4	*Lobaria macaronesica*	14459	-28,66000579	38,56355493	Faial	Azores
FA2-04l	FA2	FA2-4	*Lobaria macaronesica*	14460	-28,66000579	38,56355493	Faial	Azores
FA2-04m	FA2	FA2-4	*Lobaria macaronesica*	14461	-28,66000579	38,56355493	Faial	Azores
FA2-05b	FA2	FA2-5	*Lobaria macaronesica*	14463	-28,66003446	38,5635538	Faial	Azores
FA2-05d	FA2	FA2-5	*Lobaria macaronesica*	14465	-28,66003446	38,5635538	Faial	Azores
FA2-05e	FA2	FA2-5	*Lobaria macaronesica*	14466	-28,66003446	38,5635538	Faial	Azores
FA2-05f	FA2	FA2-5	*Lobaria macaronesica*	14467	-28,66003446	38,5635538	Faial	Azores
FA2-06e	FA2	FA2-6	*Lobaria macaronesica*	14472	-28,66004375	38,56354844	Faial	Azores
FA2-06f	FA2	FA2-6	*Lobaria macaronesica*	14473	-28,66004375	38,56354844	Faial	Azores
FA2-06h	FA2	FA2-6	*Lobaria macaronesica*	14475	-28,66004375	38,56354844	Faial	Azores
FA2-06i	FA2	FA2-6	*Lobaria macaronesica*	14476	-28,66004375	38,56354844	Faial	Azores
FA2-06k	FA2	FA2-6	*Lobaria macaronesica*	14478	-28,66004375	38,56354844	Faial	Azores
FA2-07a	FA2	FA2-7	*Lobaria macaronesica*	14479	-28,660155	38,5635702	Faial	Azores
FA2-07b	FA2	FA2-7	*Lobaria macaronesica*	14480	-28,660155	38,5635702	Faial	Azores
FA2-07c	FA2	FA2-7	*Lobaria macaronesica*	14481	-28,660155	38,5635702	Faial	Azores
FA2-07d	FA2	FA2-7	*Lobaria macaronesica*	14482	-28,660155	38,5635702	Faial	Azores

ID	Pop	PopTree	Species	VoucherID	X	Y	Location	Area
FA2-08a	FA2	FA2-8	*Lobaria macaronesica*	14487	-28,66016076	38,56357093	Faial	Azores
FA2-08b	FA2	FA2-8	*Lobaria macaronesica*	14488	-28,66016076	38,56357093	Faial	Azores
FA2-08c	FA2	FA2-8	*Lobaria macaronesica*	14489	-28,66016076	38,56357093	Faial	Azores
FA2-08d	FA2	FA2-8	*Lobaria macaronesica*	14490	-28,66016076	38,56357093	Faial	Azores
FA2-08e	FA2	FA2-8	*Lobaria macaronesica*	14491	-28,66016076	38,56357093	Faial	Azores
FA2-09a	FA2	FA2-9	*Lobaria macaronesica*	14495	-28,66019407	38,5635774	Faial	Azores
FA2-09l	FA2	FA2-9	*Lobaria macaronesica*	14506	-28,66019407	38,5635774	Faial	Azores
FA2-09m	FA2	FA2-9	*Lobaria macaronesica*	14507	-28,66019407	38,5635774	Faial	Azores
FA3-01b	FA3	FA3-1	*Lobaria macaronesica*	14565	-28,707	38,61661	Faial	Azores
FA3-01c	FA3	FA3-1	*Lobaria macaronesica*	14566	-28,707	38,61661	Faial	Azores
FA3-01d	FA3	FA3-1	*Lobaria macaronesica*	14567	-28,707	38,61661	Faial	Azores
FA3-01e	FA3	FA3-1	*Lobaria macaronesica*	14568	-28,707	38,61661	Faial	Azores
FA3-01f	FA3	FA3-1	*Lobaria macaronesica*	14569	-28,707	38,61661	Faial	Azores
FA3-02a	FA3	FA3-2	*Lobaria macaronesica*	14570	-28,70696739	38,61666764	Faial	Azores
FA3-02b	FA3	FA3-2	*Lobaria macaronesica*	14571	-28,70696739	38,61666764	Faial	Azores
FA3-03a	FA3	FA3-3	*Lobaria macaronesica*	14572	-28,70696364	38,61684781	Faial	Azores
FA3-03b	FA3	FA3-3	*Lobaria macaronesica*	14573	-28,70696364	38,61684781	Faial	Azores
FA3-03c	FA3	FA3-3	*Lobaria macaronesica*	14574	-28,70696364	38,61684781	Faial	Azores
FA3-03d	FA3	FA3-3	*Lobaria macaronesica*	14575	-28,70696364	38,61684781	Faial	Azores
FA3-03e	FA3	FA3-3	*Lobaria macaronesica*	14576	-28,70696364	38,61684781	Faial	Azores
FA3-03f	FA3	FA3-3	*Lobaria macaronesica*	14577	-28,70696364	38,61684781	Faial	Azores
FA3-03g	FA3	FA3-3	*Lobaria macaronesica*	14578	-28,70696364	38,61684781	Faial	Azores
FA3-03h	FA3	FA3-3	*Lobaria macaronesica*	14579	-28,70696364	38,61684781	Faial	Azores
FA3-03i	FA3	FA3-3	*Lobaria macaronesica*	14580	-28,70696364	38,61684781	Faial	Azores
FA3-03k	FA3	FA3-3	*Lobaria macaronesica*	14582	-28,70696364	38,61684781	Faial	Azores
FA3-03l	FA3	FA3-3	*Lobaria macaronesica*	14583	-28,70696364	38,61684781	Faial	Azores
FA3-06a	FA3	FA3-6	*Lobaria macaronesica*	14596	-28,70041	38,61265	Faial	Azores

ID	Pop	PopTree	Species	VoucherID	X	Y	Location	Area
FA3-06b	FA3	FA3-6	*Lobaria macaronesica*	14597	-28,70041	38,61265	Faial	Azores
FA3-06c	FA3	FA3-6	*Lobaria macaronesica*	14598	-28,70041	38,61265	Faial	Azores
FA3-07a	FA3	FA3-7	*Lobaria macaronesica*	14599	-28,70019984	38,61249657	Faial	Azores
FA3-07c	FA3	FA3-7	*Lobaria macaronesica*	14601	-28,70019984	38,61249657	Faial	Azores
FA3-08a	FA3	FA3-8	*Lobaria macaronesica*	14602	-28,70025391	38,61255483	Faial	Azores
FA3-08b	FA3	FA3-8	*Lobaria macaronesica*	14603	-28,70025391	38,61255483	Faial	Azores
FA3-08c	FA3	FA3-8	*Lobaria macaronesica*	14604	-28,70025391	38,61255483	Faial	Azores
FA3-08d	FA3	FA3-8	*Lobaria macaronesica*	14605	-28,70025391	38,61255483	Faial	Azores
FR1-01a	FR1	FR1-1	*Lobaria macaronesica*	15159	-31,164153	39,44671	Flores	Azores
FR1-10a	FR1	FR1-10	*Lobaria macaronesica*	15205	-31,16397096	39,44664916	Flores	Azores
FR1-10b	FR1	FR1-10	*Lobaria macaronesica*	15206	-31,16397096	39,44664916	Flores	Azores
FR1-10c	FR1	FR1-10	*Lobaria macaronesica*	15207	-31,16397096	39,44664916	Flores	Azores
FR1-10d	FR1	FR1-10	*Lobaria macaronesica*	15208	-31,16397096	39,44664916	Flores	Azores
FR1-12a	FR1	FR1-12	*Lobaria macaronesica*	15212	-31,16394429	39,44673594	Flores	Azores
FR1-15a	FR1	FR1-15	*Lobaria macaronesica*	15221	-31,1634445	39,44668727	Flores	Azores
FR1-15b	FR1	FR1-15	*Lobaria macaronesica*	15222	-31,1634445	39,44668727	Flores	Azores
FR1-15c	FR1	FR1-15	*Lobaria macaronesica*	15223	-31,1634445	39,44668727	Flores	Azores
FR1-15d	FR1	FR1-15	*Lobaria macaronesica*	15224	-31,1634445	39,44668727	Flores	Azores
FR1-15e	FR1	FR1-15	*Lobaria macaronesica*	15225	-31,1634445	39,44668727	Flores	Azores
FR1-15f	FR1	FR1-15	*Lobaria macaronesica*	15226	-31,1634445	39,44668727	Flores	Azores
FR1-16a	FR1	FR1-16	*Lobaria macaronesica*	15227	-31,16408215	39,44646888	Flores	Azores
FR1-19c	FR1	FR1-19	*Lobaria macaronesica*	15239	-31,1641172	39,44648722	Flores	Azores
FR1-19d	FR1	FR1-19	*Lobaria macaronesica*	15240	-31,1641172	39,44648722	Flores	Azores
FR1-20c	FR1	FR1-20	*Lobaria macaronesica*	15245	-31,16415971	39,44647266	Flores	Azores
FR1-20e	FR1	FR1-20	*Lobaria macaronesica*	15247	-31,16415971	39,44647266	Flores	Azores
FR1-20f	FR1	FR1-20	*Lobaria macaronesica*	15248	-31,16415971	39,44647266	Flores	Azores
FR1-06b	FR1	FR1-6	*Lobaria macaronesica*	15188	-31,16412267	39,44673033	Flores	Azores

ID	Pop	PopTree	Species	VoucherID	X	Y	Location	Area
FR1-06c	FR1	FR1-6	*Lobaria macaronesica*	15189	-31,16412267	39,44673033	Flores	Azores
FR1-07c	FR1	FR1-7	*Lobaria macaronesica*	15193	-31,16412267	39,44673033	Flores	Azores
FR1-08d	FR1	FR1-8	*Lobaria macaronesica*	15197	-31,16408769	39,44670661	Flores	Azores
FR1-08h	FR1	FR1-8	*Lobaria macaronesica*	15201	-31,16408769	39,44670661	Flores	Azores
FR2-01a	FR2	FR2-1	*Lobaria macaronesica*	15253	-31,251258	39,448117	Flores	Azores
FR2-01b	FR2	FR2-1	*Lobaria macaronesica*	15254	-31,251258	39,448117	Flores	Azores
FR2-01c	FR2	FR2-1	*Lobaria macaronesica*	15255	-31,251258	39,448117	Flores	Azores
FR2-01d	FR2	FR2-1	*Lobaria macaronesica*	15256	-31,251258	39,448117	Flores	Azores
FR2-01e	FR2	FR2-1	*Lobaria macaronesica*	15257	-31,251258	39,448117	Flores	Azores
FR2-10a	FR2	FR2-10	*Lobaria macaronesica*	15289	-31,25146236	39,44684673	Flores	Azores
FR2-10b	FR2	FR2-10	*Lobaria macaronesica*	15290	-31,25146236	39,44684673	Flores	Azores
FR2-10c	FR2	FR2-10	*Lobaria macaronesica*	15291	-31,25146236	39,44684673	Flores	Azores
FR2-10d	FR2	FR2-10	*Lobaria macaronesica*	15292	-31,25146236	39,44684673	Flores	Azores
FR2-10e	FR2	FR2-10	*Lobaria macaronesica*	15293	-31,25146236	39,44684673	Flores	Azores
FR2-11a	FR2	FR2-11	*Lobaria macaronesica*	15294	-31,25145655	39,44684664	Flores	Azores
FR2-12a	FR2	FR2-12	*Lobaria macaronesica*	15295	-31,2515222	39,44690975	Flores	Azores
FR2-12b	FR2	FR2-12	*Lobaria macaronesica*	15296	-31,2515222	39,44690975	Flores	Azores
FR2-13a	FR2	FR2-13	*Lobaria macaronesica*	15297	-31,25142816	39,44669667	Flores	Azores
FR2-13b	FR2	FR2-13	*Lobaria macaronesica*	15298	-31,25142816	39,44669667	Flores	Azores
FR2-13d	FR2	FR2-13	*Lobaria macaronesica*	15300	-31,25142816	39,44669667	Flores	Azores
FR2-14a	FR2	FR2-14	*Lobaria macaronesica*	15302	-31,25108965	39,4459296	Flores	Azores
FR2-15a	FR2	FR2-15	*Lobaria macaronesica*	15305	-31,25105318	39,44589454	Flores	Azores
FR2-15b	FR2	FR2-15	*Lobaria macaronesica*	15306	-31,25105318	39,44589454	Flores	Azores
FR2-15c	FR2	FR2-15	*Lobaria macaronesica*	15307	-31,25105318	39,44589454	Flores	Azores
FR2-16a	FR2	FR2-16	*Lobaria macaronesica*	15308	-31,25097263	39,44582961	Flores	Azores
FR2-16b	FR2	FR2-16	*Lobaria macaronesica*	15309	-31,25097263	39,44582961	Flores	Azores
FR2-16c	FR2	FR2-16	*Lobaria macaronesica*	15310	-31,25097263	39,44582961	Flores	Azores

**ID**	**Pop**	**PopTree**	**Species**	**VoucherID**	**X**	**Y**	**Location**	**Area**
FR2-16d	FR2	FR2-16	*Lobaria macaronesica*	15311	-31,25097263	39,44582961	Flores	Azores
FR2-17a	FR2	FR2-17	*Lobaria macaronesica*	15312	-31,25095468	39,44574062	Flores	Azores
FR2-17b	FR2	FR2-17	*Lobaria macaronesica*	15313	-31,25095468	39,44574062	Flores	Azores
FR2-17c	FR2	FR2-17	*Lobaria macaronesica*	15314	-31,25095468	39,44574062	Flores	Azores
FR2-18a	FR2	FR2-18	*Lobaria macaronesica*	15315	-31,25092229	39,44570323	Flores	Azores
FR2-18b	FR2	FR2-18	*Lobaria macaronesica*	15316	-31,25092229	39,44570323	Flores	Azores
FR2-18d	FR2	FR2-18	*Lobaria macaronesica*	15318	-31,25092229	39,44570323	Flores	Azores
FR2-19a	FR2	FR2-19	*Lobaria macaronesica*	15319	-31,25014491	39,44480588	Flores	Azores
FR2-19b	FR2	FR2-19	*Lobaria macaronesica*	15320	-31,25014491	39,44480588	Flores	Azores
FR2-19c	FR2	FR2-19	*Lobaria macaronesica*	15321	-31,25014491	39,44480588	Flores	Azores
FR2-19d	FR2	FR2-19	*Lobaria macaronesica*	15322	-31,25014491	39,44480588	Flores	Azores
FR2-19e	FR2	FR2-19	*Lobaria macaronesica*	15323	-31,25014491	39,44480588	Flores	Azores
FR2-19f	FR2	FR2-19	*Lobaria macaronesica*	15324	-31,25014491	39,44480588	Flores	Azores
FR2-02a	FR2	FR2-2	*Lobaria macaronesica*	15259	-31,25126946	39,44810682	Flores	Azores
FR2-02c	FR2	FR2-2	*Lobaria macaronesica*	15261	-31,25126946	39,44810682	Flores	Azores
FR2-02d	FR2	FR2-2	*Lobaria macaronesica*	15262	-31,25126946	39,44810682	Flores	Azores
FR2-02e	FR2	FR2-2	*Lobaria macaronesica*	15263	-31,25126946	39,44810682	Flores	Azores
FR2-20a	FR2	FR2-20	*Lobaria macaronesica*	15325	-31,2501308	39,44478117	Flores	Azores
FR2-20b	FR2	FR2-20	*Lobaria macaronesica*	15326	-31,2501308	39,44478117	Flores	Azores
FR2-03a	FR2	FR2-3	*Lobaria macaronesica*	15265	-31,25128595	39,44810245	Flores	Azores
FR2-03b	FR2	FR2-3	*Lobaria macaronesica*	15266	-31,25128595	39,44810245	Flores	Azores
FR2-03c	FR2	FR2-3	*Lobaria macaronesica*	15267	-31,25128595	39,44810245	Flores	Azores
FR2-03d	FR2	FR2-3	*Lobaria macaronesica*	15268	-31,25128595	39,44810245	Flores	Azores
FR2-03e	FR2	FR2-3	*Lobaria macaronesica*	15269	-31,25128595	39,44810245	Flores	Azores
FR2-04a	FR2	FR2-4	*Lobaria macaronesica*	15270	-31,25133187	39,44809688	Flores	Azores
FR2-04b	FR2	FR2-4	*Lobaria macaronesica*	15271	-31,25133187	39,44809688	Flores	Azores
FR2-04c	FR2	FR2-4	*Lobaria macaronesica*	15272	-31,25133187	39,44809688	Flores	Azores

ID	Pop	PopTree	Species	VoucherID	X	Y	Location	Area
FR2-04d	FR2	FR2-4	*Lobaria macaronesica*	15273	-31,25133187	39,44809688	Flores	Azores
FR2-05a	FR2	FR2-5	*Lobaria macaronesica*	15274	-31,25110619	39,44758549	Flores	Azores
FR2-05b	FR2	FR2-5	*Lobaria macaronesica*	15275	-31,25110619	39,44758549	Flores	Azores
FR2-05c	FR2	FR2-5	*Lobaria macaronesica*	15276	-31,25110619	39,44758549	Flores	Azores
FR2-06a	FR2	FR2-6	*Lobaria macaronesica*	15277	-31,25127837	39,44756462	Flores	Azores
FR2-06b	FR2	FR2-6	*Lobaria macaronesica*	15278	-31,25127837	39,44756462	Flores	Azores
FR2-06c	FR2	FR2-6	*Lobaria macaronesica*	15279	-31,25127837	39,44756462	Flores	Azores
FR2-07a	FR2	FR2-7	*Lobaria macaronesica*	15280	-31,25128285	39,44754694	Flores	Azores
FR2-07b	FR2	FR2-7	*Lobaria macaronesica*	15281	-31,25128285	39,44754694	Flores	Azores
FR2-07c	FR2	FR2-7	*Lobaria macaronesica*	15282	-31,25128285	39,44754694	Flores	Azores
FR2-08c	FR2	FR2-8	*Lobaria macaronesica*	15285	-31,25140292	39,44739269	Flores	Azores
FR2-09a	FR2	FR2-9	*Lobaria macaronesica*	15286	-31,25144884	39,44738712	Flores	Azores
FR2-09b	FR2	FR2-9	*Lobaria macaronesica*	15287	-31,25144884	39,44738712	Flores	Azores
FR2-09c	FR2	FR2-9	*Lobaria macaronesica*	15288	-31,25144884	39,44738712	Flores	Azores
FR3-11a	FR3	FR3-11	*Lobaria macaronesica*	15373	-31,15405975	39,4473737	Flores	Azores
FR3-13a	FR3	FR3-13	*Lobaria macaronesica*	15381	-31,15404937	39,4473797	Flores	Azores
FR3-13b	FR3	FR3-13	*Lobaria macaronesica*	15382	-31,15404937	39,4473797	Flores	Azores
FR3-13c	FR3	FR3-13	*Lobaria macaronesica*	15383	-31,15404937	39,4473797	Flores	Azores
FR3-15c	FR3	FR3-15	*Lobaria macaronesica*	15392	-31,15402924	39,44736449	Flores	Azores
FR3-16d	FR3	FR3-16	*Lobaria macaronesica*	15396	-31,15400216	39,44733927	Flores	Azores
FR3-16e	FR3	FR3-16	*Lobaria macaronesica*	15397	-31,15400216	39,44733927	Flores	Azores
FR3-03b	FR3	FR3-3	*Lobaria macaronesica*	15341	-31,15415085	39,44726703	Flores	Azores
FR3-03d	FR3	FR3-3	*Lobaria macaronesica*	15343	-31,15415085	39,44726703	Flores	Azores
FR3-03e	FR3	FR3-3	*Lobaria macaronesica*	15344	-31,15412987	39,44730903	Flores	Azores
FR3-03f	FR3	FR3-3	*Lobaria macaronesica*	15345	-31,15412987	39,44730903	Flores	Azores
FR3-03g	FR3	FR3-3	*Lobaria macaronesica*	15346	-31,15412987	39,44730903	Flores	Azores
FR3-05c	FR3	FR3-5	*Lobaria macaronesica*	15355	-31,1541071	39,4473491	Flores	Azores

ID	Pop	PopTree	Species	VoucherID	X	Y	Location	Area
FR3-06b	FR3	FR3-6	*Lobaria macaronesica*	15357	-31,15411627	39,44734097	Flores	Azores
FR3-07a	FR3	FR3-7	*Lobaria macaronesica*	15359	-31,15411177	39,44735864	Flores	Azores
MA1-04a	MA1	MA1-04	*Lobaria macaronesica*	25462	36,9724	-25,08885	Santa Maria	Azores
MA1-04c	MA1	MA1-04	*Lobaria macaronesica*	25464	36,9724	-25,08885	Santa Maria	Azores
MA1-04d	MA1	MA1-04	*Lobaria macaronesica*	25465	36,9724	-25,08885	Santa Maria	Azores
MA1-04e	MA1	MA1-04	*Lobaria macaronesica*	25466	36,9724	-25,08885	Santa Maria	Azores
MA1-05c	MA1	MA1-05	*Lobaria macaronesica*	25469	36,9675	-25,08493333	Santa Maria	Azores
MA1-05g	MA1	MA1-05	*Lobaria macaronesica*	25473	36,9675	-25,08493333	Santa Maria	Azores
MA1-06c	MA1	MA1-06	*Lobaria macaronesica*	25476	36,96749008	-25,08491599	Santa Maria	Azores
MA1-06d	MA1	MA1-06	*Lobaria macaronesica*	25477	36,96749008	-25,08491599	Santa Maria	Azores
MA1-06e	MA1	MA1-06	*Lobaria macaronesica*	25478	36,96749008	-25,08491599	Santa Maria	Azores
MA1-06f	MA1	MA1-06	*Lobaria macaronesica*	25479	36,96749008	-25,08491599	Santa Maria	Azores
MA1-06l	MA1	MA1-06	*Lobaria macaronesica*	25485	36,96749008	-25,08491599	Santa Maria	Azores
MA1-07a	MA1	MA1-07	*Lobaria macaronesica*	25486	36,96748972	-25,08489354	Santa Maria	Azores
MA1-07b	MA1	MA1-07	*Lobaria macaronesica*	25487	36,96748972	-25,08489354	Santa Maria	Azores
MA1-07c	MA1	MA1-07	*Lobaria macaronesica*	25488	36,96748972	-25,08489354	Santa Maria	Azores
MA1-07g	MA1	MA1-07	*Lobaria macaronesica*	25492	36,96748972	-25,08489354	Santa Maria	Azores
MA1-07h	MA1	MA1-07	*Lobaria macaronesica*	25495	36,96748972	-25,08489354	Santa Maria	Azores
MA1-07i	MA1	MA1-07	*Lobaria macaronesica*	25496	36,96748972	-25,08489354	Santa Maria	Azores
MA1-07k	MA1	MA1-07	*Lobaria macaronesica*	31771	36,96748972	-25,08489354	Santa Maria	Azores
MA2-01f	MA2	MA2-01	*Lobaria macaronesica*	25505	36,9836	-25,0904	Santa Maria	Azores
MA2-01l	MA2	MA2-01	*Lobaria macaronesica*	25511	36,9836	-25,0904	Santa Maria	Azores
MA2-02d	MA2	MA2-02	*Lobaria macaronesica*	25515	36,98358333	-25,0904	Santa Maria	Azores
MA2-02e	MA2	MA2-02	*Lobaria macaronesica*	25516	36,98358333	-25,0904	Santa Maria	Azores
MA2-02i	MA2	MA2-02	*Lobaria macaronesica*	25520	36,98358333	-25,0904	Santa Maria	Azores
MA2-02l	MA2	MA2-02	*Lobaria macaronesica*	25523	36,98358333	-25,0904	Santa Maria	Azores
MA2-02m	MA2	MA2-02	*Lobaria macaronesica*	25524	36,98358333	-25,0904	Santa Maria	Azores

ID	Pop	PopTree	Species	VoucherID	X	Y	Location	Area
MA2-03b	MA2	MA2-03	*Lobaria macaronesica*	25528	36,98358333	-25,09043333	Santa Maria	Azores
MA2-03c	MA2	MA2-03	*Lobaria macaronesica*	25529	36,98358333	-25,09043333	Santa Maria	Azores
MA2-03i	MA2	MA2-03	*Lobaria macaronesica*	25535	36,98358333	-25,09043333	Santa Maria	Azores
MA2-03j	MA2	MA2-03	*Lobaria macaronesica*	25536	36,98358333	-25,09043333	Santa Maria	Azores
MA2-10b	MA2	MA2-10	*Lobaria macaronesica*	25602	-25,09046667	36,98355	Santa Maria	Azores
MA2-10c	MA2	MA2-10	*Lobaria macaronesica*	25603	-25,09046667	36,98355	Santa Maria	Azores
MA2-10d	MA2	MA2-10	*Lobaria macaronesica*	25604	-25,09046667	36,98355	Santa Maria	Azores
MA2-10e	MA2	MA2-10	*Lobaria macaronesica*	25605	-25,09046667	36,98355	Santa Maria	Azores
MA2-10f	MA2	MA2-10	*Lobaria macaronesica*	25606	-25,09046667	36,98355	Santa Maria	Azores
MA2-10g	MA2	MA2-10	*Lobaria macaronesica*	25607	-25,09046667	36,98355	Santa Maria	Azores
MA2-10i	MA2	MA2-10	*Lobaria macaronesica*	25609	-25,09046667	36,98355	Santa Maria	Azores
MA2-10j	MA2	MA2-10	*Lobaria macaronesica*	25610	-25,09046667	36,98355	Santa Maria	Azores
MA2-10m	MA2	MA2-10	*Lobaria macaronesica*	25613	-25,09046667	36,98355	Santa Maria	Azores
MA2-04c	MA2	MA2-4	*Lobaria macaronesica*	25539	-25,09011667	36,98396667	Santa Maria	Azores
MA2-04e	MA2	MA2-4	*Lobaria macaronesica*	25541	-25,09011667	36,98396667	Santa Maria	Azores
MA2-04h	MA2	MA2-4	*Lobaria macaronesica*	25544	-25,09011667	36,98396667	Santa Maria	Azores
MA2-05c	MA2	MA2-5	*Lobaria macaronesica*	25549	-25,09028333	36,98371667	Santa Maria	Azores
MA2-05h	MA2	MA2-5	*Lobaria macaronesica*	25554	-25,09028333	36,98371667	Santa Maria	Azores
MA2-05i	MA2	MA2-5	*Lobaria macaronesica*	25555	-25,09028333	36,98371667	Santa Maria	Azores
MA2-05j	MA2	MA2-5	*Lobaria macaronesica*	25556	-25,09028333	36,98371667	Santa Maria	Azores
MA2-05k	MA2	MA2-5	*Lobaria macaronesica*	25557	-25,09028333	36,98371667	Santa Maria	Azores
MA2-06b	MA2	MA2-6	*Lobaria macaronesica*	25559	-25,09038333	36,98356667	Santa Maria	Azores
MA2-06c	MA2	MA2-6	*Lobaria macaronesica*	25560	-25,09038333	36,98356667	Santa Maria	Azores
MA2-06e	MA2	MA2-6	*Lobaria macaronesica*	25562	-25,09038333	36,98356667	Santa Maria	Azores
MA2-06f	MA2	MA2-6	*Lobaria macaronesica*	25563	-25,09038333	36,98356667	Santa Maria	Azores
MA2-06g	MA2	MA2-6	*Lobaria macaronesica*	25564	-25,09038333	36,98356667	Santa Maria	Azores
MA2-06h	MA2	MA2-6	*Lobaria macaronesica*	25565	-25,09038333	36,98356667	Santa Maria	Azores

ID	Pop	PopTree	Species	VoucherID	X	Y	Location	Area
MA2-06i	MA2	MA2-6	*Lobaria macaronesica*	25566	-25,09038333	36,98356667	Santa Maria	Azores
MA2-07b	MA2	MA2-7	*Lobaria macaronesica*	25570	-25,0904	36,98355	Santa Maria	Azores
MA2-07d	MA2	MA2-7	*Lobaria macaronesica*	25572	-25,0904	36,98355	Santa Maria	Azores
MA2-07e	MA2	MA2-7	*Lobaria macaronesica*	25573	-25,0904	36,98355	Santa Maria	Azores
MA2-07g	MA2	MA2-7	*Lobaria macaronesica*	25575	-25,0904	36,98355	Santa Maria	Azores
MA2-07h	MA2	MA2-7	*Lobaria macaronesica*	25576	-25,0904	36,98355	Santa Maria	Azores
MA2-08a	MA2	MA2-8	*Lobaria macaronesica*	25579	-25,09043333	36,98351667	Santa Maria	Azores
MA2-08b	MA2	MA2-8	*Lobaria macaronesica*	25580	-25,09043333	36,98351667	Santa Maria	Azores
MA2-08c	MA2	MA2-8	*Lobaria macaronesica*	25581	-25,09043333	36,98351667	Santa Maria	Azores
MA2-08h	MA2	MA2-8	*Lobaria macaronesica*	25586	-25,09043333	36,98351667	Santa Maria	Azores
MA2-08i	MA2	MA2-8	*Lobaria macaronesica*	25587	-25,09043333	36,98351667	Santa Maria	Azores
MA2-08j	MA2	MA2-8	*Lobaria macaronesica*	25588	-25,09043333	36,98351667	Santa Maria	Azores
MA2-08k	MA2	MA2-8	*Lobaria macaronesica*	25589	-25,09043333	36,98351667	Santa Maria	Azores
MA2-09a	MA2	MA2-9	*Lobaria macaronesica*	25591	-25,09048333	36,9835	Santa Maria	Azores
MA2-09b	MA2	MA2-9	*Lobaria macaronesica*	25592	-25,09048333	36,9835	Santa Maria	Azores
MA2-09e	MA2	MA2-9	*Lobaria macaronesica*	25595	-25,09048333	36,9835	Santa Maria	Azores
MA2-09f	MA2	MA2-9	*Lobaria macaronesica*	25596	-25,09048333	36,9835	Santa Maria	Azores
MA2-09g	MA2	MA2-9	*Lobaria macaronesica*	25597	-25,09048333	36,9835	Santa Maria	Azores
MA2-09h	MA2	MA2-9	*Lobaria macaronesica*	25598	-25,09048333	36,9835	Santa Maria	Azores
MA2-09i	MA2	MA2-9	*Lobaria macaronesica*	25599	-25,09048333	36,9835	Santa Maria	Azores
MA2-09j	MA2	MA2-9	*Lobaria macaronesica*	25600	-25,09048333	36,9835	Santa Maria	Azores
PI1-10a	PI1	PI1-10	*Lobaria macaronesica*	15107	-28,42784573	38,46798549	Pico	Azores
PI1-10b	PI1	PI1-10	*Lobaria macaronesica*	15108	-28,42784573	38,46798549	Pico	Azores
PI1-10c	PI1	PI1-10	*Lobaria macaronesica*	15109	-28,42784573	38,46798549	Pico	Azores
PI1-11a	PI1	PI1-11	*Lobaria macaronesica*	15110	-28,42783388	38,46800093	Pico	Azores
PI1-11b	PI1	PI1-11	*Lobaria macaronesica*	15111	-28,42783388	38,46800093	Pico	Azores
PI1-12e	PI1	PI1-12	*Lobaria macaronesica*	15116	-28,42782258	38,46801111	Pico	Azores

ID	Pop	PopTree	Species	VoucherID	X	Y	Location	Area
PI1-12f	PI1	PI1-12	*Lobaria macaronesica*	15117	-28,42782258	38,46801111	Pico	Azores
PI1-12g	PI1	PI1-12	*Lobaria macaronesica*	15118	-28,42782258	38,46801111	Pico	Azores
PI1-13a	PI1	PI1-13	*Lobaria macaronesica*	15119	-28,42780338	38,46800127	Pico	Azores
PI1-15a	PI1	PI1-15	*Lobaria macaronesica*	15124	-28,42770882	38,46803789	Pico	Azores
PI1-15b	PI1	PI1-15	*Lobaria macaronesica*	15125	-28,42770882	38,46803789	Pico	Azores
PI1-15c	PI1	PI1-15	*Lobaria macaronesica*	15126	-28,42770882	38,46803789	Pico	Azores
PI1-15f	PI1	PI1-15	*Lobaria macaronesica*	15129	-28,42770882	38,46803789	Pico	Azores
PI1-15h	PI1	PI1-15	*Lobaria macaronesica*	15131	-28,42770882	38,46803789	Pico	Azores
PI1-15i	PI1	PI1-15	*Lobaria macaronesica*	15132	-28,42770882	38,46803789	Pico	Azores
PI1-15j	PI1	PI1-15	*Lobaria macaronesica*	15133	-28,42770882	38,46803789	Pico	Azores
PI1-16a	PI1	PI1-16	*Lobaria macaronesica*	15134	-28,42771902	38,46804199	Pico	Azores
PI1-16b	PI1	PI1-16	*Lobaria macaronesica*	15135	-28,42771902	38,46804199	Pico	Azores
PI1-16c	PI1	PI1-16	*Lobaria macaronesica*	15136	-28,42771902	38,46804199	Pico	Azores
PI1-17a	PI1	PI1-17	*Lobaria macaronesica*	15137	-28,42772475	38,46804192	Pico	Azores
PI1-17b	PI1	PI1-17	*Lobaria macaronesica*	15138	-28,42772475	38,46804192	Pico	Azores
PI1-17c	PI1	PI1-17	*Lobaria macaronesica*	15139	-28,42772475	38,46804192	Pico	Azores
PI1-18a	PI1	PI1-18	*Lobaria macaronesica*	15140	-28,42772347	38,46803753	Pico	Azores
PI1-18b	PI1	PI1-18	*Lobaria macaronesica*	15141	-28,42772347	38,46803753	Pico	Azores
PI1-18c	PI1	PI1-18	*Lobaria macaronesica*	15142	-28,42772347	38,46803753	Pico	Azores
PI1-18d	PI1	PI1-18	*Lobaria macaronesica*	15143	-28,42772347	38,46803753	Pico	Azores
PI1-18e	PI1	PI1-18	*Lobaria macaronesica*	15144	-28,42772347	38,46803753	Pico	Azores
PI1-19a	PI1	PI1-19	*Lobaria macaronesica*	15145	-28,42773607	38,46802833	Pico	Azores
PI1-19b	PI1	PI1-19	*Lobaria macaronesica*	15146	-28,42773607	38,46802833	Pico	Azores
PI1-19c	PI1	PI1-19	*Lobaria macaronesica*	15147	-28,42773607	38,46802833	Pico	Azores
PI1-20a	PI1	PI1-20	*Lobaria macaronesica*	15148	-28,42774018	38,46798339	Pico	Azores
PI1-20b	PI1	PI1-20	*Lobaria macaronesica*	15149	-28,42774018	38,46798339	Pico	Azores
PI1-20c	PI1	PI1-20	*Lobaria macaronesica*	15150	-28,42774018	38,46798339	Pico	Azores

ID	Pop	PopTree	Species	VoucherID	X	Y	Location	Area
PI1-20d	PI1	PI1-20	*Lobaria macaronesica*	15151	-28,42774018	38,46798339	Pico	Azores
PI1-21a	PI1	PI1-21	*Lobaria macaronesica*	15152	-28,42774018	38,46798339	Pico	Azores
PI1-21b	PI1	PI1-21	*Lobaria macaronesica*	15153	-28,42774018	38,46798339	Pico	Azores
PI1-21c	PI1	PI1-21	*Lobaria macaronesica*	15154	-28,42774018	38,46798339	Pico	Azores
PI1-21d	PI1	PI1-21	*Lobaria macaronesica*	15155	-28,42774018	38,46798339	Pico	Azores
PI1-22a	PI1	PI1-22	*Lobaria macaronesica*	15156	-28,42772383	38,46794973	Pico	Azores
PI1-22b	PI1	PI1-22	*Lobaria macaronesica*	15157	-28,42772383	38,46794973	Pico	Azores
PI1-22c	PI1	PI1-22	*Lobaria macaronesica*	15158	-28,42772383	38,46794973	Pico	Azores
PI4-10a	PI4	PI4-10	*Lobaria macaronesica*	15051	-28,25329191	38,48064076	Pico	Azores
PI4-10d	PI4	PI4-10	*Lobaria macaronesica*	15054	-28,25329191	38,48064076	Pico	Azores
PI4-11a	PI4	PI4-11	*Lobaria macaronesica*	15055	-28,25328106	38,48064366	Pico	Azores
PI4-11b	PI4	PI4-11	*Lobaria macaronesica*	15056	-28,25328106	38,48064366	Pico	Azores
PI4-11e	PI4	PI4-11	*Lobaria macaronesica*	15059	-28,25328106	38,48064366	Pico	Azores
PI4-12a	PI4	PI4-12	*Lobaria macaronesica*	15061	-28,25327498	38,48064778	Pico	Azores
PI4-12b	PI4	PI4-12	*Lobaria macaronesica*	15062	-28,25327498	38,48064778	Pico	Azores
PI4-12c	PI4	PI4-12	*Lobaria macaronesica*	15063	-28,25327498	38,48064778	Pico	Azores
PI4-12e	PI4	PI4-12	*Lobaria macaronesica*	15065	-28,25327498	38,48064778	Pico	Azores
PI4-13e	PI4	PI4-13	*Lobaria macaronesica*	15070	-28,25327713	38,48065664	Pico	Azores
PI4-13f	PI4	PI4-13	*Lobaria macaronesica*	15071	-28,25327713	38,48065664	Pico	Azores
PI4-13g	PI4	PI4-13	*Lobaria macaronesica*	15072	-28,25327713	38,48065664	Pico	Azores
PI4-14d	PI4	PI4-14	*Lobaria macaronesica*	15076	-28,25327599	38,48065657	Pico	Azores
PI4-14e	PI4	PI4-14	*Lobaria macaronesica*	15077	-28,25327599	38,48065657	Pico	Azores
PI4-02a	PI4	PI4-2	*Lobaria macaronesica*	15010	-28,25331576	38,480565	Pico	Azores
PI4-02b	PI4	PI4-2	*Lobaria macaronesica*	15011	-28,25331576	38,480565	Pico	Azores
PI4-02c	PI4	PI4-2	*Lobaria macaronesica*	15012	-28,25331576	38,480565	Pico	Azores
PI4-02e	PI4	PI4-2	*Lobaria macaronesica*	15014	-28,25331576	38,480565	Pico	Azores
PI4-02g	PI4	PI4-2	*Lobaria macaronesica*	15016	-28,25331576	38,480565	Pico	Azores

ID	Pop	PopTree	Species	VoucherID	X	Y	Location	Area
PI4-02h	PI4	PI4-2	*Lobaria macaronesica*	15017	-28,25331576	38,480565	Pico	Azores
PI4-04c	PI4	PI4-4	*Lobaria macaronesica*	15025	-28,25327417	38,48060895	Pico	Azores
PI4-05a	PI4	PI4-5	*Lobaria macaronesica*	15027	-28,25326828	38,48061323	Pico	Azores
PI4-05b	PI4	PI4-5	*Lobaria macaronesica*	15028	-28,25326828	38,48061323	Pico	Azores
PI4-05d	PI4	PI4-5	*Lobaria macaronesica*	15030	-28,25326828	38,48061323	Pico	Azores
PI4-05e	PI4	PI4-5	*Lobaria macaronesica*	15031	-28,25326828	38,48061323	Pico	Azores
PI4-05f	PI4	PI4-5	*Lobaria macaronesica*	15032	-28,25326828	38,48061323	Pico	Azores
PI4-06a	PI4	PI4-6	*Lobaria macaronesica*	15034	-28,25327664	38,48061941	Pico	Azores
PI4-06b	PI4	PI4-6	*Lobaria macaronesica*	15035	-28,25327664	38,48061941	Pico	Azores
PI4-06c	PI4	PI4-6	*Lobaria macaronesica*	15036	-28,25327664	38,48061941	Pico	Azores
PI4-06d	PI4	PI4-6	*Lobaria macaronesica*	15037	-28,25327664	38,48061941	Pico	Azores
PI4-07a	PI4	PI4-7	*Lobaria macaronesica*	15038	-28,25327271	38,48062269	Pico	Azores
PI4-07b	PI4	PI4-7	*Lobaria macaronesica*	15039	-28,25327271	38,48062269	Pico	Azores
PI4-08c	PI4	PI4-8	*Lobaria macaronesica*	15043	-28,25327196	38,48062439	Pico	Azores
PI4-09a	PI4	PI4-9	*Lobaria macaronesica*	15046	-28,2532887	38,48062749	Pico	Azores
PI4-09d	PI4	PI4-9	*Lobaria macaronesica*	15049	-28,2532887	38,48062749	Pico	Azores
PM10-02d	PM10	PM10-2	*Lobaria macaronesica*	12410	-16,87622235	32,7631912	Madeira	Madeira
PM10-03a	PM10	PM10-3	*Lobaria macaronesica*	12411	-16,87627571	32,7631904	Madeira	Madeira
PM10-03b	PM10	PM10-3	*Lobaria macaronesica*	12412	-16,87627571	32,7631904	Madeira	Madeira
PM10-03c	PM10	PM10-3	*Lobaria macaronesica*	12413	-16,87627571	32,7631904	Madeira	Madeira
PM10-05c	PM10	PM10-5	*Lobaria macaronesica*	12421	-16,87638241	32,76318881	Madeira	Madeira
PM10-05d	PM10	PM10-5	*Lobaria macaronesica*	12422	-16,87638241	32,76318881	Madeira	Madeira
PM10-05e	PM10	PM10-5	*Lobaria macaronesica*	12423	-16,87638241	32,76318881	Madeira	Madeira
PM10-07a	PM10	PM10-7	*Lobaria macaronesica*	12427	-16,87648912	32,76318721	Madeira	Madeira
PM10-07b	PM10	PM10-7	*Lobaria macaronesica*	12428	-16,87648912	32,76318721	Madeira	Madeira
PM10-07c	PM10	PM10-7	*Lobaria macaronesica*	12429	-16,87648912	32,76318721	Madeira	Madeira
PM11-10b	PM11	PM11-10	*Lobaria macaronesica*	12458	-16,88215017	32,73867761	Madeira	Madeira

ID	Pop	PopTree	Species	VoucherID	X	Y	Location	Area
PM11-15c	PM11	PM11-15	*Lobaria macaronesica*	12482	-16,88136431	32,73844234	Madeira	Madeira
PM11-02c	PM11	PM11-2	*Lobaria macaronesica*	12440	-16,88288522	32,73841378	Madeira	Madeira
PM12-10d	PM12	PM12-10	*Lobaria macaronesica*	12535	-17,01617491	32,76162247	Madeira	Madeira
PM12-13c	PM12	PM12-13	*Lobaria macaronesica*	12545	-17,01663508	32,76014917	Madeira	Madeira
PM12-13d	PM12	PM12-13	*Lobaria macaronesica*	12546	-17,01663508	32,76014917	Madeira	Madeira
PM12-13i	PM12	PM12-13	*Lobaria macaronesica*	12565	-17,01663508	32,76014917	Madeira	Madeira
PM12-14a	PM12	PM12-14	*Lobaria macaronesica*	12551	-17,01663237	32,76019419	Madeira	Madeira
PM12-15g	PM12	PM12-15	*Lobaria macaronesica*	12564	-17,01662966	32,76023922	Madeira	Madeira
PM12-02d	PM12	PM12-2	*Lobaria macaronesica*	12494	-17,01602013	32,76156538	Madeira	Madeira
PM12-03d	PM12	PM12-3	*Lobaria macaronesica*	12500	-17,01604107	32,76156191	Madeira	Madeira
PM12-03e	PM12	PM12-3	*Lobaria macaronesica*	12501	-17,01604107	32,76156191	Madeira	Madeira
PM12-04d	PM12	PM12-4	*Lobaria macaronesica*	12505	-17,0160617	32,76153034	Madeira	Madeira
PM12-04e	PM12	PM12-4	*Lobaria macaronesica*	12506	-17,0160617	32,76153034	Madeira	Madeira
PM12-04f	PM12	PM12-4	*Lobaria macaronesica*	12507	-17,0160617	32,76153034	Madeira	Madeira
PM12-05d	PM12	PM12-5	*Lobaria macaronesica*	12512	-17,01616672	32,76159226	Madeira	Madeira
PM12-05e	PM12	PM12-5	*Lobaria macaronesica*	12513	-17,01616672	32,76159226	Madeira	Madeira
PM12-05f	PM12	PM12-5	*Lobaria macaronesica*	12514	-17,01616672	32,76159226	Madeira	Madeira
PM12-05g	PM12	PM12-5	*Lobaria macaronesica*	12515	-17,01616672	32,76159226	Madeira	Madeira
PM12-06c	PM12	PM12-6	*Lobaria macaronesica*	12518	-17,01617206	32,76159218	Madeira	Madeira
PM12-07h	PM12	PM12-7	*Lobaria macaronesica*	12570	-17,01619248	32,76159742	Madeira	Madeira
PM12-07i	PM12	PM12-7	*Lobaria macaronesica*	12571	-17,01619248	32,76159742	Madeira	Madeira
PM12-07j	PM12	PM12-7	*Lobaria macaronesica*	12572	-17,01619248	32,76159742	Madeira	Madeira
PM12-08d	PM12	PM12-8	*Lobaria macaronesica*	12526	-17,01626628	32,76160719	Madeira	Madeira
PM12-08e	PM12	PM12-8	*Lobaria macaronesica*	12527	-17,01626628	32,76160719	Madeira	Madeira
PM12-09b	PM12	PM12-9	*Lobaria macaronesica*	12530	-17,01620654	32,76162666	Madeira	Madeira
PM13-10a	PM13	PM13-10	*Lobaria macaronesica*	12629	-17,13152381	32,76343777	Madeira	Madeira
PM13-10b	PM13	PM13-10	*Lobaria macaronesica*	12630	-17,13152381	32,76343777	Madeira	Madeira

ID	Pop	PopTree	Species	VoucherID	X	Y	Location	Area
PM13-10c	PM13	PM13-10	*Lobaria macaronesica*	12631	-17,13152381	32,76343777	Madeira	Madeira
PM13-11d	PM13	PM13-11	*Lobaria macaronesica*	12636	-17,13152084	32,76342911	Madeira	Madeira
PM13-11e	PM13	PM13-11	*Lobaria macaronesica*	12637	-17,13152084	32,76342911	Madeira	Madeira
PM13-11f	PM13	PM13-11	*Lobaria macaronesica*	12638	-17,13152084	32,76342911	Madeira	Madeira
PM13-12d	PM13	PM13-12	*Lobaria macaronesica*	12645	-17,1315229	32,76343795	Madeira	Madeira
PM13-12e	PM13	PM13-12	*Lobaria macaronesica*	12646	-17,1315229	32,76343795	Madeira	Madeira
PM13-12f	PM13	PM13-12	*Lobaria macaronesica*	12647	-17,1315229	32,76343795	Madeira	Madeira
PM13-13c	PM13	PM13-13	*Lobaria macaronesica*	12650	-17,13152783	32,76341122	Madeira	Madeira
PM13-14f	PM13	PM13-14	*Lobaria macaronesica*	12660	-17,13147612	32,76353922	Madeira	Madeira
PM13-16e	PM13	PM13-16	*Lobaria macaronesica*	12668	-17,13158949	32,76363512	Madeira	Madeira
PM13-16g	PM13	PM13-16	*Lobaria macaronesica*	12670	-17,13158949	32,76363512	Madeira	Madeira
PM13-16h	PM13	PM13-16	*Lobaria macaronesica*	12671	-17,13158949	32,76363512	Madeira	Madeira
PM13-17f	PM13	PM13-17	*Lobaria macaronesica*	12677	-17,13166646	32,76369758	Madeira	Madeira
PM13-17g	PM13	PM13-17	*Lobaria macaronesica*	12678	-17,13166646	32,76369758	Madeira	Madeira
PM13-17h	PM13	PM13-17	*Lobaria macaronesica*	12679	-17,13166646	32,76369758	Madeira	Madeira
PM13-17i	PM13	PM13-17	*Lobaria macaronesica*	12680	-17,13166646	32,76369758	Madeira	Madeira
PM13-18d	PM13	PM13-18	*Lobaria macaronesica*	12684	-17,13166244	32,76370054	Madeira	Madeira
PM13-18e	PM13	PM13-18	*Lobaria macaronesica*	12685	-17,13166244	32,76370054	Madeira	Madeira
PM13-18f	PM13	PM13-18	*Lobaria macaronesica*	12731	-17,13166244	32,76370054	Madeira	Madeira
PM13-18g	PM13	PM13-18	*Lobaria macaronesica*	12732	-17,13166244	32,76370054	Madeira	Madeira
PM13-02a	PM13	PM13-2	*Lobaria macaronesica*	12576	-17,13163653	32,76336511	Madeira	Madeira
PM13-02b	PM13	PM13-2	*Lobaria macaronesica*	12577	-17,13163653	32,76336511	Madeira	Madeira
PM13-20e	PM13	PM13-20	*Lobaria macaronesica*	12693	-17,13165968	32,7637133	Madeira	Madeira
PM13-20f	PM13	PM13-20	*Lobaria macaronesica*	12694	-17,13165968	32,7637133	Madeira	Madeira
PM13-21c	PM13	PM13-21	*Lobaria macaronesica*	12697	-17,13170221	32,76371635	Madeira	Madeira
PM13-21d	PM13	PM13-21	*Lobaria macaronesica*	12698	-17,13170221	32,76371635	Madeira	Madeira
PM13-22g	PM13	PM13-22	*Lobaria macaronesica*	12706	-17,13170523	32,76371057	Madeira	Madeira

ID	Pop	PopTree	Species	VoucherID	X	Y	Location	Area
PM13-22h	PM13	PM13-22	*Lobaria macaronesica*	12707	-17,13170523	32,76371057	Madeira	Madeira
PM13-22i	PM13	PM13-22	*Lobaria macaronesica*	12708	-17,13170523	32,76371057	Madeira	Madeira
PM13-03e	PM13	PM13-3	*Lobaria macaronesica*	12587	-17,13158484	32,76339702	Madeira	Madeira
PM13-03f	PM13	PM13-3	*Lobaria macaronesica*	12588	-17,13158484	32,76339702	Madeira	Madeira
PM13-03g	PM13	PM13-3	*Lobaria macaronesica*	12589	-17,13158484	32,76339702	Madeira	Madeira
PM13-04c	PM13	PM13-4	*Lobaria macaronesica*	12592	-17,13159743	32,76341159	Madeira	Madeira
PM13-04d	PM13	PM13-4	*Lobaria macaronesica*	12593	-17,13159743	32,76341159	Madeira	Madeira
PM13-04e	PM13	PM13-4	*Lobaria macaronesica*	12594	-17,13159743	32,76341159	Madeira	Madeira
PM13-05a	PM13	PM13-5	*Lobaria macaronesica*	12595	-17,13160257	32,76340369	Madeira	Madeira
PM13-05i	PM13	PM13-5	*Lobaria macaronesica*	12603	-17,13160257	32,76340369	Madeira	Madeira
PM13-05j	PM13	PM13-5	*Lobaria macaronesica*	12604	-17,13160257	32,76340369	Madeira	Madeira
PM13-05k	PM13	PM13-5	*Lobaria macaronesica*	12605	-17,13160257	32,76340369	Madeira	Madeira
PM13-05l	PM13	PM13-5	*Lobaria macaronesica*	12606	-17,13160257	32,76340369	Madeira	Madeira
PM13-07e	PM13	PM13-7	*Lobaria macaronesica*	12612	-17,13158559	32,7634285	Madeira	Madeira
PM13-07g	PM13	PM13-7	*Lobaria macaronesica*	12614	-17,13158559	32,7634285	Madeira	Madeira
PM13-09i	PM13	PM13-9	*Lobaria macaronesica*	12627	-17,13152848	32,76343994	Madeira	Madeira
PM13-09j	PM13	PM13-9	*Lobaria macaronesica*	12628	-17,13152848	32,76343994	Madeira	Madeira
PM14-01c	PM14	PM14-1	*Lobaria macaronesica*	12739	-17,1893	32,83067	Madeira	Madeira
PM14-01d	PM14	PM14-1	*Lobaria macaronesica*	12740	-17,1893	32,83067	Madeira	Madeira
PM14-01e	PM14	PM14-1	*Lobaria macaronesica*	12741	-17,1893	32,83067	Madeira	Madeira
PM14-01f	PM14	PM14-1	*Lobaria macaronesica*	12742	-17,1893	32,83067	Madeira	Madeira
PM14-01g	PM14	PM14-1	*Lobaria macaronesica*	12743	-17,1893	32,83067	Madeira	Madeira
PM14-01h	PM14	PM14-1	*Lobaria macaronesica*	12744	-17,1893	32,83067	Madeira	Madeira
PM14-01i	PM14	PM14-1	*Lobaria macaronesica*	12745	-17,1893	32,83067	Madeira	Madeira
PM14-10b	PM14	PM14-10	*Lobaria macaronesica*	12794	-17,18926558	32,83075518	Madeira	Madeira
PM14-10c	PM14	PM14-10	*Lobaria macaronesica*	12795	-17,18926558	32,83075518	Madeira	Madeira
PM14-11e	PM14	PM14-11	*Lobaria macaronesica*	12804	-17,18924437	32,83075304	Madeira	Madeira

ID	Pop	PopTree	Species	VoucherID	X	Y	Location	Area
PM14-11f	PM14	PM14-11	*Lobaria macaronesica*	12805	-17,18924437	32,83075304	Madeira	Madeira
PM14-11g	PM14	PM14-11	*Lobaria macaronesica*	12806	-17,18924437	32,83075304	Madeira	Madeira
PM14-11h	PM14	PM14-11	*Lobaria macaronesica*	12807	-17,18924437	32,83075304	Madeira	Madeira
PM14-11i	PM14	PM14-11	*Lobaria macaronesica*	12808	-17,18924437	32,83075304	Madeira	Madeira
PM14-11k	PM14	PM14-11	*Lobaria macaronesica*	12973	-17,18924437	32,83075304	Madeira	Madeira
PM14-12d	PM14	PM14-12	*Lobaria macaronesica*	12813	-17,18927801	32,83081934	Madeira	Madeira
PM14-12e	PM14	PM14-12	*Lobaria macaronesica*	12814	-17,18927801	32,83081934	Madeira	Madeira
PM14-12f	PM14	PM14-12	*Lobaria macaronesica*	12815	-17,18927801	32,83081934	Madeira	Madeira
PM14-12g	PM14	PM14-12	*Lobaria macaronesica*	12816	-17,18927801	32,83081934	Madeira	Madeira
PM14-13f	PM14	PM14-13	*Lobaria macaronesica*	12823	-17,18929137	32,83090879	Madeira	Madeira
PM14-13g	PM14	PM14-13	*Lobaria macaronesica*	12824	-17,18929137	32,83090879	Madeira	Madeira
PM14-13h	PM14	PM14-13	*Lobaria macaronesica*	12825	-17,18929137	32,83090879	Madeira	Madeira
PM14-13j	PM14	PM14-13	*Lobaria macaronesica*	12861	-17,18929137	32,83090879	Madeira	Madeira
PM14-14d	PM14	PM14-14	*Lobaria macaronesica*	12829	-17,18930166	32,83089299	Madeira	Madeira
PM14-14e	PM14	PM14-14	*Lobaria macaronesica*	12830	-17,18930166	32,83089299	Madeira	Madeira
PM14-16f	PM14	PM14-16	*Lobaria macaronesica*	12838	-17,18922965	32,83099378	Madeira	Madeira
PM14-18d	PM14	PM14-18	*Lobaria macaronesica*	12847	-17,18919612	32,83101596	Madeira	Madeira
PM14-18e	PM14	PM14-18	*Lobaria macaronesica*	12848	-17,18919612	32,83101596	Madeira	Madeira
PM14-18f	PM14	PM14-18	*Lobaria macaronesica*	12849	-17,18919612	32,83101596	Madeira	Madeira
PM14-19c	PM14	PM14-19	*Lobaria macaronesica*	12852	-17,18919878	32,83101006	Madeira	Madeira
PM14-02c	PM14	PM14-2	*Lobaria macaronesica*	12748	-17,18929511	32,83066818	Madeira	Madeira
PM14-02d	PM14	PM14-2	*Lobaria macaronesica*	12749	-17,18929511	32,83066818	Madeira	Madeira
PM14-02e	PM14	PM14-2	*Lobaria macaronesica*	12750	-17,18929511	32,83066818	Madeira	Madeira
PM14-02f	PM14	PM14-2	*Lobaria macaronesica*	12751	-17,18929511	32,83066818	Madeira	Madeira
PM14-02g	PM14	PM14-2	*Lobaria macaronesica*	12752	-17,18929511	32,83066818	Madeira	Madeira
PM14-02h	PM14	PM14-2	*Lobaria macaronesica*	12753	-17,18929511	32,83066818	Madeira	Madeira
PM14-02i	PM14	PM14-2	*Lobaria macaronesica*	12754	-17,18929511	32,83066818	Madeira	Madeira

ID	Pop	PopTree	Species	VoucherID	X	Y	Location	Area
PM14-20f	PM14	PM14-20	*Lobaria macaronesica*	12860	-17,18917886	32,83101658	Madeira	Madeira
PM14-03b	PM14	PM14-3	*Lobaria macaronesica*	12756	-17,18930004	32,83067293	Madeira	Madeira
PM14-03c	PM14	PM14-3	*Lobaria macaronesica*	12757	-17,18930004	32,83067293	Madeira	Madeira
PM14-03d	PM14	PM14-3	*Lobaria macaronesica*	12758	-17,18930004	32,83067293	Madeira	Madeira
PM14-03e	PM14	PM14-3	*Lobaria macaronesica*	12759	-17,18930004	32,83067293	Madeira	Madeira
PM14-03f	PM14	PM14-3	*Lobaria macaronesica*	12760	-17,18930004	32,83067293	Madeira	Madeira
PM14-03g	PM14	PM14-3	*Lobaria macaronesica*	12761	-17,18930004	32,83067293	Madeira	Madeira
PM14-04c	PM14	PM14-4	*Lobaria macaronesica*	12764	-17,18928282	32,8307156	Madeira	Madeira
PM14-05e	PM14	PM14-5	*Lobaria macaronesica*	12769	-17,18928177	32,83073361	Madeira	Madeira
PM14-05f	PM14	PM14-5	*Lobaria macaronesica*	12770	-17,18928177	32,83073361	Madeira	Madeira
PM14-06c	PM14	PM14-6	*Lobaria macaronesica*	12773	-17,18933791	32,83075968	Madeira	Madeira
PM14-06d	PM14	PM14-6	*Lobaria macaronesica*	12774	-17,18933791	32,83075968	Madeira	Madeira
PM14-06e	PM14	PM14-6	*Lobaria macaronesica*	12775	-17,18933791	32,83075968	Madeira	Madeira
PM14-06f	PM14	PM14-6	*Lobaria macaronesica*	12776	-17,18933791	32,83075968	Madeira	Madeira
PM14-07c	PM14	PM14-7	*Lobaria macaronesica*	12779	-17,1893545	32,83078282	Madeira	Madeira
PM14-07d	PM14	PM14-7	*Lobaria macaronesica*	12780	-17,1893545	32,83078282	Madeira	Madeira
PM14-07e	PM14	PM14-7	*Lobaria macaronesica*	12781	-17,1893545	32,83078282	Madeira	Madeira
PM14-08a	PM14	PM14-8	*Lobaria macaronesica*	12782	-17,1893522	32,83076489	Madeira	Madeira
PM14-08b	PM14	PM14-8	*Lobaria macaronesica*	12783	-17,1893522	32,83076489	Madeira	Madeira
PM14-08c	PM14	PM14-8	*Lobaria macaronesica*	12784	-17,1893522	32,83076489	Madeira	Madeira
PM14-08d	PM14	PM14-8	*Lobaria macaronesica*	12785	-17,1893522	32,83076489	Madeira	Madeira
PM14-08e	PM14	PM14-8	*Lobaria macaronesica*	12786	-17,1893522	32,83076489	Madeira	Madeira
PM14-08f	PM14	PM14-8	*Lobaria macaronesica*	12787	-17,1893522	32,83076489	Madeira	Madeira
PM14-08g	PM14	PM14-8	*Lobaria macaronesica*	12788	-17,1893522	32,83076489	Madeira	Madeira
PM15-01f	PM15	PM15-1	*Lobaria macaronesica*	12868	-17,157942	32,826744	Madeira	Madeira
PM15-10a	PM15	PM15-10	*Lobaria macaronesica*	12902	-17,1577433	32,82589679	Madeira	Madeira
PM15-10b	PM15	PM15-10	*Lobaria macaronesica*	12903	-17,1577433	32,82589679	Madeira	Madeira

ID	Pop	PopTree	Species	VoucherID	X	Y	Location	Area
PM15-12d	PM15	PM15-12	*Lobaria macaronesica*	12912	-17,15774139	32,82589446	Madeira	Madeira
PM15-12e	PM15	PM15-12	*Lobaria macaronesica*	12913	-17,15774139	32,82589446	Madeira	Madeira
PM15-12f	PM15	PM15-12	*Lobaria macaronesica*	12914	-17,15774139	32,82589446	Madeira	Madeira
PM15-12g	PM15	PM15-12	*Lobaria macaronesica*	12915	-17,15774139	32,82589446	Madeira	Madeira
PM15-14a	PM15	PM15-14	*Lobaria macaronesica*	12919	-17,15789347	32,82579125	Madeira	Madeira
PM15-15a	PM15	PM15-15	*Lobaria macaronesica*	12920	-17,15851185	32,82564968	Madeira	Madeira
PM15-15b	PM15	PM15-15	*Lobaria macaronesica*	31772	-17,15851185	32,82564968	Madeira	Madeira
PM15-15c	PM15	PM15-15	*Lobaria macaronesica*	31773	-17,15851185	32,82564968	Madeira	Madeira
PM15-16d	PM15	PM15-16	*Lobaria macaronesica*	12924	-17,1585153	32,82564115	Madeira	Madeira
PM15-16e	PM15	PM15-16	*Lobaria macaronesica*	12925	-17,1585153	32,82564115	Madeira	Madeira
PM15-17e	PM15	PM15-17	*Lobaria macaronesica*	12930	-17,15858274	32,82571105	Madeira	Madeira
PM15-17f	PM15	PM15-17	*Lobaria macaronesica*	12931	-17,15858274	32,82571105	Madeira	Madeira
PM15-17g	PM15	PM15-17	*Lobaria macaronesica*	12932	-17,15858274	32,82571105	Madeira	Madeira
PM15-18d	PM15	PM15-18	*Lobaria macaronesica*	12936	-17,15856117	32,82575228	Madeira	Madeira
PM15-19f	PM15	PM15-19	*Lobaria macaronesica*	12942	-17,15855026	32,82530157	Madeira	Madeira
PM15-19g	PM15	PM15-19	*Lobaria macaronesica*	12943	-17,15855026	32,82530157	Madeira	Madeira
PM15-19h	PM15	PM15-19	*Lobaria macaronesica*	12944	-17,15855026	32,82530157	Madeira	Madeira
PM15-20b	PM15	PM15-20	*Lobaria macaronesica*	12949	-17,15853156	32,82529288	Madeira	Madeira
PM15-20c	PM15	PM15-20	*Lobaria macaronesica*	12950	-17,15853156	32,82529288	Madeira	Madeira
PM15-20d	PM15	PM15-20	*Lobaria macaronesica*	12951	-17,15853156	32,82529288	Madeira	Madeira
PM15-21d	PM15	PM15-21	*Lobaria macaronesica*	12955	-17,15853927	32,8253097	Madeira	Madeira
PM15-21e	PM15	PM15-21	*Lobaria macaronesica*	12956	-17,15853927	32,8253097	Madeira	Madeira
PM15-21f	PM15	PM15-21	*Lobaria macaronesica*	12957	-17,15853927	32,8253097	Madeira	Madeira
PM15-22b	PM15	PM15-22	*Lobaria macaronesica*	12961	-17,15852097	32,8253319	Madeira	Madeira
PM15-22c	PM15	PM15-22	*Lobaria macaronesica*	12962	-17,15852097	32,8253319	Madeira	Madeira
PM15-22d	PM15	PM15-22	*Lobaria macaronesica*	12963	-17,15852097	32,8253319	Madeira	Madeira
PM15-22e	PM15	PM15-22	*Lobaria macaronesica*	12964	-17,15852097	32,8253319	Madeira	Madeira

ID	Pop	PopTree	Species	VoucherID	X	Y	Location	Area
PM15-23a	PM15	PM15-23	*Lobaria macaronesica*	12965	-17,15849292	32,82526377	Madeira	Madeira
PM15-23c	PM15	PM15-23	*Lobaria macaronesica*	12967	-17,15849292	32,82526377	Madeira	Madeira
PM15-23d	PM15	PM15-23	*Lobaria macaronesica*	12968	-17,15849292	32,82526377	Madeira	Madeira
PM15-23e	PM15	PM15-23	*Lobaria macaronesica*	12969	-17,15849292	32,82526377	Madeira	Madeira
PM15-03c	PM15	PM15-3	*Lobaria macaronesica*	12873	-17,157777	32,82655697	Madeira	Madeira
PM15-03d	PM15	PM15-3	*Lobaria macaronesica*	12874	-17,157777	32,82655697	Madeira	Madeira
PM15-03e	PM15	PM15-3	*Lobaria macaronesica*	12875	-17,157777	32,82655697	Madeira	Madeira
PM15-04c	PM15	PM15-4	*Lobaria macaronesica*	12880	-17,15777569	32,82650289	Madeira	Madeira
PM15-04d	PM15	PM15-4	*Lobaria macaronesica*	12881	-17,15777569	32,82650289	Madeira	Madeira
PM15-05c	PM15	PM15-5	*Lobaria macaronesica*	12884	-17,1577519	32,82643362	Madeira	Madeira
PM15-05d	PM15	PM15-5	*Lobaria macaronesica*	12885	-17,1577519	32,82643362	Madeira	Madeira
PM15-05e	PM15	PM15-5	*Lobaria macaronesica*	12886	-17,1577519	32,82643362	Madeira	Madeira
PM15-05f	PM15	PM15-5	*Lobaria macaronesica*	12887	-17,1577519	32,82643362	Madeira	Madeira
PM15-06e	PM15	PM15-6	*Lobaria macaronesica*	12892	-17,15783216	32,82607939	Madeira	Madeira
PM15-06f	PM15	PM15-6	*Lobaria macaronesica*	12893	-17,15783216	32,82607939	Madeira	Madeira
PM15-06g	PM15	PM15-6	*Lobaria macaronesica*	12894	-17,15783216	32,82607939	Madeira	Madeira
PM15-07a	PM15	PM15-7	*Lobaria macaronesica*	12895	-17,15779583	32,82601411	Madeira	Madeira
PM15-07b	PM15	PM15-7	*Lobaria macaronesica*	12896	-17,15779583	32,82601411	Madeira	Madeira
PM15-07c	PM15	PM15-7	*Lobaria macaronesica*	12897	-17,15779583	32,82601411	Madeira	Madeira
PM15-09a	PM15	PM15-9	*Lobaria macaronesica*	12901	-17,15780551	32,82590972	Madeira	Madeira
PM16-01d	PM16	PM16-1	*Lobaria macaronesica*	13037	-17,14085	32,80675	Madeira	Madeira
PM16-01e	PM16	PM16-1	*Lobaria macaronesica*	13038	-17,14085	32,80675	Madeira	Madeira
PM16-10g	PM16	PM16-10	*Lobaria macaronesica*	13083	-17,14062971	32,80713525	Madeira	Madeira
PM16-10h	PM16	PM16-10	*Lobaria macaronesica*	13084	-17,14062971	32,80713525	Madeira	Madeira
PM16-10i	PM16	PM16-10	*Lobaria macaronesica*	13085	-17,14062971	32,80713525	Madeira	Madeira
PM16-11c	PM16	PM16-11	*Lobaria macaronesica*	13088	-17,1406093	32,80714058	Madeira	Madeira
PM16-12c	PM16	PM16-12	*Lobaria macaronesica*	13091	-17,14058125	32,80712753	Madeira	Madeira

ID	Pop	PopTree	Species	VoucherID	X	Y	Location	Area
PM16-13f	PM16	PM16-13	*Lobaria macaronesica*	13097	-17,14058053	32,80713653	Madeira	Madeira
PM16-14d	PM16	PM16-14	*Lobaria macaronesica*	13101	-17,14057899	32,80714084	Madeira	Madeira
PM16-14e	PM16	PM16-14	*Lobaria macaronesica*	13102	-17,14057899	32,80714084	Madeira	Madeira
PM16-15e	PM16	PM16-15	*Lobaria macaronesica*	13107	-17,14059819	32,80732044	Madeira	Madeira
PM16-15f	PM16	PM16-15	*Lobaria macaronesica*	13108	-17,14059819	32,80732044	Madeira	Madeira
PM16-16e	PM16	PM16-16	*Lobaria macaronesica*	13113	-17,1404083	32,80729549	Madeira	Madeira
PM16-16f	PM16	PM16-16	*Lobaria macaronesica*	13114	-17,1404083	32,80729549	Madeira	Madeira
PM16-17a	PM16	PM16-17	*Lobaria macaronesica*	13115	-17,14043564	32,80720834	Madeira	Madeira
PM16-17b	PM16	PM16-17	*Lobaria macaronesica*	13116	-17,14043564	32,80720834	Madeira	Madeira
PM16-17c	PM16	PM16-17	*Lobaria macaronesica*	13117	-17,14043564	32,80720834	Madeira	Madeira
PM16-17d	PM16	PM16-17	*Lobaria macaronesica*	13118	-17,14043564	32,80720834	Madeira	Madeira
PM16-19d	PM16	PM16-19	*Lobaria macaronesica*	13124	-17,14042758	32,80720056	Madeira	Madeira
PM16-19e	PM16	PM16-19	*Lobaria macaronesica*	13125	-17,14042758	32,80720056	Madeira	Madeira
PM16-02a	PM16	PM16-2	*Lobaria macaronesica*	13039	-17,14084554	32,80673701	Madeira	Madeira
PM16-02b	PM16	PM16-2	*Lobaria macaronesica*	13040	-17,14084554	32,80673701	Madeira	Madeira
PM16-20e	PM16	PM16-20	*Lobaria macaronesica*	13130	-17,14034214	32,80714649	Madeira	Madeira
PM16-20f	PM16	PM16-20	*Lobaria macaronesica*	13131	-17,14034214	32,80714649	Madeira	Madeira
PM16-21e	PM16	PM16-21	*Lobaria macaronesica*	13136	-17,14025594	32,8071997	Madeira	Madeira
PM16-04g	PM16	PM16-4	*Lobaria macaronesica*	13051	-17,14079231	32,80695999	Madeira	Madeira
PM16-04h	PM16	PM16-4	*Lobaria macaronesica*	13052	-17,14079231	32,80695999	Madeira	Madeira
PM16-05d	PM16	PM16-5	*Lobaria macaronesica*	13056	-17,14080872	32,80690771	Madeira	Madeira
PM16-05e	PM16	PM16-5	*Lobaria macaronesica*	13057	-17,14080872	32,80690771	Madeira	Madeira
PM16-05f	PM16	PM16-5	*Lobaria macaronesica*	13058	-17,14080872	32,80690771	Madeira	Madeira
PM16-06a	PM16	PM16-6	*Lobaria macaronesica*	13060	-17,1407127	32,80701594	Madeira	Madeira
PM16-08e	PM16	PM16-8	*Lobaria macaronesica*	13072	-17,14061861	32,80700609	Madeira	Madeira
PM17-12c	PM17	PM17-12	*Lobaria macaronesica*	13170	-16,83215236	32,74473401	Madeira	Madeira
PM17-13a	PM17	PM17-13	*Lobaria macaronesica*	13171	-16,83223653	32,74487991	Madeira	Madeira

ID	Pop	PopTree	Species	VoucherID	X	Y	Location	Area
PM17-13c	PM17	PM17-13	*Lobaria macaronesica*	13173	-16,83223653	32,74487991	Madeira	Madeira
PM17-14d	PM17	PM17-14	*Lobaria macaronesica*	13177	-16,83222912	32,74488639	Madeira	Madeira
PM17-15c	PM17	PM17-15	*Lobaria macaronesica*	13180	-16,83227811	32,74493404	Madeira	Madeira
PM17-18a	PM17	PM17-18	*Lobaria macaronesica*	13186	-16,83230988	32,74511701	Madeira	Madeira
PM17-18b	PM17	PM17-18	*Lobaria macaronesica*	13187	-16,83230988	32,74511701	Madeira	Madeira
PM17-21a	PM17	PM17-21	*Lobaria macaronesica*	13190	-16,83348034	32,74523044	Madeira	Madeira
PM17-21b	PM17	PM17-21	*Lobaria macaronesica*	13191	-16,83348034	32,74523044	Madeira	Madeira
PM17-21c	PM17	PM17-21	*Lobaria macaronesica*	13192	-16,83348034	32,74523044	Madeira	Madeira
PM17-08f	PM17	PM17-8	*Lobaria macaronesica*	13159	-16,83213442	32,74464919	Madeira	Madeira
PM17-08g	PM17	PM17-8	*Lobaria macaronesica*	13160	-16,83213442	32,74464919	Madeira	Madeira
PM17-09b	PM17	PM17-9	*Lobaria macaronesica*	13162	-16,83211324	32,74465139	Madeira	Madeira
PM9-01a	PM9	PM9-1	*Lobaria macaronesica*	12292	-16,88465	32,738	Madeira	Madeira
PM9-01b	PM9	PM9-1	*Lobaria macaronesica*	12293	-16,88465	32,738	Madeira	Madeira
PM9-01c	PM9	PM9-1	*Lobaria macaronesica*	12294	-16,88465	32,738	Madeira	Madeira
PM9-01f	PM9	PM9-1	*Lobaria macaronesica*	12297	-16,88465	32,738	Madeira	Madeira
PM9-11a	PM9	PM9-11	*Lobaria macaronesica*	12345	-16,88529006	32,73799037	Madeira	Madeira
PM9-12a	PM9	PM9-12	*Lobaria macaronesica*	12346	-16,88529539	32,73799029	Madeira	Madeira
PM9-12b	PM9	PM9-12	*Lobaria macaronesica*	12347	-16,88529539	32,73799029	Madeira	Madeira
PM9-12c	PM9	PM9-12	*Lobaria macaronesica*	12348	-16,88529539	32,73799029	Madeira	Madeira
PM9-12d	PM9	PM9-12	*Lobaria macaronesica*	12349	-16,88529539	32,73799029	Madeira	Madeira
PM9-12e	PM9	PM9-12	*Lobaria macaronesica*	12350	-16,88529539	32,73799029	Madeira	Madeira
PM9-13a	PM9	PM9-13	*Lobaria macaronesica*	12351	-16,88534339	32,73798957	Madeira	Madeira
PM9-13b	PM9	PM9-13	*Lobaria macaronesica*	12352	-16,88534339	32,73798957	Madeira	Madeira
PM9-14a	PM9	PM9-14	*Lobaria macaronesica*	12357	-16,88550341	32,73798716	Madeira	Madeira
PM9-14b	PM9	PM9-14	*Lobaria macaronesica*	12358	-16,88550341	32,73798716	Madeira	Madeira
PM9-15e	PM9	PM9-15	*Lobaria macaronesica*	12363	-16,88555675	32,73798636	Madeira	Madeira
PM9-15f	PM9	PM9-15	*Lobaria macaronesica*	12364	-16,88555675	32,73798636	Madeira	Madeira

ID	Pop	PopTree	Species	VoucherID	X	Y	Location	Area
PM9-16a	PM9	PM9-16	*Lobaria macaronesica*	12365	-16,88561008	32,73798555	Madeira	Madeira
PM9-16c	PM9	PM9-16	*Lobaria macaronesica*	12367	-16,88561008	32,73798555	Madeira	Madeira
PM9-16d	PM9	PM9-16	*Lobaria macaronesica*	12368	-16,88561008	32,73798555	Madeira	Madeira
PM9-16e	PM9	PM9-16	*Lobaria macaronesica*	12369	-16,88561008	32,73798555	Madeira	Madeira
PM9-16f	PM9	PM9-16	*Lobaria macaronesica*	12370	-16,88561008	32,73798555	Madeira	Madeira
PM9-16g	PM9	PM9-16	*Lobaria macaronesica*	12371	-16,88561008	32,73798555	Madeira	Madeira
PM9-17a	PM9	PM9-17	*Lobaria macaronesica*	12373	-16,88566342	32,73798475	Madeira	Madeira
PM9-17b	PM9	PM9-17	*Lobaria macaronesica*	12374	-16,88566342	32,73798475	Madeira	Madeira
PM9-17c	PM9	PM9-17	*Lobaria macaronesica*	12375	-16,88566342	32,73798475	Madeira	Madeira
PM9-18a	PM9	PM9-18	*Lobaria macaronesica*	12380	-16,88571676	32,73798395	Madeira	Madeira
PM9-18b	PM9	PM9-18	*Lobaria macaronesica*	12381	-16,88571676	32,73798395	Madeira	Madeira
PM9-18c	PM9	PM9-18	*Lobaria macaronesica*	12382	-16,88571676	32,73798395	Madeira	Madeira
PM9-18d	PM9	PM9-18	*Lobaria macaronesica*	12383	-16,88571676	32,73798395	Madeira	Madeira
PM9-19a	PM9	PM9-19	*Lobaria macaronesica*	12387	-16,8857701	32,73798315	Madeira	Madeira
PM9-02e	PM9	PM9-2	*Lobaria macaronesica*	12302	-16,88470334	32,7379992	Madeira	Madeira
PM9-20a	PM9	PM9-20	*Lobaria macaronesica*	12388	-16,88577543	32,73798307	Madeira	Madeira
PM9-20b	PM9	PM9-20	*Lobaria macaronesica*	12389	-16,88577543	32,73798307	Madeira	Madeira
PM9-20c	PM9	PM9-20	*Lobaria macaronesica*	12390	-16,88577543	32,73798307	Madeira	Madeira
PM9-21f	PM9	PM9-21	*Lobaria macaronesica*	12399	-16,88582344	32,73798234	Madeira	Madeira
PM9-22a	PM9	PM9-22	*Lobaria macaronesica*	12401	-16,88614346	32,73797753	Madeira	Madeira
PM9-03d	PM9	PM9-3	*Lobaria macaronesica*	12309	-16,88475668	32,73799839	Madeira	Madeira
PM9-03e	PM9	PM9-3	*Lobaria macaronesica*	12310	-16,88475668	32,73799839	Madeira	Madeira
PM9-03f	PM9	PM9-3	*Lobaria macaronesica*	12311	-16,88475668	32,73799839	Madeira	Madeira
PM9-03g	PM9	PM9-3	*Lobaria macaronesica*	12312	-16,88475668	32,73799839	Madeira	Madeira
PM9-04d	PM9	PM9-4	*Lobaria macaronesica*	12316	-16,88481001	32,73799759	Madeira	Madeira
PM9-05c	PM9	PM9-5	*Lobaria macaronesica*	12321	-16,88481535	32,73799751	Madeira	Madeira
PM9-06a	PM9	PM9-6	*Lobaria macaronesica*	12324	-16,88486335	32,73799679	Madeira	Madeira

ID	Pop	PopTree	Species	VoucherID	X	Y	Location	Area
PM9-06c	PM9	PM9-6	*Lobaria macaronesica*	12326	-16,88486335	32,73799679	Madeira	Madeira
PM9-07b	PM9	PM9-7	*Lobaria macaronesica*	12330	-16,88491669	32,73799599	Madeira	Madeira
PM9-07c	PM9	PM9-7	*Lobaria macaronesica*	12331	-16,88491669	32,73799599	Madeira	Madeira
PM9-08a	PM9	PM9-8	*Lobaria macaronesica*	12333	-16,8850767	32,73799358	Madeira	Madeira
PM9-09b	PM9	PM9-9	*Lobaria macaronesica*	12337	-16,88513004	32,73799278	Madeira	Madeira
PM9-09c	PM9	PM9-9	*Lobaria macaronesica*	12338	-16,88513004	32,73799278	Madeira	Madeira
PS-18a	PS	PS-18	*Lobaria macaronesica*	12239	-9,389	38,792	Sintra	Ib. Peninsula
PS-19a	PS	PS-19	*Lobaria macaronesica*	12243	-9,389	38,792	Sintra	Ib. Peninsula
PS-22c	PS	PS-22	*Lobaria macaronesica*	12249	-9,389	38,792	Sintra	Ib. Peninsula
PS-22d	PS	PS-22	*Lobaria macaronesica*	12250	-9,389	38,792	Sintra	Ib. Peninsula
PS-23b	PS	PS-23	*Lobaria macaronesica*	12252	-9,389	38,792	Sintra	Ib. Peninsula
PS-25a	PS	PS-25	*Lobaria macaronesica*	12257	-9,389	38,792	Sintra	Ib. Peninsula
PS-34b	PS	PS-34	*Lobaria macaronesica*	12277	-9,389279912	38,791801	Sintra	Ib. Peninsula
PS-34e	PS	PS-34	*Lobaria macaronesica*	12280	-9,389279912	38,791801	Sintra	Ib. Peninsula
PS-36c	PS	PS-36	*Lobaria macaronesica*	12287	-9,392804214	38,79182075	Sintra	Ib. Peninsula
PS-36d	PS	PS-36	*Lobaria macaronesica*	12288	-9,392804214	38,79182075	Sintra	Ib. Peninsula
PS2-02a	PS	PS2-2	*Lobaria macaronesica*	31735	-9,39024	38,791834	Sintra	Ib. Peninsula
PS2-02f	PS	PS2-2	*Lobaria macaronesica*	31740	-9,39024	38,791834	Sintra	Ib. Peninsula
PS2-04b	PS	PS2-4	*Lobaria macaronesica*	31745	-9,390095	38,787106	Sintra	Ib. Peninsula
PS2-05a	PS	PS2-5	*Lobaria macaronesica*	31746	-9,390095	38,787106	Sintra	Ib. Peninsula
SG1-01b	SG1	SG1-1	*Lobaria macaronesica*	11937	-17,29628	28,15027	La Gomera	Canary Isl.
SG1-01d	SG1	SG1-1	*Lobaria macaronesica*	11939	-17,29628	28,15027	La Gomera	Canary Isl.
SG1-10a	SG1	SG1-10	*Lobaria macaronesica*	11963	-17,29620425	28,15052903	La Gomera	Canary Isl.
SG1-10c	SG1	SG1-10	*Lobaria macaronesica*	11965	-17,29620425	28,15052903	La Gomera	Canary Isl.
SG1-11a	SG1	SG1-11	*Lobaria macaronesica*	11966	-17,29630564	28,15052103	La Gomera	Canary Isl.
SG1-11b	SG1	SG1-11	*Lobaria macaronesica*	11967	-17,29630564	28,15052103	La Gomera	Canary Isl.
SG1-11e	SG1	SG1-11	*Lobaria macaronesica*	11970	-17,29630564	28,15052103	La Gomera	Canary Isl.

ID	Pop	PopTree	Species	VoucherID	X	Y	Location	Area
SG1-13f	SG1	SG1-13	*Lobaria macaronesica*	11978	-17,29630176	28,15055861	La Gomera	Canary Isl.
SG1-13g	SG1	SG1-13	*Lobaria macaronesica*	11979	-17,29630176	28,15055861	La Gomera	Canary Isl.
SG1-14f	SG1	SG1-14	*Lobaria macaronesica*	11985	-17,29634349	28,15058443	La Gomera	Canary Isl.
SG1-14g	SG1	SG1-14	*Lobaria macaronesica*	11986	-17,29634349	28,15058443	La Gomera	Canary Isl.
SG1-14h	SG1	SG1-14	*Lobaria macaronesica*	11987	-17,29634349	28,15058443	La Gomera	Canary Isl.
SG1-16b	SG1	SG1-16	*Lobaria macaronesica*	11992	-17,29631433	28,15063614	La Gomera	Canary Isl.
SG1-16d	SG1	SG1-16	*Lobaria macaronesica*	11994	-17,29631433	28,15063614	La Gomera	Canary Isl.
SG1-17c	SG1	SG1-17	*Lobaria macaronesica*	11997	-17,2962848	28,15068352	La Gomera	Canary Isl.
SG1-17d	SG1	SG1-17	*Lobaria macaronesica*	11998	-17,2962848	28,15068352	La Gomera	Canary Isl.
SG1-18c	SG1	SG1-18	*Lobaria macaronesica*	12002	-17,29621095	28,15065311	La Gomera	Canary Isl.
SG1-18d	SG1	SG1-18	*Lobaria macaronesica*	12003	-17,29621095	28,15065311	La Gomera	Canary Isl.
SG1-02d	SG1	SG1-2	*Lobaria macaronesica*	11943	-17,29628349	28,15023405	La Gomera	Canary Isl.
SG1-21a	SG1	SG1-21	*Lobaria macaronesica*	12006	-17,29567847	28,15014655	La Gomera	Canary Isl.
SG1-21b	SG1	SG1-21	*Lobaria macaronesica*	12007	-17,29567847	28,15014655	La Gomera	Canary Isl.
SG1-03c	SG1	SG1-3	*Lobaria macaronesica*	11947	-17,29620358	28,15024792	La Gomera	Canary Isl.
SG1-04b	SG1	SG1-4	*Lobaria macaronesica*	11949	-17,29620749	28,15026563	La Gomera	Canary Isl.
SG1-04c	SG1	SG1-4	*Lobaria macaronesica*	11950	-17,29620749	28,15026563	La Gomera	Canary Isl.
SG1-04d	SG1	SG1-4	*Lobaria macaronesica*	11951	-17,29620749	28,15026563	La Gomera	Canary Isl.
SG1-06a	SG1	SG1-6	*Lobaria macaronesica*	11953	-17,2962616	28,15029601	La Gomera	Canary Isl.
SG1-06c	SG1	SG1-6	*Lobaria macaronesica*	11955	-17,2962616	28,15029601	La Gomera	Canary Isl.
SG1-07b	SG1	SG1-7	*Lobaria macaronesica*	11957	-17,2962525	28,15033118	La Gomera	Canary Isl.
SG1-08	SG1	SG1-8	*Lobaria macaronesica*	11959	-17,29620647	28,15036675	La Gomera	Canary Isl.
SG1-09a	SG1	SG1-9	*Lobaria macaronesica*	11960	-17,29620233	28,15043883	La Gomera	Canary Isl.
SG3-01d	SG3	SG3-1	*Lobaria macaronesica*	10465	-17,25633	28,13098	La Gomera	Canary Isl.
SG3-14c	SG3	SG3-14	*Lobaria macaronesica*	10499	-17,25646975	28,13183593	La Gomera	Canary Isl.
SG3-14f	SG3	SG3-14	*Lobaria macaronesica*	10502	-17,25646975	28,13183593	La Gomera	Canary Isl.
SG3-19c	SG3	SG3-19	*Lobaria macaronesica*	10516	-17,25662357	28,13205498	La Gomera	Canary Isl.

ID	Pop	PopTree	Species	VoucherID	X	Y	Location	Area
SG3-02c	SG3	SG3-2	*Lobaria macaronesica*	10468	-17,25633973	28,13098263	La Gomera	Canary Isl.
SG3-20b	SG3	SG3-20	*Lobaria macaronesica*	10520	-17,25664197	28,13207658	La Gomera	Canary Isl.
SG3-20c	SG3	SG3-20	*Lobaria macaronesica*	10521	-17,25664197	28,13207658	La Gomera	Canary Isl.
SG3-21a	SG3	SG3-21	*Lobaria macaronesica*	10522	-17,25692687	28,13197931	La Gomera	Canary Isl.
SG3-21b	SG3	SG3-21	*Lobaria macaronesica*	10523	-17,25692687	28,13197931	La Gomera	Canary Isl.
SG3-21d	SG3	SG3-21	*Lobaria macaronesica*	10525	-17,25692687	28,13197931	La Gomera	Canary Isl.
SG3-23c	SG3	SG3-23	*Lobaria macaronesica*	10532	-17,25696468	28,13196837	La Gomera	Canary Isl.
SG3-23d	SG3	SG3-23	*Lobaria macaronesica*	10533	-17,25696468	28,13196837	La Gomera	Canary Isl.
SG3-23e	SG3	SG3-23	*Lobaria macaronesica*	10534	-17,25696468	28,13196837	La Gomera	Canary Isl.
SG3-26e	SG3	SG3-26	*Lobaria macaronesica*	10545	-17,25706458	28,13200226	La Gomera	Canary Isl.
SG3-27c	SG3	SG3-27	*Lobaria macaronesica*	10549	-17,25697085	28,1319671	La Gomera	Canary Isl.
SG3-27d	SG3	SG3-27	*Lobaria macaronesica*	10550	-17,25697085	28,1319671	La Gomera	Canary Isl.
SG3-27e	SG3	SG3-27	*Lobaria macaronesica*	10551	-17,25697085	28,1319671	La Gomera	Canary Isl.
SG3-27f	SG3	SG3-27	*Lobaria macaronesica*	10552	-17,25697085	28,1319671	La Gomera	Canary Isl.
SG3-03a	SG3	SG3-3	*Lobaria macaronesica*	10469	-17,25632074	28,13098912	La Gomera	Canary Isl.
SG3-03b	SG3	SG3-3	*Lobaria macaronesica*	10470	-17,25632074	28,13098912	La Gomera	Canary Isl.
SG3-03c	SG3	SG3-3	*Lobaria macaronesica*	10471	-17,25632074	28,13098912	La Gomera	Canary Isl.
SG3-05b	SG3	SG3-5	*Lobaria macaronesica*	10474	-17,2562981	28,13101892	La Gomera	Canary Isl.
SG3-06b	SG3	SG3-6	*Lobaria macaronesica*	10478	-17,25631763	28,13110747	La Gomera	Canary Isl.
SG3-08b	SG3	SG3-8	*Lobaria macaronesica*	10483	-17,25648852	28,13129254	La Gomera	Canary Isl.
SG4-01b	SG4	SG4-1	*Lobaria macaronesica*	12016	-17,21512	28,12216	La Gomera	Canary Isl.
SG4-01c	SG4	SG4-1	*Lobaria macaronesica*	12017	-17,21512	28,12216	La Gomera	Canary Isl.
SG4-12a	SG4	SG4-12	*Lobaria macaronesica*	12051	-17,21542072	28,12171728	La Gomera	Canary Isl.
SG4-12b	SG4	SG4-12	*Lobaria macaronesica*	12052	-17,21542072	28,12171728	La Gomera	Canary Isl.
SG4-12c	SG4	SG4-12	*Lobaria macaronesica*	12053	-17,21542072	28,12171728	La Gomera	Canary Isl.
SG4-13a	SG4	SG4-13	*Lobaria macaronesica*	12054	-17,21546041	28,12163421	La Gomera	Canary Isl.
SG4-13b	SG4	SG4-13	*Lobaria macaronesica*	12055	-17,21546041	28,12163421	La Gomera	Canary Isl.

ID	Pop	PopTree	Species	VoucherID	X	Y	Location	Area
SG4-13c	SG4	SG4-13	*Lobaria macaronesica*	12056	-17,21546041	28,12163421	La Gomera	Canary Isl.
SG4-14a	SG4	SG4-14	*Lobaria macaronesica*	12057	-17,21549731	28,12156987	La Gomera	Canary Isl.
SG4-14d	SG4	SG4-14	*Lobaria macaronesica*	12060	-17,21549731	28,12156987	La Gomera	Canary Isl.
SG4-14e	SG4	SG4-14	*Lobaria macaronesica*	12061	-17,21549731	28,12156987	La Gomera	Canary Isl.
SG4-16	SG4	SG4-16	*Lobaria macaronesica*	12069	-17,21548962	28,12156753	La Gomera	Canary Isl.
SG4-18a	SG4	SG4-18	*Lobaria macaronesica*	12071	-17,21557932	28,12140559	La Gomera	Canary Isl.
SG4-19b	SG4	SG4-19	*Lobaria macaronesica*	12074	-17,21557737	28,12140612	La Gomera	Canary Isl.
SG4-19c	SG4	SG4-19	*Lobaria macaronesica*	12075	-17,21557737	28,12140612	La Gomera	Canary Isl.
SG4-02d	SG4	SG4-2	*Lobaria macaronesica*	12021	-17,21512684	28,12213362	La Gomera	Canary Isl.
SG4-20a	SG4	SG4-20	*Lobaria macaronesica*	12077	-17,21557405	28,12139759	La Gomera	Canary Isl.
SG4-20b	SG4	SG4-20	*Lobaria macaronesica*	12078	-17,21557405	28,12139759	La Gomera	Canary Isl.
SG4-21	SG4	SG4-21	*Lobaria macaronesica*	12080	-17,21564016	28,12122694	La Gomera	Canary Isl.
SG4-22a	SG4	SG4-22	*Lobaria macaronesica*	12081	-17,21573869	28,12106906	La Gomera	Canary Isl.
SG4-22b	SG4	SG4-22	*Lobaria macaronesica*	12082	-17,21573869	28,12106906	La Gomera	Canary Isl.
SG4-22c	SG4	SG4-22	*Lobaria macaronesica*	12083	-17,21573869	28,12106906	La Gomera	Canary Isl.
SG4-22d	SG4	SG4-22	*Lobaria macaronesica*	12084	-17,21573869	28,12106906	La Gomera	Canary Isl.
SG4-23b	SG4	SG4-23	*Lobaria macaronesica*	12087	-17,21575453	28,12097994	La Gomera	Canary Isl.
SG4-23c	SG4	SG4-23	*Lobaria macaronesica*	12088	-17,21575453	28,12097994	La Gomera	Canary Isl.
SG4-23d	SG4	SG4-23	*Lobaria macaronesica*	12089	-17,21575453	28,12097994	La Gomera	Canary Isl.
SG4-23e	SG4	SG4-23	*Lobaria macaronesica*	12090	-17,21575453	28,12097994	La Gomera	Canary Isl.
SG4-24a	SG4	SG4-24	*Lobaria macaronesica*	12092	-17,21585369	28,12072396	La Gomera	Canary Isl.
SG4-24b	SG4	SG4-24	*Lobaria macaronesica*	12093	-17,21585369	28,12072396	La Gomera	Canary Isl.
SG4-03b	SG4	SG4-3	*Lobaria macaronesica*	12023	-17,21515148	28,12209415	La Gomera	Canary Isl.
SG4-03d	SG4	SG4-3	*Lobaria macaronesica*	12025	-17,21515148	28,12209415	La Gomera	Canary Isl.
SG4-04b	SG4	SG4-4	*Lobaria macaronesica*	12027	-17,21515809	28,12207709	La Gomera	Canary Isl.
SG4-05e	SG4	SG4-5	*Lobaria macaronesica*	12032	-17,21517779	28,12204551	La Gomera	Canary Isl.
SG4-06b	SG4	SG4-6	*Lobaria macaronesica*	12034	-17,21522258	28,1219964	La Gomera	Canary Isl.

ID	Pop	PopTree	Species	VoucherID	X	Y	Location	Area
SG4-06c	SG4	SG4-6	*Lobaria macaronesica*	12035	-17,21522258	28,1219964	La Gomera	Canary Isl.
SG4-08b	SG4	SG4-8	*Lobaria macaronesica*	12043	-17,21526386	28,12194632	La Gomera	Canary Isl.
SG4-08c	SG4	SG4-8	*Lobaria macaronesica*	12044	-17,21526386	28,12194632	La Gomera	Canary Isl.
SG4-09c	SG4	SG4-9	*Lobaria macaronesica*	12047	-17,21530355	28,12186325	La Gomera	Canary Isl.
SG4-09d	SG4	SG4-9	*Lobaria macaronesica*	12048	-17,21530355	28,12186325	La Gomera	Canary Isl.
SG5-01a	SG5	SG5-1	*Lobaria macaronesica*	10553	-17,25633	28,13098	La Gomera	Canary Isl.
SG5-01d	SG5	SG5-1	*Lobaria macaronesica*	10556	-17,25633	28,13098	La Gomera	Canary Isl.
SG5-01e	SG5	SG5-1	*Lobaria macaronesica*	10557	-17,25633	28,13098	La Gomera	Canary Isl.
SG5-01f	SG5	SG5-1	*Lobaria macaronesica*	10558	-17,25633	28,13098	La Gomera	Canary Isl.
SG5-01g	SG5	SG5-1	*Lobaria macaronesica*	10559	-17,25633	28,13098	La Gomera	Canary Isl.
SG5-10a	SG5	SG5-10	*Lobaria macaronesica*	10588	-17,25643172	28,13081686	La Gomera	Canary Isl.
SG5-10b	SG5	SG5-10	*Lobaria macaronesica*	10589	-17,25643172	28,13081686	La Gomera	Canary Isl.
SG5-10c	SG5	SG5-10	*Lobaria macaronesica*	10590	-17,25643172	28,13081686	La Gomera	Canary Isl.
SG5-10d	SG5	SG5-10	*Lobaria macaronesica*	10591	-17,25643172	28,13081686	La Gomera	Canary Isl.
SG5-10e	SG5	SG5-10	*Lobaria macaronesica*	10592	-17,25643172	28,13081686	La Gomera	Canary Isl.
SG5-11a	SG5	SG5-11	*Lobaria macaronesica*	10593	-17,25645633	28,13077738	La Gomera	Canary Isl.
SG5-11b	SG5	SG5-11	*Lobaria macaronesica*	10594	-17,25645633	28,13077738	La Gomera	Canary Isl.
SG5-11c	SG5	SG5-11	*Lobaria macaronesica*	10595	-17,25645633	28,13077738	La Gomera	Canary Isl.
SG5-11d	SG5	SG5-11	*Lobaria macaronesica*	10596	-17,25645633	28,13077738	La Gomera	Canary Isl.
SG5-11e	SG5	SG5-11	*Lobaria macaronesica*	10597	-17,25645633	28,13077738	La Gomera	Canary Isl.
SG5-13a	SG5	SG5-13	*Lobaria macaronesica*	10600	-17,25658529	28,13058679	La Gomera	Canary Isl.
SG5-13b	SG5	SG5-13	*Lobaria macaronesica*	10601	-17,25658529	28,13058679	La Gomera	Canary Isl.
SG5-13c	SG5	SG5-13	*Lobaria macaronesica*	10602	-17,25658529	28,13058679	La Gomera	Canary Isl.
SG5-14a	SG5	SG5-14	*Lobaria macaronesica*	10604	-17,25662187	28,13067098	La Gomera	Canary Isl.
SG5-14b	SG5	SG5-14	*Lobaria macaronesica*	10605	-17,25662187	28,13067098	La Gomera	Canary Isl.
SG5-14d	SG5	SG5-14	*Lobaria macaronesica*	10607	-17,25662187	28,13067098	La Gomera	Canary Isl.
SG5-14e	SG5	SG5-14	*Lobaria macaronesica*	10608	-17,25662187	28,13067098	La Gomera	Canary Isl.

ID	Pop	PopTree	Species	VoucherID	X	Y	Location	Area
SG5-14f	SG5	SG5-14	*Lobaria macaronesica*	10609	-17,25662187	28,13067098	La Gomera	Canary Isl.
SG5-15c	SG5	SG5-15	*Lobaria macaronesica*	10612	-17,25671684	28,13063855	La Gomera	Canary Isl.
SG5-15e	SG5	SG5-15	*Lobaria macaronesica*	10614	-17,25671684	28,13063855	La Gomera	Canary Isl.
SG5-15g	SG5	SG5-15	*Lobaria macaronesica*	10616	-17,25671684	28,13063855	La Gomera	Canary Isl.
SG5-16b	SG5	SG5-16	*Lobaria macaronesica*	10618	-17,25666935	28,13065476	La Gomera	Canary Isl.
SG5-16c	SG5	SG5-16	*Lobaria macaronesica*	10619	-17,25666935	28,13065476	La Gomera	Canary Isl.
SG5-16d	SG5	SG5-16	*Lobaria macaronesica*	10620	-17,25666935	28,13065476	La Gomera	Canary Isl.
SG5-17a	SG5	SG5-17	*Lobaria macaronesica*	10623	-17,25653576	28,13058928	La Gomera	Canary Isl.
SG5-17c	SG5	SG5-17	*Lobaria macaronesica*	10625	-17,25653576	28,13058928	La Gomera	Canary Isl.
SG5-17d	SG5	SG5-17	*Lobaria macaronesica*	10626	-17,25653576	28,13058928	La Gomera	Canary Isl.
SG5-19c	SG5	SG5-19	*Lobaria macaronesica*	10634	-17,25651508	28,130574	La Gomera	Canary Isl.
SG5-02c	SG5	SG5-2	*Lobaria macaronesica*	10562	-17,25632723	28,13097886	La Gomera	Canary Isl.
SG5-02d	SG5	SG5-2	*Lobaria macaronesica*	10563	-17,25632723	28,13097886	La Gomera	Canary Isl.
SG5-02e	SG5	SG5-2	*Lobaria macaronesica*	10564	-17,25632723	28,13097886	La Gomera	Canary Isl.
SG5-20c	SG5	SG5-20	*Lobaria macaronesica*	10637	-17,25661561	28,13058801	La Gomera	Canary Isl.
SG5-20d	SG5	SG5-20	*Lobaria macaronesica*	10638	-17,25661561	28,13058801	La Gomera	Canary Isl.
SG5-20e	SG5	SG5-20	*Lobaria macaronesica*	10639	-17,25661561	28,13058801	La Gomera	Canary Isl.
SG5-21a	SG5	SG5-21	*Lobaria macaronesica*	10640	-17,25658219	28,13055401	La Gomera	Canary Isl.
SG5-21c	SG5	SG5-21	*Lobaria macaronesica*	10642	-17,25658219	28,13055401	La Gomera	Canary Isl.
SG5-21d	SG5	SG5-21	*Lobaria macaronesica*	10643	-17,25658219	28,13055401	La Gomera	Canary Isl.
SG5-22e	SG5	SG5-22	*Lobaria macaronesica*	10648	-17,25650303	28,13049731	La Gomera	Canary Isl.
SG5-22f	SG5	SG5-22	*Lobaria macaronesica*	10649	-17,25650303	28,13049731	La Gomera	Canary Isl.
SG5-22g	SG5	SG5-22	*Lobaria macaronesica*	10650	-17,25650303	28,13049731	La Gomera	Canary Isl.
SG5-24c	SG5	SG5-24	*Lobaria macaronesica*	10654	-17,25629027	28,13037514	La Gomera	Canary Isl.
SG5-24d	SG5	SG5-24	*Lobaria macaronesica*	10655	-17,25629027	28,13037514	La Gomera	Canary Isl.
SG5-24f	SG5	SG5-24	*Lobaria macaronesica*	10657	-17,25629027	28,13037514	La Gomera	Canary Isl.
SG5-03	SG5	SG5-3	*Lobaria macaronesica*	10566	-17,25632391	28,13097033	La Gomera	Canary Isl.

ID	Pop	PopTree	Species	VoucherID	X	Y	Location	Area
SG5-04b	SG5	SG5-4	*Lobaria macaronesica*	10568	-17,25636922	28,13094981	La Gomera	Canary Isl.
SG5-04c	SG5	SG5-4	*Lobaria macaronesica*	10569	-17,25636922	28,13094981	La Gomera	Canary Isl.
SG5-04d	SG5	SG5-4	*Lobaria macaronesica*	10570	-17,25636922	28,13094981	La Gomera	Canary Isl.
SG5-04e	SG5	SG5-4	*Lobaria macaronesica*	10571	-17,25636922	28,13094981	La Gomera	Canary Isl.
SG5-05b	SG5	SG5-5	*Lobaria macaronesica*	10573	-17,25636282	28,13095682	La Gomera	Canary Isl.
SG5-06b	SG5	SG5-6	*Lobaria macaronesica*	10575	-17,25635232	28,13094137	La Gomera	Canary Isl.
SG5-07b	SG5	SG5-7	*Lobaria macaronesica*	10577	-17,25640156	28,13086242	La Gomera	Canary Isl.
SG5-08a	SG5	SG5-8	*Lobaria macaronesica*	10579	-17,25642618	28,13082294	La Gomera	Canary Isl.
SG5-08b	SG5	SG5-8	*Lobaria macaronesica*	10580	-17,25642618	28,13082294	La Gomera	Canary Isl.
SG5-08d	SG5	SG5-8	*Lobaria macaronesica*	10582	-17,25642618	28,13082294	La Gomera	Canary Isl.
SG5-08e	SG5	SG5-8	*Lobaria macaronesica*	10583	-17,25642618	28,13082294	La Gomera	Canary Isl.
SG5-09a	SG5	SG5-9	*Lobaria macaronesica*	10584	-17,25643093	28,13082132	La Gomera	Canary Isl.
SG5-09b	SG5	SG5-9	*Lobaria macaronesica*	10585	-17,25643093	28,13082132	La Gomera	Canary Isl.
SG5-09c	SG5	SG5-9	*Lobaria macaronesica*	10586	-17,25643093	28,13082132	La Gomera	Canary Isl.
SG5-09d	SG5	SG5-9	*Lobaria macaronesica*	10587	-17,25643093	28,13082132	La Gomera	Canary Isl.
SH1-11c	SH1	SH1-11	*Lobaria macaronesica*	10694	-17,98015481	27,76145048	El Hierro	Canary Isl.
SH1-15b	SH1	SH1-15	*Lobaria macaronesica*	10712	-17,97985882	27,7616299	El Hierro	Canary Isl.
SH1-16d	SH1	SH1-16	*Lobaria macaronesica*	10716	-17,9796772	27,76171006	El Hierro	Canary Isl.
SH1-16e	SH1	SH1-16	*Lobaria macaronesica*	10717	-17,9796772	27,76171006	El Hierro	Canary Isl.
SH1-18c	SH1	SH1-18	*Lobaria macaronesica*	10724	-17,97945066	27,7612833	El Hierro	Canary Isl.
SH1-19d	SH1	SH1-19	*Lobaria macaronesica*	10730	-17,97948101	27,76128501	El Hierro	Canary Isl.
SH1-19e	SH1	SH1-19	*Lobaria macaronesica*	10731	-17,97948101	27,76128501	El Hierro	Canary Isl.
SH1-21d	SH1	SH1-21	*Lobaria macaronesica*	10739	-17,9796004	27,76107082	El Hierro	Canary Isl.
SH1-21e	SH1	SH1-21	*Lobaria macaronesica*	10740	-17,9796004	27,76107082	El Hierro	Canary Isl.
SH1-21f	SH1	SH1-21	*Lobaria macaronesica*	10741	-17,9796004	27,76107082	El Hierro	Canary Isl.
SH1-21g	SH1	SH1-21	*Lobaria macaronesica*	10742	-17,9796004	27,76107082	El Hierro	Canary Isl.
SH1-22c	SH1	SH1-22	*Lobaria macaronesica*	10745	-17,97966054	27,76107892	El Hierro	Canary Isl.

ID	Pop	PopTree	Species	VoucherID	X	Y	Location	Area
SH1-03f	SH1	SH1-3	*Lobaria macaronesica*	11296	-17,98080092	27,76117223	El Hierro	Canary Isl.
SH1-04a	SH1	SH1-4	*Lobaria macaronesica*	10671	-17,98077076	27,76117567	El Hierro	Canary Isl.
SH1-08c	SH1	SH1-8	*Lobaria macaronesica*	10683	-17,98047179	27,76130239	El Hierro	Canary Isl.
SH1-08d	SH1	SH1-8	*Lobaria macaronesica*	10684	-17,98047179	27,76130239	El Hierro	Canary Isl.
SH2-16b	SH2	SH2-16	*Lobaria macaronesica*	10796	-17,98684819	27,74277821	El Hierro	Canary Isl.
SM1-01c	SM1	SM1-1	*Lobaria macaronesica*	14705	-25,78249	37,85132	Sao Miguel	Azores
SM1-01d	SM1	SM1-1	*Lobaria macaronesica*	14706	-25,78249	37,85132	Sao Miguel	Azores
SM1-01g	SM1	SM1-1	*Lobaria macaronesica*	14709	-25,78249	37,85132	Sao Miguel	Azores
SM1-10c	SM1	SM1-10	*Lobaria macaronesica*	14757	-25,77994767	37,84954579	Sao Miguel	Azores
SM1-10d	SM1	SM1-10	*Lobaria macaronesica*	14758	-25,77994767	37,84954579	Sao Miguel	Azores
SM1-10e	SM1	SM1-10	*Lobaria macaronesica*	14759	-25,77994767	37,84954579	Sao Miguel	Azores
SM1-11a	SM1	SM1-11	*Lobaria macaronesica*	14760	-25,77995287	37,8495538	Sao Miguel	Azores
SM1-11c	SM1	SM1-11	*Lobaria macaronesica*	14762	-25,77995287	37,8495538	Sao Miguel	Azores
SM1-11d	SM1	SM1-11	*Lobaria macaronesica*	14763	-25,77995287	37,8495538	Sao Miguel	Azores
SM1-11e	SM1	SM1-11	*Lobaria macaronesica*	14764	-25,77995287	37,8495538	Sao Miguel	Azores
SM1-11f	SM1	SM1-11	*Lobaria macaronesica*	14765	-25,77995287	37,8495538	Sao Miguel	Azores
SM1-11g	SM1	SM1-11	*Lobaria macaronesica*	14766	-25,77995287	37,8495538	Sao Miguel	Azores
SM1-12c	SM1	SM1-12	*Lobaria macaronesica*	14769	-25,77989887	37,84960013	Sao Miguel	Azores
SM1-12d	SM1	SM1-12	*Lobaria macaronesica*	14770	-25,77989887	37,84960013	Sao Miguel	Azores
SM1-13a	SM1	SM1-13	*Lobaria macaronesica*	14772	-25,77984195	37,85004846	Sao Miguel	Azores
SM1-13b	SM1	SM1-13	*Lobaria macaronesica*	14773	-25,77984195	37,85004846	Sao Miguel	Azores
SM1-13c	SM1	SM1-13	*Lobaria macaronesica*	14774	-25,77984195	37,85004846	Sao Miguel	Azores
SM1-13d	SM1	SM1-13	*Lobaria macaronesica*	14775	-25,77984195	37,85004846	Sao Miguel	Azores
SM1-14c	SM1	SM1-14	*Lobaria macaronesica*	14778	-25,77986069	37,850091	Sao Miguel	Azores
SM1-14d	SM1	SM1-14	*Lobaria macaronesica*	14779	-25,77986069	37,850091	Sao Miguel	Azores
SM1-14e	SM1	SM1-14	*Lobaria macaronesica*	14780	-25,77986069	37,850091	Sao Miguel	Azores
SM1-14i	SM1	SM1-14	*Lobaria macaronesica*	14784	-25,77986069	37,850091	Sao Miguel	Azores

ID	Pop	PopTree	Species	VoucherID	X	Y	Location	Area
SM1-14j	SM1	SM1-14	*Lobaria macaronesica*	14785	-25,77986069	37,850091	Sao Miguel	Azores
SM1-15a	SM1	SM1-15	*Lobaria macaronesica*	14786	-25,77995407	37,85014236	Sao Miguel	Azores
SM1-15b	SM1	SM1-15	*Lobaria macaronesica*	14787	-25,77995407	37,85014236	Sao Miguel	Azores
SM1-15c	SM1	SM1-15	*Lobaria macaronesica*	14788	-25,77995407	37,85014236	Sao Miguel	Azores
SM1-15d	SM1	SM1-15	*Lobaria macaronesica*	14789	-25,77995407	37,85014236	Sao Miguel	Azores
SM1-15e	SM1	SM1-15	*Lobaria macaronesica*	14790	-25,77995407	37,85014236	Sao Miguel	Azores
SM1-16c	SM1	SM1-16	*Lobaria macaronesica*	14797	-25,77921261	37,85037101	Sao Miguel	Azores
SM1-16g	SM1	SM1-16	*Lobaria macaronesica*	14801	-25,77921261	37,85037101	Sao Miguel	Azores
SM1-16h	SM1	SM1-16	*Lobaria macaronesica*	14802	-25,77921261	37,85037101	Sao Miguel	Azores
SM1-17c	SM1	SM1-17	*Lobaria macaronesica*	14805	-25,77922983	37,85038278	Sao Miguel	Azores
SM1-17d	SM1	SM1-17	*Lobaria macaronesica*	14806	-25,77922983	37,85038278	Sao Miguel	Azores
SM1-17e	SM1	SM1-17	*Lobaria macaronesica*	14807	-25,77922983	37,85038278	Sao Miguel	Azores
SM1-17f	SM1	SM1-17	*Lobaria macaronesica*	14808	-25,77922983	37,85038278	Sao Miguel	Azores
SM1-18a	SM1	SM1-18	*Lobaria macaronesica*	14809	-25,77922953	37,8504008	Sao Miguel	Azores
SM1-18b	SM1	SM1-18	*Lobaria macaronesica*	14810	-25,77922953	37,8504008	Sao Miguel	Azores
SM1-19a	SM1	SM1-19	*Lobaria macaronesica*	14811	-25,77917344	37,85040804	Sao Miguel	Azores
SM1-19b	SM1	SM1-19	*Lobaria macaronesica*	14812	-25,77917344	37,85040804	Sao Miguel	Azores
SM1-19c	SM1	SM1-19	*Lobaria macaronesica*	14813	-25,77917344	37,85040804	Sao Miguel	Azores
SM1-19d	SM1	SM1-19	*Lobaria macaronesica*	14814	-25,77917344	37,85040804	Sao Miguel	Azores
SM1-02d	SM1	SM1-2	*Lobaria macaronesica*	14713	-25,78244388	37,85129367	Sao Miguel	Azores
SM1-02e	SM1	SM1-2	*Lobaria macaronesica*	14714	-25,78244388	37,85129367	Sao Miguel	Azores
SM1-02f	SM1	SM1-2	*Lobaria macaronesica*	14715	-25,78244388	37,85129367	Sao Miguel	Azores
SM1-02g	SM1	SM1-2	*Lobaria macaronesica*	14716	-25,78244388	37,85129367	Sao Miguel	Azores
SM1-02h	SM1	SM1-2	*Lobaria macaronesica*	14717	-25,78244388	37,85129367	Sao Miguel	Azores
SM1-20e	SM1	SM1-20	*Lobaria macaronesica*	14819	-25,77927359	37,85048144	Sao Miguel	Azores
SM1-20f	SM1	SM1-20	*Lobaria macaronesica*	14820	-25,77927359	37,85048144	Sao Miguel	Azores
SM1-20g	SM1	SM1-20	*Lobaria macaronesica*	14821	-25,77927359	37,85048144	Sao Miguel	Azores

**ID**	**Pop**	**PopTree**	**Species**	**VoucherID**	**X**	**Y**	**Location**	**Area**
SM1-03a	SM1	SM1-3	*Lobaria macaronesica*	14718	-25,7823462	37,8512476	Sao Miguel	Azores
SM1-03b	SM1	SM1-3	*Lobaria macaronesica*	14719	-25,7823462	37,8512476	Sao Miguel	Azores
SM1-03c	SM1	SM1-3	*Lobaria macaronesica*	14720	-25,7823462	37,8512476	Sao Miguel	Azores
SM1-03d	SM1	SM1-3	*Lobaria macaronesica*	14721	-25,7823462	37,8512476	Sao Miguel	Azores
SM1-03e	SM1	SM1-3	*Lobaria macaronesica*	14722	-25,7823462	37,8512476	Sao Miguel	Azores
SM1-03f	SM1	SM1-3	*Lobaria macaronesica*	14723	-25,7823462	37,8512476	Sao Miguel	Azores
SM1-03g	SM1	SM1-3	*Lobaria macaronesica*	14724	-25,7823462	37,8512476	Sao Miguel	Azores
SM1-03h	SM1	SM1-3	*Lobaria macaronesica*	14725	-25,7823462	37,8512476	Sao Miguel	Azores
SM1-04a	SM1	SM1-4	*Lobaria macaronesica*	14726	-25,78203	37,85154	Sao Miguel	Azores
SM1-04b	SM1	SM1-4	*Lobaria macaronesica*	14727	-25,78203	37,85154	Sao Miguel	Azores
SM1-04c	SM1	SM1-4	*Lobaria macaronesica*	14728	-25,78203	37,85154	Sao Miguel	Azores
SM1-04f	SM1	SM1-4	*Lobaria macaronesica*	14731	-25,78203	37,85154	Sao Miguel	Azores
SM1-05a	SM1	SM1-5	*Lobaria macaronesica*	14732	-25,78244	37,85155	Sao Miguel	Azores
SM1-05d	SM1	SM1-5	*Lobaria macaronesica*	14735	-25,78244	37,85155	Sao Miguel	Azores
SM1-07c	SM1	SM1-7	*Lobaria macaronesica*	14741	-25,78158	37,85164	Sao Miguel	Azores
SM1-07d	SM1	SM1-7	*Lobaria macaronesica*	14742	-25,78158	37,85164	Sao Miguel	Azores
SM1-07f	SM1	SM1-7	*Lobaria macaronesica*	14744	-25,78158	37,85164	Sao Miguel	Azores
SM1-07g	SM1	SM1-7	*Lobaria macaronesica*	14745	-25,78158	37,85164	Sao Miguel	Azores
SM1-09e	SM1	SM1-9	*Lobaria macaronesica*	14752	-25,77992	37,84953	Sao Miguel	Azores
SM2-01a	SM2	SM2-1	*Lobaria macaronesica*	14823	-25,32485	37,75711	Sao Miguel	Azores
SM2-01b	SM2	SM2-1	*Lobaria macaronesica*	14824	-25,32485	37,75711	Sao Miguel	Azores
SM2-01c	SM2	SM2-1	*Lobaria macaronesica*	14825	-25,32485	37,75711	Sao Miguel	Azores
SM2-14a	SM2	SM2-14	*Lobaria macaronesica*	14873	-25,32551488	37,75565951	Sao Miguel	Azores
SM2-14b	SM2	SM2-14	*Lobaria macaronesica*	14874	-25,32551488	37,75565951	Sao Miguel	Azores
SM2-14d	SM2	SM2-14	*Lobaria macaronesica*	14876	-25,32551488	37,75565951	Sao Miguel	Azores
SM2-17a	SM2	SM2-17	*Lobaria macaronesica*	14888	-25,32533	37,75440999	Sao Miguel	Azores
SM2-20c	SM2	SM2-20	*Lobaria macaronesica*	14905	-25,32571252	37,75370743	Sao Miguel	Azores

ID	Pop	PopTree	Species	VoucherID	X	Y	Location	Area
SM2-21d	SM2	SM2-21	*Lobaria macaronesica*	14910	-25,32576584	37,75362789	Sao Miguel	Azores
SM2-21e	SM2	SM2-21	*Lobaria macaronesica*	14911	-25,32576584	37,75362789	Sao Miguel	Azores
SM2-21f	SM2	SM2-21	*Lobaria macaronesica*	14912	-25,32576584	37,75362789	Sao Miguel	Azores
SM2-21g	SM2	SM2-21	*Lobaria macaronesica*	14913	-25,32576584	37,75362789	Sao Miguel	Azores
SM2-22e	SM2	SM2-22	*Lobaria macaronesica*	14918	-25,32579074	37,7535874	Sao Miguel	Azores
SM2-22f	SM2	SM2-22	*Lobaria macaronesica*	14919	-25,32579074	37,7535874	Sao Miguel	Azores
SM2-22g	SM2	SM2-22	*Lobaria macaronesica*	14920	-25,32579074	37,7535874	Sao Miguel	Azores
SM2-23b	SM2	SM2-23	*Lobaria macaronesica*	14922	-25,32586543	37,75346595	Sao Miguel	Azores
SM2-23c	SM2	SM2-23	*Lobaria macaronesica*	14923	-25,32586543	37,75346595	Sao Miguel	Azores
SM2-24d	SM2	SM2-24	*Lobaria macaronesica*	14927	-25,32589133	37,75340631	Sao Miguel	Azores
SM2-25b	SM2	SM2-25	*Lobaria macaronesica*	14929	-25,32595399	37,7532331	Sao Miguel	Azores
SM2-25d	SM2	SM2-25	*Lobaria macaronesica*	14931	-25,32595399	37,7532331	Sao Miguel	Azores
SM2-26a	SM2	SM2-26	*Lobaria macaronesica*	14932	-25,32597186	37,75309869	Sao Miguel	Azores
SM2-26b	SM2	SM2-26	*Lobaria macaronesica*	14933	-25,32597186	37,75309869	Sao Miguel	Azores
SM2-26c	SM2	SM2-26	*Lobaria macaronesica*	14934	-25,32597186	37,75309869	Sao Miguel	Azores
SM2-26d	SM2	SM2-26	*Lobaria macaronesica*	14935	-25,32597186	37,75309869	Sao Miguel	Azores
SM2-27a	SM2	SM2-27	*Lobaria macaronesica*	14936	-25,32603452	37,75292547	Sao Miguel	Azores
SM2-27b	SM2	SM2-27	*Lobaria macaronesica*	14937	-25,32603452	37,75292547	Sao Miguel	Azores
SM2-27c	SM2	SM2-27	*Lobaria macaronesica*	14938	-25,32603452	37,75292547	Sao Miguel	Azores
SM2-28a	SM2	SM2-28	*Lobaria macaronesica*	14939	-25,32603949	37,75288059	Sao Miguel	Azores
SM2-28b	SM2	SM2-28	*Lobaria macaronesica*	14940	-25,32603949	37,75288059	Sao Miguel	Azores
SM2-28c	SM2	SM2-28	*Lobaria macaronesica*	14941	-25,32603949	37,75288059	Sao Miguel	Azores
SM2-03a	SM2	SM2-3	*Lobaria macaronesica*	14828	-25,32495912	37,75685394	Sao Miguel	Azores
SM2-03b	SM2	SM2-3	*Lobaria macaronesica*	14829	-25,32495912	37,75685394	Sao Miguel	Azores
SM2-03c	SM2	SM2-3	*Lobaria macaronesica*	14830	-25,32495912	37,75685394	Sao Miguel	Azores
SM2-03e	SM2	SM2-3	*Lobaria macaronesica*	14951	-25,32495912	37,75685394	Sao Miguel	Azores
SM2-30a	SM2	SM2-30	*Lobaria macaronesica*	14948	-25,32584	37,75173	Sao Miguel	Azores

ID	Pop	PopTree	Species	VoucherID	X	Y	Location	Area
SM2-30b	SM2	SM2-30	*Lobaria macaronesica*	14949	-25,32584	37,75173	Sao Miguel	Azores
SM2-04a	SM2	SM2-4	*Lobaria macaronesica*	14832	-25,32505872	37,756692	Sao Miguel	Azores
SM2-04b	SM2	SM2-4	*Lobaria macaronesica*	14833	-25,32505872	37,756692	Sao Miguel	Azores
SM2-04c	SM2	SM2-4	*Lobaria macaronesica*	14834	-25,32505872	37,756692	Sao Miguel	Azores
SM2-04d	SM2	SM2-4	*Lobaria macaronesica*	14835	-25,32505872	37,756692	Sao Miguel	Azores
SM2-04e	SM2	SM2-4	*Lobaria macaronesica*	14836	-25,32505872	37,756692	Sao Miguel	Azores
SM2-04f	SM2	SM2-4	*Lobaria macaronesica*	14837	-25,32505872	37,756692	Sao Miguel	Azores
SM2-09b	SM2	SM2-9	*Lobaria macaronesica*	14859	-25,32513889	37,75635122	Sao Miguel	Azores
SP1-13f	SP1	SP1-13	*Lobaria macaronesica*	11638	-17,83522995	28,61173835	La Palma	Canary Isl.
SP1-17d	SP1	SP1-17	*Lobaria macaronesica*	11654	-17,83513288	28,61157011	La Palma	Canary Isl.
SP1-17e	SP1	SP1-17	*Lobaria macaronesica*	11655	-17,83513288	28,61157011	La Palma	Canary Isl.
SP1-17f	SP1	SP1-17	*Lobaria macaronesica*	11656	-17,83513288	28,61157011	La Palma	Canary Isl.
SP1-17g	SP1	SP1-17	*Lobaria macaronesica*	11657	-17,83513288	28,61157011	La Palma	Canary Isl.
SP1-02b	SP1	SP1-2	*Lobaria macaronesica*	11588	-17,83512633	28,61215728	La Palma	Canary Isl.
SP1-20e	SP1	SP1-20	*Lobaria macaronesica*	11672	-17,83510203	28,61131411	La Palma	Canary Isl.
SP1-20f	SP1	SP1-20	*Lobaria macaronesica*	11673	-17,83510203	28,61131411	La Palma	Canary Isl.
SP1-20g	SP1	SP1-20	*Lobaria macaronesica*	11674	-17,83510203	28,61131411	La Palma	Canary Isl.
SP1-21f	SP1	SP1-21	*Lobaria macaronesica*	11680	-17,83510178	28,6113051	La Palma	Canary Isl.
SP1-21h	SP1	SP1-21	*Lobaria macaronesica*	11682	-17,83510178	28,6113051	La Palma	Canary Isl.
SP1-22d	SP1	SP1-22	*Lobaria macaronesica*	11686	-17,8350973	28,61130293	La Palma	Canary Isl.
SP1-22e	SP1	SP1-22	*Lobaria macaronesica*	11687	-17,8350973	28,61130293	La Palma	Canary Isl.
SP1-23a	SP1	SP1-23	*Lobaria macaronesica*	11688	-17,83507039	28,61128997	La Palma	Canary Isl.
SP1-23b	SP1	SP1-23	*Lobaria macaronesica*	11689	-17,83507039	28,61128997	La Palma	Canary Isl.
SP1-24h	SP1	SP1-24	*Lobaria macaronesica*	11697	-17,83506033	28,61124576	La Palma	Canary Isl.
SP1-25c	SP1	SP1-25	*Lobaria macaronesica*	11700	-17,83505832	28,61123693	La Palma	Canary Isl.
SP1-25g	SP1	SP1-25	*Lobaria macaronesica*	11704	-17,83505832	28,61123693	La Palma	Canary Isl.
SP1-26h	SP1	SP1-26	*Lobaria macaronesica*	11712	-17,83501347	28,61121531	La Palma	Canary Isl.

**ID**	**Pop**	**PopTree**	**Species**	**VoucherID**	**X**	**Y**	**Location**	**Area**
SP1-26i	SP1	SP1-26	*Lobaria macaronesica*	11713	-17,83501347	28,61121531	La Palma	Canary Isl.
SP1-26j	SP1	SP1-26	*Lobaria macaronesica*	11714	-17,83501347	28,61121531	La Palma	Canary Isl.
SP1-27e	SP1	SP1-27	*Lobaria macaronesica*	11721	-17,83498283	28,61121595	La Palma	Canary Isl.
SP1-28b	SP1	SP1-28	*Lobaria macaronesica*	11724	-17,83493231	28,61120917	La Palma	Canary Isl.
SP1-28c	SP1	SP1-28	*Lobaria macaronesica*	11725	-17,83493231	28,61120917	La Palma	Canary Isl.
SP1-28d	SP1	SP1-28	*Lobaria macaronesica*	11726	-17,83493231	28,61120917	La Palma	Canary Isl.
SP1-28e	SP1	SP1-28	*Lobaria macaronesica*	11727	-17,83493231	28,61120917	La Palma	Canary Isl.
SP1-28f	SP1	SP1-28	*Lobaria macaronesica*	11728	-17,83493231	28,61120917	La Palma	Canary Isl.
SP1-28g	SP1	SP1-28	*Lobaria macaronesica*	11729	-17,83493231	28,61120917	La Palma	Canary Isl.
SP1-29d	SP1	SP1-29	*Lobaria macaronesica*	11733	-17,83488747	28,61118756	La Palma	Canary Isl.
SP1-29e	SP1	SP1-29	*Lobaria macaronesica*	11734	-17,83488747	28,61118756	La Palma	Canary Isl.
SP1-29g	SP1	SP1-29	*Lobaria macaronesica*	11736	-17,83488747	28,61118756	La Palma	Canary Isl.
SP1-29h	SP1	SP1-29	*Lobaria macaronesica*	11737	-17,83488747	28,61118756	La Palma	Canary Isl.
SP1-30a	SP1	SP1-30	*Lobaria macaronesica*	11738	-17,83493855	28,61118649	La Palma	Canary Isl.
SP1-30b	SP1	SP1-30	*Lobaria macaronesica*	11739	-17,83493855	28,61118649	La Palma	Canary Isl.
SP1-30c	SP1	SP1-30	*Lobaria macaronesica*	11740	-17,83493855	28,61118649	La Palma	Canary Isl.
SP1-34b	SP1	SP1-34	*Lobaria macaronesica*	11748	-17,83494542	28,61121114	La Palma	Canary Isl.
SP1-34c	SP1	SP1-34	*Lobaria macaronesica*	11749	-17,83494542	28,61121114	La Palma	Canary Isl.
SP1-34d	SP1	SP1-34	*Lobaria macaronesica*	11750	-17,83494542	28,61121114	La Palma	Canary Isl.
SP1-34e	SP1	SP1-34	*Lobaria macaronesica*	11751	-17,83494542	28,61121114	La Palma	Canary Isl.
SP1-04c	SP1	SP1-4	*Lobaria macaronesica*	11596	-17,83507907	28,61208313	La Palma	Canary Isl.
SP1-09d	SP1	SP1-9	*Lobaria macaronesica*	11618	-17,83528004	28,61190727	La Palma	Canary Isl.
SP2-01	SP2	SP2-1	*Lobaria macaronesica*	12098	-17,77786	28,7602	La Palma	Canary Isl.
SP2-10b	SP2	SP2-10	*Lobaria macaronesica*	12123	-17,77805327	28,76091351	La Palma	Canary Isl.
SP2-10d	SP2	SP2-10	*Lobaria macaronesica*	12125	-17,77805327	28,76091351	La Palma	Canary Isl.
SP2-10e	SP2	SP2-10	*Lobaria macaronesica*	12126	-17,77805327	28,76091351	La Palma	Canary Isl.
SP2-10f	SP2	SP2-10	*Lobaria macaronesica*	12127	-17,77805327	28,76091351	La Palma	Canary Isl.

ID	Pop	PopTree	Species	VoucherID	X	Y	Location	Area
SP2-10h	SP2	SP2-10	*Lobaria macaronesica*	12129	-17,77805327	28,76091351	La Palma	Canary Isl.
SP2-10i	SP2	SP2-10	*Lobaria macaronesica*	12130	-17,77805327	28,76091351	La Palma	Canary Isl.
SP2-10j	SP2	SP2-10	*Lobaria macaronesica*	12131	-17,77805327	28,76091351	La Palma	Canary Isl.
SP2-10k	SP2	SP2-10	*Lobaria macaronesica*	12132	-17,77805327	28,76091351	La Palma	Canary Isl.
SP2-10l	SP2	SP2-10	*Lobaria macaronesica*	12133	-17,77805327	28,76091351	La Palma	Canary Isl.
SP2-10m	SP2	SP2-10	*Lobaria macaronesica*	12134	-17,77805327	28,76091351	La Palma	Canary Isl.
SP2-11	SP2	SP2-11	*Lobaria macaronesica*	12135	-17,77807245	28,76089239	La Palma	Canary Isl.
SP2-12a	SP2	SP2-12	*Lobaria macaronesica*	12136	-17,77800061	28,76085778	La Palma	Canary Isl.
SP2-12b	SP2	SP2-12	*Lobaria macaronesica*	12137	-17,77800061	28,76085778	La Palma	Canary Isl.
SP2-12c	SP2	SP2-12	*Lobaria macaronesica*	12138	-17,77800061	28,76085778	La Palma	Canary Isl.
SP2-12d	SP2	SP2-12	*Lobaria macaronesica*	12139	-17,77800061	28,76085778	La Palma	Canary Isl.
SP2-12e	SP2	SP2-12	*Lobaria macaronesica*	12140	-17,77800061	28,76085778	La Palma	Canary Isl.
SP2-12f	SP2	SP2-12	*Lobaria macaronesica*	12141	-17,77800061	28,76085778	La Palma	Canary Isl.
SP2-12g	SP2	SP2-12	*Lobaria macaronesica*	12142	-17,77800061	28,76085778	La Palma	Canary Isl.
SP2-12h	SP2	SP2-12	*Lobaria macaronesica*	12143	-17,77800061	28,76085778	La Palma	Canary Isl.
SP2-13a	SP2	SP2-13	*Lobaria macaronesica*	12144	-17,77799822	28,76076763	La Palma	Canary Isl.
SP2-13b	SP2	SP2-13	*Lobaria macaronesica*	12145	-17,77799822	28,76076763	La Palma	Canary Isl.
SP2-13c	SP2	SP2-13	*Lobaria macaronesica*	12146	-17,77799822	28,76076763	La Palma	Canary Isl.
SP2-14b	SP2	SP2-14	*Lobaria macaronesica*	12148	-17,77798816	28,76072342	La Palma	Canary Isl.
SP2-14c	SP2	SP2-14	*Lobaria macaronesica*	12149	-17,77798816	28,76072342	La Palma	Canary Isl.
SP2-15a	SP2	SP2-15	*Lobaria macaronesica*	12150	-17,77790825	28,76066707	La Palma	Canary Isl.
SP2-15b	SP2	SP2-15	*Lobaria macaronesica*	12151	-17,77790825	28,76066707	La Palma	Canary Isl.
SP2-16	SP2	SP2-16	*Lobaria macaronesica*	12152	-17,77788164	28,76062856	La Palma	Canary Isl.
SP2-17a	SP2	SP2-17	*Lobaria macaronesica*	12153	-17,77780716	28,76046056	La Palma	Canary Isl.
SP2-17b	SP2	SP2-17	*Lobaria macaronesica*	12154	-17,77780716	28,76046056	La Palma	Canary Isl.
SP2-17c	SP2	SP2-17	*Lobaria macaronesica*	12155	-17,77780716	28,76046056	La Palma	Canary Isl.
SP2-17d	SP2	SP2-17	*Lobaria macaronesica*	12156	-17,77780716	28,76046056	La Palma	Canary Isl.

**ID**	**Pop**	**PopTree**	**Species**	**VoucherID**	**X**	**Y**	**Location**	**Area**
SP2-18	SP2	SP2-18	*Lobaria macaronesica*	12157	-17,77780788	28,76048761	La Palma	Canary Isl.
SP2-19a	SP2	SP2-19	*Lobaria macaronesica*	12158	-17,77777408	28,76045376	La Palma	Canary Isl.
SP2-19b	SP2	SP2-19	*Lobaria macaronesica*	12159	-17,77777408	28,76045376	La Palma	Canary Isl.
SP2-19c	SP2	SP2-19	*Lobaria macaronesica*	12160	-17,77777408	28,76045376	La Palma	Canary Isl.
SP2-19d	SP2	SP2-19	*Lobaria macaronesica*	12161	-17,77777408	28,76045376	La Palma	Canary Isl.
SP2-19e	SP2	SP2-19	*Lobaria macaronesica*	12162	-17,77777408	28,76045376	La Palma	Canary Isl.
SP2-02a	SP2	SP2-2	*Lobaria macaronesica*	12099	-17,77776409	28,76030563	La Palma	Canary Isl.
SP2-02b	SP2	SP2-2	*Lobaria macaronesica*	12100	-17,77776409	28,76030563	La Palma	Canary Isl.
SP2-02c	SP2	SP2-2	*Lobaria macaronesica*	12101	-17,77776409	28,76030563	La Palma	Canary Isl.
SP2-02d	SP2	SP2-2	*Lobaria macaronesica*	12102	-17,77776409	28,76030563	La Palma	Canary Isl.
SP2-20a	SP2	SP2-20	*Lobaria macaronesica*	12163	-17,77756945	28,76045797	La Palma	Canary Isl.
SP2-20b	SP2	SP2-20	*Lobaria macaronesica*	12164	-17,77756945	28,76045797	La Palma	Canary Isl.
SP2-20c	SP2	SP2-20	*Lobaria macaronesica*	12165	-17,77756945	28,76045797	La Palma	Canary Isl.
SP2-20d	SP2	SP2-20	*Lobaria macaronesica*	12166	-17,77756945	28,76045797	La Palma	Canary Isl.
SP2-21a	SP2	SP2-21	*Lobaria macaronesica*	12167	-17,77724977	28,76081005	La Palma	Canary Isl.
SP2-21b	SP2	SP2-21	*Lobaria macaronesica*	12168	-17,77724977	28,76081005	La Palma	Canary Isl.
SP2-21c	SP2	SP2-21	*Lobaria macaronesica*	12169	-17,77724977	28,76081005	La Palma	Canary Isl.
SP2-21d	SP2	SP2-21	*Lobaria macaronesica*	12170	-17,77724977	28,76081005	La Palma	Canary Isl.
SP2-21f	SP2	SP2-21	*Lobaria macaronesica*	12172	-17,77724977	28,76081005	La Palma	Canary Isl.
SP2-22a	SP2	SP2-22	*Lobaria macaronesica*	12173	-17,77694874	28,76086323	La Palma	Canary Isl.
SP2-22b	SP2	SP2-22	*Lobaria macaronesica*	12174	-17,77694874	28,76086323	La Palma	Canary Isl.
SP2-22c	SP2	SP2-22	*Lobaria macaronesica*	12175	-17,77694874	28,76086323	La Palma	Canary Isl.
SP2-22d	SP2	SP2-22	*Lobaria macaronesica*	12176	-17,77694874	28,76086323	La Palma	Canary Isl.
SP2-22e	SP2	SP2-22	*Lobaria macaronesica*	12177	-17,77694874	28,76086323	La Palma	Canary Isl.
SP2-22f	SP2	SP2-22	*Lobaria macaronesica*	12178	-17,77694874	28,76086323	La Palma	Canary Isl.
SP2-23a	SP2	SP2-23	*Lobaria macaronesica*	12179	-17,77664522	28,76082248	La Palma	Canary Isl.
SP2-23b	SP2	SP2-23	*Lobaria macaronesica*	12180	-17,77664522	28,76082248	La Palma	Canary Isl.

ID	Pop	PopTree	Species	VoucherID	X	Y	Location	Area
SP2-25a	SP2	SP2-25	*Lobaria macaronesica*	12182	-17,77667795	28,7610822	La Palma	Canary Isl.
SP2-25b	SP2	SP2-25	*Lobaria macaronesica*	12183	-17,77667795	28,7610822	La Palma	Canary Isl.
SP2-25c	SP2	SP2-25	*Lobaria macaronesica*	12184	-17,77667795	28,7610822	La Palma	Canary Isl.
SP2-25d	SP2	SP2-25	*Lobaria macaronesica*	12185	-17,77667795	28,7610822	La Palma	Canary Isl.
SP2-03b	SP2	SP2-3	*Lobaria macaronesica*	12104	-17,77776887	28,76048594	La Palma	Canary Isl.
SP2-03c	SP2	SP2-3	*Lobaria macaronesica*	12105	-17,77776887	28,76048594	La Palma	Canary Isl.
SP2-03d	SP2	SP2-3	*Lobaria macaronesica*	12106	-17,77776887	28,76048594	La Palma	Canary Isl.
SP2-05	SP2	SP2-5	*Lobaria macaronesica*	12109	-17,7777984	28,76092951	La Palma	Canary Isl.
SP2-06a	SP2	SP2-6	*Lobaria macaronesica*	12110	-17,77808928	28,76101608	La Palma	Canary Isl.
SP2-06b	SP2	SP2-6	*Lobaria macaronesica*	12111	-17,77808928	28,76101608	La Palma	Canary Isl.
SP2-07a	SP2	SP2-7	*Lobaria macaronesica*	12112	-17,77805086	28,76104586	La Palma	Canary Isl.
SP2-07b	SP2	SP2-7	*Lobaria macaronesica*	12113	-17,77805086	28,76104586	La Palma	Canary Isl.
SP2-07c	SP2	SP2-7	*Lobaria macaronesica*	12114	-17,77805086	28,76104586	La Palma	Canary Isl.
SP2-08a	SP2	SP2-8	*Lobaria macaronesica*	12115	-17,77802425	28,76100734	La Palma	Canary Isl.
SP2-08b	SP2	SP2-8	*Lobaria macaronesica*	12116	-17,77802425	28,76100734	La Palma	Canary Isl.
SP2-09a	SP2	SP2-9	*Lobaria macaronesica*	12117	-17,77805699	28,7609219	La Palma	Canary Isl.
SP2-09b	SP2	SP2-9	*Lobaria macaronesica*	12118	-17,77805699	28,7609219	La Palma	Canary Isl.
SP2-09c	SP2	SP2-9	*Lobaria macaronesica*	12119	-17,77805699	28,7609219	La Palma	Canary Isl.
SP2-09d	SP2	SP2-9	*Lobaria macaronesica*	12120	-17,77805699	28,7609219	La Palma	Canary Isl.
SP2-09e	SP2	SP2-9	*Lobaria macaronesica*	12121	-17,77805699	28,7609219	La Palma	Canary Isl.
SP3-01a	SP3	SP3-1	*Lobaria macaronesica*	10816	-17,80478	28,78824	La Palma	Canary Isl.
SP3-01b	SP3	SP3-1	*Lobaria macaronesica*	10817	-17,80478	28,78824	La Palma	Canary Isl.
SP3-01c	SP3	SP3-1	*Lobaria macaronesica*	10818	-17,80478	28,78824	La Palma	Canary Isl.
SP3-10b	SP3	SP3-10	*Lobaria macaronesica*	10839	-17,80443947	28,78837818	La Palma	Canary Isl.
SP3-10c	SP3	SP3-10	*Lobaria macaronesica*	10840	-17,80443947	28,78837818	La Palma	Canary Isl.
SP3-10d	SP3	SP3-10	*Lobaria macaronesica*	10841	-17,80443947	28,78837818	La Palma	Canary Isl.
SP3-11a	SP3	SP3-11	*Lobaria macaronesica*	10842	-17,80444149	28,78838702	La Palma	Canary Isl.

ID	Pop	PopTree	Species	VoucherID	X	Y	Location	Area
SP3-11b	SP3	SP3-11	*Lobaria macaronesica*	10843	-17,80444149	28,78838702	La Palma	Canary Isl.
SP3-12a	SP3	SP3-12	*Lobaria macaronesica*	10844	-17,80444681	28,78839472	La Palma	Canary Isl.
SP3-12b	SP3	SP3-12	*Lobaria macaronesica*	10845	-17,80444681	28,78839472	La Palma	Canary Isl.
SP3-15	SP3	SP3-15	*Lobaria macaronesica*	10849	-17,8044243	28,78861045	La Palma	Canary Isl.
SP3-16b	SP3	SP3-16	*Lobaria macaronesica*	10851	-17,80416209	28,78875122	La Palma	Canary Isl.
SP3-17a	SP3	SP3-17	*Lobaria macaronesica*	10852	-17,80375273	28,78875972	La Palma	Canary Isl.
SP3-17b	SP3	SP3-17	*Lobaria macaronesica*	10853	-17,80375273	28,78875972	La Palma	Canary Isl.
SP3-17c	SP3	SP3-17	*Lobaria macaronesica*	10854	-17,80375273	28,78875972	La Palma	Canary Isl.
SP3-18	SP3	SP3-18	*Lobaria macaronesica*	10855	-17,80373673	28,7887485	La Palma	Canary Isl.
SP3-19a	SP3	SP3-19	*Lobaria macaronesica*	10856	-17,80371645	28,78872815	La Palma	Canary Isl.
SP3-02	SP3	SP3-2	*Lobaria macaronesica*	10819	-17,80478	28,78828	La Palma	Canary Isl.
SP3-20a	SP3	SP3-20	*Lobaria macaronesica*	10858	-17,80366878	28,78874456	La Palma	Canary Isl.
SP3-20d	SP3	SP3-20	*Lobaria macaronesica*	10861	-17,80366878	28,78874456	La Palma	Canary Isl.
SP3-21a	SP3	SP3-21	*Lobaria macaronesica*	10862	-17,80364938	28,7887388	La Palma	Canary Isl.
SP3-21b	SP3	SP3-21	*Lobaria macaronesica*	10863	-17,80364938	28,7887388	La Palma	Canary Isl.
SP3-21c	SP3	SP3-21	*Lobaria macaronesica*	10864	-17,80364938	28,7887388	La Palma	Canary Isl.
SP3-21d	SP3	SP3-21	*Lobaria macaronesica*	10865	-17,80364938	28,7887388	La Palma	Canary Isl.
SP3-21e	SP3	SP3-21	*Lobaria macaronesica*	10866	-17,80364938	28,7887388	La Palma	Canary Isl.
SP3-22a	SP3	SP3-22	*Lobaria macaronesica*	10867	-17,80364962	28,78874781	La Palma	Canary Isl.
SP3-22b	SP3	SP3-22	*Lobaria macaronesica*	10868	-17,80364962	28,78874781	La Palma	Canary Isl.
SP3-22c	SP3	SP3-22	*Lobaria macaronesica*	10869	-17,80364962	28,78874781	La Palma	Canary Isl.
SP3-22d	SP3	SP3-22	*Lobaria macaronesica*	10870	-17,80364962	28,78874781	La Palma	Canary Isl.
SP3-22e	SP3	SP3-22	*Lobaria macaronesica*	10871	-17,80364962	28,78874781	La Palma	Canary Isl.
SP3-23e	SP3	SP3-23	*Lobaria macaronesica*	10876	-17,80369873	28,78861965	La Palma	Canary Isl.
SP3-03	SP3	SP3-3	*Lobaria macaronesica*	10820	-17,80461036	28,78819778	La Palma	Canary Isl.
SP3-04a	SP3	SP3-4	*Lobaria macaronesica*	10821	-17,80453356	28,78825731	La Palma	Canary Isl.
SP3-04b	SP3	SP3-4	*Lobaria macaronesica*	10822	-17,80453356	28,78825731	La Palma	Canary Isl.

**ID**	**Pop**	**PopTree**	**Species**	**VoucherID**	**X**	**Y**	**Location**	**Area**
SP3-04c	SP3	SP3-4	*Lobaria macaronesica*	10823	-17,80453356	28,78825731	La Palma	Canary Isl.
SP3-05a	SP3	SP3-5	*Lobaria macaronesica*	10824	-17,80450286	28,78825795	La Palma	Canary Isl.
SP3-05b	SP3	SP3-5	*Lobaria macaronesica*	10825	-17,80450286	28,78825795	La Palma	Canary Isl.
SP3-05c	SP3	SP3-5	*Lobaria macaronesica*	10826	-17,80450286	28,78825795	La Palma	Canary Isl.
SP3-06a	SP3	SP3-6	*Lobaria macaronesica*	10827	-17,8045089	28,78828448	La Palma	Canary Isl.
SP3-06b	SP3	SP3-6	*Lobaria macaronesica*	10828	-17,8045089	28,78828448	La Palma	Canary Isl.
SP3-06c	SP3	SP3-6	*Lobaria macaronesica*	10829	-17,8045089	28,78828448	La Palma	Canary Isl.
SP3-06d	SP3	SP3-6	*Lobaria macaronesica*	10830	-17,8045089	28,78828448	La Palma	Canary Isl.
SP3-07b	SP3	SP3-7	*Lobaria macaronesica*	10832	-17,80450122	28,78829043	La Palma	Canary Isl.
SP3-08b	SP3	SP3-8	*Lobaria macaronesica*	10834	-17,80443728	28,78836087	La Palma	Canary Isl.
SP3-08c	SP3	SP3-8	*Lobaria macaronesica*	10835	-17,80443728	28,78836087	La Palma	Canary Isl.
SP3-09a	SP3	SP3-9	*Lobaria macaronesica*	10836	-17,80443575	28,78836978	La Palma	Canary Isl.
SP3-09b	SP3	SP3-9	*Lobaria macaronesica*	10837	-17,80443575	28,78836978	La Palma	Canary Isl.
SP4-05a	SP4	SP4-5	*Lobaria macaronesica*	11769	-17,85078875	28,80282615	La Palma	Canary Isl.
SP4-05b	SP4	SP4-5	*Lobaria macaronesica*	11770	-17,85078875	28,80282615	La Palma	Canary Isl.
SP5-10f	SP5	SP5-10	*Lobaria macaronesica*	11871	-17,79174995	28,72426494	La Palma	Canary Isl.
SP5-19h	SP5	SP5-19	*Lobaria macaronesica*	11909	-17,79148076	28,72413579	La Palma	Canary Isl.
SP5-23b	SP5	SP5-23	*Lobaria macaronesica*	11925	-17,79119914	28,724078	La Palma	Canary Isl.
SP5-23c	SP5	SP5-23	*Lobaria macaronesica*	11926	-17,79119914	28,724078	La Palma	Canary Isl.
SP5-23i	SP5	SP5-23	*Lobaria macaronesica*	11932	-17,79119914	28,724078	La Palma	Canary Isl.
SP5-23l	SP5	SP5-23	*Lobaria macaronesica*	11935	-17,79119914	28,724078	La Palma	Canary Isl.
SP5-06b	SP5	SP5-6	*Lobaria macaronesica*	11848	-17,79102298	28,72613755	La Palma	Canary Isl.
SP5-07d	SP5	SP5-7	*Lobaria macaronesica*	11853	-17,79151392	28,72485593	La Palma	Canary Isl.
SP5-08e	SP5	SP5-8	*Lobaria macaronesica*	11858	-17,79158751	28,72473723	La Palma	Canary Isl.
SP5-08h	SP5	SP5-8	*Lobaria macaronesica*	11861	-17,79158751	28,72473723	La Palma	Canary Isl.
ST1-11a	ST1	ST1-11	*Lobaria macaronesica*	10906	-16,40794684	28,4203133	Tenerife	Canary Isl.
ST1-11d	ST1	ST1-11	*Lobaria macaronesica*	10909	-16,40794684	28,4203133	Tenerife	Canary Isl.

**ID**	**Pop**	**PopTree**	**Species**	**VoucherID**	**X**	**Y**	**Location**	**Area**
ST1-11e	ST1	ST1-11	*Lobaria macaronesica*	10910	-16,40794684	28,4203133	Tenerife	Canary Isl.
ST1-13b	ST1	ST1-13	*Lobaria macaronesica*	10918	-16,40794169	28,42025291	Tenerife	Canary Isl.
ST1-13c	ST1	ST1-13	*Lobaria macaronesica*	10919	-16,40794169	28,42025291	Tenerife	Canary Isl.
ST1-13d	ST1	ST1-13	*Lobaria macaronesica*	10920	-16,40794169	28,42025291	Tenerife	Canary Isl.
ST1-13e	ST1	ST1-13	*Lobaria macaronesica*	10921	-16,40794169	28,42025291	Tenerife	Canary Isl.
ST1-14b	ST1	ST1-14	*Lobaria macaronesica*	10924	-16,40800046	28,42026814	Tenerife	Canary Isl.
ST1-21d	ST1	ST1-21	*Lobaria macaronesica*	10948	-16,40762485	28,41983316	Tenerife	Canary Isl.
ST1-21e	ST1	ST1-21	*Lobaria macaronesica*	10949	-16,40762485	28,41983316	Tenerife	Canary Isl.
ST2-01h	ST2	ST2-1	*Lobaria macaronesica*	10960	-16,81055	28,32856	Tenerife	Canary Isl.
ST2-10d	ST2	ST2-10	*Lobaria macaronesica*	10998	-16,81061959	28,32874269	Tenerife	Canary Isl.
ST2-12a	ST2	ST2-12	*Lobaria macaronesica*	11006	-16,81063687	28,32875786	Tenerife	Canary Isl.
ST2-13d	ST2	ST2-13	*Lobaria macaronesica*	11014	-16,81064688	28,32875616	Tenerife	Canary Isl.
ST2-13e	ST2	ST2-13	*Lobaria macaronesica*	11015	-16,81064688	28,32875616	Tenerife	Canary Isl.
ST2-16d	ST2	ST2-16	*Lobaria macaronesica*	11026	-16,81065258	28,32866716	Tenerife	Canary Isl.
ST2-16e	ST2	ST2-16	*Lobaria macaronesica*	11027	-16,81065258	28,32866716	Tenerife	Canary Isl.
ST2-16f	ST2	ST2-16	*Lobaria macaronesica*	11028	-16,81065258	28,32866716	Tenerife	Canary Isl.
ST2-16g	ST2	ST2-16	*Lobaria macaronesica*	11029	-16,81065258	28,32866716	Tenerife	Canary Isl.
ST2-18a	ST2	ST2-18	*Lobaria macaronesica*	11035	-16,8106411	28,32854343	Tenerife	Canary Isl.
ST2-18g	ST2	ST2-18	*Lobaria macaronesica*	11041	-16,8106411	28,32854343	Tenerife	Canary Isl.
ST2-19f	ST2	ST2-19	*Lobaria macaronesica*	11047	-16,81064606	28,32853555	Tenerife	Canary Isl.
ST2-20g	ST2	ST2-20	*Lobaria macaronesica*	11297	-16,81055077	28,32856768	Tenerife	Canary Isl.
ST2-24b	ST2	ST2-24	*Lobaria macaronesica*	11067	-16,8105849	28,3284459	Tenerife	Canary Isl.
ST2-24c	ST2	ST2-24	*Lobaria macaronesica*	11068	-16,8105849	28,3284459	Tenerife	Canary Isl.
ST2-26h	ST2	ST2-26	*Lobaria macaronesica*	11084	-16,81043884	28,32841651	Tenerife	Canary Isl.
ST2-27a	ST2	ST2-27	*Lobaria macaronesica*	11087	-16,81033816	28,32840217	Tenerife	Canary Isl.
ST2-08e	ST2	ST2-8	*Lobaria macaronesica*	10988	-16,81058851	28,32873812	Tenerife	Canary Isl.
ST2-08f	ST2	ST2-8	*Lobaria macaronesica*	10989	-16,81058851	28,32873812	Tenerife	Canary Isl.

**ID**	**Pop**	**PopTree**	**Species**	**VoucherID**	**X**	**Y**	**Location**	**Area**
ST2-08g	ST2	ST2-8	*Lobaria macaronesica*	10990	-16,81058851	28,32873812	Tenerife	Canary Isl.
ST2-09c	ST2	ST2-9	*Lobaria macaronesica*	10993	-16,81056178	28,32872494	Tenerife	Canary Isl.
ST4-01a	ST4	ST4-1	*Lobaria macaronesica*	11384	-16,27123	28,5398	Tenerife	Canary Isl.
ST4-01b	ST4	ST4-1	*Lobaria macaronesica*	11385	-16,27123	28,5398	Tenerife	Canary Isl.
ST4-01c	ST4	ST4-1	*Lobaria macaronesica*	11386	-16,27123	28,5398	Tenerife	Canary Isl.
ST4-10e	ST4	ST4-10	*Lobaria macaronesica*	11415	-16,27077282	28,53977081	Tenerife	Canary Isl.
ST4-10f	ST4	ST4-10	*Lobaria macaronesica*	11416	-16,27077282	28,53977081	Tenerife	Canary Isl.
ST4-11b	ST4	ST4-11	*Lobaria macaronesica*	11418	-16,27081231	28,53979945	Tenerife	Canary Isl.
ST4-12d	ST4	ST4-12	*Lobaria macaronesica*	11422	-16,2708188	28,53979247	Tenerife	Canary Isl.
ST4-12e	ST4	ST4-12	*Lobaria macaronesica*	11423	-16,2708188	28,53979247	Tenerife	Canary Isl.
ST4-13d	ST4	ST4-13	*Lobaria macaronesica*	11428	-16,27083661	28,53980133	Tenerife	Canary Isl.
ST4-13e	ST4	ST4-13	*Lobaria macaronesica*	11429	-16,27083661	28,53980133	Tenerife	Canary Isl.
ST4-13f	ST4	ST4-13	*Lobaria macaronesica*	11430	-16,27083661	28,53980133	Tenerife	Canary Isl.
ST4-14a	ST4	ST4-14	*Lobaria macaronesica*	11431	-16,27083827	28,53979242	Tenerife	Canary Isl.
ST4-14b	ST4	ST4-14	*Lobaria macaronesica*	11432	-16,27083827	28,53979242	Tenerife	Canary Isl.
ST4-14c	ST4	ST4-14	*Lobaria macaronesica*	11433	-16,27083827	28,53979242	Tenerife	Canary Isl.
ST4-15a	ST4	ST4-15	*Lobaria macaronesica*	11434	-16,27082863	28,53978943	Tenerife	Canary Isl.
ST4-15b	ST4	ST4-15	*Lobaria macaronesica*	11435	-16,27082863	28,53978943	Tenerife	Canary Isl.
ST4-16b	ST4	ST4-16	*Lobaria macaronesica*	11437	-16,2708965	28,53961917	Tenerife	Canary Isl.
ST4-16c	ST4	ST4-16	*Lobaria macaronesica*	11438	-16,2708965	28,53961917	Tenerife	Canary Isl.
ST4-17d	ST4	ST4-17	*Lobaria macaronesica*	11442	-16,27074582	28,53964409	Tenerife	Canary Isl.
ST4-17e	ST4	ST4-17	*Lobaria macaronesica*	11443	-16,27074582	28,53964409	Tenerife	Canary Isl.
ST4-17f	ST4	ST4-17	*Lobaria macaronesica*	11444	-16,27074582	28,53964409	Tenerife	Canary Isl.
ST4-18a	ST4	ST4-18	*Lobaria macaronesica*	11445	-16,27081068	28,53957435	Tenerife	Canary Isl.
ST4-18b	ST4	ST4-18	*Lobaria macaronesica*	11446	-16,27081068	28,53957435	Tenerife	Canary Isl.
ST4-18c	ST4	ST4-18	*Lobaria macaronesica*	11447	-16,27081068	28,53957435	Tenerife	Canary Isl.
ST4-19b	ST4	ST4-19	*Lobaria macaronesica*	11449	-16,27080403	28,53956749	Tenerife	Canary Isl.

ID	Pop	PopTree	Species	VoucherID	X	Y	Location	Area
ST4-19c	ST4	ST4-19	*Lobaria macaronesica*	11450	-16,27080403	28,53956749	Tenerife	Canary Isl.
ST4-19d	ST4	ST4-19	*Lobaria macaronesica*	11451	-16,27080403	28,53956749	Tenerife	Canary Isl.
ST4-02a	ST4	ST4-2	*Lobaria macaronesica*	11387	-16,27107932	28,53982492	Tenerife	Canary Isl.
ST4-02b	ST4	ST4-2	*Lobaria macaronesica*	11388	-16,27107932	28,53982492	Tenerife	Canary Isl.
ST4-02c	ST4	ST4-2	*Lobaria macaronesica*	11389	-16,27107932	28,53982492	Tenerife	Canary Isl.
ST4-02d	ST4	ST4-2	*Lobaria macaronesica*	11390	-16,27107932	28,53982492	Tenerife	Canary Isl.
ST4-20a	ST4	ST4-20	*Lobaria macaronesica*	11452	-16,27084801	28,53954451	Tenerife	Canary Isl.
ST4-20b	ST4	ST4-20	*Lobaria macaronesica*	11453	-16,27084801	28,53954451	Tenerife	Canary Isl.
ST4-20c	ST4	ST4-20	*Lobaria macaronesica*	11454	-16,27084801	28,53954451	Tenerife	Canary Isl.
ST4-20d	ST4	ST4-20	*Lobaria macaronesica*	11455	-16,27084801	28,53954451	Tenerife	Canary Isl.
ST4-21a	ST4	ST4-21	*Lobaria macaronesica*	11456	-16,2708548	28,53952749	Tenerife	Canary Isl.
ST4-21b	ST4	ST4-21	*Lobaria macaronesica*	11457	-16,2708548	28,53952749	Tenerife	Canary Isl.
ST4-21c	ST4	ST4-21	*Lobaria macaronesica*	11458	-16,2708548	28,53952749	Tenerife	Canary Isl.
ST4-22b	ST4	ST4-22	*Lobaria macaronesica*	11460	-16,27092517	28,53949073	Tenerife	Canary Isl.
ST4-22d	ST4	ST4-22	*Lobaria macaronesica*	11462	-16,27092517	28,53949073	Tenerife	Canary Isl.
ST4-22e	ST4	ST4-22	*Lobaria macaronesica*	11463	-16,27092517	28,53949073	Tenerife	Canary Isl.
ST4-22f	ST4	ST4-22	*Lobaria macaronesica*	11464	-16,27092517	28,53949073	Tenerife	Canary Isl.
ST4-03c	ST4	ST4-3	*Lobaria macaronesica*	11393	-16,27107156	28,53983079	Tenerife	Canary Isl.
ST4-03d	ST4	ST4-3	*Lobaria macaronesica*	11394	-16,27107156	28,53983079	Tenerife	Canary Isl.
ST4-05b	ST4	ST4-5	*Lobaria macaronesica*	11397	-16,27101938	28,53974103	Tenerife	Canary Isl.
ST4-05c	ST4	ST4-5	*Lobaria macaronesica*	11398	-16,27101938	28,53974103	Tenerife	Canary Isl.
ST4-06c	ST4	ST4-6	*Lobaria macaronesica*	11401	-16,27105004	28,53974074	Tenerife	Canary Isl.
ST4-07	ST4	ST4-7	*Lobaria macaronesica*	11402	-16,27096098	28,53969647	Tenerife	Canary Isl.
ST4-08	ST4	ST4-8	*Lobaria macaronesica*	11403	-16,27095761	28,53971429	Tenerife	Canary Isl.
ST4-09e	ST4	ST4-9	*Lobaria macaronesica*	11408	-16,27076633	28,53977778	Tenerife	Canary Isl.
ST4-09f	ST4	ST4-9	*Lobaria macaronesica*	11409	-16,27076633	28,53977778	Tenerife	Canary Isl.
ST4-09g	ST4	ST4-9	*Lobaria macaronesica*	11410	-16,27076633	28,53977778	Tenerife	Canary Isl.

ID	Pop	PopTree	Species	VoucherID	X	Y	Location	Area
ST5-01a	ST5	ST5-1	*Lobaria macaronesica*	11471	-16,17671	28,55810999	Tenerife	Canary Isl.
ST5-01b	ST5	ST5-1	*Lobaria macaronesica*	11472	-16,17671	28,55810999	Tenerife	Canary Isl.
ST5-01c	ST5	ST5-1	*Lobaria macaronesica*	11473	-16,17671	28,55810999	Tenerife	Canary Isl.
ST5-10a	ST5	ST5-10	*Lobaria macaronesica*	11507	-16,17670387	28,55788394	Tenerife	Canary Isl.
ST5-10b	ST5	ST5-10	*Lobaria macaronesica*	11508	-16,17670387	28,55788394	Tenerife	Canary Isl.
ST5-10c	ST5	ST5-10	*Lobaria macaronesica*	11509	-16,17670387	28,55788394	Tenerife	Canary Isl.
ST5-12c	ST5	ST5-12	*Lobaria macaronesica*	11517	-16,17687255	28,55779709	Tenerife	Canary Isl.
ST5-12d	ST5	ST5-12	*Lobaria macaronesica*	11518	-16,17687255	28,55779709	Tenerife	Canary Isl.
ST5-13d	ST5	ST5-13	*Lobaria macaronesica*	11522	-16,17691138	28,55776774	Tenerife	Canary Isl.
ST5-13e	ST5	ST5-13	*Lobaria macaronesica*	11523	-16,17691138	28,55776774	Tenerife	Canary Isl.
ST5-13f	ST5	ST5-13	*Lobaria macaronesica*	11524	-16,17691138	28,55776774	Tenerife	Canary Isl.
ST5-14b	ST5	ST5-14	*Lobaria macaronesica*	11526	-16,17696249	28,5577673	Tenerife	Canary Isl.
ST5-16d	ST5	ST5-16	*Lobaria macaronesica*	11535	-16,17703642	28,55777623	Tenerife	Canary Isl.
ST5-16e	ST5	ST5-16	*Lobaria macaronesica*	11536	-16,17703642	28,55777623	Tenerife	Canary Isl.
ST5-16f	ST5	ST5-16	*Lobaria macaronesica*	11537	-16,17703642	28,55777623	Tenerife	Canary Isl.
ST5-18d	ST5	ST5-18	*Lobaria macaronesica*	11544	-16,17705997	28,55778182	Tenerife	Canary Isl.
ST5-18e	ST5	ST5-18	*Lobaria macaronesica*	11545	-16,17705997	28,55778182	Tenerife	Canary Isl.
ST5-19a	ST5	ST5-19	*Lobaria macaronesica*	11546	-16,17714799	28,55773593	Tenerife	Canary Isl.
ST5-19b	ST5	ST5-19	*Lobaria macaronesica*	11547	-16,17714799	28,55773593	Tenerife	Canary Isl.
ST5-19c	ST5	ST5-19	*Lobaria macaronesica*	11548	-16,17714799	28,55773593	Tenerife	Canary Isl.
ST5-02a	ST5	ST5-2	*Lobaria macaronesica*	11474	-16,17665802	28,55803228	Tenerife	Canary Isl.
ST5-02b	ST5	ST5-2	*Lobaria macaronesica*	11475	-16,17665802	28,55803228	Tenerife	Canary Isl.
ST5-02c	ST5	ST5-2	*Lobaria macaronesica*	11476	-16,17665802	28,55803228	Tenerife	Canary Isl.
ST5-20a	ST5	ST5-20	*Lobaria macaronesica*	11549	-16,17715736	28,55778029	Tenerife	Canary Isl.
ST5-20b	ST5	ST5-20	*Lobaria macaronesica*	11550	-16,17715736	28,55778029	Tenerife	Canary Isl.
ST5-20c	ST5	ST5-20	*Lobaria macaronesica*	11551	-16,17715736	28,55778029	Tenerife	Canary Isl.
ST5-21d	ST5	ST5-21	*Lobaria macaronesica*	11555	-16,17717437	28,55773774	Tenerife	Canary Isl.

ID	Pop	PopTree	Species	VoucherID	X	Y	Location	Area
ST5-21e	ST5	ST5-21	*Lobaria macaronesica*	11556	-16,17717437	28,55773774	Tenerife	Canary Isl.
ST5-21f	ST5	ST5-21	*Lobaria macaronesica*	11557	-16,17717437	28,55773774	Tenerife	Canary Isl.
ST5-22d	ST5	ST5-22	*Lobaria macaronesica*	11561	-16,17725476	28,55772451	Tenerife	Canary Isl.
ST5-22e	ST5	ST5-22	*Lobaria macaronesica*	11562	-16,17725476	28,55772451	Tenerife	Canary Isl.
ST5-23c	ST5	ST5-23	*Lobaria macaronesica*	11566	-16,177288	28,55775879	Tenerife	Canary Isl.
ST5-23d	ST5	ST5-23	*Lobaria macaronesica*	11567	-16,177288	28,55775879	Tenerife	Canary Isl.
ST5-23e	ST5	ST5-23	*Lobaria macaronesica*	11568	-16,177288	28,55775879	Tenerife	Canary Isl.
ST5-23f	ST5	ST5-23	*Lobaria macaronesica*	11569	-16,177288	28,55775879	Tenerife	Canary Isl.
ST5-24a	ST5	ST5-24	*Lobaria macaronesica*	11570	-16,17730476	28,55766976	Tenerife	Canary Isl.
ST5-24b	ST5	ST5-24	*Lobaria macaronesica*	11571	-16,17730476	28,55766976	Tenerife	Canary Isl.
ST5-24c	ST5	ST5-24	*Lobaria macaronesica*	11572	-16,17730476	28,55766976	Tenerife	Canary Isl.
ST5-25d	ST5	ST5-25	*Lobaria macaronesica*	11576	-16,1773239	28,55766342	Tenerife	Canary Isl.
ST5-25e	ST5	ST5-25	*Lobaria macaronesica*	11577	-16,1773239	28,55766342	Tenerife	Canary Isl.
ST5-25f	ST5	ST5-25	*Lobaria macaronesica*	11578	-16,1773239	28,55766342	Tenerife	Canary Isl.
ST5-26d	ST5	ST5-26	*Lobaria macaronesica*	11582	-16,17746746	28,55761587	Tenerife	Canary Isl.
ST5-26e	ST5	ST5-26	*Lobaria macaronesica*	12188	-16,17746746	28,55761587	Tenerife	Canary Isl.
ST5-26f	ST5	ST5-26	*Lobaria macaronesica*	12189	-16,17746746	28,55761587	Tenerife	Canary Isl.
ST5-03a	ST5	ST5-3	*Lobaria macaronesica*	11477	-16,17655753	28,55804883	Tenerife	Canary Isl.
ST5-03b	ST5	ST5-3	*Lobaria macaronesica*	11478	-16,17655753	28,55804883	Tenerife	Canary Isl.
ST5-03c	ST5	ST5-3	*Lobaria macaronesica*	11479	-16,17655753	28,55804883	Tenerife	Canary Isl.
ST5-04a	ST5	ST5-4	*Lobaria macaronesica*	11480	-16,17656592	28,55800432	Tenerife	Canary Isl.
ST5-04b	ST5	ST5-4	*Lobaria macaronesica*	11481	-16,17656592	28,55800432	Tenerife	Canary Isl.
ST5-04c	ST5	ST5-4	*Lobaria macaronesica*	11482	-16,17656592	28,55800432	Tenerife	Canary Isl.
ST5-05a	ST5	ST5-5	*Lobaria macaronesica*	11483	-16,17656759	28,55799541	Tenerife	Canary Isl.
ST5-05b	ST5	ST5-5	*Lobaria macaronesica*	11484	-16,17656759	28,55799541	Tenerife	Canary Isl.
ST5-05c	ST5	ST5-5	*Lobaria macaronesica*	11485	-16,17656759	28,55799541	Tenerife	Canary Isl.
ST5-06a	ST5	ST5-6	*Lobaria macaronesica*	11486	-16,17660083	28,55802969	Tenerife	Canary Isl.

ID	Pop	PopTree	Species	VoucherID	X	Y	Location	Area
ST5-06b	ST5	ST5-6	*Lobaria macaronesica*	11487	-16,17660083	28,55802969	Tenerife	Canary Isl.
ST5-06c	ST5	ST5-6	*Lobaria macaronesica*	11488	-16,17660083	28,55802969	Tenerife	Canary Isl.
ST5-07d	ST5	ST5-7	*Lobaria macaronesica*	11492	-16,17663966	28,55800035	Tenerife	Canary Isl.
ST5-07e	ST5	ST5-7	*Lobaria macaronesica*	11493	-16,17663966	28,55800035	Tenerife	Canary Isl.
ST5-07f	ST5	ST5-7	*Lobaria macaronesica*	11494	-16,17663966	28,55800035	Tenerife	Canary Isl.
ST5-08d	ST5	ST5-8	*Lobaria macaronesica*	11498	-16,17664804	28,55795583	Tenerife	Canary Isl.
ST5-08e	ST5	ST5-8	*Lobaria macaronesica*	11499	-16,17664804	28,55795583	Tenerife	Canary Isl.
ST5-08f	ST5	ST5-8	*Lobaria macaronesica*	11500	-16,17664804	28,55795583	Tenerife	Canary Isl.
ST5-09c	ST5	ST5-9	*Lobaria macaronesica*	11503	-16,17668686	28,55792649	Tenerife	Canary Isl.
ST5-09d	ST5	ST5-9	*Lobaria macaronesica*	11504	-16,17668686	28,55792649	Tenerife	Canary Isl.
ST5-09e	ST5	ST5-9	*Lobaria macaronesica*	11505	-16,17668686	28,55792649	Tenerife	Canary Isl.
ST5-09f	ST5	ST5-9	*Lobaria macaronesica*	11506	-16,17668686	28,55792649	Tenerife	Canary Isl.
ST6-01a	ST6	ST6-1	*Lobaria macaronesica*	11093	-16,22782	28,5433	Tenerife	Canary Isl.
ST6-01b	ST6	ST6-1	*Lobaria macaronesica*	11094	-16,22782	28,5433	Tenerife	Canary Isl.
ST6-01c	ST6	ST6-1	*Lobaria macaronesica*	11095	-16,22782	28,5433	Tenerife	Canary Isl.
ST6-01d	ST6	ST6-1	*Lobaria macaronesica*	11096	-16,22782	28,5433	Tenerife	Canary Isl.
ST6-10a	ST6	ST6-10	*Lobaria macaronesica*	11120	-16,22816879	28,54283656	Tenerife	Canary Isl.
ST6-10b	ST6	ST6-10	*Lobaria macaronesica*	11121	-16,22816879	28,54283656	Tenerife	Canary Isl.
ST6-10c	ST6	ST6-10	*Lobaria macaronesica*	11122	-16,22816879	28,54283656	Tenerife	Canary Isl.
ST6-11c	ST6	ST6-11	*Lobaria macaronesica*	11125	-16,22821278	28,54281359	Tenerife	Canary Isl.
ST6-12a	ST6	ST6-12	*Lobaria macaronesica*	11127	-16,22831498	28,54281267	Tenerife	Canary Isl.
ST6-12b	ST6	ST6-12	*Lobaria macaronesica*	11128	-16,22831498	28,54281267	Tenerife	Canary Isl.
ST6-13a	ST6	ST6-13	*Lobaria macaronesica*	11129	-16,2283049	28,54281119	Tenerife	Canary Isl.
ST6-13b	ST6	ST6-13	*Lobaria macaronesica*	11130	-16,2283049	28,54281119	Tenerife	Canary Isl.
ST6-15	ST6	ST6-15	*Lobaria macaronesica*	11133	-16,22827182	28,54282297	Tenerife	Canary Isl.
ST6-16a	ST6	ST6-16	*Lobaria macaronesica*	11134	-16,22825628	28,54283475	Tenerife	Canary Isl.
ST6-16b	ST6	ST6-16	*Lobaria macaronesica*	11135	-16,22825628	28,54283475	Tenerife	Canary Isl.

ID	Pop	PopTree	Species	VoucherID	X	Y	Location	Area
ST6-16c	ST6	ST6-16	*Lobaria macaronesica*	11136	-16,22825628	28,54283475	Tenerife	Canary Isl.
ST6-17a	ST6	ST6-17	*Lobaria macaronesica*	11137	-16,22824075	28,54284649	Tenerife	Canary Isl.
ST6-17b	ST6	ST6-17	*Lobaria macaronesica*	11138	-16,22824075	28,54284649	Tenerife	Canary Isl.
ST6-17c	ST6	ST6-17	*Lobaria macaronesica*	11139	-16,22824075	28,54284649	Tenerife	Canary Isl.
ST6-17d	ST6	ST6-17	*Lobaria macaronesica*	11140	-16,22824075	28,54284649	Tenerife	Canary Isl.
ST6-18b	ST6	ST6-18	*Lobaria macaronesica*	11142	-16,22823239	28,54289101	Tenerife	Canary Isl.
ST6-18c	ST6	ST6-18	*Lobaria macaronesica*	11143	-16,22823239	28,54289101	Tenerife	Canary Isl.
ST6-19a	ST6	ST6-19	*Lobaria macaronesica*	11144	-16,22813019	28,54289193	Tenerife	Canary Isl.
ST6-19b	ST6	ST6-19	*Lobaria macaronesica*	11145	-16,22813019	28,54289193	Tenerife	Canary Isl.
ST6-19c	ST6	ST6-19	*Lobaria macaronesica*	11146	-16,22813019	28,54289193	Tenerife	Canary Isl.
ST6-19d	ST6	ST6-19	*Lobaria macaronesica*	11147	-16,22813019	28,54289193	Tenerife	Canary Isl.
ST6-02a	ST6	ST6-2	*Lobaria macaronesica*	11097	-16,22785343	28,54312193	Tenerife	Canary Isl.
ST6-02b	ST6	ST6-2	*Lobaria macaronesica*	11098	-16,22785343	28,54312193	Tenerife	Canary Isl.
ST6-02c	ST6	ST6-2	*Lobaria macaronesica*	11099	-16,22785343	28,54312193	Tenerife	Canary Isl.
ST6-02d	ST6	ST6-2	*Lobaria macaronesica*	11100	-16,22785343	28,54312193	Tenerife	Canary Isl.
ST6-20c	ST6	ST6-20	*Lobaria macaronesica*	11150	-16,22813124	28,54298218	Tenerife	Canary Isl.
ST6-20d	ST6	ST6-20	*Lobaria macaronesica*	11151	-16,22813124	28,54298218	Tenerife	Canary Isl.
ST6-20e	ST6	ST6-20	*Lobaria macaronesica*	11152	-16,22813124	28,54298218	Tenerife	Canary Isl.
ST6-21a	ST6	ST6-21	*Lobaria macaronesica*	11153	-16,22812475	28,54298915	Tenerife	Canary Isl.
ST6-21d	ST6	ST6-21	*Lobaria macaronesica*	11156	-16,22812475	28,54298915	Tenerife	Canary Isl.
ST6-21e	ST6	ST6-21	*Lobaria macaronesica*	11157	-16,22812475	28,54298915	Tenerife	Canary Isl.
ST6-21f	ST6	ST6-21	*Lobaria macaronesica*	11158	-16,22812475	28,54298915	Tenerife	Canary Isl.
ST6-23b	ST6	ST6-23	*Lobaria macaronesica*	11163	-16,22807572	28,54301137	Tenerife	Canary Isl.
ST6-23c	ST6	ST6-23	*Lobaria macaronesica*	11164	-16,22807572	28,54301137	Tenerife	Canary Isl.
ST6-24a	ST6	ST6-24	*Lobaria macaronesica*	11165	-16,22827039	28,5428022	Tenerife	Canary Isl.
ST6-03a	ST6	ST6-3	*Lobaria macaronesica*	11101	-16,2281046	28,54308048	Tenerife	Canary Isl.
ST6-03b	ST6	ST6-3	*Lobaria macaronesica*	11102	-16,2281046	28,54308048	Tenerife	Canary Isl.

**ID**	**Pop**	**PopTree**	**Species**	**VoucherID**	**X**	**Y**	**Location**	**Area**
ST6-03c	ST6	ST6-3	*Lobaria macaronesica*	11103	-16,2281046	28,54308048	Tenerife	Canary Isl.
ST6-04a	ST6	ST6-4	*Lobaria macaronesica*	11104	-16,22810449	28,54307146	Tenerife	Canary Isl.
ST6-04b	ST6	ST6-4	*Lobaria macaronesica*	11105	-16,22810449	28,54307146	Tenerife	Canary Isl.
ST6-04c	ST6	ST6-4	*Lobaria macaronesica*	11106	-16,22810449	28,54307146	Tenerife	Canary Isl.
ST6-05b	ST6	ST6-5	*Lobaria macaronesica*	11108	-16,22809427	28,54307155	Tenerife	Canary Isl.
ST6-05c	ST6	ST6-5	*Lobaria macaronesica*	11109	-16,22809427	28,54307155	Tenerife	Canary Isl.
ST6-06a	ST6	ST6-6	*Lobaria macaronesica*	11110	-16,22802938	28,54314127	Tenerife	Canary Isl.
ST6-06b	ST6	ST6-6	*Lobaria macaronesica*	11111	-16,22802938	28,54314127	Tenerife	Canary Isl.
ST6-07a	ST6	ST6-7	*Lobaria macaronesica*	11113	-16,22794035	28,54309695	Tenerife	Canary Isl.
ST6-07b	ST6	ST6-7	*Lobaria macaronesica*	11114	-16,22794035	28,54309695	Tenerife	Canary Isl.
ST6-07c	ST6	ST6-7	*Lobaria macaronesica*	11115	-16,22794035	28,54309695	Tenerife	Canary Isl.
ST6-08	ST6	ST6-8	*Lobaria macaronesica*	11116	-16,22795735	28,54305443	Tenerife	Canary Isl.
ST6-09a	ST6	ST6-9	*Lobaria macaronesica*	11117	-16,22802528	28,54288415	Tenerife	Canary Isl.
ST6-09b	ST6	ST6-9	*Lobaria macaronesica*	11118	-16,22802528	28,54288415	Tenerife	Canary Isl.
TE1-02a	TE1	TE1-2	*Lobaria macaronesica*	13946	-27,20362557	38,75072921	Terceira	Azores
TE1-02b	TE1	TE1-2	*Lobaria macaronesica*	13947	-27,20362557	38,75072921	Terceira	Azores
TE1-02c	TE1	TE1-2	*Lobaria macaronesica*	13948	-27,20362557	38,75072921	Terceira	Azores
TE1-03a	TE1	TE1-3	*Lobaria macaronesica*	13950	-27,20361424	38,75073079	Terceira	Azores
TE1-06c	TE1	TE1-6	*Lobaria macaronesica*	13966	-27,20382203	38,75078216	Terceira	Azores
TE1-06d	TE1	TE1-6	*Lobaria macaronesica*	13967	-27,20382203	38,75078216	Terceira	Azores
TE1-06h	TE1	TE1-6	*Lobaria macaronesica*	13971	-27,20382203	38,75078216	Terceira	Azores
TE2-10c	TE2	TE2-10	*Lobaria macaronesica*	14173	-27,20876224	38,73381437	Terceira	Azores
TE2-11c	TE2	TE2-11	*Lobaria macaronesica*	14177	-27,20876248	38,73381349	Terceira	Azores
TE2-11d	TE2	TE2-11	*Lobaria macaronesica*	14178	-27,20876248	38,73381349	Terceira	Azores
TE2-11e	TE2	TE2-11	*Lobaria macaronesica*	14179	-27,20876248	38,73381349	Terceira	Azores
TE2-12a	TE2	TE2-12	*Lobaria macaronesica*	14183	-27,20871714	38,73380731	Terceira	Azores
TE2-12b	TE2	TE2-12	*Lobaria macaronesica*	14184	-27,20871714	38,73380731	Terceira	Azores

ID	Pop	PopTree	Species	VoucherID	X	Y	Location	Area
TE2-12c	TE2	TE2-12	*Lobaria macaronesica*	14185	-27,20871714	38,73380731	Terceira	Azores
TE2-18b	TE2	TE2-18	*Lobaria macaronesica*	14203	-27,20865356	38,73381011	Terceira	Azores
TE2-20d	TE2	TE2-20	*Lobaria macaronesica*	14212	-27,20865626	38,7338105	Terceira	Azores
TE2-22a	TE2	TE2-22	*Lobaria macaronesica*	14216	-27,20858583	38,73378516	Terceira	Azores
TE2-22b	TE2	TE2-22	*Lobaria macaronesica*	14217	-27,20858583	38,73378516	Terceira	Azores
TE2-22c	TE2	TE2-22	*Lobaria macaronesica*	14218	-27,20858583	38,73378516	Terceira	Azores
TE2-22d	TE2	TE2-22	*Lobaria macaronesica*	14219	-27,20858583	38,73378516	Terceira	Azores
TE2-23a	TE2	TE2-23	*Lobaria macaronesica*	14220	-27,2085661	38,73380735	Terceira	Azores
TE2-23b	TE2	TE2-23	*Lobaria macaronesica*	14221	-27,2085661	38,73380735	Terceira	Azores
TE2-23d	TE2	TE2-23	*Lobaria macaronesica*	14223	-27,2085661	38,73380735	Terceira	Azores
TE2-24a	TE2	TE2-24	*Lobaria macaronesica*	14224	-27,20858741	38,73382861	Terceira	Azores
TE2-24b	TE2	TE2-24	*Lobaria macaronesica*	14225	-27,20858741	38,73382861	Terceira	Azores
TE2-25a	TE2	TE2-25	*Lobaria macaronesica*	14229	-27,20847939	38,73385963	Terceira	Azores
TE2-25c	TE2	TE2-25	*Lobaria macaronesica*	14231	-27,20847939	38,73385963	Terceira	Azores
TE2-25d	TE2	TE2-25	*Lobaria macaronesica*	14232	-27,20847939	38,73385963	Terceira	Azores
TE2-25i	TE2	TE2-25	*Lobaria macaronesica*	14005	-27,20847939	38,73385963	Terceira	Azores
TE2-26a	TE2	TE2-26	*Lobaria macaronesica*	14006	-27,20846173	38,73384807	Terceira	Azores
TE2-26b	TE2	TE2-26	*Lobaria macaronesica*	14007	-27,20846173	38,73384807	Terceira	Azores
TE2-27b	TE2	TE2-27	*Lobaria macaronesica*	14010	-27,20846955	38,73383112	Terceira	Azores
TE2-27c	TE2	TE2-27	*Lobaria macaronesica*	14011	-27,20846955	38,73383112	Terceira	Azores
TE2-27d	TE2	TE2-27	*Lobaria macaronesica*	14012	-27,20846955	38,73383112	Terceira	Azores
TE2-28a	TE2	TE2-28	*Lobaria macaronesica*	14013	-27,20841202	38,73383122	Terceira	Azores
TE2-28b	TE2	TE2-28	*Lobaria macaronesica*	14014	-27,20841202	38,73383122	Terceira	Azores
TE2-29a	TE2	TE2-29	*Lobaria macaronesica*	14016	-27,2084122	38,73389431	Terceira	Azores
TE2-29b	TE2	TE2-29	*Lobaria macaronesica*	14017	-27,2084122	38,73389431	Terceira	Azores
TE2-03b	TE2	TE2-3	*Lobaria macaronesica*	14144	-27,20882946	38,73382014	Terceira	Azores
TE2-31b	TE2	TE2-31	*Lobaria macaronesica*	14020	-27,20797	38,73377	Terceira	Azores

ID	Pop	PopTree	Species	VoucherID	X	Y	Location	Area
TE2-31c	TE2	TE2-31	*Lobaria macaronesica*	14021	-27,20797	38,73377	Terceira	Azores
TE2-31e	TE2	TE2-31	*Lobaria macaronesica*	14023	-27,20797	38,73377	Terceira	Azores
TE2-33a	TE2	TE2-33	*Lobaria macaronesica*	14028	-27,20793858	38,73377472	Terceira	Azores
TE2-05c	TE2	TE2-5	*Lobaria macaronesica*	14150	-27,20869825	38,73378956	Terceira	Azores
TE2-05d	TE2	TE2-5	*Lobaria macaronesica*	14151	-27,20869825	38,73378956	Terceira	Azores
TE2-05e	TE2	TE2-5	*Lobaria macaronesica*	14152	-27,20869825	38,73378956	Terceira	Azores
TE2-06e	TE2	TE2-6	*Lobaria macaronesica*	14158	-27,20873377	38,733825	Terceira	Azores
TE2-07b	TE2	TE2-7	*Lobaria macaronesica*	14160	-27,20872619	38,73382285	Terceira	Azores
TE2-08b	TE2	TE2-8	*Lobaria macaronesica*	14163	-27,20873537	38,73382234	Terceira	Azores
TE2-08c	TE2	TE2-8	*Lobaria macaronesica*	14164	-27,20873537	38,73382234	Terceira	Azores
TE2-08d	TE2	TE2-8	*Lobaria macaronesica*	14165	-27,20873537	38,73382234	Terceira	Azores
TE2-09b	TE2	TE2-9	*Lobaria macaronesica*	14168	-27,20874093	38,73382116	Terceira	Azores
TE2-09c	TE2	TE2-9	*Lobaria macaronesica*	14169	-27,20874093	38,73382116	Terceira	Azores
TE2-09d	TE2	TE2-9	*Lobaria macaronesica*	14170	-27,20874093	38,73382116	Terceira	Azores
TE3-06b	TE3	TE3-6	*Lobaria macaronesica*	14053	-27,27895286	38,71997125	Terceira	Azores
TE4-13e	TE4	TE4-13	*Lobaria macaronesica*	14126	-27,235099	38,69493808	Terceira	Azores
TE4-13f	TE4	TE4-13	*Lobaria macaronesica*	14127	-27,235099	38,69493808	Terceira	Azores
TE4-13g	TE4	TE4-13	*Lobaria macaronesica*	14128	-27,235099	38,69493808	Terceira	Azores
TE4-14a	TE4	TE4-14	*Lobaria macaronesica*	14130	-27,23513585	38,69490348	Terceira	Azores
TE4-14b	TE4	TE4-14	*Lobaria macaronesica*	14131	-27,23513585	38,69490348	Terceira	Azores
TE4-14c	TE4	TE4-14	*Lobaria macaronesica*	14132	-27,23513585	38,69490348	Terceira	Azores
TE4-14d	TE4	TE4-14	*Lobaria macaronesica*	14133	-27,23513585	38,69490348	Terceira	Azores
TE4-14e	TE4	TE4-14	*Lobaria macaronesica*	14134	-27,23513585	38,69490348	Terceira	Azores
TE4-03a	TE4	TE4-3	*Lobaria macaronesica*	14070	-27,23520697	38,69485488	Terceira	Azores
TE4-03c	TE4	TE4-3	*Lobaria macaronesica*	14072	-27,23520697	38,69485488	Terceira	Azores
TE4-03f	TE4	TE4-3	*Lobaria macaronesica*	14075	-27,23520697	38,69485488	Terceira	Azores
TE4-04a	TE4	TE4-4	*Lobaria macaronesica*	14076	-27,23518538	38,69486109	Terceira	Azores

**ID**	**Pop**	**PopTree**	**Species**	**VoucherID**	**X**	**Y**	**Location**	**Area**
TE4-04b	TE4	TE4-4	*Lobaria macaronesica*	14077	-27,23518538	38,69486109	Terceira	Azores
TE4-04c	TE4	TE4-4	*Lobaria macaronesica*	14078	-27,23518538	38,69486109	Terceira	Azores
TE4-04d	TE4	TE4-4	*Lobaria macaronesica*	14079	-27,23518538	38,69486109	Terceira	Azores
TE4-04e	TE4	TE4-4	*Lobaria macaronesica*	14080	-27,23518538	38,69486109	Terceira	Azores
TE4-06a	TE4	TE4-6	*Lobaria macaronesica*	14086	-27,23521386	38,69492823	Terceira	Azores
TE4-06b	TE4	TE4-6	*Lobaria macaronesica*	14087	-27,23521386	38,69492823	Terceira	Azores
TE4-06d	TE4	TE4-6	*Lobaria macaronesica*	14089	-27,23521386	38,69492823	Terceira	Azores
TE4-06f	TE4	TE4-6	*Lobaria macaronesica*	14091	-27,23521386	38,69492823	Terceira	Azores
TE4-07a	TE4	TE4-7	*Lobaria macaronesica*	14092	-27,23515662	38,69500638	Terceira	Azores
EM-10	EM	EM-10	*Lobaria pulmonaria*	5342	-5,5900	36,5200	Spain	Ib. Peninsula
EM-11	EM	EM-11	*Lobaria pulmonaria*	5343	-5,5900	36,5200	Spain	Ib. Peninsula
EM-12	EM	EM-12	*Lobaria pulmonaria*	5344	-5,5900	36,5200	Spain	Ib. Peninsula
EM-13	EM	EM-13	*Lobaria pulmonaria*	5345	-5,5900	36,5200	Spain	Ib. Peninsula
EM-14a	EM	EM-14	*Lobaria pulmonaria*	5346	-5,5900	36,5200	Spain	Ib. Peninsula
EM-14b	EM	EM-14	*Lobaria pulmonaria*	5347	-5,5900	36,5200	Spain	Ib. Peninsula
EM-14c	EM	EM-14	*Lobaria pulmonaria*	5348	-5,5900	36,5200	Spain	Ib. Peninsula
EM-15a	EM	EM-15	*Lobaria pulmonaria*	5349	-5,5900	36,5200	Spain	Ib. Peninsula
EM-15b	EM	EM-15	*Lobaria pulmonaria*	5350	-5,5900	36,5200	Spain	Ib. Peninsula
EM-16	EM	EM-16	*Lobaria pulmonaria*	5351	-5,5900	36,5200	Spain	Ib. Peninsula
EM-17	EM	EM-17	*Lobaria pulmonaria*	5352	-5,5900	36,5200	Spain	Ib. Peninsula
EM-18	EM	EM-18	*Lobaria pulmonaria*	5353	-5,5900	36,5200	Spain	Ib. Peninsula
EM-19	EM	EM-19	*Lobaria pulmonaria*	5354	-5,5900	36,5200	Spain	Ib. Peninsula
EM-02	EM	EM-2	*Lobaria pulmonaria*	5330	-5,5900	36,5200	Spain	Ib. Peninsula
EM-20a	EM	EM-20	*Lobaria pulmonaria*	5355	-5,5900	36,5200	Spain	Ib. Peninsula
EM-20b	EM	EM-20	*Lobaria pulmonaria*	5356	-5,5900	36,5200	Spain	Ib. Peninsula
EM-20c	EM	EM-20	*Lobaria pulmonaria*	5357	-5,5900	36,5200	Spain	Ib. Peninsula
EM-21	EM	EM-21	*Lobaria pulmonaria*	5358	-5,5900	36,5200	Spain	Ib. Peninsula

ID	Pop	PopTree	Species	VoucherID	X	Y	Location	Area
EM-22a	EM	EM-22	*Lobaria pulmonaria*	5359	-5,5900	36,5200	Spain	Ib. Peninsula
EM-22b	EM	EM-22	*Lobaria pulmonaria*	5360	-5,5900	36,5200	Spain	Ib. Peninsula
EM-22c	EM	EM-22	*Lobaria pulmonaria*	5361	-5,5900	36,5200	Spain	Ib. Peninsula
EM-23a	EM	EM-23	*Lobaria pulmonaria*	5362	-5,5900	36,5200	Spain	Ib. Peninsula
EM-23b	EM	EM-23	*Lobaria pulmonaria*	5363	-5,5900	36,5200	Spain	Ib. Peninsula
EM-03	EM	EM-3	*Lobaria pulmonaria*	5331	-5,5900	36,5200	Spain	Ib. Peninsula
EM-04	EM	EM-4	*Lobaria pulmonaria*	5332	-5,5900	36,5200	Spain	Ib. Peninsula
EM-05	EM	EM-5	*Lobaria pulmonaria*	5333	-5,5900	36,5200	Spain	Ib. Peninsula
EM-06a	EM	EM-6	*Lobaria pulmonaria*	5334	-5,5900	36,5200	Spain	Ib. Peninsula
EM-06b	EM	EM-6	*Lobaria pulmonaria*	5335	-5,5900	36,5200	Spain	Ib. Peninsula
EM-07a	EM	EM-7	*Lobaria pulmonaria*	5336	-5,5900	36,5200	Spain	Ib. Peninsula
EM-07b	EM	EM-7	*Lobaria pulmonaria*	5337	-5,5900	36,5200	Spain	Ib. Peninsula
EM-07c	EM	EM-7	*Lobaria pulmonaria*	5338	-5,5900	36,5200	Spain	Ib. Peninsula
EM-08a	EM	EM-8	*Lobaria pulmonaria*	5339	-5,5900	36,5200	Spain	Ib. Peninsula
EM-08b	EM	EM-8	*Lobaria pulmonaria*	5340	-5,5900	36,5200	Spain	Ib. Peninsula
EM-09	EM	EM-9	*Lobaria pulmonaria*	5341	-5,5900	36,5200	Spain	Ib. Peninsula
FA1-20b	FA1	FA1-20	*Lobaria pulmonaria*	14342	-28,77180559	38,58477268	Faial	Azores
FA1-22d	FA1	FA1-22	*Lobaria pulmonaria*	14351	-28,77179502	38,58476935	Faial	Azores
FA1-39a	FA1	FA1-39	*Lobaria pulmonaria*	14409	-28,77119246	38,58444418	Faial	Azores
FA1-06e	FA1	FA1-6	*Lobaria pulmonaria*	14270	-28,77211981	38,58479203	Faial	Azores
FA2-01f	FA2	FA2-1	*Lobaria pulmonaria*	14431	-28,65995	38,56355	Faial	Azores
FA2-21a	FA2	FA2-21	*Lobaria pulmonaria*	14559	-28,65978694	38,56362048	Faial	Azores
FA2-03a	FA2	FA2-3	*Lobaria pulmonaria*	14442	-28,65998386	38,56354965	Faial	Azores
FA2-03b	FA2	FA2-3	*Lobaria pulmonaria*	14443	-28,65998386	38,56354965	Faial	Azores
FA2-03c	FA2	FA2-3	*Lobaria pulmonaria*	14444	-28,65998386	38,56354965	Faial	Azores
FA2-03e	FA2	FA2-3	*Lobaria pulmonaria*	14446	-28,65998386	38,56354965	Faial	Azores
FA2-03g	FA2	FA2-3	*Lobaria pulmonaria*	14448	-28,65998386	38,56354965	Faial	Azores

ID	Pop	PopTree	Species	VoucherID	X	Y	Location	Area
FA2-04a	FA2	FA2-4	*Lobaria pulmonaria*	14449	-28,66000579	38,56355493	Faial	Azores
FA2-04c	FA2	FA2-4	*Lobaria pulmonaria*	14451	-28,66000579	38,56355493	Faial	Azores
FA2-06g	FA2	FA2-6	*Lobaria pulmonaria*	14474	-28,66004375	38,56354844	Faial	Azores
FA2-06j	FA2	FA2-6	*Lobaria pulmonaria*	14477	-28,66004375	38,56354844	Faial	Azores
FA3-14a	FA3	FA3-14	*Lobaria pulmonaria*	14628	-28,69744916	38,61236583	Faial	Azores
FA3-14b	FA3	FA3-14	*Lobaria pulmonaria*	14629	-28,69744916	38,61236583	Faial	Azores
FA3-14c	FA3	FA3-14	*Lobaria pulmonaria*	14630	-28,69744916	38,61236583	Faial	Azores
FA3-14d	FA3	FA3-14	*Lobaria pulmonaria*	14631	-28,69744916	38,61236583	Faial	Azores
FA3-14e	FA3	FA3-14	*Lobaria pulmonaria*	14632	-28,69744916	38,61236583	Faial	Azores
FA3-15f	FA3	FA3-15	*Lobaria pulmonaria*	14638	-28,69754752	38,61231935	Faial	Azores
FA3-23d	FA3	FA3-23	*Lobaria pulmonaria*	14668	-28,68914	38,60943	Faial	Azores
FA3-23e	FA3	FA3-23	*Lobaria pulmonaria*	14669	-28,68914	38,60943	Faial	Azores
FA3-23f	FA3	FA3-23	*Lobaria pulmonaria*	14670	-28,68914	38,60943	Faial	Azores
FA3-04a	FA3	FA3-4	*Lobaria pulmonaria*	14584	-28,70741628	38,61725537	Faial	Azores
FA3-04b	FA3	FA3-4	*Lobaria pulmonaria*	14585	-28,70741628	38,61725537	Faial	Azores
FA3-04c	FA3	FA3-4	*Lobaria pulmonaria*	14586	-28,70741628	38,61725537	Faial	Azores
FA3-04f	FA3	FA3-4	*Lobaria pulmonaria*	14589	-28,70741628	38,61725537	Faial	Azores
FA3-05a	FA3	FA3-5	*Lobaria pulmonaria*	14590	-28,70561	38,6156	Faial	Azores
FA3-05b	FA3	FA3-5	*Lobaria pulmonaria*	14591	-28,70561	38,6156	Faial	Azores
FA3-05c	FA3	FA3-5	*Lobaria pulmonaria*	14592	-28,70561	38,6156	Faial	Azores
FA3-05d	FA3	FA3-5	*Lobaria pulmonaria*	14593	-28,70561	38,6156	Faial	Azores
FA3-05e	FA3	FA3-5	*Lobaria pulmonaria*	14594	-28,70561	38,6156	Faial	Azores
FA3-05f	FA3	FA3-5	*Lobaria pulmonaria*	14595	-28,70561	38,6156	Faial	Azores
FR1-12b	FR1	FR1-12	*Lobaria pulmonaria*	15213	-31,16394429	39,44673594	Flores	Azores
FR1-12c	FR1	FR1-12	*Lobaria pulmonaria*	15214	-31,16394429	39,44673594	Flores	Azores
FR1-16b	FR1	FR1-16	*Lobaria pulmonaria*	15228	-31,16408215	39,44646888	Flores	Azores
FR1-19a	FR1	FR1-19	*Lobaria pulmonaria*	15237	-31,1641172	39,44648722	Flores	Azores

ID	Pop	PopTree	Species	VoucherID	X	Y	Location	Area
FR1-21d	FR1	FR1-21	*Lobaria pulmonaria*	15252	-31,16427369	39,44649011	Flores	Azores
FR1-04h	FR1	FR1-4	*Lobaria pulmonaria*	15182	-31,16422496	39,44668961	Flores	Azores
FR1-04i	FR1	FR1-4	*Lobaria pulmonaria*	15183	-31,16422496	39,44668961	Flores	Azores
FR1-06d	FR1	FR1-6	*Lobaria pulmonaria*	15190	-31,16412267	39,44673033	Flores	Azores
FR2-08b	FR2	FR2-8	*Lobaria pulmonaria*	15284	-31,25140292	39,44739269	Flores	Azores
FR3-20a	FR3	FR3-20	*Lobaria pulmonaria*	15416	-31,15389886	39,44738917	Flores	Azores
FR3-22e	FR3	FR3-22	*Lobaria pulmonaria*	15428	-31,15382212	39,44739832	Flores	Azores
FR3-03a	FR3	FR3-3	*Lobaria pulmonaria*	15340	-31,15415085	39,44726703	Flores	Azores
FR3-04c	FR3	FR3-4	*Lobaria pulmonaria*	15351	-31,15412082	39,44731468	Flores	Azores
FR3-06a	FR3	FR3-6	*Lobaria pulmonaria*	15356	-31,15411627	39,44734097	Flores	Azores
FR3-09a	FR3	FR3-9	*Lobaria pulmonaria*	15368	-31,15407887	39,44735674	Flores	Azores
IRK-01	IRK	IRK-1	*Lobaria pulmonaria*	2455	-9,5300	52,0200	Killarney	Ireland
IRK-10	IRK	IRK-10	*Lobaria pulmonaria*	2464	-9,5300	52,0200	Killarney	Ireland
IRK-11	IRK	IRK-11	*Lobaria pulmonaria*	2465	-9,5300	52,0200	Killarney	Ireland
IRK-12	IRK	IRK-12	*Lobaria pulmonaria*	2466	-9,5300	52,0200	Killarney	Ireland
IRK-13	IRK	IRK-13	*Lobaria pulmonaria*	2467	-9,5300	52,0200	Killarney	Ireland
IRK-14	IRK	IRK-14	*Lobaria pulmonaria*	2468	-9,5300	52,0200	Killarney	Ireland
IRK-15	IRK	IRK-15	*Lobaria pulmonaria*	2469	-9,5300	52,0200	Killarney	Ireland
IRK-16	IRK	IRK-16	*Lobaria pulmonaria*	2470	-9,5300	52,0200	Killarney	Ireland
IRK-17	IRK	IRK-17	*Lobaria pulmonaria*	2471	-9,5300	52,0200	Killarney	Ireland
IRK-18	IRK	IRK-18	*Lobaria pulmonaria*	2472	-9,5300	52,0200	Killarney	Ireland
IRK-19	IRK	IRK-19	*Lobaria pulmonaria*	2473	-9,5300	52,0200	Killarney	Ireland
IRK-02	IRK	IRK-2	*Lobaria pulmonaria*	2456	-9,5300	52,0200	Killarney	Ireland
IRK-20	IRK	IRK-20	*Lobaria pulmonaria*	2474	-9,5300	52,0200	Killarney	Ireland
IRK-21	IRK	IRK-21	*Lobaria pulmonaria*	2475	-9,5300	52,0200	Killarney	Ireland
IRK-22	IRK	IRK-22	*Lobaria pulmonaria*	2476	-9,5300	52,0200	Killarney	Ireland
IRK-24	IRK	IRK-24	*Lobaria pulmonaria*	2478	-9,5300	52,0200	Killarney	Ireland

ID	Pop	PopTree	Species	VoucherID	X	Y	Location	Area
IRK-25	IRK	IRK-25	*Lobaria pulmonaria*	2479	-9,5300	52,0200	Killarney	Ireland
IRK-26	IRK	IRK-26	*Lobaria pulmonaria*	2480	-9,5300	52,0200	Killarney	Ireland
IRK-28	IRK	IRK-28	*Lobaria pulmonaria*	2482	-9,5300	52,0200	Killarney	Ireland
IRK-29	IRK	IRK-29	*Lobaria pulmonaria*	2483	-9,5300	52,0200	Killarney	Ireland
IRK-31	IRK	IRK-31	*Lobaria pulmonaria*	2484	-9,5300	52,0200	Killarney	Ireland
IRK-32	IRK	IRK-32	*Lobaria pulmonaria*	2485	-9,5300	52,0200	Killarney	Ireland
IRK-33	IRK	IRK-33	*Lobaria pulmonaria*	2486	-9,5300	52,0200	Killarney	Ireland
IRK-34	IRK	IRK-34	*Lobaria pulmonaria*	2487	-9,5300	52,0200	Killarney	Ireland
IRK-35	IRK	IRK-35	*Lobaria pulmonaria*	2488	-9,5300	52,0200	Killarney	Ireland
IRK-37	IRK	IRK-37	*Lobaria pulmonaria*	2490	-9,5300	52,0200	Killarney	Ireland
IRK-38	IRK	IRK-38	*Lobaria pulmonaria*	2491	-9,5300	52,0200	Killarney	Ireland
IRK-39	IRK	IRK-39	*Lobaria pulmonaria*	2492	-9,5300	52,0200	Killarney	Ireland
IRK-04	IRK	IRK-4	*Lobaria pulmonaria*	2458	-9,5300	52,0200	Killarney	Ireland
IRK-05	IRK	IRK-5	*Lobaria pulmonaria*	2459	-9,5300	52,0200	Killarney	Ireland
IRK-06	IRK	IRK-6	*Lobaria pulmonaria*	2460	-9,5300	52,0200	Killarney	Ireland
IRK-07	IRK	IRK-7	*Lobaria pulmonaria*	2461	-9,5300	52,0200	Killarney	Ireland
IRK-08	IRK	IRK-8	*Lobaria pulmonaria*	2462	-9,5300	52,0200	Killarney	Ireland
IRK-09	IRK	IRK-9	*Lobaria pulmonaria*	2463	-9,5300	52,0200	Killarney	Ireland
LG-01a	LG	LG-1	*Lobaria pulmonaria*	11299	-15,61932	28,03949	Gran Canaria	Canary Isl.
LG-01b	LG	LG-1	*Lobaria pulmonaria*	11300	-15,61932	28,03949	Gran Canaria	Canary Isl.
LG-02a	LG	LG-2	*Lobaria pulmonaria*	11301	-15,61961822	28,03943235	Gran Canaria	Canary Isl.
LG-02b	LG	LG-2	*Lobaria pulmonaria*	11302	-15,61961822	28,03943235	Gran Canaria	Canary Isl.
LG-02c	LG	LG-2	*Lobaria pulmonaria*	11303	-15,61961822	28,03943235	Gran Canaria	Canary Isl.
LG-03a	LG	LG-3	*Lobaria pulmonaria*	11304	-15,61952072	28,03953651	Gran Canaria	Canary Isl.
LG-03d	LG	LG-3	*Lobaria pulmonaria*	11307	-15,61952072	28,03953651	Gran Canaria	Canary Isl.
LG-04	LG	LG-4	*Lobaria pulmonaria*	11308	-15,6196116	28,0399806	Gran Canaria	Canary Isl.
LG-05	LG	LG-5	*Lobaria pulmonaria*	11309	-15,61968987	28,04003827	Gran Canaria	Canary Isl.

**ID**	**Pop**	**PopTree**	**Species**	**VoucherID**	**X**	**Y**	**Location**	**Area**
LG-06	LG	LG-6	*Lobaria pulmonaria*	11310	-15,6196909	28,0402188	Gran Canaria	Canary Isl.
LG-07a	LG	LG-7	*Lobaria pulmonaria*	11311	-15,61977927	28,04026354	Gran Canaria	Canary Isl.
LG-07c	LG	LG-7	*Lobaria pulmonaria*	11313	-15,61977927	28,04026354	Gran Canaria	Canary Isl.
LG-07d	LG	LG-7	*Lobaria pulmonaria*	11314	-15,61977927	28,04026354	Gran Canaria	Canary Isl.
LG-07e	LG	LG-7	*Lobaria pulmonaria*	11315	-15,61977927	28,04026354	Gran Canaria	Canary Isl.
LG-07f	LG	LG-7	*Lobaria pulmonaria*	11316	-15,61977927	28,04026354	Gran Canaria	Canary Isl.
LG-07g	LG	LG-7	*Lobaria pulmonaria*	11317	-15,61977927	28,04026354	Gran Canaria	Canary Isl.
LG-07h	LG	LG-7	*Lobaria pulmonaria*	11318	-15,61977927	28,04026354	Gran Canaria	Canary Isl.
LG-07j	LG	LG-7	*Lobaria pulmonaria*	11320	-15,61977927	28,04026354	Gran Canaria	Canary Isl.
LG-08a	LG	LG-8	*Lobaria pulmonaria*	11321	-15,61964927	28,04040243	Gran Canaria	Canary Isl.
LG-08b	LG	LG-8	*Lobaria pulmonaria*	11322	-15,61964927	28,04040243	Gran Canaria	Canary Isl.
MA1-05d	MA1	MA1-05	*Lobaria pulmonaria*	25470	36,9675	-25,08493333	Santa Maria	Azores
MA1-06g	MA1	MA1-06	*Lobaria pulmonaria*	25480	36,96749008	-25,08491599	Santa Maria	Azores
MA1-06h	MA1	MA1-06	*Lobaria pulmonaria*	25481	36,96749008	-25,08491599	Santa Maria	Azores
MA1-06i	MA1	MA1-06	*Lobaria pulmonaria*	25482	36,96749008	-25,08491599	Santa Maria	Azores
MA1-06j	MA1	MA1-06	*Lobaria pulmonaria*	25483	36,96749008	-25,08491599	Santa Maria	Azores
MA2-01b	MA2	MA2-01	*Lobaria pulmonaria*	25501	36,9836	-25,0904	Santa Maria	Azores
MA2-01c	MA2	MA2-01	*Lobaria pulmonaria*	25502	36,9836	-25,0904	Santa Maria	Azores
MA2-01i	MA2	MA2-01	*Lobaria pulmonaria*	25508	36,9836	-25,0904	Santa Maria	Azores
MA2-02a	MA2	MA2-02	*Lobaria pulmonaria*	25512	36,98358333	-25,0904	Santa Maria	Azores
MA2-02b	MA2	MA2-02	*Lobaria pulmonaria*	25513	36,98358333	-25,0904	Santa Maria	Azores
MA2-02c	MA2	MA2-02	*Lobaria pulmonaria*	25514	36,98358333	-25,0904	Santa Maria	Azores
MA2-02k	MA2	MA2-02	*Lobaria pulmonaria*	25522	36,98358333	-25,0904	Santa Maria	Azores
MA2-02n	MA2	MA2-02	*Lobaria pulmonaria*	25525	36,98358333	-25,0904	Santa Maria	Azores
MA2-02o	MA2	MA2-02	*Lobaria pulmonaria*	25526	36,98358333	-25,0904	Santa Maria	Azores
MA2-03a	MA2	MA2-03	*Lobaria pulmonaria*	25527	36,98358333	-25,09043333	Santa Maria	Azores
MA2-04a	MA2	MA2-04	*Lobaria pulmonaria*	25537	36,98396667	-25,09011667	Santa Maria	Azores

ID	Pop	PopTree	Species	VoucherID	X	Y	Location	Area
MA2-04b	MA2	MA2-04	*Lobaria pulmonaria*	25538	36,98396667	-25,09011667	Santa Maria	Azores
MA2-10h	MA2	MA2-10	*Lobaria pulmonaria*	25608	-25,09046667	36,98355	Santa Maria	Azores
MA2-04f	MA2	MA2-4	*Lobaria pulmonaria*	25542	-25,09011667	36,98396667	Santa Maria	Azores
MA2-04g	MA2	MA2-4	*Lobaria pulmonaria*	25543	-25,09011667	36,98396667	Santa Maria	Azores
MA2-05d	MA2	MA2-5	*Lobaria pulmonaria*	25550	-25,09028333	36,98371667	Santa Maria	Azores
MA2-05e	MA2	MA2-5	*Lobaria pulmonaria*	25551	-25,09028333	36,98371667	Santa Maria	Azores
MA2-05g	MA2	MA2-5	*Lobaria pulmonaria*	25553	-25,09028333	36,98371667	Santa Maria	Azores
MA2-06d	MA2	MA2-6	*Lobaria pulmonaria*	25561	-25,09038333	36,98356667	Santa Maria	Azores
MA2-08d	MA2	MA2-8	*Lobaria pulmonaria*	25582	-25,09043333	36,98351667	Santa Maria	Azores
PI1-01a	PI1	PI1-1	*Lobaria pulmonaria*	15078	-28,42791	38,46787	Pico	Azores
PI1-01b	PI1	PI1-1	*Lobaria pulmonaria*	15079	-28,42791	38,46787	Pico	Azores
PI1-01c	PI1	PI1-1	*Lobaria pulmonaria*	15080	-28,42791	38,46787	Pico	Azores
PI1-01d	PI1	PI1-1	*Lobaria pulmonaria*	15081	-28,42791	38,46787	Pico	Azores
PI1-12a	PI1	PI1-12	*Lobaria pulmonaria*	15112	-28,42782258	38,46801111	Pico	Azores
PI1-12b	PI1	PI1-12	*Lobaria pulmonaria*	15113	-28,42782258	38,46801111	Pico	Azores
PI1-12c	PI1	PI1-12	*Lobaria pulmonaria*	15114	-28,42782258	38,46801111	Pico	Azores
PI1-12d	PI1	PI1-12	*Lobaria pulmonaria*	15115	-28,42782258	38,46801111	Pico	Azores
PI1-14c	PI1	PI1-14	*Lobaria pulmonaria*	15122	-28,42782205	38,46802396	Pico	Azores
PI1-14d	PI1	PI1-14	*Lobaria pulmonaria*	15123	-28,42782205	38,46802396	Pico	Azores
PI1-15d	PI1	PI1-15	*Lobaria pulmonaria*	15127	-28,42770882	38,46803789	Pico	Azores
PI1-15e	PI1	PI1-15	*Lobaria pulmonaria*	15128	-28,42770882	38,46803789	Pico	Azores
PI1-15g	PI1	PI1-15	*Lobaria pulmonaria*	15130	-28,42770882	38,46803789	Pico	Azores
PI1-02a	PI1	PI1-2	*Lobaria pulmonaria*	15082	-28,42791643	38,46786253	Pico	Azores
PI1-03a	PI1	PI1-3	*Lobaria pulmonaria*	15083	-28,42791643	38,46786253	Pico	Azores
PI1-03b	PI1	PI1-3	*Lobaria pulmonaria*	15084	-28,42791643	38,46786253	Pico	Azores
PI1-04a	PI1	PI1-4	*Lobaria pulmonaria*	15085	-28,42791639	38,46786073	Pico	Azores
PI1-04b	PI1	PI1-4	*Lobaria pulmonaria*	15086	-28,42791639	38,46786073	Pico	Azores

ID	Pop	PopTree	Species	VoucherID	X	Y	Location	Area
PI1-04c	PI1	PI1-4	*Lobaria pulmonaria*	15087	-28,42791639	38,46786073	Pico	Azores
PI1-04d	PI1	PI1-4	*Lobaria pulmonaria*	15088	-28,42791639	38,46786073	Pico	Azores
PI1-04e	PI1	PI1-4	*Lobaria pulmonaria*	15089	-28,42791639	38,46786073	Pico	Azores
PI1-05a	PI1	PI1-5	*Lobaria pulmonaria*	15090	-28,42792362	38,46785374	Pico	Azores
PI1-05b	PI1	PI1-5	*Lobaria pulmonaria*	15091	-28,42792362	38,46785374	Pico	Azores
PI1-05c	PI1	PI1-5	*Lobaria pulmonaria*	15092	-28,42792362	38,46785374	Pico	Azores
PI1-05d	PI1	PI1-5	*Lobaria pulmonaria*	15093	-28,42792362	38,46785374	Pico	Azores
PI1-06a	PI1	PI1-6	*Lobaria pulmonaria*	15094	-28,42789751	38,46789385	Pico	Azores
PI1-06b	PI1	PI1-6	*Lobaria pulmonaria*	15095	-28,42789751	38,46789385	Pico	Azores
PI1-06c	PI1	PI1-6	*Lobaria pulmonaria*	15096	-28,42789751	38,46789385	Pico	Azores
PI1-07a	PI1	PI1-7	*Lobaria pulmonaria*	15097	-28,42788619	38,46789242	Pico	Azores
PI1-07b	PI1	PI1-7	*Lobaria pulmonaria*	15098	-28,42788619	38,46789242	Pico	Azores
PI1-07c	PI1	PI1-7	*Lobaria pulmonaria*	15099	-28,42788619	38,46789242	Pico	Azores
PI1-07d	PI1	PI1-7	*Lobaria pulmonaria*	15100	-28,42788619	38,46789242	Pico	Azores
PI1-08a	PI1	PI1-8	*Lobaria pulmonaria*	15101	-28,42788905	38,46789831	Pico	Azores
PI1-08b	PI1	PI1-8	*Lobaria pulmonaria*	15102	-28,42788905	38,46789831	Pico	Azores
PI1-08c	PI1	PI1-8	*Lobaria pulmonaria*	15103	-28,42788905	38,46789831	Pico	Azores
PI1-09a	PI1	PI1-9	*Lobaria pulmonaria*	15104	-28,42789072	38,46790262	Pico	Azores
PI1-09b	PI1	PI1-9	*Lobaria pulmonaria*	15105	-28,42789072	38,46790262	Pico	Azores
PI1-09c	PI1	PI1-9	*Lobaria pulmonaria*	15106	-28,42789072	38,46790262	Pico	Azores
PI3-13a	PI3	PI3-13	*Lobaria pulmonaria*	14990	-28,257245	38,45601532	Pico	Azores
PI3-13b	PI3	PI3-13	*Lobaria pulmonaria*	14991	-28,257245	38,45601532	Pico	Azores
PI3-13c	PI3	PI3-13	*Lobaria pulmonaria*	14992	-28,257245	38,45601532	Pico	Azores
PI3-15a	PI3	PI3-15	*Lobaria pulmonaria*	15001	-28,25719031	38,45599131	Pico	Azores
PI3-05a	PI3	PI3-5	*Lobaria pulmonaria*	14968	-28,25745	38,45609	Pico	Azores
PI3-05b	PI3	PI3-5	*Lobaria pulmonaria*	14969	-28,25745	38,45609	Pico	Azores
PI3-05c	PI3	PI3-5	*Lobaria pulmonaria*	14970	-28,25745	38,45609	Pico	Azores

**ID**	**Pop**	**PopTree**	**Species**	**VoucherID**	**X**	**Y**	**Location**	**Area**
PI4-01a	PI4	PI4-1	*Lobaria pulmonaria*	15007	-28,253378	38,480439	Pico	Azores
PI4-01b	PI4	PI4-1	*Lobaria pulmonaria*	15008	-28,253378	38,480439	Pico	Azores
PI4-01c	PI4	PI4-1	*Lobaria pulmonaria*	15009	-28,253378	38,480439	Pico	Azores
PI4-10c	PI4	PI4-10	*Lobaria pulmonaria*	15053	-28,25329191	38,48064076	Pico	Azores
PI4-11c	PI4	PI4-11	*Lobaria pulmonaria*	15057	-28,25328106	38,48064366	Pico	Azores
PI4-11d	PI4	PI4-11	*Lobaria pulmonaria*	15058	-28,25328106	38,48064366	Pico	Azores
PI4-11f	PI4	PI4-11	*Lobaria pulmonaria*	15060	-28,25328106	38,48064366	Pico	Azores
PI4-12d	PI4	PI4-12	*Lobaria pulmonaria*	15064	-28,25327498	38,48064778	Pico	Azores
PI4-14a	PI4	PI4-14	*Lobaria pulmonaria*	15073	-28,25327599	38,48065657	Pico	Azores
PI4-14b	PI4	PI4-14	*Lobaria pulmonaria*	15074	-28,25327599	38,48065657	Pico	Azores
PI4-02d	PI4	PI4-2	*Lobaria pulmonaria*	15013	-28,25331576	38,480565	Pico	Azores
PI4-02f	PI4	PI4-2	*Lobaria pulmonaria*	15015	-28,25331576	38,480565	Pico	Azores
PI4-04a	PI4	PI4-4	*Lobaria pulmonaria*	15023	-28,25327417	38,48060895	Pico	Azores
PI4-04b	PI4	PI4-4	*Lobaria pulmonaria*	15024	-28,25327417	38,48060895	Pico	Azores
PI4-05c	PI4	PI4-5	*Lobaria pulmonaria*	15029	-28,25326828	38,48061323	Pico	Azores
PI4-07c	PI4	PI4-7	*Lobaria pulmonaria*	15040	-28,25327271	38,48062269	Pico	Azores
PI4-08a	PI4	PI4-8	*Lobaria pulmonaria*	15041	-28,25327196	38,48062439	Pico	Azores
PI4-08b	PI4	PI4-8	*Lobaria pulmonaria*	15042	-28,25327196	38,48062439	Pico	Azores
PI4-08d	PI4	PI4-8	*Lobaria pulmonaria*	15044	-28,25327196	38,48062439	Pico	Azores
PI4-09b	PI4	PI4-9	*Lobaria pulmonaria*	15047	-28,2532887	38,48062749	Pico	Azores
PI4-09e	PI4	PI4-9	*Lobaria pulmonaria*	15050	-28,2532887	38,48062749	Pico	Azores
PM11-01e	PM11	PM11-1	*Lobaria pulmonaria*	12436	-16,882969	32,7384	Madeira	Madeira
PM11-01f	PM11	PM11-1	*Lobaria pulmonaria*	12437	-16,882969	32,7384	Madeira	Madeira
PM11-11a	PM11	PM11-11	*Lobaria pulmonaria*	12459	-16,88176343	32,73883014	Madeira	Madeira
PM11-11b	PM11	PM11-11	*Lobaria pulmonaria*	12460	-16,88176343	32,73883014	Madeira	Madeira
PM11-11c	PM11	PM11-11	*Lobaria pulmonaria*	12461	-16,88176343	32,73883014	Madeira	Madeira
PM11-11g	PM11	PM11-11	*Lobaria pulmonaria*	12465	-16,88176343	32,73883014	Madeira	Madeira

ID	Pop	PopTree	Species	VoucherID	X	Y	Location	Area
PM11-12a	PM11	PM11-12	*Lobaria pulmonaria*	12466	-16,88163739	32,73870768	Madeira	Madeira
PM11-12b	PM11	PM11-12	*Lobaria pulmonaria*	12467	-16,88163739	32,73870768	Madeira	Madeira
PM11-13d	PM11	PM11-13	*Lobaria pulmonaria*	12474	-16,88146234	32,73853759	Madeira	Madeira
PM11-13e	PM11	PM11-13	*Lobaria pulmonaria*	12475	-16,88146234	32,73853759	Madeira	Madeira
PM11-14a	PM11	PM11-14	*Lobaria pulmonaria*	12476	-16,88142032	32,73849677	Madeira	Madeira
PM11-14b	PM11	PM11-14	*Lobaria pulmonaria*	12477	-16,88142032	32,73849677	Madeira	Madeira
PM11-02d	PM11	PM11-2	*Lobaria pulmonaria*	12488	-16,88288522	32,73841378	Madeira	Madeira
PM11-03	PM11	PM11-3	*Lobaria pulmonaria*	12441	-16,88288844	32,73856705	Madeira	Madeira
PM11-05d	PM11	PM11-5	*Lobaria pulmonaria*	12487	-16,88268926	32,73878208	Madeira	Madeira
PM11-07a	PM11	PM11-7	*Lobaria pulmonaria*	12448	-16,88262887	32,7388511	Madeira	Madeira
PM11-07b	PM11	PM11-7	*Lobaria pulmonaria*	12449	-16,88262887	32,7388511	Madeira	Madeira
PM11-09c	PM11	PM11-9	*Lobaria pulmonaria*	12456	-16,88246632	32,73871984	Madeira	Madeira
PM12-01a	PM12	PM12-1	*Lobaria pulmonaria*	12490	-17,01588	32,7615	Madeira	Madeira
PM12-10a	PM12	PM12-10	*Lobaria pulmonaria*	12532	-17,01617491	32,76162247	Madeira	Madeira
PM12-07e	PM12	PM12-7	*Lobaria pulmonaria*	12567	-17,01619248	32,76159742	Madeira	Madeira
PM12-07f	PM12	PM12-7	*Lobaria pulmonaria*	12568	-17,01619248	32,76159742	Madeira	Madeira
PM12-07g	PM12	PM12-7	*Lobaria pulmonaria*	12569	-17,01619248	32,76159742	Madeira	Madeira
PM12-09a	PM12	PM12-9	*Lobaria pulmonaria*	12529	-17,01620654	32,76162666	Madeira	Madeira
PM13-10d	PM13	PM13-10	*Lobaria pulmonaria*	12632	-17,13152381	32,76343777	Madeira	Madeira
PM13-11a	PM13	PM13-11	*Lobaria pulmonaria*	12633	-17,13152084	32,76342911	Madeira	Madeira
PM13-11b	PM13	PM13-11	*Lobaria pulmonaria*	12634	-17,13152084	32,76342911	Madeira	Madeira
PM13-13a	PM13	PM13-13	*Lobaria pulmonaria*	12648	-17,13152783	32,76341122	Madeira	Madeira
PM13-13b	PM13	PM13-13	*Lobaria pulmonaria*	12649	-17,13152783	32,76341122	Madeira	Madeira
PM13-14a	PM13	PM13-14	*Lobaria pulmonaria*	12655	-17,13147612	32,76353922	Madeira	Madeira
PM13-16d	PM13	PM13-16	*Lobaria pulmonaria*	12667	-17,13158949	32,76363512	Madeira	Madeira
PM13-17a	PM13	PM13-17	*Lobaria pulmonaria*	12672	-17,13166646	32,76369758	Madeira	Madeira
PM13-02c	PM13	PM13-2	*Lobaria pulmonaria*	12578	-17,13163653	32,76336511	Madeira	Madeira

ID	Pop	PopTree	Species	VoucherID	X	Y	Location	Area
PM13-02d	PM13	PM13-2	*Lobaria pulmonaria*	12579	-17,13163653	32,76336511	Madeira	Madeira
PM13-02e	PM13	PM13-2	*Lobaria pulmonaria*	12580	-17,13163653	32,76336511	Madeira	Madeira
PM13-21a	PM13	PM13-21	*Lobaria pulmonaria*	12695	-17,13170221	32,76371635	Madeira	Madeira
PM13-21b	PM13	PM13-21	*Lobaria pulmonaria*	12696	-17,13170221	32,76371635	Madeira	Madeira
PM13-23a	PM13	PM13-23	*Lobaria pulmonaria*	12709	-17,1317268	32,76366934	Madeira	Madeira
PM13-03a	PM13	PM13-3	*Lobaria pulmonaria*	12583	-17,13158484	32,76339702	Madeira	Madeira
PM13-04a	PM13	PM13-4	*Lobaria pulmonaria*	12590	-17,13159743	32,76341159	Madeira	Madeira
PM13-04b	PM13	PM13-4	*Lobaria pulmonaria*	12591	-17,13159743	32,76341159	Madeira	Madeira
PM13-05b	PM13	PM13-5	*Lobaria pulmonaria*	12596	-17,13160257	32,76340369	Madeira	Madeira
PM13-08a	PM13	PM13-8	*Lobaria pulmonaria*	12615	-17,13159083	32,76342763	Madeira	Madeira
PM13-08c	PM13	PM13-8	*Lobaria pulmonaria*	12617	-17,13159083	32,76342763	Madeira	Madeira
PM13-08d	PM13	PM13-8	*Lobaria pulmonaria*	12618	-17,13159083	32,76342763	Madeira	Madeira
PM13-09a	PM13	PM13-9	*Lobaria pulmonaria*	12619	-17,13152848	32,76343994	Madeira	Madeira
PM13-09b	PM13	PM13-9	*Lobaria pulmonaria*	12620	-17,13152848	32,76343994	Madeira	Madeira
PM13-09c	PM13	PM13-9	*Lobaria pulmonaria*	12621	-17,13152848	32,76343994	Madeira	Madeira
PM13-09d	PM13	PM13-9	*Lobaria pulmonaria*	12622	-17,13152848	32,76343994	Madeira	Madeira
PM13-09e	PM13	PM13-9	*Lobaria pulmonaria*	12623	-17,13152848	32,76343994	Madeira	Madeira
PM13-09f	PM13	PM13-9	*Lobaria pulmonaria*	12624	-17,13152848	32,76343994	Madeira	Madeira
PM14-01b	PM14	PM14-1	*Lobaria pulmonaria*	12738	-17,18930	32,83067	Madeira	Madeira
PM14-10a	PM14	PM14-10	*Lobaria pulmonaria*	12793	-17,18926558	32,83075518	Madeira	Madeira
PM14-13a	PM14	PM14-13	*Lobaria pulmonaria*	12818	-17,18929137	32,83090879	Madeira	Madeira
PM14-16a	PM14	PM14-16	*Lobaria pulmonaria*	12833	-17,18922965	32,83099378	Madeira	Madeira
PM14-16b	PM14	PM14-16	*Lobaria pulmonaria*	12834	-17,18922965	32,83099378	Madeira	Madeira
PM14-17b	PM14	PM14-17	*Lobaria pulmonaria*	12840	-17,18921723	32,83101872	Madeira	Madeira
PM14-17c	PM14	PM14-17	*Lobaria pulmonaria*	12841	-17,18921723	32,83101872	Madeira	Madeira
PM14-17d	PM14	PM14-17	*Lobaria pulmonaria*	12842	-17,18921723	32,83101872	Madeira	Madeira
PM14-20a	PM14	PM14-20	*Lobaria pulmonaria*	12855	-17,18917886	32,83101658	Madeira	Madeira

ID	Pop	PopTree	Species	VoucherID	X	Y	Location	Area
PM15-01d	PM15	PM15-1	*Lobaria pulmonaria*	12866	-17,157942	32,826744	Madeira	Madeira
PM15-01e	PM15	PM15-1	*Lobaria pulmonaria*	12867	-17,157942	32,826744	Madeira	Madeira
PM15-02b	PM15	PM15-2	*Lobaria pulmonaria*	12870	-17,15788252	32,82657081	Madeira	Madeira
PM15-23b	PM15	PM15-23	*Lobaria pulmonaria*	12966	-17,15849292	32,82526377	Madeira	Madeira
PM16-01c	PM16	PM16-1	*Lobaria pulmonaria*	13036	-17,14085	32,80675	Madeira	Madeira
PM16-10d	PM16	PM16-10	*Lobaria pulmonaria*	13080	-17,14062971	32,80713525	Madeira	Madeira
PM16-10e	PM16	PM16-10	*Lobaria pulmonaria*	13081	-17,14062971	32,80713525	Madeira	Madeira
PM16-10f	PM16	PM16-10	*Lobaria pulmonaria*	13082	-17,14062971	32,80713525	Madeira	Madeira
PM16-13e	PM16	PM16-13	*Lobaria pulmonaria*	13096	-17,14058053	32,80713653	Madeira	Madeira
PM16-04c	PM16	PM16-4	*Lobaria pulmonaria*	13047	-17,14079231	32,80695999	Madeira	Madeira
PM16-04d	PM16	PM16-4	*Lobaria pulmonaria*	13048	-17,14079231	32,80695999	Madeira	Madeira
PM16-04e	PM16	PM16-4	*Lobaria pulmonaria*	13049	-17,14079231	32,80695999	Madeira	Madeira
PM16-04f	PM16	PM16-4	*Lobaria pulmonaria*	13050	-17,14079231	32,80695999	Madeira	Madeira
PM16-05c	PM16	PM16-5	*Lobaria pulmonaria*	13055	-17,14080872	32,80690771	Madeira	Madeira
PM16-07f	PM16	PM16-7	*Lobaria pulmonaria*	13066	-17,14062748	32,80701111	Madeira	Madeira
PM17-13b	PM17	PM17-13	*Lobaria pulmonaria*	13172	-16,83223653	32,74487991	Madeira	Madeira
PM17-17a	PM17	PM17-17	*Lobaria pulmonaria*	13183	-16,83228391	32,744938	Madeira	Madeira
PM17-17b	PM17	PM17-17	*Lobaria pulmonaria*	13184	-16,83228391	32,744938	Madeira	Madeira
PM17-17c	PM17	PM17-17	*Lobaria pulmonaria*	13185	-16,83228391	32,744938	Madeira	Madeira
PM17-20a	PM17	PM17-20	*Lobaria pulmonaria*	13189	-16,83303808	32,74497826	Madeira	Madeira
PM17-22a	PM17	PM17-22	*Lobaria pulmonaria*	13196	-16,83389492	32,74551424	Madeira	Madeira
PM17-06b	PM17	PM17-6	*Lobaria pulmonaria*	13152	-16,8318848	32,7445219	Madeira	Madeira
PM9-16b	PM9	PM9-16	*Lobaria pulmonaria*	12366	-16,88561008	32,73798555	Madeira	Madeira
PM9-02f	PM9	PM9-2	*Lobaria pulmonaria*	12303	-16,88470334	32,7379992	Madeira	Madeira
PM9-02g	PM9	PM9-2	*Lobaria pulmonaria*	12304	-16,88470334	32,7379992	Madeira	Madeira
PM9-05a	PM9	PM9-5	*Lobaria pulmonaria*	12319	-16,88481535	32,73799751	Madeira	Madeira
PM9-05b	PM9	PM9-5	*Lobaria pulmonaria*	12320	-16,88481535	32,73799751	Madeira	Madeira

ID	Pop	PopTree	Species	VoucherID	X	Y	Location	Area
PO2-01a	PO2	PO2-1	*Lobaria pulmonaria*	3345	-8,8800	39,4900	Arrimal	Ib. Peninsula
PO2-10	PO2	PO2-10	*Lobaria pulmonaria*	3368	-8,8800	39,4900	Arrimal	Ib. Peninsula
PO2-11a	PO2	PO2-11	*Lobaria pulmonaria*	3369	-8,8800	39,4900	Arrimal	Ib. Peninsula
PO2-12a	PO2	PO2-12	*Lobaria pulmonaria*	3375	-8,8800	39,4900	Arrimal	Ib. Peninsula
PO2-13	PO2	PO2-13	*Lobaria pulmonaria*	3378	-8,8800	39,4900	Arrimal	Ib. Peninsula
PO2-15a	PO2	PO2-15	*Lobaria pulmonaria*	3382	-8,8800	39,4900	Arrimal	Ib. Peninsula
PO2-16a	PO2	PO2-16	*Lobaria pulmonaria*	3385	-8,8800	39,4900	Arrimal	Ib. Peninsula
PO2-18	PO2	PO2-18	*Lobaria pulmonaria*	3388	-8,8800	39,4900	Arrimal	Ib. Peninsula
PO2-19	PO2	PO2-19	*Lobaria pulmonaria*	3389	-8,8800	39,4900	Arrimal	Ib. Peninsula
PO2-02	PO2	PO2-2	*Lobaria pulmonaria*	3351	-8,8800	39,4900	Arrimal	Ib. Peninsula
PO2-20a	PO2	PO2-20	*Lobaria pulmonaria*	3390	-8,8800	39,4900	Arrimal	Ib. Peninsula
PO2-22a	PO2	PO2-22	*Lobaria pulmonaria*	3393	-8,8800	39,4900	Arrimal	Ib. Peninsula
PO2-23a	PO2	PO2-23	*Lobaria pulmonaria*	3395	-8,8800	39,4900	Arrimal	Ib. Peninsula
PO2-23b	PO2	PO2-23	*Lobaria pulmonaria*	3396	-8,8800	39,4900	Arrimal	Ib. Peninsula
PO2-24	PO2	PO2-24	*Lobaria pulmonaria*	3398	-8,8800	39,4900	Arrimal	Ib. Peninsula
PO2-25	PO2	PO2-25	*Lobaria pulmonaria*	3399	-8,8800	39,4900	Arrimal	Ib. Peninsula
PO2-26	PO2	PO2-26	*Lobaria pulmonaria*	3400	-8,8800	39,4900	Arrimal	Ib. Peninsula
PO2-27	PO2	PO2-27	*Lobaria pulmonaria*	3401	-8,8800	39,4900	Arrimal	Ib. Peninsula
PO2-28	PO2	PO2-28	*Lobaria pulmonaria*	3402	-8,8800	39,4900	Arrimal	Ib. Peninsula
PO2-03	PO2	PO2-3	*Lobaria pulmonaria*	3352	-8,8800	39,4900	Arrimal	Ib. Peninsula
PO2-30	PO2	PO2-30	*Lobaria pulmonaria*	3404	-8,8800	39,4900	Arrimal	Ib. Peninsula
PO2-31	PO2	PO2-31	*Lobaria pulmonaria*	3405	-8,8800	39,4900	Arrimal	Ib. Peninsula
PO2-32	PO2	PO2-32	*Lobaria pulmonaria*	3406	-8,8800	39,4900	Arrimal	Ib. Peninsula
PO2-06a	PO2	PO2-6	*Lobaria pulmonaria*	3361	-8,8800	39,4900	Arrimal	Ib. Peninsula
PO2-06c	PO2	PO2-6	*Lobaria pulmonaria*	3363	-8,8800	39,4900	Arrimal	Ib. Peninsula
PO2-07a	PO2	PO2-7	*Lobaria pulmonaria*	3364	-8,8800	39,4900	Arrimal	Ib. Peninsula
PO2-08	PO2	PO2-8	*Lobaria pulmonaria*	3366	-8,8800	39,4900	Arrimal	Ib. Peninsula

ID	Pop	PopTree	Species	VoucherID	X	Y	Location	Area
PO2-09	PO2	PO2-9	*Lobaria pulmonaria*	3367	-8,8800	39,4900	Arrimal	Ib. Peninsula
PS-01a	PS	PS-1	*Lobaria pulmonaria*	12193	-9,388582	38,789359	Sintra	Ib. Peninsula
PS-01b	PS	PS-1	*Lobaria pulmonaria*	12194	-9,388582	38,789359	Sintra	Ib. Peninsula
PS-10	PS	PS-10	*Lobaria pulmonaria*	12211	-9,388741423	38,7901998	Sintra	Ib. Peninsula
PS-11	PS	PS-11	*Lobaria pulmonaria*	12212	-9,388841381	38,79024453	Sintra	Ib. Peninsula
PS-12	PS	PS-12	*Lobaria pulmonaria*	12213	-9,388881318	38,79026242	Sintra	Ib. Peninsula
PS-13	PS	PS-13	*Lobaria pulmonaria*	12214	-9,3886653	38,79032478	Sintra	Ib. Peninsula
PS-14a	PS	PS-14	*Lobaria pulmonaria*	12215	-9,389571713	38,79019659	Sintra	Ib. Peninsula
PS-14b	PS	PS-14	*Lobaria pulmonaria*	12216	-9,389571713	38,79019659	Sintra	Ib. Peninsula
PS-14c	PS	PS-14	*Lobaria pulmonaria*	12217	-9,389571713	38,79019659	Sintra	Ib. Peninsula
PS-15a	PS	PS-15	*Lobaria pulmonaria*	12225	-9,389	38,790	Sintra	Ib. Peninsula
PS-15b	PS	PS-15	*Lobaria pulmonaria*	12226	-9,389	38,790	Sintra	Ib. Peninsula
PS-15c	PS	PS-15	*Lobaria pulmonaria*	12227	-9,389	38,790	Sintra	Ib. Peninsula
PS-15d	PS	PS-15	*Lobaria pulmonaria*	12228	-9,389	38,790	Sintra	Ib. Peninsula
PS-15e	PS	PS-15	*Lobaria pulmonaria*	12229	-9,389	38,790	Sintra	Ib. Peninsula
PS-15f	PS	PS-15	*Lobaria pulmonaria*	12230	-9,389	38,790	Sintra	Ib. Peninsula
PS-15g	PS	PS-15	*Lobaria pulmonaria*	12231	-9,389	38,790	Sintra	Ib. Peninsula
PS-15h	PS	PS-15	*Lobaria pulmonaria*	12232	-9,389	38,790	Sintra	Ib. Peninsula
PS-15i	PS	PS-15	*Lobaria pulmonaria*	12233	-9,389	38,790	Sintra	Ib. Peninsula
PS-15j	PS	PS-15	*Lobaria pulmonaria*	12234	-9,389	38,790	Sintra	Ib. Peninsula
PS-15k	PS	PS-15	*Lobaria pulmonaria*	12235	-9,389	38,790	Sintra	Ib. Peninsula
PS-15l	PS	PS-15	*Lobaria pulmonaria*	12236	-9,389	38,790	Sintra	Ib. Peninsula
PS-16a	PS	PS-16	*Lobaria pulmonaria*	12237	-9,389	38,790	Sintra	Ib. Peninsula
PS-16b	PS	PS-16	*Lobaria pulmonaria*	12238	-9,389	38,790	Sintra	Ib. Peninsula
PS-18b	PS	PS-18	*Lobaria pulmonaria*	12240	-9,389	38,792	Sintra	Ib. Peninsula
PS-18c	PS	PS-18	*Lobaria pulmonaria*	12241	-9,389	38,792	Sintra	Ib. Peninsula
PS-02	PS	PS-2	*Lobaria pulmonaria*	12195	-9,388582	38,789359	Sintra	Ib. Peninsula

ID	Pop	PopTree	Species	VoucherID	X	Y	Location	Area
PS-20a	PS	PS-20	*Lobaria pulmonaria*	12245	-9,389	38,792	Sintra	Ib. Peninsula
PS-20b	PS	PS-20	*Lobaria pulmonaria*	12246	-9,389	38,792	Sintra	Ib. Peninsula
PS-22a	PS	PS-22	*Lobaria pulmonaria*	12247	-9,389	38,792	Sintra	Ib. Peninsula
PS-22b	PS	PS-22	*Lobaria pulmonaria*	12248	-9,389	38,792	Sintra	Ib. Peninsula
PS-23a	PS	PS-23	*Lobaria pulmonaria*	12251	-9,389	38,792	Sintra	Ib. Peninsula
PS-24a	PS	PS-24	*Lobaria pulmonaria*	12253	-9,389	38,792	Sintra	Ib. Peninsula
PS-24b	PS	PS-24	*Lobaria pulmonaria*	12254	-9,389	38,792	Sintra	Ib. Peninsula
PS-24c	PS	PS-24	*Lobaria pulmonaria*	12255	-9,389	38,792	Sintra	Ib. Peninsula
PS-26a	PS	PS-26	*Lobaria pulmonaria*	12258	-9,389	38,792	Sintra	Ib. Peninsula
PS-26b	PS	PS-26	*Lobaria pulmonaria*	12259	-9,389	38,792	Sintra	Ib. Peninsula
PS-29a	PS	PS-29	*Lobaria pulmonaria*	12260	-9,388434	38,792938	Sintra	Ib. Peninsula
PS-29b	PS	PS-29	*Lobaria pulmonaria*	12261	-9,388434	38,792938	Sintra	Ib. Peninsula
PS-29c	PS	PS-29	*Lobaria pulmonaria*	12262	-9,388434	38,792938	Sintra	Ib. Peninsula
PS-03a	PS	PS-3	*Lobaria pulmonaria*	12196	-9,388471555	38,78946292	Sintra	Ib. Peninsula
PS-03b	PS	PS-3	*Lobaria pulmonaria*	12197	-9,388471555	38,78946292	Sintra	Ib. Peninsula
PS-03c	PS	PS-3	*Lobaria pulmonaria*	12198	-9,388471555	38,78946292	Sintra	Ib. Peninsula
PS-03d	PS	PS-3	*Lobaria pulmonaria*	12199	-9,388471555	38,78946292	Sintra	Ib. Peninsula
PS-30a	PS	PS-30	*Lobaria pulmonaria*	12263	-9,388436439	38,79338859	Sintra	Ib. Peninsula
PS-30b	PS	PS-30	*Lobaria pulmonaria*	12264	-9,388436439	38,79338859	Sintra	Ib. Peninsula
PS-30c	PS	PS-30	*Lobaria pulmonaria*	12265	-9,388436439	38,79338859	Sintra	Ib. Peninsula
PS-31a	PS	PS-31	*Lobaria pulmonaria*	12266	-9,388480697	38,79341737	Sintra	Ib. Peninsula
PS-31b	PS	PS-31	*Lobaria pulmonaria*	12267	-9,388480697	38,79341737	Sintra	Ib. Peninsula
PS-31c	PS	PS-31	*Lobaria pulmonaria*	12268	-9,388480697	38,79341737	Sintra	Ib. Peninsula
PS-31d	PS	PS-31	*Lobaria pulmonaria*	12269	-9,388480697	38,79341737	Sintra	Ib. Peninsula
PS-32a	PS	PS-32	*Lobaria pulmonaria*	12270	-9,388483	38,79341736	Sintra	Ib. Peninsula
PS-32b	PS	PS-32	*Lobaria pulmonaria*	12271	-9,388483	38,79341736	Sintra	Ib. Peninsula
PS-32c	PS	PS-32	*Lobaria pulmonaria*	12272	-9,388483	38,79341736	Sintra	Ib. Peninsula

ID	Pop	PopTree	Species	VoucherID	X	Y	Location	Area
PS-33a	PS	PS-33	*Lobaria pulmonaria*	12273	-9,388501397	38,79349724	Sintra	Ib. Peninsula
PS-33b	PS	PS-33	*Lobaria pulmonaria*	12274	-9,388501397	38,79349724	Sintra	Ib. Peninsula
PS-33c	PS	PS-33	*Lobaria pulmonaria*	12275	-9,388501397	38,79349724	Sintra	Ib. Peninsula
PS-34a	PS	PS-34	*Lobaria pulmonaria*	12276	-9,389279912	38,791801	Sintra	Ib. Peninsula
PS-34c	PS	PS-34	*Lobaria pulmonaria*	12278	-9,389279912	38,791801	Sintra	Ib. Peninsula
PS-34d	PS	PS-34	*Lobaria pulmonaria*	12279	-9,389279912	38,791801	Sintra	Ib. Peninsula
PS-34f	PS	PS-34	*Lobaria pulmonaria*	12281	-9,389279912	38,791801	Sintra	Ib. Peninsula
PS-35a	PS	PS-35	*Lobaria pulmonaria*	12282	-9,392734263	38,79178944	Sintra	Ib. Peninsula
PS-35b	PS	PS-35	*Lobaria pulmonaria*	12283	-9,392734263	38,79178944	Sintra	Ib. Peninsula
PS-35c	PS	PS-35	*Lobaria pulmonaria*	12284	-9,392734263	38,79178944	Sintra	Ib. Peninsula
PS-36a	PS	PS-36	*Lobaria pulmonaria*	12285	-9,392804214	38,79182075	Sintra	Ib. Peninsula
PS-36b	PS	PS-36	*Lobaria pulmonaria*	12286	-9,392804214	38,79182075	Sintra	Ib. Peninsula
PS-37a	PS	PS-37	*Lobaria pulmonaria*	12289	-9,393	38,792	Sintra	Ib. Peninsula
PS-37b	PS	PS-37	*Lobaria pulmonaria*	12290	-9,393	38,792	Sintra	Ib. Peninsula
PS-04a	PS	PS-4	*Lobaria pulmonaria*	12200	-9,388869939	38,79030841	Sintra	Ib. Peninsula
PS-04b	PS	PS-4	*Lobaria pulmonaria*	12201	-9,388869939	38,79030841	Sintra	Ib. Peninsula
PS-05a	PS	PS-5	*Lobaria pulmonaria*	12202	-9,388824221	38,79037102	Sintra	Ib. Peninsula
PS-05b	PS	PS-5	*Lobaria pulmonaria*	12203	-9,388824221	38,79037102	Sintra	Ib. Peninsula
PS-05c	PS	PS-5	*Lobaria pulmonaria*	12204	-9,388824221	38,79037102	Sintra	Ib. Peninsula
PS-06	PS	PS-6	*Lobaria pulmonaria*	12205	-9,388647193	38,79025572	Sintra	Ib. Peninsula
PS-07	PS	PS-7	*Lobaria pulmonaria*	12206	-9,388602936	38,79022694	Sintra	Ib. Peninsula
PS-08a	PS	PS-8	*Lobaria pulmonaria*	12207	-9,388558679	38,79019807	Sintra	Ib. Peninsula
PS-08b	PS	PS-8	*Lobaria pulmonaria*	12208	-9,388558679	38,79019807	Sintra	Ib. Peninsula
PS-08c	PS	PS-8	*Lobaria pulmonaria*	12209	-9,388558679	38,79019807	Sintra	Ib. Peninsula
PS-09a	PS	PS-9	*Lobaria pulmonaria*	12221	-9,388514423	38,79016929	Sintra	Ib. Peninsula
PS-09b	PS	PS-9	*Lobaria pulmonaria*	12222	-9,388514423	38,79016929	Sintra	Ib. Peninsula
PS-09c	PS	PS-9	*Lobaria pulmonaria*	12223	-9,388514423	38,79016929	Sintra	Ib. Peninsula

ID	Pop	PopTree	Species	VoucherID	X	Y	Location	Area
PS2-01a	PS	PS2-1	*Lobaria pulmonaria*	31731	-9,395743	38,794302	Sintra	Ib. Peninsula
PS2-01b	PS	PS2-1	*Lobaria pulmonaria*	31732	-9,395743	38,794302	Sintra	Ib. Peninsula
PS2-01c	PS	PS2-1	*Lobaria pulmonaria*	31733	-9,395743	38,794302	Sintra	Ib. Peninsula
PS2-01d	PS	PS2-2	*Lobaria pulmonaria*	31734	-9,395743	38,794302	Sintra	Ib. Peninsula
PS2-02b	PS	PS2-2	*Lobaria pulmonaria*	31736	-9,39024	38,791834	Sintra	Ib. Peninsula
PS2-02c	PS	PS2-2	*Lobaria pulmonaria*	31737	-9,39024	38,791834	Sintra	Ib. Peninsula
PS2-02d	PS	PS2-2	*Lobaria pulmonaria*	31738	-9,39024	38,791834	Sintra	Ib. Peninsula
PS2-02e	PS	PS2-2	*Lobaria pulmonaria*	31739	-9,39024	38,791834	Sintra	Ib. Peninsula
PS2-02g	PS	PS2-2	*Lobaria pulmonaria*	31741	-9,39024	38,791834	Sintra	Ib. Peninsula
PS2-03a	PS	PS2-3	*Lobaria pulmonaria*	31742	-9,395743	38,794302	Sintra	Ib. Peninsula
PS2-03b	PS	PS2-3	*Lobaria pulmonaria*	31743	-9,395743	38,794302	Sintra	Ib. Peninsula
SG1-01a	SG1	SG1-1	*Lobaria pulmonaria*	11936	-17,29628	28,15027	La Gomera	Canary Isl.
SG1-01b	SG1	SG1-1	*Lobaria pulmonaria*	11937	-17,29628	28,15027	La Gomera	Canary Isl.
SG1-01c	SG1	SG1-1	*Lobaria pulmonaria*	11938	-17,29628	28,15027	La Gomera	Canary Isl.
SG1-01c1	SG1	SG1-1	*Lobaria pulmonaria*	11938	-17,29628	28,15027	La Gomera	Canary Isl.
SG1-01d	SG1	SG1-1	*Lobaria pulmonaria*	11939	-17,29628	28,15027	La Gomera	Canary Isl.
SG1-16a	SG1	SG1-16	*Lobaria pulmonaria*	11991	-17,29631433	28,15063614	La Gomera	Canary Isl.
SG1-19	SG1	SG1-19	*Lobaria pulmonaria*	12004	-17,2960208	28,1505887	La Gomera	Canary Isl.
SG1-02a	SG1	SG1-2	*Lobaria pulmonaria*	11940	-17,29628349	28,15023405	La Gomera	Canary Isl.
SG1-05	SG1	SG1-5	*Lobaria pulmonaria*	11952	-17,2962111	28,15030157	La Gomera	Canary Isl.
SG1-06b	SG1	SG1-6	*Lobaria pulmonaria*	11954	-17,2962616	28,15029601	La Gomera	Canary Isl.
SG1-09b	SG1	SG1-9	*Lobaria pulmonaria*	11961	-17,29620233	28,15043883	La Gomera	Canary Isl.
SG1-09c	SG1	SG1-9	*Lobaria pulmonaria*	11962	-17,29620233	28,15043883	La Gomera	Canary Isl.
SG3-13d	SG3	SG3-13	*Lobaria pulmonaria*	10496	-17,25645306	28,1318256	La Gomera	Canary Isl.
SG3-14d	SG3	SG3-14	*Lobaria pulmonaria*	10500	-17,25646975	28,13183593	La Gomera	Canary Isl.
SG3-15b	SG3	SG3-15	*Lobaria pulmonaria*	10504	-17,25652323	28,13189035	La Gomera	Canary Isl.
SG3-15c	SG3	SG3-15	*Lobaria pulmonaria*	10505	-17,25652323	28,13189035	La Gomera	Canary Isl.

ID	Pop	PopTree	Species	VoucherID	X	Y	Location	Area
SG3-16a	SG3	SG3-16	*Lobaria pulmonaria*	10506	-17,25653157	28,13189552	La Gomera	Canary Isl.
SG3-16b	SG3	SG3-16	*Lobaria pulmonaria*	10507	-17,25653157	28,13189552	La Gomera	Canary Isl.
SG3-17b	SG3	SG3-17	*Lobaria pulmonaria*	10512	-17,25658408	28,13197279	La Gomera	Canary Isl.
SG3-18	SG3	SG3-18	*Lobaria pulmonaria*	10513	-17,25659731	28,13201635	La Gomera	Canary Isl.
SG3-19b	SG3	SG3-19	*Lobaria pulmonaria*	10515	-17,25662357	28,13205498	La Gomera	Canary Isl.
SG3-19d	SG3	SG3-19	*Lobaria pulmonaria*	10517	-17,25662357	28,13205498	La Gomera	Canary Isl.
SG3-19e	SG3	SG3-19	*Lobaria pulmonaria*	10518	-17,25662357	28,13205498	La Gomera	Canary Isl.
SG3-20a	SG3	SG3-20	*Lobaria pulmonaria*	10519	-17,25664197	28,13207658	La Gomera	Canary Isl.
SG3-22a	SG3	SG3-22	*Lobaria pulmonaria*	10526	-17,25693125	28,13193436	La Gomera	Canary Isl.
SG3-22b	SG3	SG3-22	*Lobaria pulmonaria*	10527	-17,25693125	28,13193436	La Gomera	Canary Isl.
SG3-22c	SG3	SG3-22	*Lobaria pulmonaria*	10528	-17,25693125	28,13193436	La Gomera	Canary Isl.
SG3-22d	SG3	SG3-22	*Lobaria pulmonaria*	10529	-17,25693125	28,13193436	La Gomera	Canary Isl.
SG3-23b	SG3	SG3-23	*Lobaria pulmonaria*	10531	-17,25696468	28,13196837	La Gomera	Canary Isl.
SG3-24a	SG3	SG3-24	*Lobaria pulmonaria*	10535	-17,25697418	28,1319716	La Gomera	Canary Isl.
SG3-24b	SG3	SG3-24	*Lobaria pulmonaria*	10536	-17,25697418	28,1319716	La Gomera	Canary Isl.
SG3-24c	SG3	SG3-24	*Lobaria pulmonaria*	10537	-17,25697418	28,1319716	La Gomera	Canary Isl.
SG3-25a	SG3	SG3-25	*Lobaria pulmonaria*	10538	-17,257025	28,13197391	La Gomera	Canary Isl.
SG3-25b	SG3	SG3-25	*Lobaria pulmonaria*	10539	-17,257025	28,13197391	La Gomera	Canary Isl.
SG3-25c	SG3	SG3-25	*Lobaria pulmonaria*	10540	-17,257025	28,13197391	La Gomera	Canary Isl.
SG3-26f	SG3	SG3-26	*Lobaria pulmonaria*	10546	-17,25706458	28,13200226	La Gomera	Canary Isl.
SG3-06a	SG3	SG3-6	*Lobaria pulmonaria*	10477	-17,25631763	28,13110747	La Gomera	Canary Isl.
SG3-08c	SG3	SG3-8	*Lobaria pulmonaria*	10484	-17,25648852	28,13129254	La Gomera	Canary Isl.
SG3-09	SG3	SG3-9	*Lobaria pulmonaria*	10486	-17,25650189	28,13130614	La Gomera	Canary Isl.
SG4-14b	SG4	SG4-14	*Lobaria pulmonaria*	12058	-17,21549731	28,12156987	La Gomera	Canary Isl.
SG4-14c	SG4	SG4-14	*Lobaria pulmonaria*	12059	-17,21549731	28,12156987	La Gomera	Canary Isl.
SG4-15e	SG4	SG4-15	*Lobaria pulmonaria*	12066	-17,21549443	28,121569	La Gomera	Canary Isl.
SG4-15f	SG4	SG4-15	*Lobaria pulmonaria*	12067	-17,21549443	28,121569	La Gomera	Canary Isl.

ID	Pop	PopTree	Species	VoucherID	X	Y	Location	Area
SG4-15g	SG4	SG4-15	*Lobaria pulmonaria*	12068	-17,21549443	28,121569	La Gomera	Canary Isl.
SG4-17	SG4	SG4-17	*Lobaria pulmonaria*	12070	-17,21552396	28,12150209	La Gomera	Canary Isl.
SG4-19a	SG4	SG4-19	*Lobaria pulmonaria*	12073	-17,21557737	28,12140612	La Gomera	Canary Isl.
SG4-19d	SG4	SG4-19	*Lobaria pulmonaria*	12076	-17,21557737	28,12140612	La Gomera	Canary Isl.
SG4-20c	SG4	SG4-20	*Lobaria pulmonaria*	12079	-17,21557405	28,12139759	La Gomera	Canary Isl.
SG4-23f	SG4	SG4-23	*Lobaria pulmonaria*	12091	-17,21575453	28,12097994	La Gomera	Canary Isl.
SG4-25a	SG4	SG4-25	*Lobaria pulmonaria*	12095	-17,21618424	28,1198707	La Gomera	Canary Isl.
SG4-25b	SG4	SG4-25	*Lobaria pulmonaria*	12096	-17,21618424	28,1198707	La Gomera	Canary Isl.
SG4-03c	SG4	SG4-3	*Lobaria pulmonaria*	12024	-17,21515148	28,12209415	La Gomera	Canary Isl.
SG4-06d	SG4	SG4-6	*Lobaria pulmonaria*	12036	-17,21522258	28,1219964	La Gomera	Canary Isl.
SG4-09b	SG4	SG4-9	*Lobaria pulmonaria*	12046	-17,21530355	28,12186325	La Gomera	Canary Isl.
SG5-12b	SG5	SG5-12	*Lobaria pulmonaria*	10599	-17,25655227	28,13067212	La Gomera	Canary Isl.
SG5-13d	SG5	SG5-13	*Lobaria pulmonaria*	10603	-17,25658529	28,13058679	La Gomera	Canary Isl.
SG5-14c	SG5	SG5-14	*Lobaria pulmonaria*	10606	-17,25662187	28,13067098	La Gomera	Canary Isl.
SG5-18d	SG5	SG5-18	*Lobaria pulmonaria*	10630	-17,25652	28,1305661	La Gomera	Canary Isl.
SG5-18e	SG5	SG5-18	*Lobaria pulmonaria*	10631	-17,25652	28,1305661	La Gomera	Canary Isl.
SG5-02a	SG5	SG5-2	*Lobaria pulmonaria*	10560	-17,25632723	28,13097886	La Gomera	Canary Isl.
SG5-02f	SG5	SG5-2	*Lobaria pulmonaria*	10565	-17,25632723	28,13097886	La Gomera	Canary Isl.
SG5-24e	SG5	SG5-24	*Lobaria pulmonaria*	10656	-17,25629027	28,13037514	La Gomera	Canary Isl.
SG5-24g	SG5	SG5-24	*Lobaria pulmonaria*	10658	-17,25629027	28,13037514	La Gomera	Canary Isl.
SG5-06a	SG5	SG5-6	*Lobaria pulmonaria*	10574	-17,25635232	28,13094137	La Gomera	Canary Isl.
SG5-07a	SG5	SG5-7	*Lobaria pulmonaria*	10576	-17,25640156	28,13086242	La Gomera	Canary Isl.
SG5-07c	SG5	SG5-7	*Lobaria pulmonaria*	10578	-17,25640156	28,13086242	La Gomera	Canary Isl.
SG5-08c	SG5	SG5-8	*Lobaria pulmonaria*	10581	-17,25642618	28,13082294	La Gomera	Canary Isl.
SH1-10b	SH1	SH1-10	*Lobaria pulmonaria*	10691	-17,98020643	27,76137287	El Hierro	Canary Isl.
SH1-11d	SH1	SH1-11	*Lobaria pulmonaria*	10695	-17,98015481	27,76145048	El Hierro	Canary Isl.
SH1-11e	SH1	SH1-11	*Lobaria pulmonaria*	10696	-17,98015481	27,76145048	El Hierro	Canary Isl.

**ID**	**Pop**	**PopTree**	**Species**	**VoucherID**	**X**	**Y**	**Location**	**Area**
SH1-12a	SH1	SH1-12	*Lobaria pulmonaria*	10697	-17,98015383	27,76141442	El Hierro	Canary Isl.
SH1-12b	SH1	SH1-12	*Lobaria pulmonaria*	10698	-17,98015383	27,76141442	El Hierro	Canary Isl.
SH1-12c	SH1	SH1-12	*Lobaria pulmonaria*	10699	-17,98015383	27,76141442	El Hierro	Canary Isl.
SH1-13d	SH1	SH1-13	*Lobaria pulmonaria*	10703	-17,98009082	27,76144346	El Hierro	Canary Isl.
SH1-14d	SH1	SH1-14	*Lobaria pulmonaria*	10707	-17,98004861	27,7614185	El Hierro	Canary Isl.
SH1-14f	SH1	SH1-14	*Lobaria pulmonaria*	10709	-17,98004861	27,7614185	El Hierro	Canary Isl.
SH1-14g	SH1	SH1-14	*Lobaria pulmonaria*	10710	-17,98004861	27,7614185	El Hierro	Canary Isl.
SH1-18d	SH1	SH1-18	*Lobaria pulmonaria*	10725	-17,97945066	27,7612833	El Hierro	Canary Isl.
SH1-02a	SH1	SH1-2	*Lobaria pulmonaria*	10660	-17,98084152	27,76108956	El Hierro	Canary Isl.
SH1-02b	SH1	SH1-2	*Lobaria pulmonaria*	10661	-17,98084152	27,76108956	El Hierro	Canary Isl.
SH1-02c	SH1	SH1-2	*Lobaria pulmonaria*	10662	-17,98084152	27,76108956	El Hierro	Canary Isl.
SH1-02h	SH1	SH1-2	*Lobaria pulmonaria*	10667	-17,98084152	27,76108956	El Hierro	Canary Isl.
SH1-20a	SH1	SH1-20	*Lobaria pulmonaria*	10733	-17,97952449	27,76115538	El Hierro	Canary Isl.
SH1-20b	SH1	SH1-20	*Lobaria pulmonaria*	10734	-17,97952449	27,76115538	El Hierro	Canary Isl.
SH1-20c	SH1	SH1-20	*Lobaria pulmonaria*	10735	-17,97952449	27,76115538	El Hierro	Canary Isl.
SH1-03d	SH1	SH1-3	*Lobaria pulmonaria*	11294	-17,98080092	27,76117223	El Hierro	Canary Isl.
SH1-03e	SH1	SH1-3	*Lobaria pulmonaria*	11295	-17,98080092	27,76117223	El Hierro	Canary Isl.
SH1-08a	SH1	SH1-8	*Lobaria pulmonaria*	10681	-17,98047179	27,76130239	El Hierro	Canary Isl.
SH1-08b	SH1	SH1-8	*Lobaria pulmonaria*	10682	-17,98047179	27,76130239	El Hierro	Canary Isl.
SH1-08e	SH1	SH1-8	*Lobaria pulmonaria*	10685	-17,98047179	27,76130239	El Hierro	Canary Isl.
SH1-09d	SH1	SH1-9	*Lobaria pulmonaria*	10689	-17,98037572	27,76127361	El Hierro	Canary Isl.
SH2-01a	SH2	SH2-1	*Lobaria pulmonaria*	10746	-17,98651999	27,74317	El Hierro	Canary Isl.
SH2-01b	SH2	SH2-1	*Lobaria pulmonaria*	10747	-17,98651999	27,74317	El Hierro	Canary Isl.
SH2-01c	SH2	SH2-1	*Lobaria pulmonaria*	10748	-17,98651999	27,74317	El Hierro	Canary Isl.
SH2-10b	SH2	SH2-10	*Lobaria pulmonaria*	10775	-17,98673516	27,74280424	El Hierro	Canary Isl.
SH2-10c	SH2	SH2-10	*Lobaria pulmonaria*	10776	-17,98673516	27,74280424	El Hierro	Canary Isl.
SH2-11a	SH2	SH2-11	*Lobaria pulmonaria*	10777	-17,98673962	27,74274125	El Hierro	Canary Isl.

ID	Pop	PopTree	Species	VoucherID	X	Y	Location	Area
SH2-11b	SH2	SH2-11	*Lobaria pulmonaria*	10778	-17,98673962	27,74274125	El Hierro	Canary Isl.
SH2-11c	SH2	SH2-11	*Lobaria pulmonaria*	10779	-17,98673962	27,74274125	El Hierro	Canary Isl.
SH2-12a	SH2	SH2-12	*Lobaria pulmonaria*	10780	-17,98679153	27,74271306	El Hierro	Canary Isl.
SH2-12c	SH2	SH2-12	*Lobaria pulmonaria*	10782	-17,98679153	27,74271306	El Hierro	Canary Isl.
SH2-13b	SH2	SH2-13	*Lobaria pulmonaria*	10785	-17,98694353	27,74270978	El Hierro	Canary Isl.
SH2-13c	SH2	SH2-13	*Lobaria pulmonaria*	10786	-17,98694353	27,74270978	El Hierro	Canary Isl.
SH2-14a	SH2	SH2-14	*Lobaria pulmonaria*	10787	-17,98695092	27,74272657	El Hierro	Canary Isl.
SH2-14e	SH2	SH2-14	*Lobaria pulmonaria*	10791	-17,98695092	27,74272657	El Hierro	Canary Isl.
SH2-15a	SH2	SH2-15	*Lobaria pulmonaria*	10792	-17,98694121	27,7427424	El Hierro	Canary Isl.
SH2-15b	SH2	SH2-15	*Lobaria pulmonaria*	10793	-17,98694121	27,7427424	El Hierro	Canary Isl.
SH2-15c	SH2	SH2-15	*Lobaria pulmonaria*	10794	-17,98694121	27,7427424	El Hierro	Canary Isl.
SH2-16a	SH2	SH2-16	*Lobaria pulmonaria*	10795	-17,98684819	27,74277821	El Hierro	Canary Isl.
SH2-17a	SH2	SH2-17	*Lobaria pulmonaria*	10798	-17,98692665	27,74279641	El Hierro	Canary Isl.
SH2-17b	SH2	SH2-17	*Lobaria pulmonaria*	10799	-17,98692665	27,74279641	El Hierro	Canary Isl.
SH2-17c	SH2	SH2-17	*Lobaria pulmonaria*	10800	-17,98692665	27,74279641	El Hierro	Canary Isl.
SH2-18a	SH2	SH2-18	*Lobaria pulmonaria*	10801	-17,98684595	27,74253557	El Hierro	Canary Isl.
SH2-18b	SH2	SH2-18	*Lobaria pulmonaria*	10802	-17,98684595	27,74253557	El Hierro	Canary Isl.
SH2-18c	SH2	SH2-18	*Lobaria pulmonaria*	10803	-17,98684595	27,74253557	El Hierro	Canary Isl.
SH2-19a	SH2	SH2-19	*Lobaria pulmonaria*	10805	-17,98690084	27,74248247	El Hierro	Canary Isl.
SH2-19b	SH2	SH2-19	*Lobaria pulmonaria*	10806	-17,98690084	27,74248247	El Hierro	Canary Isl.
SH2-19c	SH2	SH2-19	*Lobaria pulmonaria*	10807	-17,98690084	27,74248247	El Hierro	Canary Isl.
SH2-02a	SH2	SH2-2	*Lobaria pulmonaria*	10749	-17,98652453	27,74317202	El Hierro	Canary Isl.
SH2-02b	SH2	SH2-2	*Lobaria pulmonaria*	10750	-17,98652453	27,74317202	El Hierro	Canary Isl.
SH2-02c	SH2	SH2-2	*Lobaria pulmonaria*	10751	-17,98652453	27,74317202	El Hierro	Canary Isl.
SH2-20a	SH2	SH2-20	*Lobaria pulmonaria*	10809	-17,98693118	27,74230414	El Hierro	Canary Isl.
SH2-20b	SH2	SH2-20	*Lobaria pulmonaria*	10810	-17,98693118	27,74230414	El Hierro	Canary Isl.
SH2-20c	SH2	SH2-20	*Lobaria pulmonaria*	10811	-17,98693118	27,74230414	El Hierro	Canary Isl.

ID	Pop	PopTree	Species	VoucherID	X	Y	Location	Area
SH2-03a	SH2	SH2-3	*Lobaria pulmonaria*	10752	-17,9865244	27,74316751	El Hierro	Canary Isl.
SH2-03b	SH2	SH2-3	*Lobaria pulmonaria*	10753	-17,9865244	27,74316751	El Hierro	Canary Isl.
SH2-03c	SH2	SH2-3	*Lobaria pulmonaria*	10754	-17,9865244	27,74316751	El Hierro	Canary Isl.
SH2-04a	SH2	SH2-4	*Lobaria pulmonaria*	10755	-17,98661927	27,74306181	El Hierro	Canary Isl.
SH2-04b	SH2	SH2-4	*Lobaria pulmonaria*	10756	-17,98661927	27,74306181	El Hierro	Canary Isl.
SH2-04c	SH2	SH2-4	*Lobaria pulmonaria*	10757	-17,98661927	27,74306181	El Hierro	Canary Isl.
SH2-05a	SH2	SH2-5	*Lobaria pulmonaria*	10758	-17,98663445	27,74297264	El Hierro	Canary Isl.
SH2-05b	SH2	SH2-5	*Lobaria pulmonaria*	10759	-17,98663445	27,74297264	El Hierro	Canary Isl.
SH2-06a	SH2	SH2-6	*Lobaria pulmonaria*	10761	-17,98664024	27,74295536	El Hierro	Canary Isl.
SH2-06b	SH2	SH2-6	*Lobaria pulmonaria*	10762	-17,98664024	27,74295536	El Hierro	Canary Isl.
SH2-07a	SH2	SH2-7	*Lobaria pulmonaria*	10764	-17,98661067	27,74288819	El Hierro	Canary Isl.
SH2-07b	SH2	SH2-7	*Lobaria pulmonaria*	10765	-17,98661067	27,74288819	El Hierro	Canary Isl.
SH2-07c	SH2	SH2-7	*Lobaria pulmonaria*	10766	-17,98661067	27,74288819	El Hierro	Canary Isl.
SH2-08b	SH2	SH2-8	*Lobaria pulmonaria*	10769	-17,98672392	27,74284871	El Hierro	Canary Isl.
SH2-08c	SH2	SH2-8	*Lobaria pulmonaria*	10770	-17,98672392	27,74284871	El Hierro	Canary Isl.
SH2-09b	SH2	SH2-9	*Lobaria pulmonaria*	10772	-17,98670281	27,74288971	El Hierro	Canary Isl.
SH3-10a	SH3	SH3-10	*Lobaria pulmonaria*	11185	-18,01245973	27,73128006	El Hierro	Canary Isl.
SH3-10b	SH3	SH3-10	*Lobaria pulmonaria*	11186	-18,01245973	27,73128006	El Hierro	Canary Isl.
SH3-10c	SH3	SH3-10	*Lobaria pulmonaria*	11187	-18,01245973	27,73128006	El Hierro	Canary Isl.
SH3-10d	SH3	SH3-10	*Lobaria pulmonaria*	11188	-18,01245973	27,73128006	El Hierro	Canary Isl.
SH3-10e	SH3	SH3-10	*Lobaria pulmonaria*	11189	-18,01245973	27,73128006	El Hierro	Canary Isl.
SH3-10f	SH3	SH3-10	*Lobaria pulmonaria*	11190	-18,01245973	27,73128006	El Hierro	Canary Isl.
SH3-12b	SH3	SH3-12	*Lobaria pulmonaria*	11198	-18,01245157	27,73128584	El Hierro	Canary Isl.
SH3-13	SH3	SH3-13	*Lobaria pulmonaria*	11200	-18,01264362	27,73122828	El Hierro	Canary Isl.
SH3-14a	SH3	SH3-14	*Lobaria pulmonaria*	11201	-18,01277759	27,73109289	El Hierro	Canary Isl.
SH3-14b	SH3	SH3-14	*Lobaria pulmonaria*	11202	-18,01277759	27,73109289	El Hierro	Canary Isl.
SH3-14c	SH3	SH3-14	*Lobaria pulmonaria*	11203	-18,01277759	27,73109289	El Hierro	Canary Isl.

ID	Pop	PopTree	Species	VoucherID	X	Y	Location	Area
SH3-16a	SH3	SH3-16	*Lobaria pulmonaria*	11204	-18,01279446	27,73105041	El Hierro	Canary Isl.
SH3-16b	SH3	SH3-16	*Lobaria pulmonaria*	11205	-18,01279446	27,73105041	El Hierro	Canary Isl.
SH3-16c	SH3	SH3-16	*Lobaria pulmonaria*	11206	-18,01279446	27,73105041	El Hierro	Canary Isl.
SH3-17a	SH3	SH3-17	*Lobaria pulmonaria*	11207	-18,01279142	27,73104683	El Hierro	Canary Isl.
SH3-17b	SH3	SH3-17	*Lobaria pulmonaria*	11208	-18,01279142	27,73104683	El Hierro	Canary Isl.
SH3-17c	SH3	SH3-17	*Lobaria pulmonaria*	11209	-18,01279142	27,73104683	El Hierro	Canary Isl.
SH3-18a	SH3	SH3-18	*Lobaria pulmonaria*	11210	-18,01279391	27,73104841	El Hierro	Canary Isl.
SH3-18b	SH3	SH3-18	*Lobaria pulmonaria*	11211	-18,01279391	27,73104841	El Hierro	Canary Isl.
SH3-19	SH3	SH3-19	*Lobaria pulmonaria*	11212	-18,0127976	27,73104299	El Hierro	Canary Isl.
SH3-02b	SH3	SH3-2	*Lobaria pulmonaria*	11170	-18,01163668	27,73286091	El Hierro	Canary Isl.
SH3-20a	SH3	SH3-20	*Lobaria pulmonaria*	11213	-18,01287332	27,73082786	El Hierro	Canary Isl.
SH3-20b	SH3	SH3-20	*Lobaria pulmonaria*	11214	-18,01287332	27,73082786	El Hierro	Canary Isl.
SH3-21	SH3	SH3-21	*Lobaria pulmonaria*	11215	-18,01288849	27,7307848	El Hierro	Canary Isl.
SH3-22	SH3	SH3-22	*Lobaria pulmonaria*	11216	-18,01291638	27,73074715	El Hierro	Canary Isl.
SH3-23	SH3	SH3-23	*Lobaria pulmonaria*	11217	-18,01298086	27,73072085	El Hierro	Canary Isl.
SH3-24	SH3	SH3-24	*Lobaria pulmonaria*	11218	-18,01300064	27,73071695	El Hierro	Canary Isl.
SH3-25a	SH3	SH3-25	*Lobaria pulmonaria*	11219	-18,01311074	27,73062362	El Hierro	Canary Isl.
SH3-25b	SH3	SH3-25	*Lobaria pulmonaria*	11220	-18,01311074	27,73062362	El Hierro	Canary Isl.
SH3-25c	SH3	SH3-25	*Lobaria pulmonaria*	11221	-18,01311074	27,73062362	El Hierro	Canary Isl.
SH3-26a	SH3	SH3-26	*Lobaria pulmonaria*	11222	-18,01304316	27,73074484	El Hierro	Canary Isl.
SH3-26b	SH3	SH3-26	*Lobaria pulmonaria*	11223	-18,01304316	27,73074484	El Hierro	Canary Isl.
SH3-26c	SH3	SH3-26	*Lobaria pulmonaria*	11224	-18,01304316	27,73074484	El Hierro	Canary Isl.
SH3-26d	SH3	SH3-26	*Lobaria pulmonaria*	11225	-18,01304316	27,73074484	El Hierro	Canary Isl.
SH3-28a	SH3	SH3-28	*Lobaria pulmonaria*	11232	-18,01297216	27,73076838	El Hierro	Canary Isl.
SH3-28b	SH3	SH3-28	*Lobaria pulmonaria*	11233	-18,01297216	27,73076838	El Hierro	Canary Isl.
SH3-28c	SH3	SH3-28	*Lobaria pulmonaria*	11234	-18,01297216	27,73076838	El Hierro	Canary Isl.
SH3-28g	SH3	SH3-28	*Lobaria pulmonaria*	11238	-18,01297216	27,73076838	El Hierro	Canary Isl.

ID	Pop	PopTree	Species	VoucherID	X	Y	Location	Area
SH3-28h	SH3	SH3-28	*Lobaria pulmonaria*	11239	-18,01297216	27,73076838	El Hierro	Canary Isl.
SH3-29a	SH3	SH3-29	*Lobaria pulmonaria*	11240	-18,01292079	27,73081193	El Hierro	Canary Isl.
SH3-29b	SH3	SH3-29	*Lobaria pulmonaria*	11241	-18,01292079	27,73081193	El Hierro	Canary Isl.
SH3-29c	SH3	SH3-29	*Lobaria pulmonaria*	11242	-18,01292079	27,73081193	El Hierro	Canary Isl.
SH3-29d	SH3	SH3-29	*Lobaria pulmonaria*	11243	-18,01292079	27,73081193	El Hierro	Canary Isl.
SH3-29e	SH3	SH3-29	*Lobaria pulmonaria*	11244	-18,01292079	27,73081193	El Hierro	Canary Isl.
SH3-29f	SH3	SH3-29	*Lobaria pulmonaria*	11245	-18,01292079	27,73081193	El Hierro	Canary Isl.
SH3-30a	SH3	SH3-30	*Lobaria pulmonaria*	11249	-18,01291757	27,73082972	El Hierro	Canary Isl.
SH3-30b	SH3	SH3-30	*Lobaria pulmonaria*	11250	-18,01291757	27,73082972	El Hierro	Canary Isl.
SH3-30d	SH3	SH3-30	*Lobaria pulmonaria*	11252	-18,01291757	27,73082972	El Hierro	Canary Isl.
SH3-31a	SH3	SH3-31	*Lobaria pulmonaria*	11253	-18,01287002	27,73086342	El Hierro	Canary Isl.
SH3-31b	SH3	SH3-31	*Lobaria pulmonaria*	11254	-18,01287002	27,73086342	El Hierro	Canary Isl.
SH3-31c	SH3	SH3-31	*Lobaria pulmonaria*	11255	-18,01287002	27,73086342	El Hierro	Canary Isl.
SH3-32a	SH3	SH3-32	*Lobaria pulmonaria*	11259	-18,01291864	27,7308762	El Hierro	Canary Isl.
SH3-04	SH3	SH3-4	*Lobaria pulmonaria*	11172	-18,01177608	27,73217366	El Hierro	Canary Isl.
SH3-06a	SH3	SH3-6	*Lobaria pulmonaria*	11175	-18,01182877	27,73213403	El Hierro	Canary Isl.
SH3-06b	SH3	SH3-6	*Lobaria pulmonaria*	11176	-18,01182877	27,73213403	El Hierro	Canary Isl.
SH3-06d	SH3	SH3-6	*Lobaria pulmonaria*	11178	-18,01182877	27,73213403	El Hierro	Canary Isl.
SH3-06e	SH3	SH3-6	*Lobaria pulmonaria*	11179	-18,01182877	27,73213403	El Hierro	Canary Isl.
SH3-07	SH3	SH3-7	*Lobaria pulmonaria*	11180	-18,01225952	27,7316325	El Hierro	Canary Isl.
SH3-08b	SH3	SH3-8	*Lobaria pulmonaria*	11182	-18,01229963	27,73145566	El Hierro	Canary Isl.
SH3-08c	SH3	SH3-8	*Lobaria pulmonaria*	11183	-18,01229963	27,73145566	El Hierro	Canary Isl.
SH3-09	SH3	SH3-9	*Lobaria pulmonaria*	11184	-18,01230135	27,73139255	El Hierro	Canary Isl.
SM1-01e	SM1	SM1-1	*Lobaria pulmonaria*	14707	-25,78249	37,85132	Sao Miguel	Azores
SM1-01f	SM1	SM1-1	*Lobaria pulmonaria*	14708	-25,78249	37,85132	Sao Miguel	Azores
SM1-12e	SM1	SM1-12	*Lobaria pulmonaria*	14771	-25,77989887	37,84960013	Sao Miguel	Azores
SM1-16f	SM1	SM1-16	*Lobaria pulmonaria*	14800	-25,77921261	37,85037101	Sao Miguel	Azores

**ID**	**Pop**	**PopTree**	**Species**	**VoucherID**	**X**	**Y**	**Location**	**Area**
SM1-07e	SM1	SM1-7	*Lobaria pulmonaria*	14743	-25,78158	37,85164	Sao Miguel	Azores
SM1-08a	SM1	SM1-8	*Lobaria pulmonaria*	14746	-25,78155377	37,85158045	Sao Miguel	Azores
SM1-08b	SM1	SM1-8	*Lobaria pulmonaria*	14747	-25,78155377	37,85158045	Sao Miguel	Azores
SM1-09f	SM1	SM1-9	*Lobaria pulmonaria*	14753	-25,77992	37,84953	Sao Miguel	Azores
SM1-09g	SM1	SM1-9	*Lobaria pulmonaria*	14754	-25,77992	37,84953	Sao Miguel	Azores
SM2-10b	SM2	SM2-10	*Lobaria pulmonaria*	14861	-25,32515881	37,75631884	Sao Miguel	Azores
SM2-10c	SM2	SM2-10	*Lobaria pulmonaria*	14862	-25,32515881	37,75631884	Sao Miguel	Azores
SM2-10d	SM2	SM2-10	*Lobaria pulmonaria*	14863	-25,32515881	37,75631884	Sao Miguel	Azores
SM2-12a	SM2	SM2-12	*Lobaria pulmonaria*	14867	-25,32533161	37,75598804	Sao Miguel	Azores
SM2-12b	SM2	SM2-12	*Lobaria pulmonaria*	14868	-25,32533161	37,75598804	Sao Miguel	Azores
SM2-12c	SM2	SM2-12	*Lobaria pulmonaria*	14869	-25,32533161	37,75598804	Sao Miguel	Azores
SM2-16a	SM2	SM2-16	*Lobaria pulmonaria*	14881	-25,32552647	37,75540774	Sao Miguel	Azores
SM2-16b	SM2	SM2-16	*Lobaria pulmonaria*	14882	-25,32552647	37,75540774	Sao Miguel	Azores
SM2-16c	SM2	SM2-16	*Lobaria pulmonaria*	14883	-25,32552647	37,75540774	Sao Miguel	Azores
SM2-16d	SM2	SM2-16	*Lobaria pulmonaria*	14884	-25,32552647	37,75540774	Sao Miguel	Azores
SM2-16e	SM2	SM2-16	*Lobaria pulmonaria*	14885	-25,32552647	37,75540774	Sao Miguel	Azores
SM2-16g	SM2	SM2-16	*Lobaria pulmonaria*	14887	-25,32552647	37,75540774	Sao Miguel	Azores
SM2-18a	SM2	SM2-18	*Lobaria pulmonaria*	14889	-25,32545216	37,75415767	Sao Miguel	Azores
SM2-18b	SM2	SM2-18	*Lobaria pulmonaria*	14890	-25,32545216	37,75415767	Sao Miguel	Azores
SM2-18c	SM2	SM2-18	*Lobaria pulmonaria*	14891	-25,32545216	37,75415767	Sao Miguel	Azores
SM2-18d	SM2	SM2-18	*Lobaria pulmonaria*	14892	-25,32545216	37,75415767	Sao Miguel	Azores
SM2-20a	SM2	SM2-20	*Lobaria pulmonaria*	14903	-25,32571252	37,75370743	Sao Miguel	Azores
SM2-20d	SM2	SM2-20	*Lobaria pulmonaria*	14906	-25,32571252	37,75370743	Sao Miguel	Azores
SM2-23a	SM2	SM2-23	*Lobaria pulmonaria*	14921	-25,32586543	37,75346595	Sao Miguel	Azores
SM2-24b	SM2	SM2-24	*Lobaria pulmonaria*	14925	-25,32589133	37,75340631	Sao Miguel	Azores
SM2-24c	SM2	SM2-24	*Lobaria pulmonaria*	14926	-25,32589133	37,75340631	Sao Miguel	Azores
SM2-25a	SM2	SM2-25	*Lobaria pulmonaria*	14928	-25,32595399	37,7532331	Sao Miguel	Azores

ID	Pop	PopTree	Species	VoucherID	X	Y	Location	Area
SM2-28d	SM2	SM2-28	*Lobaria pulmonaria*	14942	-25,32603949	37,75288059	Sao Miguel	Azores
SM2-29a	SM2	SM2-29	*Lobaria pulmonaria*	14943	-25,32609375	37,75265947	Sao Miguel	Azores
SM2-29b	SM2	SM2-29	*Lobaria pulmonaria*	14944	-25,32609375	37,75265947	Sao Miguel	Azores
SM2-29c	SM2	SM2-29	*Lobaria pulmonaria*	14945	-25,32609375	37,75265947	Sao Miguel	Azores
SM2-29d	SM2	SM2-29	*Lobaria pulmonaria*	14946	-25,32609375	37,75265947	Sao Miguel	Azores
SM2-29e	SM2	SM2-29	*Lobaria pulmonaria*	14947	-25,32609375	37,75265947	Sao Miguel	Azores
SM2-30c	SM2	SM2-30	*Lobaria pulmonaria*	14950	-25,32584	37,75173	Sao Miguel	Azores
SM2-05a	SM2	SM2-5	*Lobaria pulmonaria*	14838	-25,32509955	37,75666071	Sao Miguel	Azores
SM2-05b	SM2	SM2-5	*Lobaria pulmonaria*	14839	-25,32509955	37,75666071	Sao Miguel	Azores
SM2-05c	SM2	SM2-5	*Lobaria pulmonaria*	14840	-25,32509955	37,75666071	Sao Miguel	Azores
SM2-07d	SM2	SM2-7	*Lobaria pulmonaria*	14845	-25,32508411	37,75663662	Sao Miguel	Azores
SM2-07e	SM2	SM2-7	*Lobaria pulmonaria*	14846	-25,32508411	37,75663662	Sao Miguel	Azores
SM2-07f	SM2	SM2-7	*Lobaria pulmonaria*	14847	-25,32508411	37,75663662	Sao Miguel	Azores
SM2-07g	SM2	SM2-7	*Lobaria pulmonaria*	14848	-25,32508411	37,75663662	Sao Miguel	Azores
SM2-07h	SM2	SM2-7	*Lobaria pulmonaria*	14849	-25,32508411	37,75663662	Sao Miguel	Azores
SM2-07i	SM2	SM2-7	*Lobaria pulmonaria*	14850	-25,32508411	37,75663662	Sao Miguel	Azores
SM2-07j	SM2	SM2-7	*Lobaria pulmonaria*	14851	-25,32508411	37,75663662	Sao Miguel	Azores
SM2-08d	SM2	SM2-8	*Lobaria pulmonaria*	14855	-25,32519424	37,75647904	Sao Miguel	Azores
SM2-08e	SM2	SM2-8	*Lobaria pulmonaria*	14856	-25,32519424	37,75647904	Sao Miguel	Azores
SM2-08f	SM2	SM2-8	*Lobaria pulmonaria*	14857	-25,32519424	37,75647904	Sao Miguel	Azores
SM2-09a	SM2	SM2-9	*Lobaria pulmonaria*	14858	-25,32513889	37,75635122	Sao Miguel	Azores
SP1-01a	SP1	SP1-1	*Lobaria pulmonaria*	11584	-17,83511	28,6122	La Palma	Canary Isl.
SP1-01b	SP1	SP1-1	*Lobaria pulmonaria*	11585	-17,83511	28,6122	La Palma	Canary Isl.
SP1-01c	SP1	SP1-1	*Lobaria pulmonaria*	11586	-17,83511	28,6122	La Palma	Canary Isl.
SP1-10a	SP1	SP1-10	*Lobaria pulmonaria*	11619	-17,83530453	28,6118677	La Palma	Canary Isl.
SP1-10c	SP1	SP1-10	*Lobaria pulmonaria*	11621	-17,83530453	28,6118677	La Palma	Canary Isl.
SP1-11a	SP1	SP1-11	*Lobaria pulmonaria*	11622	-17,83528843	28,61179697	La Palma	Canary Isl.

ID	Pop	PopTree	Species	VoucherID	X	Y	Location	Area
SP1-11b	SP1	SP1-11	*Lobaria pulmonaria*	11623	-17,83528843	28,61179697	La Palma	Canary Isl.
SP1-11c	SP1	SP1-11	*Lobaria pulmonaria*	11624	-17,83528843	28,61179697	La Palma	Canary Isl.
SP1-11d	SP1	SP1-11	*Lobaria pulmonaria*	11625	-17,83528843	28,61179697	La Palma	Canary Isl.
SP1-11e	SP1	SP1-11	*Lobaria pulmonaria*	11626	-17,83528843	28,61179697	La Palma	Canary Isl.
SP1-11f	SP1	SP1-11	*Lobaria pulmonaria*	11627	-17,83528843	28,61179697	La Palma	Canary Isl.
SP1-12a	SP1	SP1-12	*Lobaria pulmonaria*	11628	-17,83524001	28,61178256	La Palma	Canary Isl.
SP1-12b	SP1	SP1-12	*Lobaria pulmonaria*	11629	-17,83524001	28,61178256	La Palma	Canary Isl.
SP1-12c	SP1	SP1-12	*Lobaria pulmonaria*	11630	-17,83524001	28,61178256	La Palma	Canary Isl.
SP1-12d	SP1	SP1-12	*Lobaria pulmonaria*	11631	-17,83524001	28,61178256	La Palma	Canary Isl.
SP1-12e	SP1	SP1-12	*Lobaria pulmonaria*	11632	-17,83524001	28,61178256	La Palma	Canary Isl.
SP1-13a	SP1	SP1-13	*Lobaria pulmonaria*	11633	-17,83522995	28,61173835	La Palma	Canary Isl.
SP1-13b	SP1	SP1-13	*Lobaria pulmonaria*	11634	-17,83522995	28,61173835	La Palma	Canary Isl.
SP1-13c	SP1	SP1-13	*Lobaria pulmonaria*	11635	-17,83522995	28,61173835	La Palma	Canary Isl.
SP1-13d	SP1	SP1-13	*Lobaria pulmonaria*	11636	-17,83522995	28,61173835	La Palma	Canary Isl.
SP1-13e	SP1	SP1-13	*Lobaria pulmonaria*	11637	-17,83522995	28,61173835	La Palma	Canary Isl.
SP1-14a	SP1	SP1-14	*Lobaria pulmonaria*	11640	-17,83512779	28,61174049	La Palma	Canary Isl.
SP1-14b	SP1	SP1-14	*Lobaria pulmonaria*	11641	-17,83512779	28,61174049	La Palma	Canary Isl.
SP1-14c	SP1	SP1-14	*Lobaria pulmonaria*	11642	-17,83512779	28,61174049	La Palma	Canary Isl.
SP1-15a	SP1	SP1-15	*Lobaria pulmonaria*	11643	-17,8351012	28,61170198	La Palma	Canary Isl.
SP1-15b	SP1	SP1-15	*Lobaria pulmonaria*	11644	-17,8351012	28,61170198	La Palma	Canary Isl.
SP1-15c	SP1	SP1-15	*Lobaria pulmonaria*	11645	-17,8351012	28,61170198	La Palma	Canary Isl.
SP1-15d	SP1	SP1-15	*Lobaria pulmonaria*	11646	-17,8351012	28,61170198	La Palma	Canary Isl.
SP1-16a	SP1	SP1-16	*Lobaria pulmonaria*	11647	-17,83511655	28,61161283	La Palma	Canary Isl.
SP1-16b	SP1	SP1-16	*Lobaria pulmonaria*	11648	-17,83511655	28,61161283	La Palma	Canary Isl.
SP1-16c	SP1	SP1-16	*Lobaria pulmonaria*	11649	-17,83511655	28,61161283	La Palma	Canary Isl.
SP1-16d	SP1	SP1-16	*Lobaria pulmonaria*	11650	-17,83511655	28,61161283	La Palma	Canary Isl.
SP1-17a	SP1	SP1-17	*Lobaria pulmonaria*	11651	-17,83513288	28,61157011	La Palma	Canary Isl.

ID	Pop	PopTree	Species	VoucherID	X	Y	Location	Area
SP1-17b	SP1	SP1-17	*Lobaria pulmonaria*	11652	-17,83513288	28,61157011	La Palma	Canary Isl.
SP1-17c	SP1	SP1-17	*Lobaria pulmonaria*	11653	-17,83513288	28,61157011	La Palma	Canary Isl.
SP1-18a	SP1	SP1-18	*Lobaria pulmonaria*	11658	-17,83511276	28,61148169	La Palma	Canary Isl.
SP1-18b	SP1	SP1-18	*Lobaria pulmonaria*	11659	-17,83511276	28,61148169	La Palma	Canary Isl.
SP1-18c	SP1	SP1-18	*Lobaria pulmonaria*	11660	-17,83511276	28,61148169	La Palma	Canary Isl.
SP1-18d	SP1	SP1-18	*Lobaria pulmonaria*	11661	-17,83511276	28,61148169	La Palma	Canary Isl.
SP1-19a	SP1	SP1-19	*Lobaria pulmonaria*	11662	-17,835079	28,61144785	La Palma	Canary Isl.
SP1-19b	SP1	SP1-19	*Lobaria pulmonaria*	11663	-17,835079	28,61144785	La Palma	Canary Isl.
SP1-19c	SP1	SP1-19	*Lobaria pulmonaria*	11664	-17,835079	28,61144785	La Palma	Canary Isl.
SP1-19d	SP1	SP1-19	*Lobaria pulmonaria*	11665	-17,835079	28,61144785	La Palma	Canary Isl.
SP1-19e	SP1	SP1-19	*Lobaria pulmonaria*	11666	-17,835079	28,61144785	La Palma	Canary Isl.
SP1-19f	SP1	SP1-19	*Lobaria pulmonaria*	11667	-17,835079	28,61144785	La Palma	Canary Isl.
SP1-02a	SP1	SP1-2	*Lobaria pulmonaria*	11587	-17,83512633	28,61215728	La Palma	Canary Isl.
SP1-02c	SP1	SP1-2	*Lobaria pulmonaria*	11589	-17,83512633	28,61215728	La Palma	Canary Isl.
SP1-02d	SP1	SP1-2	*Lobaria pulmonaria*	11590	-17,83512633	28,61215728	La Palma	Canary Isl.
SP1-20a	SP1	SP1-20	*Lobaria pulmonaria*	11668	-17,83510203	28,61131411	La Palma	Canary Isl.
SP1-20b	SP1	SP1-20	*Lobaria pulmonaria*	11669	-17,83510203	28,61131411	La Palma	Canary Isl.
SP1-20c	SP1	SP1-20	*Lobaria pulmonaria*	11670	-17,83510203	28,61131411	La Palma	Canary Isl.
SP1-20d	SP1	SP1-20	*Lobaria pulmonaria*	11671	-17,83510203	28,61131411	La Palma	Canary Isl.
SP1-21a	SP1	SP1-21	*Lobaria pulmonaria*	11675	-17,83510178	28,6113051	La Palma	Canary Isl.
SP1-21b	SP1	SP1-21	*Lobaria pulmonaria*	11676	-17,83510178	28,6113051	La Palma	Canary Isl.
SP1-21c	SP1	SP1-21	*Lobaria pulmonaria*	11677	-17,83510178	28,6113051	La Palma	Canary Isl.
SP1-21g	SP1	SP1-21	*Lobaria pulmonaria*	11681	-17,83510178	28,6113051	La Palma	Canary Isl.
SP1-22a	SP1	SP1-22	*Lobaria pulmonaria*	11683	-17,8350973	28,61130293	La Palma	Canary Isl.
SP1-22b	SP1	SP1-22	*Lobaria pulmonaria*	11684	-17,8350973	28,61130293	La Palma	Canary Isl.
SP1-22c	SP1	SP1-22	*Lobaria pulmonaria*	11685	-17,8350973	28,61130293	La Palma	Canary Isl.
SP1-24a	SP1	SP1-24	*Lobaria pulmonaria*	11690	-17,83506033	28,61124576	La Palma	Canary Isl.

ID	Pop	PopTree	Species	VoucherID	X	Y	Location	Area
SP1-24b	SP1	SP1-24	*Lobaria pulmonaria*	11691	-17,83506033	28,61124576	La Palma	Canary Isl.
SP1-24c	SP1	SP1-24	*Lobaria pulmonaria*	11692	-17,83506033	28,61124576	La Palma	Canary Isl.
SP1-24e	SP1	SP1-24	*Lobaria pulmonaria*	11694	-17,83506033	28,61124576	La Palma	Canary Isl.
SP1-24f	SP1	SP1-24	*Lobaria pulmonaria*	11695	-17,83506033	28,61124576	La Palma	Canary Isl.
SP1-24g	SP1	SP1-24	*Lobaria pulmonaria*	11696	-17,83506033	28,61124576	La Palma	Canary Isl.
SP1-25a	SP1	SP1-25	*Lobaria pulmonaria*	11698	-17,83505832	28,61123693	La Palma	Canary Isl.
SP1-25b	SP1	SP1-25	*Lobaria pulmonaria*	11699	-17,83505832	28,61123693	La Palma	Canary Isl.
SP1-26a	SP1	SP1-26	*Lobaria pulmonaria*	11705	-17,83501347	28,61121531	La Palma	Canary Isl.
SP1-26b	SP1	SP1-26	*Lobaria pulmonaria*	11706	-17,83501347	28,61121531	La Palma	Canary Isl.
SP1-26g	SP1	SP1-26	*Lobaria pulmonaria*	11711	-17,83501347	28,61121531	La Palma	Canary Isl.
SP1-26k	SP1	SP1-26	*Lobaria pulmonaria*	11715	-17,83501347	28,61121531	La Palma	Canary Isl.
SP1-26l	SP1	SP1-26	*Lobaria pulmonaria*	11716	-17,83501347	28,61121531	La Palma	Canary Isl.
SP1-27a	SP1	SP1-27	*Lobaria pulmonaria*	11717	-17,83498283	28,61121595	La Palma	Canary Isl.
SP1-27b	SP1	SP1-27	*Lobaria pulmonaria*	11718	-17,83498283	28,61121595	La Palma	Canary Isl.
SP1-27c	SP1	SP1-27	*Lobaria pulmonaria*	11719	-17,83498283	28,61121595	La Palma	Canary Isl.
SP1-27d	SP1	SP1-27	*Lobaria pulmonaria*	11720	-17,83498283	28,61121595	La Palma	Canary Isl.
SP1-27f	SP1	SP1-27	*Lobaria pulmonaria*	11722	-17,83498283	28,61121595	La Palma	Canary Isl.
SP1-28a	SP1	SP1-28	*Lobaria pulmonaria*	11723	-17,83493231	28,61120917	La Palma	Canary Isl.
SP1-29f	SP1	SP1-29	*Lobaria pulmonaria*	11735	-17,83488747	28,61118756	La Palma	Canary Isl.
SP1-03a	SP1	SP1-3	*Lobaria pulmonaria*	11591	-17,83505458	28,6121227	La Palma	Canary Isl.
SP1-03b	SP1	SP1-3	*Lobaria pulmonaria*	11592	-17,83505458	28,6121227	La Palma	Canary Isl.
SP1-03c	SP1	SP1-3	*Lobaria pulmonaria*	11593	-17,83505458	28,6121227	La Palma	Canary Isl.
SP1-04a	SP1	SP1-4	*Lobaria pulmonaria*	11594	-17,83507907	28,61208313	La Palma	Canary Isl.
SP1-04b	SP1	SP1-4	*Lobaria pulmonaria*	11595	-17,83507907	28,61208313	La Palma	Canary Isl.
SP1-05a	SP1	SP1-5	*Lobaria pulmonaria*	11597	-17,83510356	28,61204355	La Palma	Canary Isl.
SP1-05b	SP1	SP1-5	*Lobaria pulmonaria*	11598	-17,83510356	28,61204355	La Palma	Canary Isl.
SP1-05c	SP1	SP1-5	*Lobaria pulmonaria*	11599	-17,83510356	28,61204355	La Palma	Canary Isl.

ID	Pop	PopTree	Species	VoucherID	X	Y	Location	Area
SP1-05d	SP1	SP1-5	*Lobaria pulmonaria*	11600	-17,83510356	28,61204355	La Palma	Canary Isl.
SP1-05e	SP1	SP1-5	*Lobaria pulmonaria*	11601	-17,83510356	28,61204355	La Palma	Canary Isl.
SP1-06a	SP1	SP1-6	*Lobaria pulmonaria*	11602	-17,83514275	28,61198023	La Palma	Canary Isl.
SP1-06b	SP1	SP1-6	*Lobaria pulmonaria*	11603	-17,83514275	28,61198023	La Palma	Canary Isl.
SP1-06c	SP1	SP1-6	*Lobaria pulmonaria*	11604	-17,83514275	28,61198023	La Palma	Canary Isl.
SP1-06d	SP1	SP1-6	*Lobaria pulmonaria*	11605	-17,83514275	28,61198023	La Palma	Canary Isl.
SP1-06e	SP1	SP1-6	*Lobaria pulmonaria*	11606	-17,83514275	28,61198023	La Palma	Canary Isl.
SP1-07a	SP1	SP1-7	*Lobaria pulmonaria*	11607	-17,83519034	28,61196381	La Palma	Canary Isl.
SP1-07b	SP1	SP1-7	*Lobaria pulmonaria*	11608	-17,83519034	28,61196381	La Palma	Canary Isl.
SP1-07c	SP1	SP1-7	*Lobaria pulmonaria*	11609	-17,83519034	28,61196381	La Palma	Canary Isl.
SP1-08a	SP1	SP1-8	*Lobaria pulmonaria*	11610	-17,83522952	28,6119005	La Palma	Canary Isl.
SP1-08b	SP1	SP1-8	*Lobaria pulmonaria*	11611	-17,83522952	28,6119005	La Palma	Canary Isl.
SP1-08c	SP1	SP1-8	*Lobaria pulmonaria*	11612	-17,83522952	28,6119005	La Palma	Canary Isl.
SP1-08d	SP1	SP1-8	*Lobaria pulmonaria*	11613	-17,83522952	28,6119005	La Palma	Canary Isl.
SP1-08e	SP1	SP1-8	*Lobaria pulmonaria*	11614	-17,83522952	28,6119005	La Palma	Canary Isl.
SP1-09a	SP1	SP1-9	*Lobaria pulmonaria*	11615	-17,83528004	28,61190727	La Palma	Canary Isl.
SP1-09b	SP1	SP1-9	*Lobaria pulmonaria*	11616	-17,83528004	28,61190727	La Palma	Canary Isl.
SP1-09c	SP1	SP1-9	*Lobaria pulmonaria*	11617	-17,83528004	28,61190727	La Palma	Canary Isl.
SP3-13	SP3	SP3-13	*Lobaria pulmonaria*	10846	-17,80440837	28,78842451	La Palma	Canary Isl.
SP4-01a	SP4	SP4-1	*Lobaria pulmonaria*	11757	-17,85016	28,80232	La Palma	Canary Isl.
SP4-01b	SP4	SP4-1	*Lobaria pulmonaria*	11758	-17,85016	28,80232	La Palma	Canary Isl.
SP4-01c	SP4	SP4-1	*Lobaria pulmonaria*	11759	-17,85016	28,80232	La Palma	Canary Isl.
SP4-01d	SP4	SP4-1	*Lobaria pulmonaria*	11760	-17,85016	28,80232	La Palma	Canary Isl.
SP4-01e	SP4	SP4-1	*Lobaria pulmonaria*	11761	-17,85016	28,80232	La Palma	Canary Isl.
SP4-10a	SP4	SP4-10	*Lobaria pulmonaria*	11787	-17,85094647	28,80260108	La Palma	Canary Isl.
SP4-10b	SP4	SP4-10	*Lobaria pulmonaria*	11788	-17,85094647	28,80260108	La Palma	Canary Isl.
SP4-10c	SP4	SP4-10	*Lobaria pulmonaria*	11789	-17,85094647	28,80260108	La Palma	Canary Isl.

ID	Pop	PopTree	Species	VoucherID	X	Y	Location	Area
SP4-11a	SP4	SP4-11	*Lobaria pulmonaria*	11790	-17,85099414	28,80258465	La Palma	Canary Isl.
SP4-11b	SP4	SP4-11	*Lobaria pulmonaria*	11791	-17,85099414	28,80258465	La Palma	Canary Isl.
SP4-11c	SP4	SP4-11	*Lobaria pulmonaria*	11792	-17,85099414	28,80258465	La Palma	Canary Isl.
SP4-12a	SP4	SP4-12	*Lobaria pulmonaria*	11793	-17,85109649	28,80258249	La Palma	Canary Isl.
SP4-12b	SP4	SP4-12	*Lobaria pulmonaria*	11794	-17,85109649	28,80258249	La Palma	Canary Isl.
SP4-12c	SP4	SP4-12	*Lobaria pulmonaria*	11795	-17,85109649	28,80258249	La Palma	Canary Isl.
SP4-13a	SP4	SP4-13	*Lobaria pulmonaria*	11796	-17,85055731	28,80232328	La Palma	Canary Isl.
SP4-13b	SP4	SP4-13	*Lobaria pulmonaria*	11797	-17,85055731	28,80232328	La Palma	Canary Isl.
SP4-13c	SP4	SP4-13	*Lobaria pulmonaria*	11798	-17,85055731	28,80232328	La Palma	Canary Isl.
SP4-13d	SP4	SP4-13	*Lobaria pulmonaria*	11799	-17,85055731	28,80232328	La Palma	Canary Isl.
SP4-14b	SP4	SP4-14	*Lobaria pulmonaria*	11801	-17,85055731	28,80232328	La Palma	Canary Isl.
SP4-15a	SP4	SP4-15	*Lobaria pulmonaria*	11803	-17,85055392	28,80231989	La Palma	Canary Isl.
SP4-15b	SP4	SP4-15	*Lobaria pulmonaria*	11804	-17,85055392	28,80231989	La Palma	Canary Isl.
SP4-15c	SP4	SP4-15	*Lobaria pulmonaria*	11805	-17,85055392	28,80231989	La Palma	Canary Isl.
SP4-16a	SP4	SP4-16	*Lobaria pulmonaria*	11806	-17,85045859	28,80235276	La Palma	Canary Isl.
SP4-16b	SP4	SP4-16	*Lobaria pulmonaria*	11807	-17,85045859	28,80235276	La Palma	Canary Isl.
SP4-16c	SP4	SP4-16	*Lobaria pulmonaria*	11808	-17,85045859	28,80235276	La Palma	Canary Isl.
SP4-17a	SP4	SP4-17	*Lobaria pulmonaria*	11809	-17,85055895	28,80233498	La Palma	Canary Isl.
SP4-17b	SP4	SP4-17	*Lobaria pulmonaria*	11810	-17,85055895	28,80233498	La Palma	Canary Isl.
SP4-17c	SP4	SP4-17	*Lobaria pulmonaria*	11811	-17,85055895	28,80233498	La Palma	Canary Isl.
SP4-17d	SP4	SP4-17	*Lobaria pulmonaria*	11812	-17,85055895	28,80233498	La Palma	Canary Isl.
SP4-17e	SP4	SP4-17	*Lobaria pulmonaria*	11813	-17,85055895	28,80233498	La Palma	Canary Isl.
SP4-18a	SP4	SP4-18	*Lobaria pulmonaria*	11814	-17,85064882	28,80237818	La Palma	Canary Isl.
SP4-18b	SP4	SP4-18	*Lobaria pulmonaria*	11815	-17,85064882	28,80237818	La Palma	Canary Isl.
SP4-18c	SP4	SP4-18	*Lobaria pulmonaria*	11816	-17,85064882	28,80237818	La Palma	Canary Isl.
SP4-19a	SP4	SP4-19	*Lobaria pulmonaria*	11817	-17,85067893	28,80237284	La Palma	Canary Isl.
SP4-19b	SP4	SP4-19	*Lobaria pulmonaria*	11818	-17,85067893	28,80237284	La Palma	Canary Isl.

ID	Pop	PopTree	Species	VoucherID	X	Y	Location	Area
SP4-19c	SP4	SP4-19	*Lobaria pulmonaria*	11819	-17,85067893	28,80237284	La Palma	Canary Isl.
SP4-19d	SP4	SP4-19	*Lobaria pulmonaria*	11820	-17,85067893	28,80237284	La Palma	Canary Isl.
SP4-02a	SP4	SP4-2	*Lobaria pulmonaria*	11762	-17,850363	28,802523	La Palma	Canary Isl.
SP4-02b	SP4	SP4-2	*Lobaria pulmonaria*	11763	-17,850363	28,802523	La Palma	Canary Isl.
SP4-02e	SP4	SP4-2	*Lobaria pulmonaria*	11766	-17,850363	28,802523	La Palma	Canary Isl.
SP4-20a	SP4	SP4-20	*Lobaria pulmonaria*	11821	-17,85068874	28,80234721	La Palma	Canary Isl.
SP4-20b	SP4	SP4-20	*Lobaria pulmonaria*	11822	-17,85068874	28,80234721	La Palma	Canary Isl.
SP4-20c	SP4	SP4-20	*Lobaria pulmonaria*	11823	-17,85068874	28,80234721	La Palma	Canary Isl.
SP4-20d	SP4	SP4-20	*Lobaria pulmonaria*	11824	-17,85068874	28,80234721	La Palma	Canary Isl.
SP4-20e	SP4	SP4-20	*Lobaria pulmonaria*	11825	-17,85068874	28,80234721	La Palma	Canary Isl.
SP4-21a	SP4	SP4-21	*Lobaria pulmonaria*	11826	-17,8507786	28,80239041	La Palma	Canary Isl.
SP4-21b	SP4	SP4-21	*Lobaria pulmonaria*	11827	-17,8507786	28,80239041	La Palma	Canary Isl.
SP4-21c	SP4	SP4-21	*Lobaria pulmonaria*	11828	-17,8507786	28,80239041	La Palma	Canary Isl.
SP4-21d	SP4	SP4-21	*Lobaria pulmonaria*	11829	-17,8507786	28,80239041	La Palma	Canary Isl.
SP4-21e	SP4	SP4-21	*Lobaria pulmonaria*	11830	-17,8507786	28,80239041	La Palma	Canary Isl.
SP4-03	SP4	SP4-3	*Lobaria pulmonaria*	11767	-17,85051668	28,80240384	La Palma	Canary Isl.
SP4-04	SP4	SP4-4	*Lobaria pulmonaria*	11768	-17,85091656	28,80268521	La Palma	Canary Isl.
SP4-05c	SP4	SP4-5	*Lobaria pulmonaria*	11771	-17,85078875	28,80282615	La Palma	Canary Isl.
SP4-05d	SP4	SP4-5	*Lobaria pulmonaria*	11772	-17,85078875	28,80282615	La Palma	Canary Isl.
SP4-06a	SP4	SP4-6	*Lobaria pulmonaria*	11773	-17,85082258	28,80285998	La Palma	Canary Isl.
SP4-06b	SP4	SP4-6	*Lobaria pulmonaria*	11774	-17,85082258	28,80285998	La Palma	Canary Isl.
SP4-06c	SP4	SP4-6	*Lobaria pulmonaria*	11775	-17,85082258	28,80285998	La Palma	Canary Isl.
SP4-06d	SP4	SP4-6	*Lobaria pulmonaria*	11776	-17,85082258	28,80285998	La Palma	Canary Isl.
SP4-07a	SP4	SP4-7	*Lobaria pulmonaria*	11777	-17,85083857	28,80288308	La Palma	Canary Isl.
SP4-07b	SP4	SP4-7	*Lobaria pulmonaria*	11778	-17,85083857	28,80288308	La Palma	Canary Isl.
SP4-08a	SP4	SP4-8	*Lobaria pulmonaria*	11780	-17,85090397	28,80271217	La Palma	Canary Isl.
SP4-08b	SP4	SP4-8	*Lobaria pulmonaria*	11781	-17,85090397	28,80271217	La Palma	Canary Isl.

ID	Pop	PopTree	Species	VoucherID	X	Y	Location	Area
SP4-08c	SP4	SP4-8	*Lobaria pulmonaria*	11782	-17,85090397	28,80271217	La Palma	Canary Isl.
SP4-08d	SP4	SP4-8	*Lobaria pulmonaria*	11783	-17,85090397	28,80271217	La Palma	Canary Isl.
SP4-09a	SP4	SP4-9	*Lobaria pulmonaria*	11784	-17,85091377	28,80268654	La Palma	Canary Isl.
SP4-09b	SP4	SP4-9	*Lobaria pulmonaria*	11785	-17,85091377	28,80268654	La Palma	Canary Isl.
SP5-01a	SP5	SP5-1	*Lobaria pulmonaria*	11831	-17,79069915	28,72601296	La Palma	Canary Isl.
SP5-01b	SP5	SP5-1	*Lobaria pulmonaria*	11832	-17,79069915	28,72601296	La Palma	Canary Isl.
SP5-01c	SP5	SP5-1	*Lobaria pulmonaria*	11833	-17,79069915	28,72601296	La Palma	Canary Isl.
SP5-10a	SP5	SP5-10	*Lobaria pulmonaria*	11866	-17,79174995	28,72426494	La Palma	Canary Isl.
SP5-10b	SP5	SP5-10	*Lobaria pulmonaria*	11867	-17,79174995	28,72426494	La Palma	Canary Isl.
SP5-10c	SP5	SP5-10	*Lobaria pulmonaria*	11868	-17,79174995	28,72426494	La Palma	Canary Isl.
SP5-10d	SP5	SP5-10	*Lobaria pulmonaria*	11869	-17,79174995	28,72426494	La Palma	Canary Isl.
SP5-10e	SP5	SP5-10	*Lobaria pulmonaria*	11870	-17,79174995	28,72426494	La Palma	Canary Isl.
SP5-10g	SP5	SP5-10	*Lobaria pulmonaria*	11872	-17,79174995	28,72426494	La Palma	Canary Isl.
SP5-10h	SP5	SP5-10	*Lobaria pulmonaria*	11873	-17,79174995	28,72426494	La Palma	Canary Isl.
SP5-10i	SP5	SP5-10	*Lobaria pulmonaria*	11874	-17,79174995	28,72426494	La Palma	Canary Isl.
SP5-11a	SP5	SP5-11	*Lobaria pulmonaria*	11875	-17,79164965	28,72428267	La Palma	Canary Isl.
SP5-11b	SP5	SP5-11	*Lobaria pulmonaria*	11876	-17,79164965	28,72428267	La Palma	Canary Isl.
SP5-11c	SP5	SP5-11	*Lobaria pulmonaria*	11877	-17,79164965	28,72428267	La Palma	Canary Isl.
SP5-12a	SP5	SP5-12	*Lobaria pulmonaria*	11878	-17,79161586	28,72424882	La Palma	Canary Isl.
SP5-12b	SP5	SP5-12	*Lobaria pulmonaria*	11879	-17,79161586	28,72424882	La Palma	Canary Isl.
SP5-13a	SP5	SP5-13	*Lobaria pulmonaria*	11880	-17,79161485	28,7242444	La Palma	Canary Isl.
SP5-13b	SP5	SP5-13	*Lobaria pulmonaria*	11881	-17,79161485	28,7242444	La Palma	Canary Isl.
SP5-14a	SP5	SP5-14	*Lobaria pulmonaria*	11882	-17,7916541	28,7241811	La Palma	Canary Isl.
SP5-14b	SP5	SP5-14	*Lobaria pulmonaria*	11883	-17,7916541	28,7241811	La Palma	Canary Isl.
SP5-14c	SP5	SP5-14	*Lobaria pulmonaria*	11884	-17,7916541	28,7241811	La Palma	Canary Isl.
SP5-15a	SP5	SP5-15	*Lobaria pulmonaria*	11885	-17,79157729	28,72424067	La Palma	Canary Isl.
SP5-15b	SP5	SP5-15	*Lobaria pulmonaria*	11886	-17,79157729	28,72424067	La Palma	Canary Isl.

ID	Pop	PopTree	Species	VoucherID	X	Y	Location	Area
SP5-15c	SP5	SP5-15	*Lobaria pulmonaria*	11887	-17,79157729	28,72424067	La Palma	Canary Isl.
SP5-15d	SP5	SP5-15	*Lobaria pulmonaria*	11888	-17,79157729	28,72424067	La Palma	Canary Isl.
SP5-15e	SP5	SP5-15	*Lobaria pulmonaria*	11889	-17,79157729	28,72424067	La Palma	Canary Isl.
SP5-15f	SP5	SP5-15	*Lobaria pulmonaria*	11890	-17,79157729	28,72424067	La Palma	Canary Isl.
SP5-16a	SP5	SP5-16	*Lobaria pulmonaria*	11891	-17,79152883	28,72422624	La Palma	Canary Isl.
SP5-16b	SP5	SP5-16	*Lobaria pulmonaria*	11892	-17,79152883	28,72422624	La Palma	Canary Isl.
SP5-16c	SP5	SP5-16	*Lobaria pulmonaria*	11893	-17,79152883	28,72422624	La Palma	Canary Isl.
SP5-16d	SP5	SP5-16	*Lobaria pulmonaria*	11894	-17,79152883	28,72422624	La Palma	Canary Isl.
SP5-17a	SP5	SP5-17	*Lobaria pulmonaria*	11895	-17,79150222	28,72418773	La Palma	Canary Isl.
SP5-17b	SP5	SP5-17	*Lobaria pulmonaria*	11896	-17,79150222	28,72418773	La Palma	Canary Isl.
SP5-17c	SP5	SP5-17	*Lobaria pulmonaria*	11897	-17,79150222	28,72418773	La Palma	Canary Isl.
SP5-17d	SP5	SP5-17	*Lobaria pulmonaria*	11898	-17,79150222	28,72418773	La Palma	Canary Isl.
SP5-18a	SP5	SP5-18	*Lobaria pulmonaria*	11899	-17,79145458	28,72420414	La Palma	Canary Isl.
SP5-18b	SP5	SP5-18	*Lobaria pulmonaria*	11900	-17,79145458	28,72420414	La Palma	Canary Isl.
SP5-18c	SP5	SP5-18	*Lobaria pulmonaria*	11901	-17,79145458	28,72420414	La Palma	Canary Isl.
SP5-19a	SP5	SP5-19	*Lobaria pulmonaria*	11902	-17,79148076	28,72413579	La Palma	Canary Isl.
SP5-19b	SP5	SP5-19	*Lobaria pulmonaria*	11903	-17,79148076	28,72413579	La Palma	Canary Isl.
SP5-19c	SP5	SP5-19	*Lobaria pulmonaria*	11904	-17,79148076	28,72413579	La Palma	Canary Isl.
SP5-19d	SP5	SP5-19	*Lobaria pulmonaria*	11905	-17,79148076	28,72413579	La Palma	Canary Isl.
SP5-19e	SP5	SP5-19	*Lobaria pulmonaria*	11906	-17,79148076	28,72413579	La Palma	Canary Isl.
SP5-19f	SP5	SP5-19	*Lobaria pulmonaria*	11907	-17,79148076	28,72413579	La Palma	Canary Isl.
SP5-19g	SP5	SP5-19	*Lobaria pulmonaria*	11908	-17,79148076	28,72413579	La Palma	Canary Isl.
SP5-19i	SP5	SP5-19	*Lobaria pulmonaria*	11910	-17,79148076	28,72413579	La Palma	Canary Isl.
SP5-02a	SP5	SP5-2	*Lobaria pulmonaria*	11834	-17,79078653	28,72596605	La Palma	Canary Isl.
SP5-02b	SP5	SP5-2	*Lobaria pulmonaria*	11835	-17,79078653	28,72596605	La Palma	Canary Isl.
SP5-02c	SP5	SP5-2	*Lobaria pulmonaria*	11836	-17,79078653	28,72596605	La Palma	Canary Isl.
SP5-20a	SP5	SP5-20	*Lobaria pulmonaria*	11911	-17,79137848	28,7241379	La Palma	Canary Isl.

ID	Pop	PopTree	Species	VoucherID	X	Y	Location	Area
SP5-20b	SP5	SP5-20	*Lobaria pulmonaria*	11912	-17,79137848	28,7241379	La Palma	Canary Isl.
SP5-20c	SP5	SP5-20	*Lobaria pulmonaria*	11913	-17,79137848	28,7241379	La Palma	Canary Isl.
SP5-20d	SP5	SP5-20	*Lobaria pulmonaria*	11914	-17,79137848	28,7241379	La Palma	Canary Isl.
SP5-20e	SP5	SP5-20	*Lobaria pulmonaria*	11915	-17,79137848	28,7241379	La Palma	Canary Isl.
SP5-21a	SP5	SP5-21	*Lobaria pulmonaria*	11916	-17,79141843	28,72416607	La Palma	Canary Isl.
SP5-21b	SP5	SP5-21	*Lobaria pulmonaria*	11917	-17,79141843	28,72416607	La Palma	Canary Isl.
SP5-21c	SP5	SP5-21	*Lobaria pulmonaria*	11918	-17,79141843	28,72416607	La Palma	Canary Isl.
SP5-22a	SP5	SP5-22	*Lobaria pulmonaria*	11919	-17,79135085	28,72409836	La Palma	Canary Isl.
SP5-22b	SP5	SP5-22	*Lobaria pulmonaria*	11920	-17,79135085	28,72409836	La Palma	Canary Isl.
SP5-22c	SP5	SP5-22	*Lobaria pulmonaria*	11921	-17,79135085	28,72409836	La Palma	Canary Isl.
SP5-22d	SP5	SP5-22	*Lobaria pulmonaria*	11922	-17,79135085	28,72409836	La Palma	Canary Isl.
SP5-22e	SP5	SP5-22	*Lobaria pulmonaria*	11923	-17,79135085	28,72409836	La Palma	Canary Isl.
SP5-23a	SP5	SP5-23	*Lobaria pulmonaria*	11924	-17,79119914	28,724078	La Palma	Canary Isl.
SP5-23d	SP5	SP5-23	*Lobaria pulmonaria*	11927	-17,79119914	28,724078	La Palma	Canary Isl.
SP5-23e	SP5	SP5-23	*Lobaria pulmonaria*	11928	-17,79119914	28,724078	La Palma	Canary Isl.
SP5-23f	SP5	SP5-23	*Lobaria pulmonaria*	11929	-17,79119914	28,724078	La Palma	Canary Isl.
SP5-23g	SP5	SP5-23	*Lobaria pulmonaria*	11930	-17,79119914	28,724078	La Palma	Canary Isl.
SP5-23j	SP5	SP5-23	*Lobaria pulmonaria*	11933	-17,79119914	28,724078	La Palma	Canary Isl.
SP5-03a	SP5	SP5-3	*Lobaria pulmonaria*	11837	-17,79086334	28,72590648	La Palma	Canary Isl.
SP5-03b	SP5	SP5-3	*Lobaria pulmonaria*	11838	-17,79086334	28,72590648	La Palma	Canary Isl.
SP5-03c	SP5	SP5-3	*Lobaria pulmonaria*	11839	-17,79086334	28,72590648	La Palma	Canary Isl.
SP5-04a	SP5	SP5-4	*Lobaria pulmonaria*	11840	-17,79096146	28,72574822	La Palma	Canary Isl.
SP5-04b	SP5	SP5-4	*Lobaria pulmonaria*	11841	-17,79096146	28,72574822	La Palma	Canary Isl.
SP5-04c	SP5	SP5-4	*Lobaria pulmonaria*	11842	-17,79096146	28,72574822	La Palma	Canary Isl.
SP5-05a	SP5	SP5-5	*Lobaria pulmonaria*	11843	-17,79112111	28,72597928	La Palma	Canary Isl.
SP5-05b	SP5	SP5-5	*Lobaria pulmonaria*	11844	-17,79112111	28,72597928	La Palma	Canary Isl.
SP5-05c	SP5	SP5-5	*Lobaria pulmonaria*	11845	-17,79112111	28,72597928	La Palma	Canary Isl.

ID	Pop	PopTree	Species	VoucherID	X	Y	Location	Area
SP5-05d	SP5	SP5-5	*Lobaria pulmonaria*	11846	-17,79112111	28,72597928	La Palma	Canary Isl.
SP5-06a	SP5	SP5-6	*Lobaria pulmonaria*	11847	-17,79102298	28,72613755	La Palma	Canary Isl.
SP5-06c	SP5	SP5-6	*Lobaria pulmonaria*	11849	-17,79102298	28,72613755	La Palma	Canary Isl.
SP5-07a	SP5	SP5-7	*Lobaria pulmonaria*	11850	-17,79151392	28,72485593	La Palma	Canary Isl.
SP5-07b	SP5	SP5-7	*Lobaria pulmonaria*	11851	-17,79151392	28,72485593	La Palma	Canary Isl.
SP5-07c	SP5	SP5-7	*Lobaria pulmonaria*	11852	-17,79151392	28,72485593	La Palma	Canary Isl.
SP5-08a	SP5	SP5-8	*Lobaria pulmonaria*	11854	-17,79158751	28,72473723	La Palma	Canary Isl.
SP5-08b	SP5	SP5-8	*Lobaria pulmonaria*	11855	-17,79158751	28,72473723	La Palma	Canary Isl.
SP5-08c	SP5	SP5-8	*Lobaria pulmonaria*	11856	-17,79158751	28,72473723	La Palma	Canary Isl.
SP5-08d	SP5	SP5-8	*Lobaria pulmonaria*	11857	-17,79158751	28,72473723	La Palma	Canary Isl.
SP5-08f	SP5	SP5-8	*Lobaria pulmonaria*	11859	-17,79158751	28,72473723	La Palma	Canary Isl.
SP5-08g	SP5	SP5-8	*Lobaria pulmonaria*	11860	-17,79158751	28,72473723	La Palma	Canary Isl.
SP5-09a	SP5	SP5-9	*Lobaria pulmonaria*	11862	-17,79175115	28,72431002	La Palma	Canary Isl.
SP5-09c	SP5	SP5-9	*Lobaria pulmonaria*	11864	-17,79175115	28,72431002	La Palma	Canary Isl.
ST1-01b	ST1	ST1-1	*Lobaria pulmonaria*	10878	-16,40779	28,42053	Tenerife	Canary Isl.
ST1-01c	ST1	ST1-1	*Lobaria pulmonaria*	10879	-16,40779	28,42053	Tenerife	Canary Isl.
ST1-10a	ST1	ST1-10	*Lobaria pulmonaria*	10903	-16,40794177	28,42032608	Tenerife	Canary Isl.
ST1-10b	ST1	ST1-10	*Lobaria pulmonaria*	10904	-16,40794177	28,42032608	Tenerife	Canary Isl.
ST1-10c	ST1	ST1-10	*Lobaria pulmonaria*	10905	-16,40794177	28,42032608	Tenerife	Canary Isl.
ST1-11b	ST1	ST1-11	*Lobaria pulmonaria*	10907	-16,40794684	28,4203133	Tenerife	Canary Isl.
ST1-11c	ST1	ST1-11	*Lobaria pulmonaria*	10908	-16,40794684	28,4203133	Tenerife	Canary Isl.
ST1-11f	ST1	ST1-11	*Lobaria pulmonaria*	10911	-16,40794684	28,4203133	Tenerife	Canary Isl.
ST1-12a	ST1	ST1-12	*Lobaria pulmonaria*	10913	-16,4079355	28,42026009	Tenerife	Canary Isl.
ST1-12b	ST1	ST1-12	*Lobaria pulmonaria*	10914	-16,4079355	28,42026009	Tenerife	Canary Isl.
ST1-12c	ST1	ST1-12	*Lobaria pulmonaria*	10915	-16,4079355	28,42026009	Tenerife	Canary Isl.
ST1-13f	ST1	ST1-13	*Lobaria pulmonaria*	10922	-16,40794169	28,42025291	Tenerife	Canary Isl.
ST1-14a	ST1	ST1-14	*Lobaria pulmonaria*	10923	-16,40800046	28,42026814	Tenerife	Canary Isl.

ID	Pop	PopTree	Species	VoucherID	X	Y	Location	Area
ST1-15a	ST1	ST1-15	*Lobaria pulmonaria*	10926	-16,40808781	28,420105	Tenerife	Canary Isl.
ST1-16a	ST1	ST1-16	*Lobaria pulmonaria*	10928	-16,40789763	28,42003934	Tenerife	Canary Isl.
ST1-16b	ST1	ST1-16	*Lobaria pulmonaria*	10929	-16,40789763	28,42003934	Tenerife	Canary Isl.
ST1-16c	ST1	ST1-16	*Lobaria pulmonaria*	10930	-16,40789763	28,42003934	Tenerife	Canary Isl.
ST1-17a	ST1	ST1-17	*Lobaria pulmonaria*	10931	-16,40789098	28,4200325	Tenerife	Canary Isl.
ST1-17b	ST1	ST1-17	*Lobaria pulmonaria*	10932	-16,40789098	28,4200325	Tenerife	Canary Isl.
ST1-17c	ST1	ST1-17	*Lobaria pulmonaria*	10933	-16,40789098	28,4200325	Tenerife	Canary Isl.
ST1-18a	ST1	ST1-18	*Lobaria pulmonaria*	10934	-16,40783776	28,41997774	Tenerife	Canary Isl.
ST1-18b	ST1	ST1-18	*Lobaria pulmonaria*	10935	-16,40783776	28,41997774	Tenerife	Canary Isl.
ST1-18c	ST1	ST1-18	*Lobaria pulmonaria*	10936	-16,40783776	28,41997774	Tenerife	Canary Isl.
ST1-19a	ST1	ST1-19	*Lobaria pulmonaria*	10937	-16,40780893	28,41991019	Tenerife	Canary Isl.
ST1-19b	ST1	ST1-19	*Lobaria pulmonaria*	10938	-16,40780893	28,41991019	Tenerife	Canary Isl.
ST1-19c	ST1	ST1-19	*Lobaria pulmonaria*	10939	-16,40780893	28,41991019	Tenerife	Canary Isl.
ST1-02a	ST1	ST1-2	*Lobaria pulmonaria*	10880	-16,40781604	28,42056881	Tenerife	Canary Isl.
ST1-02b	ST1	ST1-2	*Lobaria pulmonaria*	10881	-16,40781604	28,42056881	Tenerife	Canary Isl.
ST1-02c	ST1	ST1-2	*Lobaria pulmonaria*	10882	-16,40781604	28,42056881	Tenerife	Canary Isl.
ST1-20a	ST1	ST1-20	*Lobaria pulmonaria*	10940	-16,40771385	28,41987736	Tenerife	Canary Isl.
ST1-20b	ST1	ST1-20	*Lobaria pulmonaria*	10941	-16,40771385	28,41987736	Tenerife	Canary Isl.
ST1-20c	ST1	ST1-20	*Lobaria pulmonaria*	10942	-16,40771385	28,41987736	Tenerife	Canary Isl.
ST1-20d	ST1	ST1-20	*Lobaria pulmonaria*	10943	-16,40771385	28,41987736	Tenerife	Canary Isl.
ST1-20e	ST1	ST1-20	*Lobaria pulmonaria*	10944	-16,40771385	28,41987736	Tenerife	Canary Isl.
ST1-21a	ST1	ST1-21	*Lobaria pulmonaria*	10945	-16,40762485	28,41983316	Tenerife	Canary Isl.
ST1-21b	ST1	ST1-21	*Lobaria pulmonaria*	10946	-16,40762485	28,41983316	Tenerife	Canary Isl.
ST1-21c	ST1	ST1-21	*Lobaria pulmonaria*	10947	-16,40762485	28,41983316	Tenerife	Canary Isl.
ST1-22a	ST1	ST1-22	*Lobaria pulmonaria*	10950	-16,40767485	28,41975448	Tenerife	Canary Isl.
ST1-22b	ST1	ST1-22	*Lobaria pulmonaria*	10951	-16,40767485	28,41975448	Tenerife	Canary Isl.
ST1-22c	ST1	ST1-22	*Lobaria pulmonaria*	10952	-16,40767485	28,41975448	Tenerife	Canary Isl.

ID	Pop	PopTree	Species	VoucherID	X	Y	Location	Area
ST1-03a	ST1	ST1-3	*Lobaria pulmonaria*	10883	-16,40781305	28,42034321	Tenerife	Canary Isl.
ST1-03b	ST1	ST1-3	*Lobaria pulmonaria*	10884	-16,40781305	28,42034321	Tenerife	Canary Isl.
ST1-03c	ST1	ST1-3	*Lobaria pulmonaria*	10885	-16,40781305	28,42034321	Tenerife	Canary Isl.
ST1-04a	ST1	ST1-4	*Lobaria pulmonaria*	10886	-16,4078411	28,42030551	Tenerife	Canary Isl.
ST1-04b	ST1	ST1-4	*Lobaria pulmonaria*	10887	-16,4078411	28,42030551	Tenerife	Canary Isl.
ST1-05b	ST1	ST1-5	*Lobaria pulmonaria*	10889	-16,40787205	28,42036244	Tenerife	Canary Isl.
ST1-05c	ST1	ST1-5	*Lobaria pulmonaria*	10890	-16,40787205	28,42036244	Tenerife	Canary Isl.
ST1-06a	ST1	ST1-6	*Lobaria pulmonaria*	10891	-16,40791076	28,42033303	Tenerife	Canary Isl.
ST1-06b	ST1	ST1-6	*Lobaria pulmonaria*	10892	-16,40791076	28,42033303	Tenerife	Canary Isl.
ST1-06c	ST1	ST1-6	*Lobaria pulmonaria*	10893	-16,40791076	28,42033303	Tenerife	Canary Isl.
ST1-07a	ST1	ST1-7	*Lobaria pulmonaria*	10894	-16,40791779	28,42032101	Tenerife	Canary Isl.
ST1-07b	ST1	ST1-7	*Lobaria pulmonaria*	10895	-16,40791779	28,42032101	Tenerife	Canary Isl.
ST1-08a	ST1	ST1-8	*Lobaria pulmonaria*	10897	-16,40792591	28,42032649	Tenerife	Canary Isl.
ST1-08b	ST1	ST1-8	*Lobaria pulmonaria*	10898	-16,40792591	28,42032649	Tenerife	Canary Isl.
ST1-08c	ST1	ST1-8	*Lobaria pulmonaria*	10899	-16,40792591	28,42032649	Tenerife	Canary Isl.
ST1-09b	ST1	ST1-9	*Lobaria pulmonaria*	10901	-16,40793403	28,42033195	Tenerife	Canary Isl.
ST2-10e	ST2	ST2-10	*Lobaria pulmonaria*	10999	-16,81061959	28,32874269	Tenerife	Canary Isl.
ST2-18f	ST2	ST2-18	*Lobaria pulmonaria*	11040	-16,8106411	28,32854343	Tenerife	Canary Isl.
ST2-19d	ST2	ST2-19	*Lobaria pulmonaria*	11045	-16,81064606	28,32853555	Tenerife	Canary Isl.
ST2-19e	ST2	ST2-19	*Lobaria pulmonaria*	11046	-16,81064606	28,32853555	Tenerife	Canary Isl.
ST2-20a	ST2	ST2-20	*Lobaria pulmonaria*	11048	-16,81055077	28,32856768	Tenerife	Canary Isl.
ST2-20b	ST2	ST2-20	*Lobaria pulmonaria*	11049	-16,81055077	28,32856768	Tenerife	Canary Isl.
ST2-20c	ST2	ST2-20	*Lobaria pulmonaria*	11050	-16,81055077	28,32856768	Tenerife	Canary Isl.
ST2-20h	ST2	ST2-20	*Lobaria pulmonaria*	11298	-16,81055077	28,32856768	Tenerife	Canary Isl.
ST2-21a	ST2	ST2-21	*Lobaria pulmonaria*	11054	-16,81058421	28,32848244	Tenerife	Canary Isl.
ST2-22a	ST2	ST2-22	*Lobaria pulmonaria*	11058	-16,81063386	28,32840362	Tenerife	Canary Isl.
ST2-22b	ST2	ST2-22	*Lobaria pulmonaria*	11059	-16,81063386	28,32840362	Tenerife	Canary Isl.

ID	Pop	PopTree	Species	VoucherID	X	Y	Location	Area
ST2-22c	ST2	ST2-22	*Lobaria pulmonaria*	11060	-16,81063386	28,32840362	Tenerife	Canary Isl.
ST2-22d	ST2	ST2-22	*Lobaria pulmonaria*	11061	-16,81063386	28,32840362	Tenerife	Canary Isl.
ST2-23a	ST2	ST2-23	*Lobaria pulmonaria*	11062	-16,81063386	28,32840362	Tenerife	Canary Isl.
ST2-23b	ST2	ST2-23	*Lobaria pulmonaria*	11063	-16,8106138	28,32845477	Tenerife	Canary Isl.
ST2-23c	ST2	ST2-23	*Lobaria pulmonaria*	11064	-16,8106138	28,32845477	Tenerife	Canary Isl.
ST2-25a	ST2	ST2-25	*Lobaria pulmonaria*	11069	-16,81058987	28,32843802	Tenerife	Canary Isl.
ST2-25b	ST2	ST2-25	*Lobaria pulmonaria*	11070	-16,81058987	28,32843802	Tenerife	Canary Isl.
ST2-26a	ST2	ST2-26	*Lobaria pulmonaria*	11077	-16,81043884	28,32841651	Tenerife	Canary Isl.
ST2-26b	ST2	ST2-26	*Lobaria pulmonaria*	11078	-16,81043884	28,32841651	Tenerife	Canary Isl.
ST2-26c	ST2	ST2-26	*Lobaria pulmonaria*	11079	-16,81043884	28,32841651	Tenerife	Canary Isl.
ST2-26i	ST2	ST2-26	*Lobaria pulmonaria*	11085	-16,81043884	28,32841651	Tenerife	Canary Isl.
ST2-26j	ST2	ST2-26	*Lobaria pulmonaria*	11086	-16,81043884	28,32841651	Tenerife	Canary Isl.
ST2-27b	ST2	ST2-27	*Lobaria pulmonaria*	11088	-16,81033816	28,32840217	Tenerife	Canary Isl.
ST2-27c	ST2	ST2-27	*Lobaria pulmonaria*	11089	-16,81033816	28,32840217	Tenerife	Canary Isl.
ST2-07e	ST2	ST2-7	*Lobaria pulmonaria*	10983	-16,81052413	28,3288081	Tenerife	Canary Isl.
ST2-08d	ST2	ST2-8	*Lobaria pulmonaria*	10987	-16,81058851	28,32873812	Tenerife	Canary Isl.
ST3-01a	ST3	ST3-1	*Lobaria pulmonaria*	11323	-16,42443	28,40196	Tenerife	Canary Isl.
ST3-01b	ST3	ST3-1	*Lobaria pulmonaria*	11324	-16,42443	28,40196	Tenerife	Canary Isl.
ST3-01c	ST3	ST3-1	*Lobaria pulmonaria*	11325	-16,42443	28,40196	Tenerife	Canary Isl.
ST3-10a	ST3	ST3-10	*Lobaria pulmonaria*	11346	-16,42531084	28,40168362	Tenerife	Canary Isl.
ST3-10b	ST3	ST3-10	*Lobaria pulmonaria*	11347	-16,42531084	28,40168362	Tenerife	Canary Isl.
ST3-10c	ST3	ST3-10	*Lobaria pulmonaria*	11348	-16,42531084	28,40168362	Tenerife	Canary Isl.
ST3-11a	ST3	ST3-11	*Lobaria pulmonaria*	11349	-16,4253898	28,40174081	Tenerife	Canary Isl.
ST3-11b	ST3	ST3-11	*Lobaria pulmonaria*	11350	-16,4253898	28,40174081	Tenerife	Canary Isl.
ST3-11c	ST3	ST3-11	*Lobaria pulmonaria*	11351	-16,4253898	28,40174081	Tenerife	Canary Isl.
ST3-12a	ST3	ST3-12	*Lobaria pulmonaria*	11352	-16,42545447	28,40167099	Tenerife	Canary Isl.
ST3-12b	ST3	ST3-12	*Lobaria pulmonaria*	11353	-16,42545447	28,40167099	Tenerife	Canary Isl.

ID	Pop	PopTree	Species	VoucherID	X	Y	Location	Area
ST3-12c	ST3	ST3-12	*Lobaria pulmonaria*	11354	-16,42545447	28,40167099	Tenerife	Canary Isl.
ST3-13a	ST3	ST3-13	*Lobaria pulmonaria*	11355	-16,42555079	28,40170086	Tenerife	Canary Isl.
ST3-13b	ST3	ST3-13	*Lobaria pulmonaria*	11356	-16,42555079	28,40170086	Tenerife	Canary Isl.
ST3-13c	ST3	ST3-13	*Lobaria pulmonaria*	11357	-16,42555079	28,40170086	Tenerife	Canary Isl.
ST3-13d	ST3	ST3-13	*Lobaria pulmonaria*	11358	-16,42555079	28,40170086	Tenerife	Canary Isl.
ST3-14a	ST3	ST3-14	*Lobaria pulmonaria*	11359	-16,42555409	28,40168304	Tenerife	Canary Isl.
ST3-14b	ST3	ST3-14	*Lobaria pulmonaria*	11360	-16,42555409	28,40168304	Tenerife	Canary Isl.
ST3-14c	ST3	ST3-14	*Lobaria pulmonaria*	11361	-16,42555409	28,40168304	Tenerife	Canary Isl.
ST3-14d	ST3	ST3-14	*Lobaria pulmonaria*	11362	-16,42555409	28,40168304	Tenerife	Canary Isl.
ST3-15a	ST3	ST3-15	*Lobaria pulmonaria*	11363	-16,42556732	28,4016118	Tenerife	Canary Isl.
ST3-15b	ST3	ST3-15	*Lobaria pulmonaria*	11364	-16,42556732	28,4016118	Tenerife	Canary Isl.
ST3-15c	ST3	ST3-15	*Lobaria pulmonaria*	11365	-16,42556732	28,4016118	Tenerife	Canary Isl.
ST3-16a	ST3	ST3-16	*Lobaria pulmonaria*	11366	-16,42551696	28,40160449	Tenerife	Canary Isl.
ST3-16b	ST3	ST3-16	*Lobaria pulmonaria*	11367	-16,42551696	28,40160449	Tenerife	Canary Isl.
ST3-16c	ST3	ST3-16	*Lobaria pulmonaria*	11368	-16,42551696	28,40160449	Tenerife	Canary Isl.
ST3-17a	ST3	ST3-17	*Lobaria pulmonaria*	11369	-16,42556694	28,40152581	Tenerife	Canary Isl.
ST3-17b	ST3	ST3-17	*Lobaria pulmonaria*	11370	-16,42556694	28,40152581	Tenerife	Canary Isl.
ST3-17c	ST3	ST3-17	*Lobaria pulmonaria*	11371	-16,42556694	28,40152581	Tenerife	Canary Isl.
ST3-18a	ST3	ST3-18	*Lobaria pulmonaria*	11372	-16,4255309	28,40144137	Tenerife	Canary Isl.
ST3-18b	ST3	ST3-18	*Lobaria pulmonaria*	11373	-16,4255309	28,40144137	Tenerife	Canary Isl.
ST3-18c	ST3	ST3-18	*Lobaria pulmonaria*	11374	-16,4255309	28,40144137	Tenerife	Canary Isl.
ST3-19a	ST3	ST3-19	*Lobaria pulmonaria*	11375	-16,42556324	28,40140647	Tenerife	Canary Isl.
ST3-19b	ST3	ST3-19	*Lobaria pulmonaria*	11376	-16,42556324	28,40140647	Tenerife	Canary Isl.
ST3-19c	ST3	ST3-19	*Lobaria pulmonaria*	11377	-16,42556324	28,40140647	Tenerife	Canary Isl.
ST3-02a	ST3	ST3-2	*Lobaria pulmonaria*	11326	-16,42446234	28,4019251	Tenerife	Canary Isl.
ST3-02b	ST3	ST3-2	*Lobaria pulmonaria*	11327	-16,42446234	28,4019251	Tenerife	Canary Isl.
ST3-02c	ST3	ST3-2	*Lobaria pulmonaria*	11328	-16,42446234	28,4019251	Tenerife	Canary Isl.

ID	Pop	PopTree	Species	VoucherID	X	Y	Location	Area
ST3-20a	ST3	ST3-20	*Lobaria pulmonaria*	11378	-16,42568167	28,40149225	Tenerife	Canary Isl.
ST3-20b	ST3	ST3-20	*Lobaria pulmonaria*	11379	-16,42568167	28,40149225	Tenerife	Canary Isl.
ST3-20c	ST3	ST3-20	*Lobaria pulmonaria*	11380	-16,42568167	28,40149225	Tenerife	Canary Isl.
ST3-21a	ST3	ST3-21	*Lobaria pulmonaria*	11381	-16,42574635	28,40142243	Tenerife	Canary Isl.
ST3-21b	ST3	ST3-21	*Lobaria pulmonaria*	11382	-16,42574635	28,40142243	Tenerife	Canary Isl.
ST3-21c	ST3	ST3-21	*Lobaria pulmonaria*	11383	-16,42574635	28,40142243	Tenerife	Canary Isl.
ST3-03	ST3	ST3-3	*Lobaria pulmonaria*	11329	-16,42442927	28,40210322	Tenerife	Canary Isl.
ST3-04a	ST3	ST3-4	*Lobaria pulmonaria*	11330	-16,42471821	28,40219278	Tenerife	Canary Isl.
ST3-04b	ST3	ST3-4	*Lobaria pulmonaria*	11331	-16,42471821	28,40219278	Tenerife	Canary Isl.
ST3-05a	ST3	ST3-5	*Lobaria pulmonaria*	11332	-16,42496812	28,40179936	Tenerife	Canary Isl.
ST3-05b	ST3	ST3-5	*Lobaria pulmonaria*	11333	-16,42496812	28,40179936	Tenerife	Canary Isl.
ST3-06a	ST3	ST3-6	*Lobaria pulmonaria*	11334	-16,42517224	28,40179723	Tenerife	Canary Isl.
ST3-06b	ST3	ST3-6	*Lobaria pulmonaria*	11335	-16,42517224	28,40179723	Tenerife	Canary Isl.
ST3-06c	ST3	ST3-6	*Lobaria pulmonaria*	11336	-16,42517224	28,40179723	Tenerife	Canary Isl.
ST3-07a	ST3	ST3-7	*Lobaria pulmonaria*	11337	-16,42512486	28,40176291	Tenerife	Canary Isl.
ST3-07b	ST3	ST3-7	*Lobaria pulmonaria*	11338	-16,42512486	28,40176291	Tenerife	Canary Isl.
ST3-07c	ST3	ST3-7	*Lobaria pulmonaria*	11339	-16,42512486	28,40176291	Tenerife	Canary Isl.
ST3-08a	ST3	ST3-8	*Lobaria pulmonaria*	11340	-16,42522118	28,40179277	Tenerife	Canary Isl.
ST3-08b	ST3	ST3-8	*Lobaria pulmonaria*	11341	-16,42522118	28,40179277	Tenerife	Canary Isl.
ST3-08c	ST3	ST3-8	*Lobaria pulmonaria*	11342	-16,42522118	28,40179277	Tenerife	Canary Isl.
ST3-09a	ST3	ST3-9	*Lobaria pulmonaria*	11343	-16,42528585	28,40172296	Tenerife	Canary Isl.
ST3-09b	ST3	ST3-9	*Lobaria pulmonaria*	11344	-16,42528585	28,40172296	Tenerife	Canary Isl.
ST3-09c	ST3	ST3-9	*Lobaria pulmonaria*	11345	-16,42528585	28,40172296	Tenerife	Canary Isl.
ST4-10d	ST4	ST4-10	*Lobaria pulmonaria*	11414	-16,27077282	28,53977081	Tenerife	Canary Isl.
ST4-12f	ST4	ST4-12	*Lobaria pulmonaria*	11424	-16,2708188	28,53979247	Tenerife	Canary Isl.
ST4-13b	ST4	ST4-13	*Lobaria pulmonaria*	11426	-16,27083661	28,53980133	Tenerife	Canary Isl.
ST4-03b	ST4	ST4-3	*Lobaria pulmonaria*	11392	-16,27107156	28,53983079	Tenerife	Canary Isl.

**ID**	**Pop**	**PopTree**	**Species**	**VoucherID**	**X**	**Y**	**Location**	**Area**
ST4-04	ST4	ST4-4	*Lobaria pulmonaria*	11395	-16,27102044	28,5398313	Tenerife	Canary Isl.
ST5-11b	ST5	ST5-11	*Lobaria pulmonaria*	11511	-16,17684743	28,55783639	Tenerife	Canary Isl.
ST5-11c	ST5	ST5-11	*Lobaria pulmonaria*	11512	-16,17684743	28,55783639	Tenerife	Canary Isl.
ST5-11d	ST5	ST5-11	*Lobaria pulmonaria*	11513	-16,17684743	28,55783639	Tenerife	Canary Isl.
ST5-22f	ST5	ST5-22	*Lobaria pulmonaria*	11563	-16,17725476	28,55772451	Tenerife	Canary Isl.
ST5-26a	ST5	ST5-26	*Lobaria pulmonaria*	11579	-16,17746746	28,55761587	Tenerife	Canary Isl.
ST5-26b	ST5	ST5-26	*Lobaria pulmonaria*	11580	-16,17746746	28,55761587	Tenerife	Canary Isl.
TE1-01c	TE1	TE1-1	*Lobaria pulmonaria*	13945	-27,20365	38,75077	Terceira	Azores
TE1-10a	TE1	TE1-10	*Lobaria pulmonaria*	13982	-27,2037961	38,7508026	Terceira	Azores
TE1-10b	TE1	TE1-10	*Lobaria pulmonaria*	13983	-27,2037961	38,7508026	Terceira	Azores
TE1-10c	TE1	TE1-10	*Lobaria pulmonaria*	13984	-27,2037961	38,7508026	Terceira	Azores
TE1-10d	TE1	TE1-10	*Lobaria pulmonaria*	13985	-27,2037961	38,7508026	Terceira	Azores
TE1-11a	TE1	TE1-11	*Lobaria pulmonaria*	13986	-27,20379297	38,75080638	Terceira	Azores
TE1-11b	TE1	TE1-11	*Lobaria pulmonaria*	13987	-27,20379297	38,75080638	Terceira	Azores
TE1-12c	TE1	TE1-12	*Lobaria pulmonaria*	13990	-27,20368618	38,75077281	Terceira	Azores
TE1-03b	TE1	TE1-3	*Lobaria pulmonaria*	13951	-27,20361424	38,75073079	Terceira	Azores
TE1-03c	TE1	TE1-3	*Lobaria pulmonaria*	13952	-27,20361424	38,75073079	Terceira	Azores
TE1-03d	TE1	TE1-3	*Lobaria pulmonaria*	13953	-27,20361424	38,75073079	Terceira	Azores
TE1-04a	TE1	TE1-4	*Lobaria pulmonaria*	13955	-27,20361719	38,75072802	Terceira	Azores
TE1-04b	TE1	TE1-4	*Lobaria pulmonaria*	13956	-27,20361719	38,75072802	Terceira	Azores
TE1-04c	TE1	TE1-4	*Lobaria pulmonaria*	13957	-27,20361719	38,75072802	Terceira	Azores
TE1-04d	TE1	TE1-4	*Lobaria pulmonaria*	13958	-27,20361719	38,75072802	Terceira	Azores
TE1-06a	TE1	TE1-6	*Lobaria pulmonaria*	13964	-27,20382203	38,75078216	Terceira	Azores
TE1-06b	TE1	TE1-6	*Lobaria pulmonaria*	13965	-27,20382203	38,75078216	Terceira	Azores
TE1-06e	TE1	TE1-6	*Lobaria pulmonaria*	13968	-27,20382203	38,75078216	Terceira	Azores
TE1-06f	TE1	TE1-6	*Lobaria pulmonaria*	13969	-27,20382203	38,75078216	Terceira	Azores
TE1-06g	TE1	TE1-6	*Lobaria pulmonaria*	13970	-27,20382203	38,75078216	Terceira	Azores

ID	Pop	PopTree	Species	VoucherID	X	Y	Location	Area
TE1-07a	TE1	TE1-7	*Lobaria pulmonaria*	13973	-27,20374464	38,75079968	Terceira	Azores
TE1-07b	TE1	TE1-7	*Lobaria pulmonaria*	13974	-27,20374464	38,75079968	Terceira	Azores
TE1-07c	TE1	TE1-7	*Lobaria pulmonaria*	13975	-27,20374464	38,75079968	Terceira	Azores
TE1-09a	TE1	TE1-9	*Lobaria pulmonaria*	13979	-27,20379611	38,75080711	Terceira	Azores
TE1-09b	TE1	TE1-9	*Lobaria pulmonaria*	13980	-27,20379611	38,75080711	Terceira	Azores
TE1-09c	TE1	TE1-9	*Lobaria pulmonaria*	13981	-27,20379611	38,75080711	Terceira	Azores
TE2-11g	TE2	TE2-11	*Lobaria pulmonaria*	14181	-27,20876248	38,73381349	Terceira	Azores
TE2-13a	TE2	TE2-13	*Lobaria pulmonaria*	14186	-27,2087194	38,73380699	Terceira	Azores
TE2-13b	TE2	TE2-13	*Lobaria pulmonaria*	14187	-27,2087194	38,73380699	Terceira	Azores
TE2-14a	TE2	TE2-14	*Lobaria pulmonaria*	14189	-27,20869103	38,73379155	Terceira	Azores
TE2-15a	TE2	TE2-15	*Lobaria pulmonaria*	14194	-27,20874808	38,73378514	Terceira	Azores
TE2-15b	TE2	TE2-15	*Lobaria pulmonaria*	14195	-27,20874808	38,73378514	Terceira	Azores
TE2-15d	TE2	TE2-15	*Lobaria pulmonaria*	14197	-27,20874808	38,73378514	Terceira	Azores
TE2-16b	TE2	TE2-16	*Lobaria pulmonaria*	14199	-27,20874574	38,73378529	Terceira	Azores
TE2-18a	TE2	TE2-18	*Lobaria pulmonaria*	14202	-27,20865356	38,73381011	Terceira	Azores
TE2-19d	TE2	TE2-19	*Lobaria pulmonaria*	14208	-27,20865438	38,73380948	Terceira	Azores
TE2-02a	TE2	TE2-2	*Lobaria pulmonaria*	14139	-27,20885247	38,7338201	Terceira	Azores
TE2-02b	TE2	TE2-2	*Lobaria pulmonaria*	14140	-27,20885247	38,7338201	Terceira	Azores
TE2-20a	TE2	TE2-20	*Lobaria pulmonaria*	14209	-27,20865626	38,7338105	Terceira	Azores
TE2-20b	TE2	TE2-20	*Lobaria pulmonaria*	14210	-27,20865626	38,7338105	Terceira	Azores
TE2-20c	TE2	TE2-20	*Lobaria pulmonaria*	14211	-27,20865626	38,7338105	Terceira	Azores
TE2-21a	TE2	TE2-21	*Lobaria pulmonaria*	14213	-27,20864143	38,73379672	Terceira	Azores
TE2-21b	TE2	TE2-21	*Lobaria pulmonaria*	14214	-27,20864143	38,73379672	Terceira	Azores
TE2-21c	TE2	TE2-21	*Lobaria pulmonaria*	14215	-27,20864143	38,73379672	Terceira	Azores
TE2-23c	TE2	TE2-23	*Lobaria pulmonaria*	14222	-27,2085661	38,73380735	Terceira	Azores
TE2-25b	TE2	TE2-25	*Lobaria pulmonaria*	14230	-27,20847939	38,73385963	Terceira	Azores
TE2-25e	TE2	TE2-25	*Lobaria pulmonaria*	14001	-27,20847939	38,73385963	Terceira	Azores

ID	Pop	PopTree	Species	VoucherID	X	Y	Location	Area
TE2-26c	TE2	TE2-26	*Lobaria pulmonaria*	14008	-27,20846173	38,73384807	Terceira	Azores
TE2-27a	TE2	TE2-27	*Lobaria pulmonaria*	14009	-27,20846955	38,73383112	Terceira	Azores
TE2-33b	TE2	TE2-33	*Lobaria pulmonaria*	14029	-27,20793858	38,73377472	Terceira	Azores
TE2-33d	TE2	TE2-33	*Lobaria pulmonaria*	14031	-27,20793858	38,73377472	Terceira	Azores
TE2-05a	TE2	TE2-5	*Lobaria pulmonaria*	14148	-27,20869825	38,73378956	Terceira	Azores
TE2-05b	TE2	TE2-5	*Lobaria pulmonaria*	14149	-27,20869825	38,73378956	Terceira	Azores
TE2-06a	TE2	TE2-6	*Lobaria pulmonaria*	14154	-27,20873377	38,733825	Terceira	Azores
TE2-06b	TE2	TE2-6	*Lobaria pulmonaria*	14155	-27,20873377	38,733825	Terceira	Azores
TE2-06c	TE2	TE2-6	*Lobaria pulmonaria*	14156	-27,20873377	38,733825	Terceira	Azores
TE2-06d	TE2	TE2-6	*Lobaria pulmonaria*	14157	-27,20873377	38,733825	Terceira	Azores
TE2-07a	TE2	TE2-7	*Lobaria pulmonaria*	14159	-27,20872619	38,73382285	Terceira	Azores
TE2-08a	TE2	TE2-8	*Lobaria pulmonaria*	14162	-27,20873537	38,73382234	Terceira	Azores
TE2-09a	TE2	TE2-9	*Lobaria pulmonaria*	14167	-27,20874093	38,73382116	Terceira	Azores
TE3-01a	TE3	TE3-1	*Lobaria pulmonaria*	14036	-27,2791	38,71999	Terceira	Azores
TE3-02a	TE3	TE3-2	*Lobaria pulmonaria*	14037	-27,27908374	38,71991904	Terceira	Azores
TE3-02b	TE3	TE3-2	*Lobaria pulmonaria*	14038	-27,27908374	38,71991904	Terceira	Azores
TE3-02c	TE3	TE3-2	*Lobaria pulmonaria*	14039	-27,27908374	38,71991904	Terceira	Azores
TE3-02d	TE3	TE3-2	*Lobaria pulmonaria*	14040	-27,27908374	38,71991904	Terceira	Azores
TE3-02e	TE3	TE3-2	*Lobaria pulmonaria*	14041	-27,27908374	38,71991904	Terceira	Azores
TE3-02f	TE3	TE3-2	*Lobaria pulmonaria*	14042	-27,27908374	38,71991904	Terceira	Azores
TE3-03a	TE3	TE3-3	*Lobaria pulmonaria*	14043	-27,27901477	38,71992109	Terceira	Azores
TE3-03b	TE3	TE3-3	*Lobaria pulmonaria*	14044	-27,27901477	38,71992109	Terceira	Azores
TE3-03c	TE3	TE3-3	*Lobaria pulmonaria*	14045	-27,27901477	38,71992109	Terceira	Azores
TE3-03d	TE3	TE3-3	*Lobaria pulmonaria*	14046	-27,27901477	38,71992109	Terceira	Azores
TE3-04b	TE3	TE3-4	*Lobaria pulmonaria*	14048	-27,27895746	38,7199173	Terceira	Azores
TE3-04c	TE3	TE3-4	*Lobaria pulmonaria*	14049	-27,27895746	38,7199173	Terceira	Azores
TE3-05a	TE3	TE3-5	*Lobaria pulmonaria*	14050	-27,27895746	38,7199173	Terceira	Azores

ID	Pop	PopTree	Species	VoucherID	X	Y	Location	Area
TE3-05b	TE3	TE3-5	*Lobaria pulmonaria*	14051	-27,27895746	38,7199173	Terceira	Azores
TE3-06a	TE3	TE3-6	*Lobaria pulmonaria*	14052	-27,27895286	38,71997125	Terceira	Azores
TE3-06c	TE3	TE3-6	*Lobaria pulmonaria*	14054	-27,27895286	38,71997125	Terceira	Azores
TE3-06d	TE3	TE3-6	*Lobaria pulmonaria*	14055	-27,27895286	38,71997125	Terceira	Azores
TE3-06e	TE3	TE3-6	*Lobaria pulmonaria*	14056	-27,27895286	38,71997125	Terceira	Azores
TE3-06f	TE3	TE3-6	*Lobaria pulmonaria*	14057	-27,27895286	38,71997125	Terceira	Azores
TE3-07a	TE3	TE3-7	*Lobaria pulmonaria*	14059	-27,27879888	38,71991025	Terceira	Azores
TE3-07b	TE3	TE3-7	*Lobaria pulmonaria*	14060	-27,27879888	38,71991025	Terceira	Azores
TE3-08a	TE3	TE3-8	*Lobaria pulmonaria*	14062	-27,27888955	38,71992255	Terceira	Azores
TE4-01a	TE4	TE4-1	*Lobaria pulmonaria*	14063	-27,23517	38,69481	Terceira	Azores
TE4-10a	TE4	TE4-10	*Lobaria pulmonaria*	14104	-27,23502367	38,69494484	Terceira	Azores
TE4-10b	TE4	TE4-10	*Lobaria pulmonaria*	14105	-27,23502367	38,69494484	Terceira	Azores
TE4-10c	TE4	TE4-10	*Lobaria pulmonaria*	14106	-27,23502367	38,69494484	Terceira	Azores
TE4-10d	TE4	TE4-10	*Lobaria pulmonaria*	14107	-27,23502367	38,69494484	Terceira	Azores
TE4-10e	TE4	TE4-10	*Lobaria pulmonaria*	14108	-27,23502367	38,69494484	Terceira	Azores
TE4-10f	TE4	TE4-10	*Lobaria pulmonaria*	14109	-27,23502367	38,69494484	Terceira	Azores
TE4-10g	TE4	TE4-10	*Lobaria pulmonaria*	14110	-27,23502367	38,69494484	Terceira	Azores
TE4-11a	TE4	TE4-11	*Lobaria pulmonaria*	14111	-27,23502161	38,69492689	Terceira	Azores
TE4-11b	TE4	TE4-11	*Lobaria pulmonaria*	14112	-27,23502161	38,69492689	Terceira	Azores
TE4-11c	TE4	TE4-11	*Lobaria pulmonaria*	14113	-27,23502161	38,69492689	Terceira	Azores
TE4-11d	TE4	TE4-11	*Lobaria pulmonaria*	14114	-27,23502161	38,69492689	Terceira	Azores
TE4-11e	TE4	TE4-11	*Lobaria pulmonaria*	14115	-27,23502161	38,69492689	Terceira	Azores
TE4-11f	TE4	TE4-11	*Lobaria pulmonaria*	14116	-27,23502161	38,69492689	Terceira	Azores
TE4-11g	TE4	TE4-11	*Lobaria pulmonaria*	14117	-27,23502161	38,69492689	Terceira	Azores
TE4-12a	TE4	TE4-12	*Lobaria pulmonaria*	14118	-27,23501144	38,69491597	Terceira	Azores
TE4-12b	TE4	TE4-12	*Lobaria pulmonaria*	14119	-27,23501144	38,69491597	Terceira	Azores
TE4-12c	TE4	TE4-12	*Lobaria pulmonaria*	14120	-27,23501144	38,69491597	Terceira	Azores

ID	Pop	PopTree	Species	VoucherID	X	Y	Location	Area
TE4-13d	TE4	TE4-13	*Lobaria pulmonaria*	14125	-27,235099	38,69493808	Terceira	Azores
TE4-13h	TE4	TE4-13	*Lobaria pulmonaria*	14129	-27,235099	38,69493808	Terceira	Azores
TE4-03b	TE4	TE4-3	*Lobaria pulmonaria*	14071	-27,23520697	38,69485488	Terceira	Azores
TE4-03d	TE4	TE4-3	*Lobaria pulmonaria*	14073	-27,23520697	38,69485488	Terceira	Azores
TE4-05e	TE4	TE4-5	*Lobaria pulmonaria*	14085	-27,23526088	38,69490229	Terceira	Azores
TE4-06e	TE4	TE4-6	*Lobaria pulmonaria*	14090	-27,23521386	38,69492823	Terceira	Azores
TN-01a	TN	TN-1	*Lobaria pulmonaria*	18462	-53,97149284	48,55771111	Terra Nova	Canada
TN-01b	TN	TN-1	*Lobaria pulmonaria*	18463	-53,97149284	48,55771111	Terra Nova	Canada
TN-01c	TN	TN-1	*Lobaria pulmonaria*	18464	-53,97149284	48,55771111	Terra Nova	Canada
TN-15a	TN	TN-15	*Lobaria pulmonaria*	18487	-53,97155486	48,55759839	Terra Nova	Canada
TN-15b	TN	TN-15	*Lobaria pulmonaria*	18488	-53,97155486	48,55759839	Terra Nova	Canada
TN-15c	TN	TN-15	*Lobaria pulmonaria*	18489	-53,97155486	48,55759839	Terra Nova	Canada
TN-22a	TN	TN-22	*Lobaria pulmonaria*	18501	-53,97160351	48,55777133	Terra Nova	Canada
TN-22b	TN	TN-22	*Lobaria pulmonaria*	18502	-53,97160351	48,55777133	Terra Nova	Canada
TN-22c	TN	TN-22	*Lobaria pulmonaria*	18503	-53,97160351	48,55777133	Terra Nova	Canada
TN-27a	TN	TN-27	*Lobaria pulmonaria*	18512	-53,97173127	48,55827568	Terra Nova	Canada
TN-27b	TN	TN-27	*Lobaria pulmonaria*	18513	-53,97173127	48,55827568	Terra Nova	Canada
TN-27c	TN	TN-27	*Lobaria pulmonaria*	18514	-53,97173127	48,55827568	Terra Nova	Canada
TN-30a	TN	TN-30	*Lobaria pulmonaria*	18518	-53,972106	48,55846845	Terra Nova	Canada
TN-30b	TN	TN-30	*Lobaria pulmonaria*	18519	-53,972106	48,55846845	Terra Nova	Canada
TN-30c	TN	TN-30	*Lobaria pulmonaria*	18520	-53,972106	48,55846845	Terra Nova	Canada
TN-32a	TN	TN-32	*Lobaria pulmonaria*	18522	-53,97311651	48,55823449	Terra Nova	Canada
TN-32b	TN	TN-32	*Lobaria pulmonaria*	18523	-53,97311651	48,55823449	Terra Nova	Canada
TN-32c	TN	TN-32	*Lobaria pulmonaria*	18524	-53,97311651	48,55823449	Terra Nova	Canada
TN-34a	TN	TN-34	*Lobaria pulmonaria*	18526	-53,97433459	48,558203	Terra Nova	Canada
TN-34b	TN	TN-34	*Lobaria pulmonaria*	18527	-53,97433459	48,558203	Terra Nova	Canada
TN-34c	TN	TN-34	*Lobaria pulmonaria*	18528	-53,97433459	48,558203	Terra Nova	Canada

ID	Pop	PopTree	Species	VoucherID	X	Y	Location	Area
TN-41a	TN	TN-41	*Lobaria pulmonaria*	18540	-53,97853081	48,5574564	Terra Nova	Canada
TN-41b	TN	TN-41	*Lobaria pulmonaria*	18541	-53,97853081	48,5574564	Terra Nova	Canada
TN-41c	TN	TN-41	*Lobaria pulmonaria*	18542	-53,97853081	48,5574564	Terra Nova	Canada
TN-45a	TN	TN-45	*Lobaria pulmonaria*	18548	-53,98106165	48,55739084	Terra Nova	Canada
